# A methodological approach to correlate tumor heterogeneity with drug distribution profile in mass spectrometry imaging data

**DOI:** 10.1093/gigascience/giaa131

**Published:** 2020-11-25

**Authors:** Mridula Prasad, Geert Postma, Pietro Franceschi, Lavinia Morosi, Silvia Giordano, Francesca Falcetta, Raffaella Giavazzi, Enrico Davoli, Lutgarde M C Buydens, Jeroen Jansen

**Affiliations:** IMM/ Analytical Chemistry, Radboud University, Heyendaalseweg, 6525 AJ Nijmegen, Netherlands; Unit of Computational Biology, Research and Innovation Center, Fondazione Edmund Mach, 38010 San Michele all’ Adige, Italy; IMM/ Analytical Chemistry, Radboud University, Heyendaalseweg, 6525 AJ Nijmegen, Netherlands; Unit of Computational Biology, Research and Innovation Center, Fondazione Edmund Mach, 38010 San Michele all’ Adige, Italy; Department of Oncology, Istituto di Ricerche Farmacologiche Mario Negri IRCCS, Via La Masa, 19-20156 Milan, Italy; Mass Spectrometry Laboratory, Istituto di Ricerche Farmacologiche Mario Negri IRCCS, Via La Masa, 19-20156 Milan, Italy; Department of Oncology, Istituto di Ricerche Farmacologiche Mario Negri IRCCS, Via La Masa, 19-20156 Milan, Italy; Department of Oncology, Istituto di Ricerche Farmacologiche Mario Negri IRCCS, Via La Masa, 19-20156 Milan, Italy; Mass Spectrometry Laboratory, Istituto di Ricerche Farmacologiche Mario Negri IRCCS, Via La Masa, 19-20156 Milan, Italy; IMM/ Analytical Chemistry, Radboud University, Heyendaalseweg, 6525 AJ Nijmegen, Netherlands; IMM/ Analytical Chemistry, Radboud University, Heyendaalseweg, 6525 AJ Nijmegen, Netherlands

**Keywords:** Mass spectrometry imaging, drug distribution, tumor heterogeneity, spatial methods

## Abstract

**Background:**

Drug mass spectrometry imaging (MSI) data contain knowledge about drug and several other molecular ions present in a biological sample. However, a proper approach to fully explore the potential of such type of data is still missing. Therefore, a computational pipeline that combines different spatial and non-spatial methods is proposed to link the observed drug distribution profile with tumor heterogeneity in solid tumor. Our data analysis steps include pre-processing of MSI data, cluster analysis, drug local indicators of spatial association (LISA) map, and ions selection.

**Results:**

The number of clusters identified from different tumor tissues. The spatial homogeneity of the individual cluster was measured using a modified version of our drug homogeneity method. The clustered image and drug LISA map were simultaneously analyzed to link identified clusters with observed drug distribution profile. Finally, ions selection was performed using the spatially aware method.

**Conclusions:**

In this paper, we have shown an approach to correlate the drug distribution with spatial heterogeneity in untargeted MSI data. Our approach is freely available in an R package 'CorrDrugTumorMSI'.

## Introduction

Heterogeneity of the tumor microenvironment directly affects drug penetration, reducing therapeutic efficacy and contributing to the development of resistance [1, 2]. For these reasons, knowledge about tumor spatial heterogeneity is of paramount importance to optimize therapeutic outcomes [3]. To elucidate the relation between drug distribution and tissue homogeneity/heterogeneity, an imaging technique able to investigate both phenomena simultaneously would be desirable [4].

Mass spectrometry imaging (MSI) is a molecular imaging technique that provides simultaneous information about spatial localization of drugs and other small molecules present in the biological sample [5, 6]. It is a valuable technique to characterize tumor tissue subtypes [7–9], and it is also applied to map the distribution of drugs within the tissue [10–13]. Untargeted MSI datasets provide unprecedented opportunities to understand drug distribution in association with tissue heterogeneity. Several computational algorithms have been implemented on MSI data for the identification of tumor tissue areas [7, 8, 14] and selection of important molecular ions [8, 9, 15]. To our knowledge no previous work combines tumor heterogeneity with a drug distribution profile. In a recent article, we proposed a new objective index (Drug Homogeneity Index [DHI]) [16] to measure drug homogeneity in different MSI datasets, and we aim to extend this work, proposing a framework that can be used to investigate the relation between drug homogeneity and the observed tumor heterogeneity. The proposed approach encompasses the following steps.

1. Identification of the different tissue subpopulations from an untargeted multisample MSI dataset (segmentation).
2. Quantitative analysis of the spatial arrangement of the tissue subtypes across different samples.
3. Extraction of discrete drug distribution maps.
4. Matching of the segmented MSI dataset with the drug distribution maps.
5. Selection of ion signals that can be used to differentiate specific tissue areas.

Because the accessibility of a drug to a particular spatial location is expected to be linked to the metabolic and histological characteristics of the underlying tissues, multivariate unsupervised clustering was used to group the pixels into a limited number of groups that represent the different tissue subtypes [8, 9, 14, 17–19] (Step 1). This step was performed after removing the drug-related peaks to avoid their influence in group formation. The quantitative assessment of the spatial arrangement of the different clusters (Step 2) was performed by using a modified version of our DHI [16], which was optimized to characterize the spatial arrangement of the pixels on the clustered images. The local indicators of spatial association (LISA) [20, 21] method was instead applied to create discrete drug distribution maps (Step 3). The association between LISA maps and identified segments was derived using the Cramer [22] V method (Step 4). The identification of important ion signals able to differentiate the tissue subtypes was finally performed by using a spatial-aware statistical method that corrects for spatial autocorrelation [23] (Step 5). Because the application of these models in MSI is still uncommon, the optimal spatial model for biomarker selection was tested on a synthetic autocorrelated spatial dataset.

In our study, the identification of tissue subpopulations was performed using *k*-means clustering with correlation distance. This non-spatial clustering method has already been used in other MSI research [17, 24, 25] and was able to efficiently extract relevant structures. The spatially relevant clusters in drug ion images were detected on the basis of a Moran scatter plot or LISA map. The LISA map is a commonly used method in spatial statistics in which pixels are grouped into different clusters (zones) based on their similar or different behavior with neighboring pixels [26, 27]. The potential of the proposed approach was tested on the same datasets that we used in our previous work [16, 28], which consisted of the MSI untargeted analysis of a set of different tumor xenograft models from ovarian (A2780) and colon (HCT116) cancer cell lines. The study was designed to assess the effect of an anti-angiogenesis compound (bevacizumab) on the drug (paclitaxel) distribution [28]. For this purpose, half of the animals were pretreated twice (5 and 1 days before excision) with bevacizumab, before being administered paclitaxel a few hours (6) before excision. Even if it is impossible to exclude that the anticancer drug could be responsible for the observed spatial heterogeneity in metabolic profile [29], any relevant difference between the 2 treatment groups is likely to be associated with the direct or indirect effect of bevacizumab.

## Experimental section

### MSI datasets

MSI data were derived from tumor-bearing mice treated with paclitaxel (60 mg/kg) alone or in combination with bevacizumab (2 intraperitoneal injections at 150 μg per mouse). For the mice experiment, Istituto di Ricerche Farmacologiche Mario Negri adhered to the principles set out in the following laws, regulations, and policies governing the care and use of laboratory animals: Italian Governing Law (D.lgs 26/2014; Authorization n.19/2008-A issued 6 March 2008 by Ministry of Health); Mario Negri Institutional Regulations and Policies providing internal authorization for persons conducting animal experiments (Quality Management System Certificate–UNI EN ISO 9001:2008–Reg. No. 6121); the NIH Guide for the Care and Use of Laboratory Animals (2011 edition), and EU directives and guidelines (EEC Council Directive 2010/63/UE).

The complete details of the experiments have been previously described [12, 16, 28]. Briefly, tumors were collected, frozen in liquid nitrogen, and prepared for MSI analysis. A matrix-assisted laser desorption/ionization (MALDI) 4800 TOF-TOF (AB SCIEX, Framingham, MA) was used, and mass spectra were recorded in full-scan-profile mode over a limited mass range (*m*/*z* 199–500). Images of tissue sections were acquired using the 4800 Imaging Tool software with an imaging raster of 100  ×  100  μm (pixel dimension of ∼0.01 mm^2^). The obtained dataset consisted of 131,349 (A2780) and 59,652 (HCT116) raw spectra from the tissue areas of all tumor models.

### MSI data pre-processing

The initial data files in Analyze 7.5 format were opened in R [30] free software version 3.4.3 using the MALDIquant [31] package. Each MSI datafile contains mass spectra collected from both tumor tissue and a glass slide. To avoid any bias in pre-processing and data analysis steps, the mass spectra belonging to tumor tissue only were used. The identification of tumor tissue was performed by constructing a mask of the ion signal detected in the *m*/*z* = 281.1–281.44 range. This ion was confirmed to be a reliable tissue marker by visually comparing all MS images with their optical counterparts. To correct for possible spectral misalignments across the different datasets, an adaptive binning approach was applied. To do this, first, a reference spectrum was created, which is a single maximum-intensity spectrum of all the spectra. The main reasoning behind it is to acquire knowledge about the location and shape of all the ion peaks in our data and then optimize the bin size accordingly. Smoothing of the reference spectrum was performed by applying discrete wavelet transformation (Daubechies least asymmetric 8-tap filter with hard shrinkage) using the msDenoiseWavelet function from the msProcess [32] R package. Peaks in the reference spectrum were identified by local maxima search above a certain threshold [16]. Initial bins were created on the basis of those identified peaks. The bin size was further optimized on the bases of instrument characteristics merging together peaks showing a difference of <0.05*m*/*z*. To avoid missing peaks in low-intensity spectra, an extra bin of size 0.5 was added in the presence of a large gap between 2 identified peaks (especially towards the end of the spectra). The complete workflow of bin creation and maximum-intensity spectra from the MSI data of 2 tumors are shown in Additional File 1.

After bin identification, each MSI data file was reopened and peak picking was performed using an approach similar to the one applied to the reference spectrum, and the identified peaks were assigned to their corresponding bins. To focus only on the more common ions, peaks that were present in <20% of the tissue area were removed. Peaks only present in a single tumor model were rejected. To remove spatial noise, median filtering with a window size of 3 × 3 pixels was performed on each extracted ion image. We also performed edge correction in the individual dataset because marginal pixels have enormously high intensity due to the surface difference between tissue and glass slide [33]. image. To make the spectra collected on the different pixels comparable median, normalization was performed. Generalized log transformation was performed as a variance stabilization step using LMGene [34] in R. A plausible batch effect between the slices of certain mouse models was removed using the removeBatchEffect function from the R limma [35] package. Ion peaks with correlation with the drug compound (paclitaxel) (>0.9) were removed before cluster analysis. The overall dimensions of the final data matrix belonging to A2780 and HCT116 are equal to 131,349 × 173 and 59,652 × 155, respectively.

### MSI data analysis

#### Unsupervised data clustering

Segmentation of MSI data collected on the complete set of sections from a specific cell line was performed by unsupervised bisecting *k*-means clustering [36] using correlation as a similarity measure. The individual clusters were allowed to split further until the largest cluster contained 40% of the pixels included in the initial data matrix. The number of clusters at each step was selected on the basis of the calinhara internal validity index [37]. The clustering and validation were implemented using R packages amap [38] and fpc [39], respectively. The outcomes of segmentation for the different sample groups (cell lines and bevacizumab treatment) were analyzed on the basis of the following parameters: (i) size and (ii) homogeneity of the individual clusters. The homogeneity of the individual clusters was assessed by using a slightly modified version of the DHI (see Additional File 2). Higher cluster homogeneity means the more continuous/homogeneous distribution of a particular tissue type. All these parameter values were normalized by the total number of pixels from the tumor models of each treatment condition. Statistical significance of pixel and homogeneity ratio under 2 treatment conditions was calculated using linear mixed models with the nlme [40] R package.

#### Quantification assessment of the drug distribution

Spatial quantification of the drug heterogeneity was performed using the Moran I scatter plot [23], also known as the LISA map (see Additional File 2). This spatial-aware method was selected because it is expected to yield more robust results in the presence of the spatially autocorrelated drug signal.

**Figure 1:**
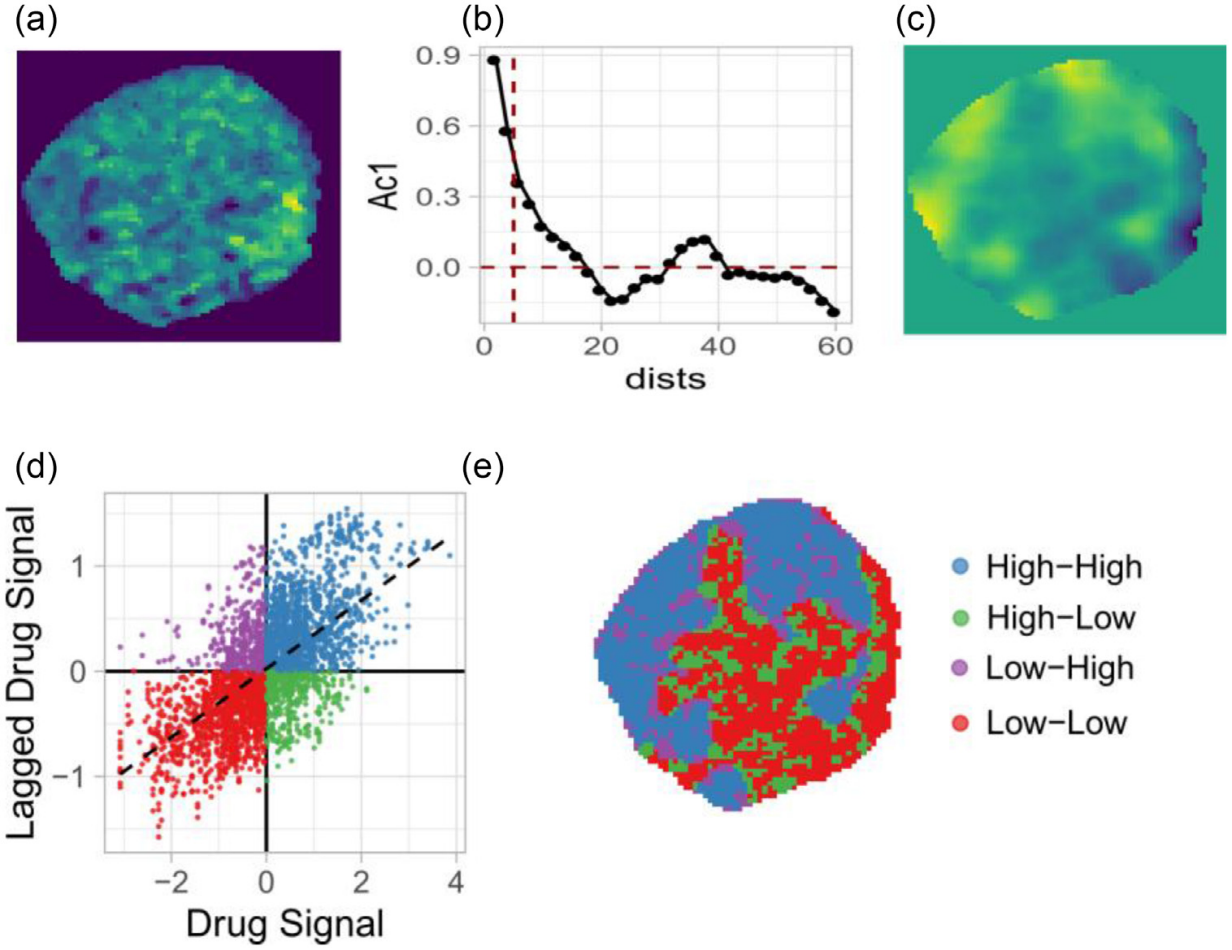
Schematic workflow of drug LISA map creation. (a) Original drug ion image. (b) Spatial correlogram of the drug-ion image, where Moran I values (Ac1) are plotted against the lag distance (dists). (c) Spatially lagged image of the drug-ion. (d) Moran I scatter plot, where the drug signal and its spatially lagged version are regressed against each other. (e) LISA clustered map of the drug ion where pixels falling in the same quadrant of the Moran scatter plot are grouped.

To create a Moran I scatter plot and/or LISA map the required inputs are as follows: original variable, spatially lagged variable, and spatial weight matrix. The original variable in our case is the ion intensity map of the drug peak. The spatially lagged variable is constructed by multiplying the autoscaled version of the original variable with the help of the spatial weight matrix [23, 41]. This weight matrix stores the connections between nearby observations (e.g., in a binary weight matrix the observations that lie within a certain range of autocorrelation receive a value of 1, else 0). The optimal range of autocorrelation can be decided on the basis of a spatial correlogram. A spatial correlogram [23] is a 2D plot where the spatial autocorrelation index (Moran I) is plotted as a function of lag distance, where a positive value indicates the presence of autocorrelation within a certain distance range. To create a LISA map of a drug ion image the following steps were performed:

- the optimal spatial weight matrix was created on the basis of the spatial correlogram plot (Fig. 1b).
- the original variable was converted into its spatially lagged version (Fig. 1c).
- Moran I scatter plot was created by regressing the original variable against its spatially lagged version, where pixels are grouped into 4 different zones usually called high-high, low-low, high-low, and low-high (Fig. 1d).
- Finally, a LISA map was constructed, which is a 2D image where pixels are labeled according to their class in the Moran I scatter plot (Fig. 1e).

In a LISA map or Moran I scatter plot, the high-high (HH) zone contains pixels that have a high intensity or above-average value and are surrounded by a similar type of high-intensity pixels. The low-low (LL) zone contains pixels that have a lower intensity or below-average value and are surrounded by a similar type of low-intensity pixels. The high-low (HL) zone contains pixels that have above-average value themselves but are surrounded by neighbors with below-average value, and vice versa for the pixels that fall in the low-high (LH) zone. Note, in a LISA map pixels falling in the HH and LL zones show positive spatial autocorrelation and are spatially smooth. Therefore, a single zone of a LISA map may contain multiple clusters with approximately similar profiles.

#### Association between clustered image and drug LISA map

The obtained drug LISA maps were analyzed for their association with the unsupervised clusters obtained using unsupervised clustering. A quantitative analysis was performed to elucidate which cluster subtypes overlap most with which zone of the LISA map, for which the fraction of pixels in different zones and clusters in each tumor model was calculated. Furthermore, the strength of association between those 2 vector classes (LISA map zones and unsupervised clusters) was estimated using the Cramer V [22] method. Cramer V is a statistical measure similar to the Pearson correlation to find the correlation between 2 nominal variables and returns a correlation value within the range of 0–1.

#### Representative ion signal selection from the identified clusters

The method used to select representative ion signals from the identified clusters in the MSI data was first validated on synthetic spatially autocorrelated data. Two spatial approaches (spatial error model [SE] and spatial lag model [SL]) were compared with a standard non-spatial approach (ordinary least squares [OLS]). Both spatial models [23, 42] are modified versions of an OLS model and include spatial autocorrelation in a different component of the OLS model. The comparison of the performance of the aforementioned statistical methods with complete description of the synthetic data generation process is given in Additional File 3.

All spatial models were fitted with the spdep [23] R package. Similar to what was done in the case of the LISA map, the right threshold for the spatial weight matrix was decided on the basis of the spatial correlogram.

In MSI data, the selection of ion signals from identified clusters was performed using the method that gives the best performance on our synthetic data. To do that, the outcomes of the original clustering were converted into a set of 2-class images where each cluster is, in turn, compared with all the others. Because variable selection using the spatial method is computationally intensive, we only used a few tumor models in which the cluster of interest was present. Thus, per cluster 5 different tumor slices were selected, i.e., MSI data from 4–5 different tumor models. If a particular ion was found to be important in all 5 datasets, then it was considered as a significant ion signal for the respective cluster. The important ions were selected on the bases of the model *P*-values corrected for multiple testing by using the procedure of Benjamini and Hochberg [43].

## Results

### Unsupervised clustering of MSI data

#### A2780 cell-line–based MSI data

**Figure 2:**
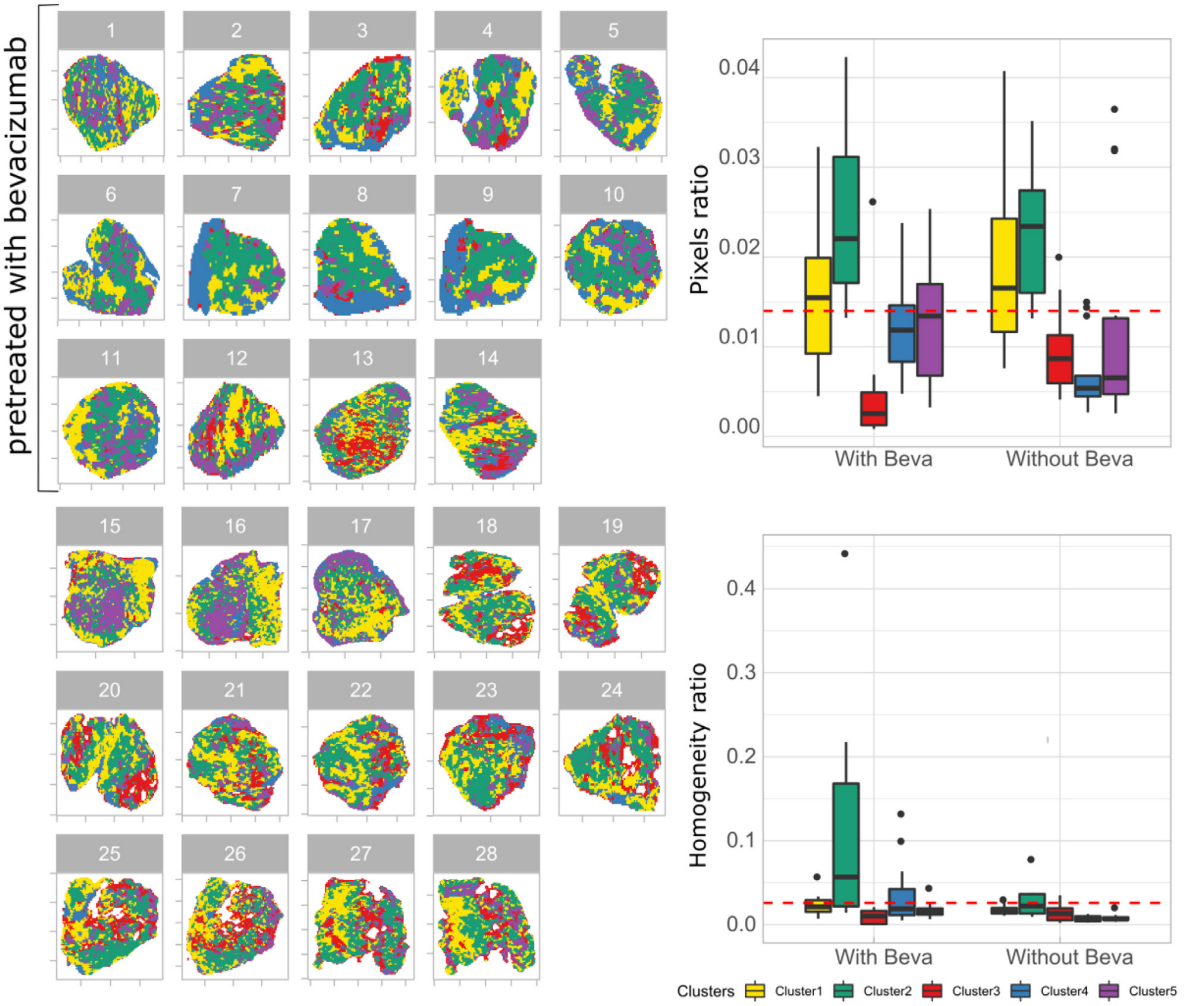
Cluster analysis of A2780 tumor MSI data generated in the presence and absence of bevacizumab treatment. Left: Representation of clusters detected by the *k*-means method. Right: Boxplots show the ratio of (top) pixels and (bottom) homogeneity calculated from individual clusters under 2 treatment conditions. The red horizontal line indicates the global mean value of pixel and homogeneity ratio. Here, pixel ratio = number of pixels in individual clusters/total number of pixels from all tumor MSI data under particular treatment conditions. Homogeneity ratio = size-zone of individual clusters for a given tumor model/total number of pixels in that particular tumor model. With beva: pretreated with bevacizumab; without beva: without bevacizumab pretreatment. Clusters: different clusters identified in A2780 tumor MSI data.

The clustering method identified 5 unique clusters in the combined set of A2780 xenograft models (Fig. 2, left). The majority of the replicates possess the 5 clusters in different ratios, whereas Clusters 1 and 2 were predominant in all tumor models in both treatment conditions. The relative contribution of Cluster 3 is reduced and Cluster 4 is enhanced in the presence of bevacizumab (Fig. 2, top right). Cluster 3 showed a high overlap with the necrosis area [12] and was noted to be present in a relatively higher fraction among the tumor models not pretreated with bevacizumab. A small fraction of Cluster 5 is present in all tumor models.

Similar to the number of pixels, the homogeneity of clusters (parameter ii) for the individual tumors was calculated using the modified version of our DHI and is shown in Fig. 2, bottom right. The figure highlights the clear difference in cluster homogeneity under the 2 treatment conditions, especially for Cluster 2. The homogeneity of Cluster 2 in the presence of bevacizumab treatment is much higher than the homogeneity of any other cluster in either treatment condition. Without bevacizumab treatment tumor models also show high homogeneity for Cluster 2 followed by Cluster 3.

#### HCT116 cell-line–based MSI data

Five clusters were identified in the HCT116 tumor cell line MSI data (see Additional File 4). Similar to the A2780–1A9 tumor MSI data, there was not a large observed difference in cluster population under the 2 treatment conditions. Clusters 2 and 3 were predominantly present in all tumor models irrespective of treatment condition (see Additional File 4 Fig. S1, top right). Cluster 1 was observed in a moderate amount and very small fractions of Clusters 4 and 5 were present in all tumor models. The homogeneity assessment of the individual clusters in the 2 treatment conditions shows that Cluster 3 has high homogeneity in the case of bevacizumab treatment. In the absence of bevacizumab treatment, Clusters 2 and 3 show more homogeneity (see Additional File 4 Fig. S1, bottom right).

The statistical analysis of pixel and homogeneity ratio values was performed using a linear mixed model approach where range and *P*-value from both tumor models are given in Additional File 4 Table S1. For the A2780–1A9 MSI data, the pixel ratios are not significantly different in the 2 treatment conditions. The homogeneity value of Cluster 2 is close to significant, which is derived with parameter Nu = 5 in our homogeneity formula. In HCT116 MSI data, the number of pixels in Cluster 1 and homogeneity ratio for Cluster 2 are statistically significant between the 2 treatment conditions.

### Association between clustered image and drug LISA map

The spatially distinct regions based on observed drug distribution profiles were identified using LISA maps in all tumor models. The LISA map was created using the spatial weight matrix with an autocorrelation value equal to 5 because in the spatial correlogram derived from different tumor MSI data, high positive spatial autocorrelation was observed within this range (see Additional File 5 Figure S1).

**Figure 3:**
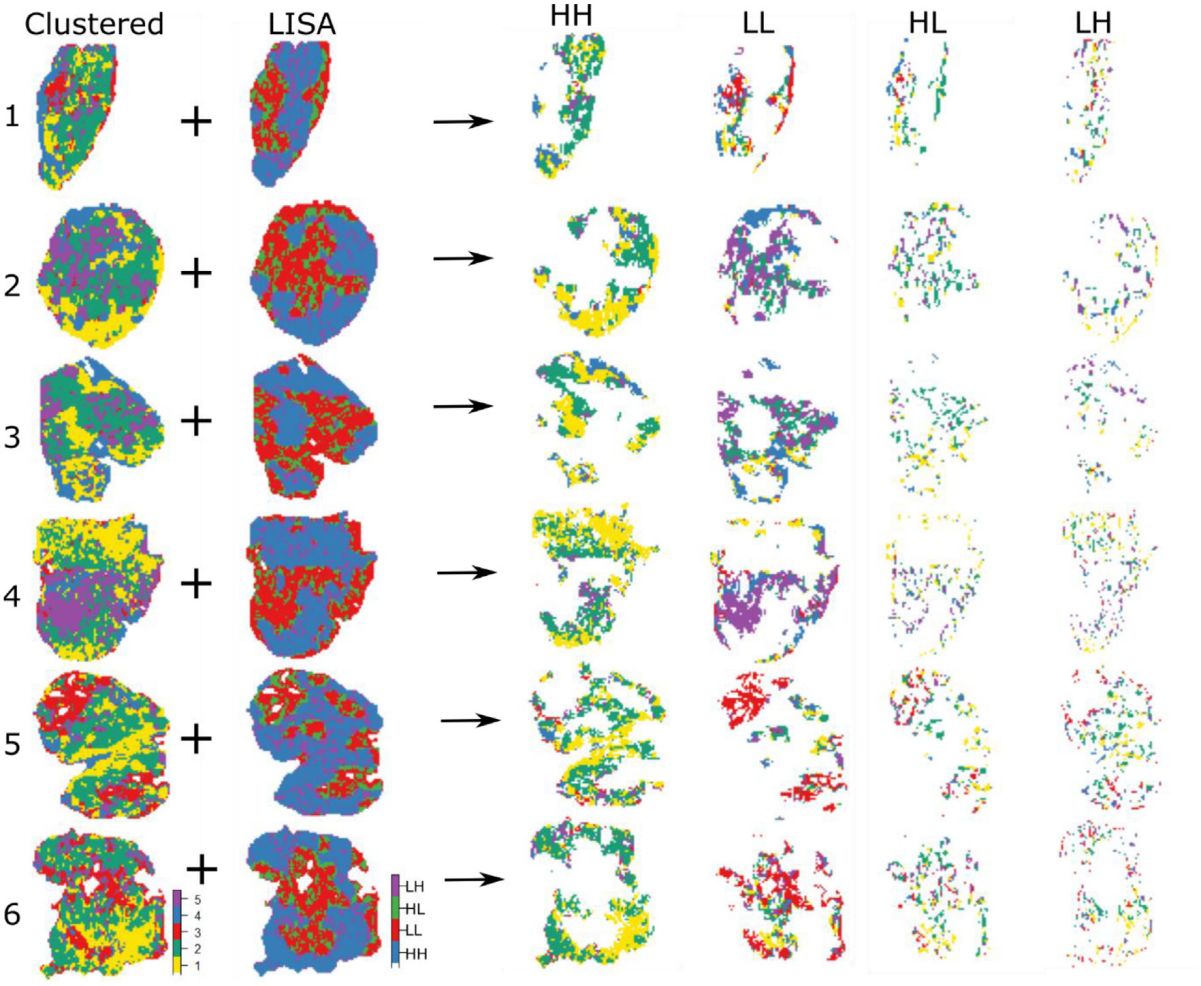
Individual clustered image (first column), LISA map (second column), and their combination are shown for a few tumor models from A2780–1A9 MSI data. The clusters found in high-high (HH), low-low (LL), high-low (HL), and low-high (LH) zones of LISA map are highlighted. In the LISA map, HH, LL, HL, and LH are zones identified in the Moran I scatter plot.

**Figure 4:**
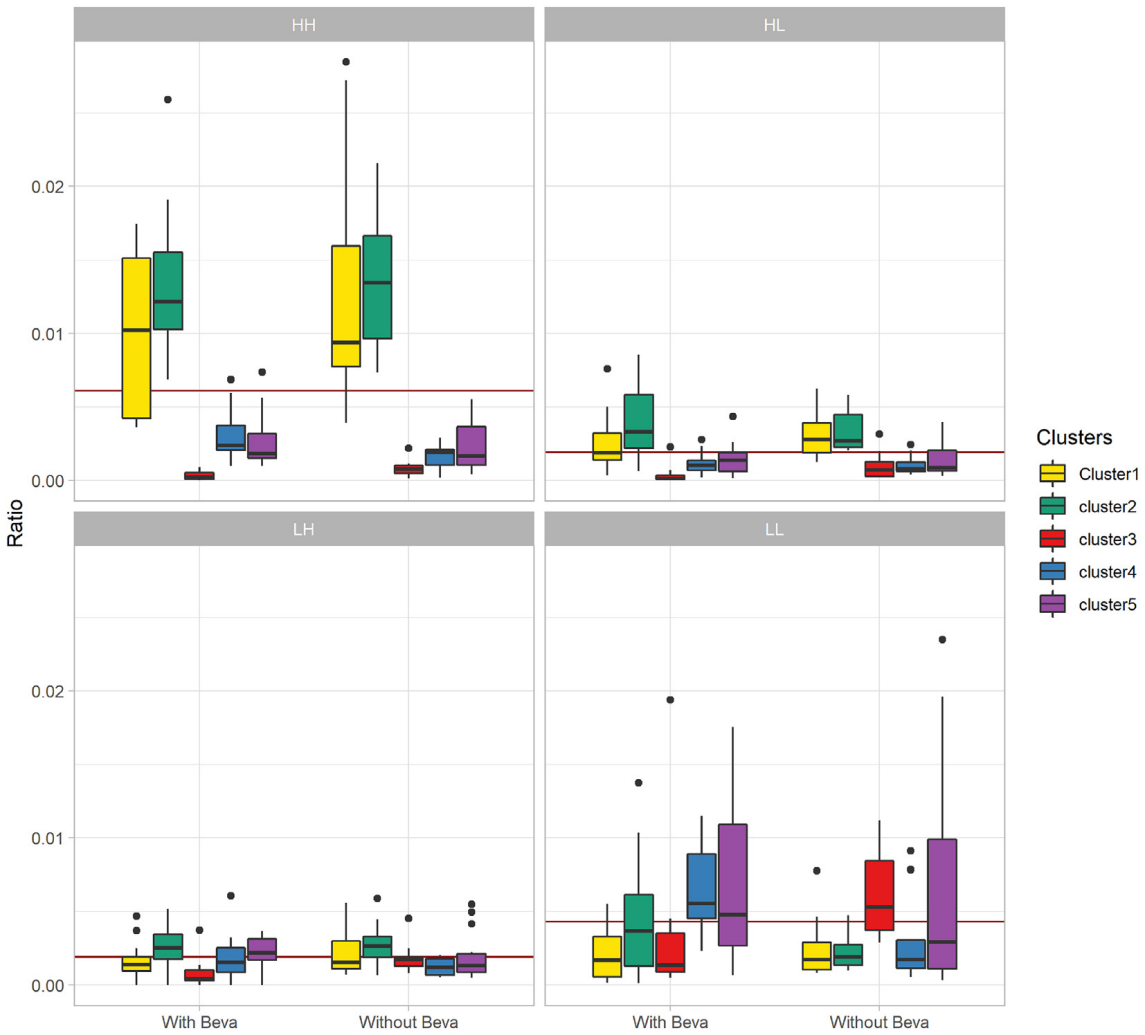
Quantitative analysis to find the association between drug LISA maps and identified clusters from complete A2780–1A9 tumor MSI data. Here, each subplot highlights the fraction of pixels present in different zones of the LISA map under 2 treatment conditions. The red horizontal line in each subplot is a global mean value for pixel ratio for that particular zone. HH: high-high; HL: high-low; LH: low-high; LL: low-low; With Beva: pretreated with bevacizumab; Without Beva: without bevacizumab pretreatment. Clusters: different clusters identified in A2780 tumor MSI data.

A visual comparison of the clustered images and drug LISA maps confirms the link between the drug distribution profile and the underlying clusters (Fig. 3). For example, homogeneously high drug distribution areas (HH zone in the LISA map) are mostly associated with Clusters 1 and 2, while homogeneously low drug distribution areas (LL zone in the LISA map) correspond to Clusters 3 and 5 (Table 1). The observed association between cluster types and the different zones of the LISA map is irrespective of treatment condition across all tumor models (Fig. 4).

**Table 1:** The percentages of pixels belonging to different cluster classes falling into HH, LL, HL, and LH zones of the LISA map for tumor MSI data shown in Fig. 3

Image No.	Zone	Cluster 1 (%)	Cluster 2 (%)	Cluster 3 (%)	Cluster 4 (%)	Cluster 5 (%)	Cramer V**
1	HH	**8.28**	**23.5**0	0.58	5.75	3.5	0.501
	LL	4.48	4.92	**7.9**0	5.26	3.46	
	HL	3.36	7.85	0.87	2.68	1.22	
	LH	2.87	5.46	2.44	2.58	2.88	
2	HH	**18.15**	**13.34**	0.31	2.98	1.94	0.643
	LL	0.86	9.39	0.80	7.49	**15.93**	
	HL	2.83	8.50	0.09	1.60	3.76	
	LH	2.89	3.57	0.43	2.10	3.01	
3	HH	**14.69**	**13.55**	0.05	5.77	2.19	0.463
	LL	4.20	11.57	0.66	9.22	**14.78**	
	HL	4.02	7.19	0.036	1.56	1.69	
	LH	1.38	2.65	0.29	1.79	2.69	
4	HH	**21.55**	**17.09**	0.09	1.95	3.89	0.69
	LL	3.67	1.91	2.29	6.20	**18.62**	
	HL	3.12	2.20	0.21	1.93	3.16	
	LH	3.30	3.44	0.67	1.40	3.29	
5	HH	**16.41**	**20.53**	2.49	4.20	2.95	0.601
	LL	2.20	3.10	**11.89**	2.33	2.15	
	HL	3.51	4.43	2.89	1.43	1.79	
	LH	3.36	5.99	3.13	2.74	2.44	
6	HH	**15.04**	**23.57**	1.53	0.99	3.23	0.62
	LL	4.04	4.12	**13.44**	2.05	**4.37**	
	HL	3.71	6.59	2.12	1.19	2.23	
	LH	2.85	3.64	2.83	0.98	1.44	

^In above table, clusters with large percentage in HH and LL regions are highlighted in bold.^

* Cramer correlation is calculated between HH, LL zones of LISA map and unsupervised clusters. HH: high-high; HL: high-low; LH: low-high; LL: low-low.

Similar observations were made from HCT116 tumor MSI data (see Additional File 5). In HCT116 MSI data, Clusters 1–3 show clear overlapping with spatially homogeneous zones of the LISA maps. Clusters 1 and 3 overlapped with high drug concentration areas in the tissue and Cluster 2 with low drug concentration areas (see Additional File 5). The association between Cluster 2 and low drug concentration areas in the LISA maps is clearer for HCT116 data than for A2780–1A9 data (Additional File 5 Table S1).

The statistical correlation between the clustered image and drug LISA map was calculated using the Cramer V method. A very small fraction of pixels falls within the HL and LH zones of the LISA map. Therefore, the Cramer V is calculated between HH, LL zones of the LISA map with unsupervised clustered classes. For the MSI data of both tumors, the Cramer value across all tumor models was found to be within the range of 0.5–0.8 (see Additional File 5 Table S1), which confirmed the dependency of the drug on different tumor tissue areas.

### Ion signal selection from the identified clusters

For variable selection, the performance of the spatial (SE and SL) and non-spatial (OLS) methods was tested on synthetic spatially autocorrelated data. The SL method completely outperformed the other 2 methods (see Additional File 3) and was therefore used for selecting *m*/*z* values from unsupervised clusters identified in the 2 MSI cancer datasets (Table 2). Note, Cluster 4 identified in A2780–1A9 tumor data was present in a single tumor model and Clusters 4 and 5 for HCT116 were present in a very small fraction and did not follow any proper spatial structure; therefore those clusters were excluded from the ion selection step. The list of important ions is given in Additional File 9. In the A2780 cancer data, Cluster 2 had 28 ions showing a significant difference. In particular, the ion at *m*/*z* = 335.41 had high intensity in all tumor models. The ion image of this particular ion showed a homogeneous distribution in the cluster (see Additional File 6 Fig. S1, top row). A larger number of significantly different ions were identified for Cluster 3, mostly with low signal intensity.

**Table 2:** The number of ion signals selected from different clusters in MSI data from 2 tumor cell lines

Cluster type	A2780	HCT116
1	26	22
2	28	83
3	91	70
5	35	

For the HCT116 MSI data, a large number of significant ions were identified in Clusters 2 and 3. In Cluster 3, the majority of ions had a high signal or positive regression coefficients in the spatial model, with the ion at *m*/*z* = 281.315 showing the highest value. In Cluster 2, ions with both high and low signals were present in equal portions (see Additional File 6 Fig. S1, bottom row).

## Discussion

Several studies have shown that the tissue spatial heterogeneity within a solid tumor affects the drug distribution [2, 10, 12, 28, 44, 11]. The fact that different tissue characteristics and tumor microenvironment affect the drug distribution means that the concentration of a drug at a given spatial location could be related to the tissue composition at that point. It is also true that the presence of the drug can induce a modification of the tumor structure [29], so in general, it is impossible to disentangle the 2 phenomena. In our case, the situation was fortunate because some of the tumor-bearing mice had received bevacizumab treatment before drug (paclitaxel) treatment. Therefore, we assume that the observed differences in the spatial organization of tissue areas characterized by similar metabolic fingerprints can be interpreted as a direct or indirect effect of bevacizumab treatment, which was given twice before drug injection. This was also suggested by our previous study, which showed an increase in drug homogeneity in samples treated with bevacizumab [28]. The main goal of this research was to show that computational methods can be used to explore and quantify spatial heterogeneity within tumors and link the observed homogeneous or heterogeneous drug distribution to the alteration in microenvironment due to applied therapeutic strategy. To achieve these objectives, our data analysis involved a combination of methods from different research streams. First, the clustering of combined MSI data was performed using *k*-means with correlation distance and then those clusters were linked with drug distribution patterns obtained using the LISA method. As stated in the Introduction, *k*-means clustering has efficiently been used in several studies performed on MSI data for the selection of relevant clusters [17, 24, 25]. Moreover, in another study, the authors also tested this method and compared it with several spatial methods, i.e., using simulated and real data, and came to the same conclusion (unpublished results from M. Prasad). In the spatial data analysis field, the LISA map is a commonly known technique that identifies the spatially relevant clusters in a single 2D image [20, 21, 41]. The LISA map provides an automated way to find spatially homogeneous clusters that are difficult to generate using a simple thresholding approach (see Additional File 8). Simple binary images were created using different threshold values. The images constructed with a threshold value of 4 show some resemblance with the LISA map (see Additional File 8). The binary images are not fully able to mimic the drug distribution profile. Moreover, the selection of the right threshold value from the drug ion image histogram is not very straightforward. In contrast, the LISA map is able to highlight the observed high- and low-intensity spatially homogeneous areas in drug ion images efficiently, where a spatial weight matrix can be selected on the basis of a spatial correlogram.

**Figure 5:**
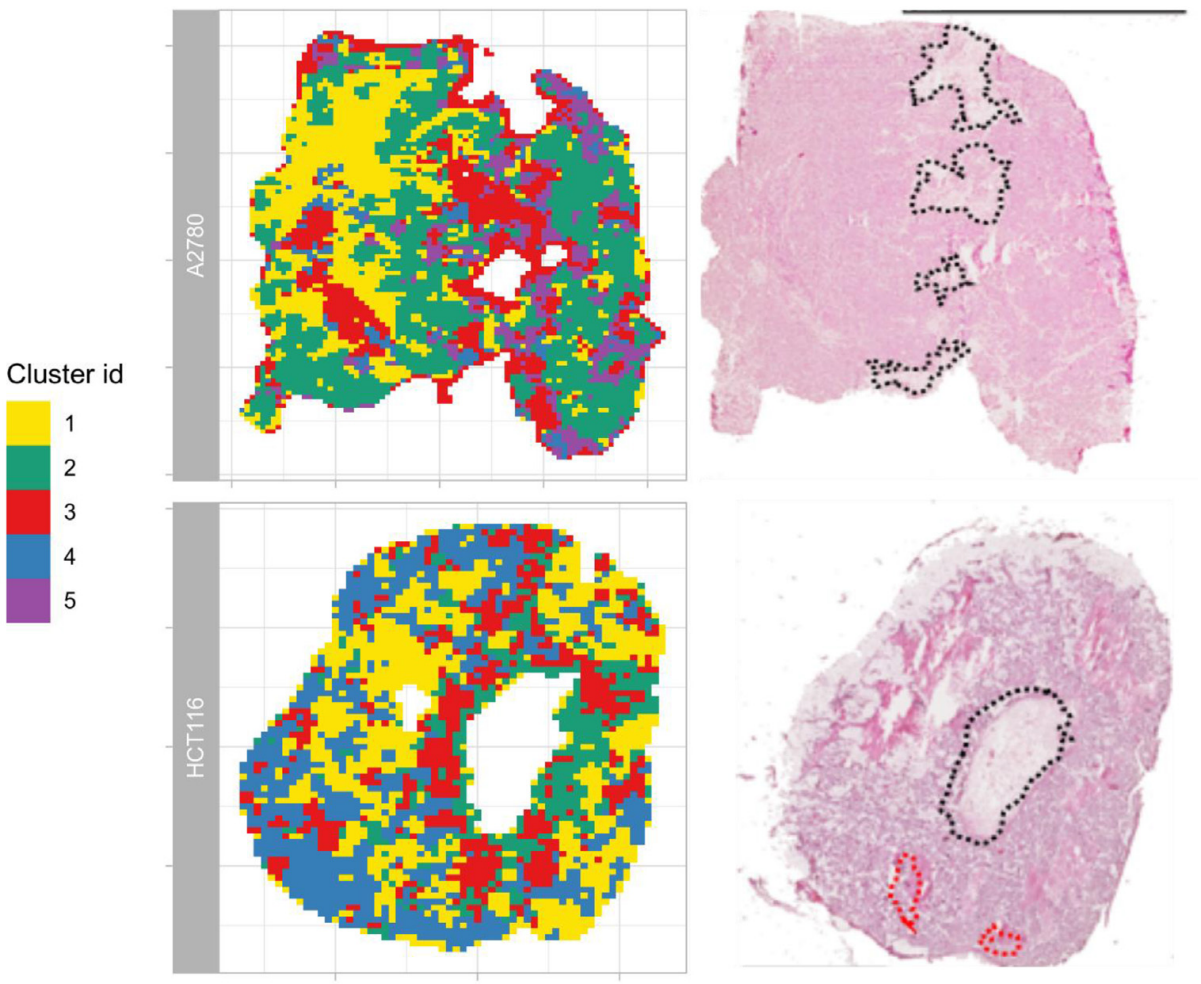
Comparison of tumor tissue optical image (right column) with its clustered image (left column) from MSI data of 2 tumor cell lines (A2780–1A9, HCT116). The black and red dots in the H&E-stained images represent the necrotic and fibrotic area, respectively. The optical images are adapted from Giordano et al. [10] published under CC BY license.

The clustering of MSI data was able to identify metabolically separated regions that cannot be observed in hematoxylin-eosin (H&E)-stained tissue images [17]. This is clear if one compares the images shown in Fig. 5. The segmented image shows tissue subtypes in addition to the one that can be associated with the necrotic and fibrous regions on the H&E-stained image. The effect of the treatment with bevacizumab was visible in the segmented images.  In particular, the antiangiogenic compound was increasing homogeneity (both in the pattern of clusters and in the drug) even if no anti-angiogenesis treatment–specific cluster was identified (Fig. 2 and Additional File 4). Interestingly, the average drug concentration in the individual clusters under the 2 treatment conditions was approximately equal (see Additional File 7). This confirmed the results of our previous publication [16] and our homogeneity assessment of clustered images (Fig. 2, bottom right) that the tumor tissues from bevacizumab treatment are more homogeneous.

Quantitative analysis of the LISA maps shows the dependency of the observed drug distribution profiles and the underlying tissue types (Fig. 4 and Additional File 5). In A2780–1A9 tumors, Clusters 1 and 2 have a high affinity for the drug while Clusters 3 and 5 show a lower than average drug concentration (Fig. 4 and Table 1).

For HCT116 tumors, Clusters 1 and 3 showed some overlap with the HH zone, and Cluster 2 with the LL zone (see Additional File 5). Remarkably, the clusters associated with the HH and LL drug distribution regions were always the same, regardless of the pretreatment with bevacizumab, which was instead affecting the arrangement of the tissue subpopulations.

Two commonly known spatial methods (LISA map and spatial lagged regression) were used in our study to find the spatially homogeneous clusters and spatially relevant ions from different clusters, respectively. Both spatial methods required the spatial weight matrix as an input. Therefore, the dependency of results on the spatial weight matrix was tested (see Additional Files 10 and 11). The variable selection was performed using the SL method for a range of autocorrelation or lag distance values (1–15) in the spatial weight matrix in tumor MSI data (see Additional File 10). The SL method selects mostly the same molecular ions at different lag distance values. All variables selected have positive Moran I value at the aforementioned autocorrelation range. Therefore, based on our analysis of MSI data we had not noticed the dependency of the SL method on the spatial weight matrix. A similar observation was made when this analysis was performed on synthetic data (see Additional File 3). In the case of our synthetic data, the accuracy of SL starts decreasing after a lag distance of 6 because some false-positive selection was observed, but sensitivity remains constant as the true variables were all selected. However, in the case of real MSI data, this type of plot is not feasible. Therefore we looked at the Moran spatial autocorrelation value of all selected variables (see Additional File 10). After the lag distance of 5 the Moran I value of the selected variables starts to approach zero. Therefore, even though in our analysis the list of molecular ions was mostly consistent we do not recommend going beyond the lag distance of 5 because at such large autocorrelation range we may start including some noisy variables in our list.

A similar approach is used to test the dependency of a LISA map on the spatial weight matrix. The LISA map of the drug ion image for a particular tumor tissue was constructed with different spatial weight matrices (see Additional File 10). The LISA map created with the lag distance of 1 contains large homogeneous areas (LL and HH). Upon increasing the lag distance, the pixels from different areas start mixing, i.e., the sizes of HL and LH regions gradually start increasing and the LISA map becomes less reliable. Therefore similar to the spatial regression method, the spatial weight matrix with a maximum lag distance of 5 is preferable for a LISA map.

In summary, in this article, we provide a computational approach to understand the problem of drug homogeneity in association with tumor heterogeneity that validates the few conclusions made in previous studies. We provide a complete workflow of data pre-processing of MSI data, the association between the drug and identified clusters, and the selection of molecular ions from identified clusters. In our article, we have used different methods to get different pieces of information. The dependency of spatial methods on the spatial weight matrix is discussed above. Apart from that, if the user wants to implement our approach on their MSI data, they also need to consider the parameters used in our studies, such as peak removal with <20% coverage area and the clustering index. In our case, before setting a 20% threshold we tried a 10–30% threshold to be sure that the majority of noisy peaks have been removed and no other important peaks. A similar task was performed during the clustering of MSI data. The number of clusters was selected using the internal clustering index method, but the stopping criterion in bisecting clustering (40%) was set according to our data dimensions and the number of expected clusters in our MSI data. If the MSI data contain very small spatial structures, then this threshold needs to be reduced. In general, we believe that our article completes the pipeline for the analysis of untargeted drug MSI data and will be useful for the groups working with a similar problem. An R pipeline including all the methods from our publications is available (CorrDrugTumorMSI, RRID:SCR_018962) (bio.tools: corrdrugtumormsi).

## Conclusions

In cancer research, one of the causes of drug therapy failure is tumor drug resistance often induced by incomplete drug penetration. This phenomenon is supposed to be linked to the presence of diverse tumor microenvironments that are difficult to identify with established histological techniques. In this work, we show that a molecular imaging technique like MSI, coupled with advanced data analysis strategies, offers a great opportunity to investigate the link between drug distribution and tissue heterogeneity. Our approach allowed us to simultaneously investigate tissue spatial heterogeneity and drug distribution, and it was capable of detecting the effects on the tumor heterogeneity induced by a specific intervention (treatment with bevacizumab). We hope that the unsupervised approach proposed here will help oncologists to quantitatively evaluate the efficacy of therapeutic strategies.

## Availability of Supporting Code and Requirements

CorrDrugTumorMSI is developed in R. All source codes from this work are freely accessible at https://github.com/mridulaprasad/CorrDrugTumorMSI (Licence: GPL-3). CorrDrugTumorMSI is also registered in the bio.tools (corrdrugtumormsi) and SciCrunch (RRID:SCR_018962) databases.

## Data Availability

The datasets and R script used to generate the results of this article are available on the DNAS-KNAW repository [45] Other data further supporting this work are openly available in the GigaScience repository, GigaDB [46].

## Additional Files

**Additional File 1:** Supplementary Figure S1. Schematic workflow of adaptive bins creation. Supplementary Figure S2. Maximum intensity reference spectrum from 2 tumor MSI data.

**Additional File 2:** Supplementary Text S1. Description of the statistical methods (Moran's index, Moran's scatter plot and homogeneity index) used in this article.

**Additional File 3:** Supplmentary Text S1. Description of the synthetic spatially autocorrelated data generation steps. Supplementary Figure S1. Characterstics of synthetic spatial data. Supplementary Text S2. Description of non-spatial and spatial methods used for variables selection. Supplmentary Figure S2. Comparison of spatial and non-spatial methods for variables selection on synthetic data.

**Additional File 4:** Supplementary Figure S1. Cluster analysis of HCT116 MSI. Supplementary Table S1. The range and significance values of pixels and homogeneity ratio from clusters belong to two tumor MSI data.

**Additional File 5:** Supplementary Figure S1. Spatial correlogram of the drug ion from A2780 and HCT116 tumor MSI data. Supplementary Figure S2. Overlay of clustered image and LISA map. Supplmentary Table S1 contains is the contigency table for Supplementary Figure S2. Supplementary Figure S3. Quantitative analysis of LISA maps for HCT116 tumor MSI data.

**Additional File 6:** Supplementary Figure S1. MS images of selected molecular ions from different clusters from tumor MSI data.

**Additional File 7:** Supplementary Figure S1.Plot of normalized average drug concentration in each cluster unders two treatment conditions from tumor MSI data.

**Additional File 8:** Supplementary Figure S1. Comparison of drug binary image created by selecting manual threshold value based on histogram with LISA map.

**Additional File 9:** Supplementary Sheet S1. Excel sheet 1 and 2 contain list of important ion signals selected from identified clusters of A2780 and HCT116 tumor MSI data.

**Additional File 10:** Supplementary Figure S1. The number of variables selected at different lag distances using spatial lagged method. Supplementary Figure S2. Drug ion spatial correlogram and LISA map created at different lag distance.

**Additional File 11:** Supplementary Sheet S1. List of molecular ions selected for clustered image shown in Additional File 10 Supplementary Figure S1.

## Abbreviations

Ac1: Moran I autocorrelation; CC: Creative Commons; dists: lag distance; DHI: Drug Homogeneity Index; H&E: hematoxylin-eosin; HH: high-high; HL: high-low; LH: low-high; LL: low-low; LISA: local indicators of spatial association; *m*/*z*: mass-to-charge ratio; MALDI: matrix-assisted laser desorption/ionization; MSI: mass spectrometry imaging; NIH: National Institutes of Health; Nu: value of smallest size-zone in DHI formula; OLS: ordinary least squares; SE: spatial error model; SL: spatial lag model; TOF: time of flight.

## Competing Interests

The authors declare that they have no competing interests.

## Funding

M.P.'s position was funded by the Fondazione Edmund Mach, Italy (FIRST_2012_AM_07_CdC 3707–P1111021I), Institute for Molecules and Materials (IMM), Radboud University, Netherlands, and University of Turin (2016-UNTOBIO-0001145). All the experimental work was funded by the Cariplo Foundation (2013–0692).

## Authors' Contributions

M.P. analyzed the MSI data and wrote the first draft of the manuscript under the supervision of G.P., P.F., J.J., and L.M.C.B. G.P., P.F., and J.J. edited the manuscript. E.D., L.M., S.G., F.F., and R.G. did the MSI experiment and reviewed the manuscript. All authors read and approved the final manuscript.

## Supplementary Material

giaa131_GIGA-D-20-00114_Original_Submission

giaa131_GIGA-D-20-00114_Revision_1

giaa131_GIGA-D-20-00114_Revision_2

giaa131_Response_to_Reviewer_Comments_Original_Submission

giaa131_Response_to_Reviewer_Comments_Revision_1

giaa131_Reviewer_1_Report_Original_Submission Nathan Heath Patterson -- 5/29/2020 Reviewed

giaa131_Reviewer_1_Report_Revision_1 Nathan Heath Patterson -- 9/24/2020 Reviewed

giaa131_Reviewer_2_Report_Original_Submission Chanchala Kaddi -- 5/29/2020 Reviewed

giaa131_Supplemental_Files
