# Supplementary material for: A methodological approach to correlate tumor heterogeneity with drug distribution profile in mass spectrometry imaging data
Source: Gigascience. 2020 Nov 25;9(11):giaa131. doi: 10.1093/gigascience/giaa131 (PMC7688471; doi:10.1093/gigascience/giaa131)
Supplement: giaa131_GIGA-D-20-00114_Original_Submission [file giaa131_giga-d-20-00114_original_submission.pdf]

GigaScience

A methodological approach to correlate tumor heterogeneity with drug distribution profile in mass spectrometry imaging (MSI) data

--Manuscript Draft--

**Manuscript Number:**	GIGA-D-20-00114
**Full Title:**	A methodological approach to correlate tumor heterogeneity with drug distribution profile in mass spectrometry imaging (MSI) data
**Article Type:**	Research
**Funding Information:**	
**Abstract:**	Mass spectrometry imaging (MSI) has become a valuable tool in drug imaging because of its ability to provide a simultaneous spatial distribution of the drug and several other molecular ions present in the biological sample. An important application is the evaluation of homogeneity/heterogeneity of drug distribution in solid tumors. Solid tumors are known to be made up of different tissue subpopulations and their heterogeneity is supposed to have a direct and/or indirect influence on drug distribution. Hence, for further enhancement of penetration therapy performance, it is important to link a characterization of the tumor microenvironment with drug homogeneity. In this study, untargeted MSI data were used to understand the spatial heterogeneity within solid tumors, assessing its impact on the drug (paclitaxel) distribution. The proposed approach was applied on MSI datasets already analyzed, focusing on tumor drug distribution. Untargeted MSI datasets were collected on different tumor xenograft models (ovarian and colon cancer cell lines) pre-treated or not with anti-angiogenesis compound (bevacizumab). Our main data analysis steps involved: a) pre-processing of MSI data to make all biological samples directly comparable, b) unsupervised data clustering to find different tissue subtypes, c) quantification of drug heterogeneity using local indicators of spatial association (LISA) map and d) selection of important ion signals from identified clusters of interest using the spatial -aware statistical tools. Our clustering results show variation in tumor subpopulations and less spatial heterogeneity in the MSI data collected on samples treated with the anti-angiogenesis compound consistently with our previous data. The local spatial structures identified in drug ion LISA maps show a correlation with clusters identified using a clustering method. Using the right spatial method, we were able to reduce the number of false-positive ions selected and identified the one that shows relevant spatial patterns in different tissue subtypes. Finally, our overall study shows that there is a direct association in drug homogeneity and spatial arrangement of different tissue subtypes in a solid tumor.
**Corresponding Author:**	Geert Postma<br>Radboud Universiteit<br>Nijmegen, NETHERLANDS
**Corresponding Author Secondary Information:**	
**Corresponding Author's Institution:**	Radboud Universiteit
**Corresponding Author's Secondary Institution:**	
**First Author:**	Mridula Prasad, M.Sc.
**First Author Secondary Information:**	
**Order of Authors:**	Mridula Prasad, M.Sc.
	Geert Postma
	Pietro Franceschi
	Lavinia Morosi
	Silvia Giordano
	Francesca Falcetta
	Raffaella Giavazzi
	Enrico Davoli

Powered by Editorial Manager® and ProduXion Manager® from Aries Systems Corporation

	Lutgarde M.C. Buydens
	Jeroen Jansen
**Order of Authors Secondary Information:**	
**Additional Information:**	
**Question**	**Response**
Are you submitting this manuscript to a special series or article collection?	No
**Experimental design and statistics**<br><br>Full details of the experimental design and statistical methods used should be given in the Methods section, as detailed in our Minimum Standards Reporting Checklist. Information essential to interpreting the data presented should be made available in the figure legends.<br><br>Have you included all the information requested in your manuscript?	Yes
**Resources**<br><br>A description of all resources used, including antibodies, cell lines, animals and software tools, with enough information to allow them to be uniquely identified, should be included in the Methods section. Authors are strongly encouraged to cite Research Resource Identifiers (RRIDs) for antibodies, model organisms and tools, where possible.<br><br>Have you included the information requested as detailed in our Minimum Standards Reporting Checklist?	Yes
**Availability of data and materials**<br><br>All datasets and code on which the conclusions of the paper rely must be either included in your submission or deposited in publicly available repositories (where available and ethically appropriate), referencing such data using a unique identifier in the references and in the "Availability of Data and Materials" section of your manuscript.<br><br>Have you have met the above requirement as detailed in our Minimum Standards Reporting Checklist?	Yes

Powered by Editorial Manager® and ProduXion Manager® from Aries Systems Corporation

Manuscript

Click here to download Manuscript Manuscript.docx

A methodological approach to correlate tumor heterogeneity with drug distribution profile in mass spectrometry imaging (MSI) data

Mridula Prasad[1,2], Geert Postma[1*], Pietro Franceschi[2], Lavinia Morosi[3], Silvia Giordano[4], Francesca Falcetta[3], Raffaella Giavazzi[3], Enrico Davoli[4], Lutgarde M.C. Buydens[1], Jeroen Jansen[1]

[1]IMM/ Analytical Chemistry, Radboud University, Heyendaalseweg, 6525 AJ Nijmegen, Netherlands.

[2]Unit of Computational Biology, Research and Innovation Center, Fondazione Edmund Mach, 38010, San Michele all' Adige, Italy.

[3]Department of Oncology, Istituto di Ricerche Farmacologiche Mario Negri IRCCS, Via La Masa, 19-20156 Milan Italy.

[4]Mass Spectrometry Laboratory, Istituto di Ricerche Farmacologiche Mario Negri IRCCS, Via La Masa, 19-20156 Milan Italy,

ABSTRACT: Mass spectrometry imaging (MSI) has become a valuable tool in drug imaging because of its ability to provide a simultaneous spatial distribution of the drug and several other molecular ions present in the biological sample. An important application is the evaluation of homogeneity/heterogeneity of drug distribution in solid tumors. Solid tumors are known to be made up of different tissue subpopulations and their heterogeneity is supposed to have a direct and/or indirect influence on drug distribution. Hence, for further enhancement of penetration therapy performance, it is important to link a characterization of the tumor microenvironment with drug homogeneity. In this study, untargeted MSI data were used to understand the spatial heterogeneity within solid tumors, assessing its impact on the drug (paclitaxel) distribution. The proposed approach was applied on MSI datasets already analyzed, focusing on tumor drug distribution. Untargeted MSI datasets were collected on different tumor xenograft models (ovarian and colon cancer cell lines) pre-treated or not with anti-angiogenesis compound

(bevacizumab). Our main data analysis steps involved: a) pre-processing of MSI data to make all biological samples directly comparable, b) unsupervised data clustering to find different tissue subtypes, c) quantification of drug heterogeneity using local indicators of spatial association (LISA) map and d) selection of important ion signals from identified clusters of interest using the spatial -aware statistical tools. Our clustering results show variation in tumor subpopulations and less spatial heterogeneity in the MSI data collected on samples treated with the anti-angiogenesis compound consistently with our previous data. The local spatial structures identified in drug ion LISA maps show a correlation with clusters identified using a clustering method. Using the right spatial method, we were able to reduce the number of false-positive ions selected and identified the one that shows relevant spatial patterns in different tissue subtypes. Finally, our overall study shows that there is a direct association in drug homogeneity and spatial arrangement of different tissue subtypes in a solid tumor.

Introduction

Heterogeneity of the tumor microenvironment directly affects drug penetration reducing the therapeutic efficacy and contributing to the development of resistance[1,2]. For the above-mentioned reasons, knowledge about tumor spatial heterogeneity is then of paramount importance to optimize therapeutic outcomes[3]. In order to understand the relation between drug distribution and tissue homogeneity/heterogeneity, an imaging technique able to investigate both phenomena simultaneously would be highly desirable[4].

Mass spectrometry imaging (MSI) is a molecular imaging technique that provides simultaneous information about spatial localization of drugs and other small molecules present in the biological sample[5,6]. It is one of the techniques of election to characterize tumor tissue subtypes[7–9]and it is also applied to map the distribution of drugs within the tissue[10–13]. Untargeted MSI datasets provide unprecedented opportunities to understand the drug distribution in association with tissue heterogeneity. Several computational algorithms have been implemented on MSI data for the identification of tumor tissue areas[7,8,14]and important molecular ions selection[8,15,16]. As far as our knowledge there is no work done that combines the tumor heterogeneity with a drug distribution profile. In a recent paper, we

have proposed a new objective index (DHI)[17] to measure drug homogeneity in different MSI datasets and we aim at extending this work proposing a framework that can be used to investigate the relation between drug homogeneity and the observed tumor heterogeneity. The proposed approach encompasses the following steps.

1. Identification of the different tissue sub-populations from an untargeted multi-sample MSI dataset (segmentation).
2. Quantitative analysis of the spatial arrangement of the tissue subtypes across different samples.
3. Extraction of discrete drug distribution maps.
4. Matching of the segmented MSI dataset with the drug distribution maps.
5. Selection of ion signals that can be used to differentiate specific tissue areas.

Since the accessibility of a drug to a particular spatial location is expected to be linked to the metabolic and histological characteristics of the underlying tissues, multivariate unsupervised clustering was used to group the pixels into a limited number of groups which represent the different tissue subtypes[8,9,14,18–20] (Step 1). This step was performed after removing the drug-related peaks to avoid their influence in groups formation. The quantitative assessment of the spatial arrangement of the different clusters (Step 2) was performed by using a modified version of our DHI[17] index, which was optimized to characterize the spatial arrangement of the pixels on the clustered images. The *local indicators of spatial association (LISA)*[21,22] method was instead applied to create discrete drug distribution maps (Step 3). The association between LISA maps and identified segments was derived using Cramer's[23] V method (Step 4). The identification of important ion signals able to differentiate the tissue subtypes was finally performed by using a spatial-aware statistical method that corrects for spatial autocorrelation[24] (Step 5). Since the application of these models in MSI is still uncommon, the optimal spatial model for biomarker selection was tested on a synthetic autocorrelated spatial dataset.

In our study, the identification of tissue subpopulations was performed using K-means clustering with correlation distance. This non-spatial clustering method has already been used in many other MSI research[25–27] and was able to efficiently extract relevant structures. The spatially relevant clusters in drug ion image were detected based on Moran's scatter plot or LISA map. LISA map is a commonly used method in spatial statistics where pixels are grouped into different clusters (zones) based on their similar

or different behavior with neighboring pixels[28,29]. The potential of the proposed approach was tested on the same datasets that we used in our previous work[17,30] which consisted of the MSI untargeted analysis of a set of different tumor xenograft models from ovarian (A2780) and colon (HCT116) cancer cell lines. The study was designed to assess the effect of an anti-angiogenesis compound (bevacizumab) on the drug (paclitaxel) distribution[30]. For this purpose, half of the animals were pretreated twice (5 and 1 days before excision) with bevacizumab, before being administered with paclitaxel a few hours (six) before excision. Even if it is impossible to exclude that the anticancer drug could be responsible for the observed spatial heterogeneity in metabolic profile[31], any relevant difference between the two treatment groups is likely to be associated with the direct or indirect effect of bevacizumab.

EXPERIMENTAL SECTION

MSI datasets

MSI data were derived from the tumor-bearing mice, treated with paclitaxel (60 mg/kg) alone or in combination with bevacizumab (two intraperitoneal injections at 150 µg per mouse). For mice experiment, IRFMN adheres to the principles set out in the following laws, regulations, and policies governing the care and use of laboratory animals: Italian Governing Law (D.lgs 26/2014; Authorization n.19/2008-A issued March 6, 2008 by Ministry of Health); Mario Negri Institutional Regulations and Policies providing internal authorization for persons conducting animal experiments (Quality Management System Certificate–UNI EN ISO 9001:2008 –Reg. N° 6121); the NIH Guide for the Care and Use of Laboratory Animals (2011 edition) and EU directives and guidelines (EEC Council Directive 2010/63/UE).

The complete details of the experiment experiments described here.[12,17,30]· Briefly, tumors were collected, frozen in liquid nitrogen and prepared for MSI analysis. A MALDI 4800 TOF-TOF (AB SCIEX, Old Connecticut Path, Framingham, MA) was used. And, mass spectra were recorded in full-scan-profile mode over a limited mass range (*m*/*z* 199–500). Images of tissue sections were acquired using the 4800 Imaging Tool software with an imaging raster of 100 × 100 µm (pixel dimension of ca. 0.01

mm^2). The obtained dataset consisted of 131 349 (A2780) and 59 652 (HCT116) raw spectra from the tissue areas of all tumor models.

MSI data pre-processing

The initial data files in Analyze 7.5 format were opened in R[32] free software version 3.4.3 using the MALDIquant[33] package. Each MSI datafile contains mass spectra collected from both tumor tissue and a glass slide. To avoid any bias in pre-processing and data analysis steps, the mass spectra belonging to tumor tissue only were used. The identification of tumor tissue was performed by constructing a mask of the ion signal detected in the m/z = 281.1-281.44 range. This ion was confirmed to be a reliable tissue marker by visually comparing all MS images with their optical counterparts. To correct for possible spectral misalignments across the different datasets, an adaptive binning approach was applied. To do this, first, a reference spectrum was created, which is a single maximum intensity spectrum of all the spectra. The main reasoning behind it is to acquire knowledge about the location and shape of all the ion peaks in our data and then optimize the bin size accordingly. Smoothing of the reference spectrum was performed by applying discrete wavelet transformation (Daubechies least asymmetric 8-tap filter with hard shrinkage) using a msDenoiseWavelet function from msProcess[34] R package. Peaks in the reference spectrum were identified by local maxima search above a certain threshold[17]. Initial bins were created based on those identified peaks. The bin size was further optimized on the bases of instrument characteristics merging together peaks showing a difference of less than 0.05m/z. To avoid that peaks are missed in low-intensity spectra, an extra bin of size 0.5 was added in the presence of a large gap between two identified peaks (especially towards the end of the spectra). The complete workflow of bin creation and maximum intensity spectra from two tumor MSI data are shown in Additional file 1.

After bin identification, each MSI data file was reopened and peak picking was performed using an approach similar to the one applied to the reference spectrum and the identified peaks were assigned to their corresponding bins. To focus only on the more common ions, peaks that were present in less than 20% of the tissue area were removed. Peaks only present in a single tumor model were rejected. To remove

spatial noise, median filtering with a window size of 3x3 pixels was performed on each extracted ion image. We also performed edge correction in the individual dataset, as marginal pixels have enormously high intensity due to the surface difference between tissue and glass slide[35]. image. To make the spectra collected on the different pixels comparable median normalization was performed. Generalized log transformation was performed as a variance stabilization step using LMGene[36] in R. A plausible batch effect between the slices of certain mouse models was removed using the removeBatchEffect function from R limma[37] package. Ion peaks with correlation with the drug compound (paclitaxel) (>0.5) were removed before cluster analysis. The overall dimension of the final data matrix belonging to A2780 and HCT116 is equal to 131 349 x 173 and 59 652 x 155, respectively.

MSI data analysis

Unsupervised data clustering

Segmentation of MSI data collected on the complete set of sections from a specific cell-line was performed by unsupervised bisect k-means clustering[38] using correlation as a similarity measure. The individual clusters were allowed to split further until the largest cluster contains 40% of the pixels included in the initial data matrix. The number of clusters at each step was selected based on the calinhara internal validity index[39]. The clustering and validation were implemented using R-packages amap[40] and fpc[41], respectively. The outcomes of segmentation for the different sample groups (cell lines and bevacizumab treatment) were analyzed based on the following parameters: a) size and b) homogeneity of the individual clusters. The homogeneity of the individual clusters was assessed by using a slightly modified version of the drug homogeneity index (DHI) (see Additional File 2). Higher cluster homogeneity means the more continuous/homogeneous distribution of a particular tissue-type. All these parameter values were normalized by the total number of pixels from the tumor models of each treatment condition. Statistical significance of pixel and homogeneity ratio under two treatment conditions was calculated using linear mixed models with the nlme[42] R-package.

Quantification assessment of the drug distribution

Spatial quantification of the drug heterogeneity was performed using the Moran's I scatter plot[24], also known as the local indicators of spatial autocorrelation (LISA) map (see Additional File 2). This spatial-aware method was selected because is expected to yield more robust results in the presence of the spatially autocorrelated drug signal.

To create a Moran's I scatter plot and/or LISA map required inputs are: original variable, spatially lagged variable and spatial weight matrix. The original variable in our case it is the ion intensity map of the drug peak. The spatially lagged variable is constructed by multiplying the autoscaled version of the original variable with the help of the spatial weight matrix[24,43]. This weight matrix stores the connections between nearby observations (e.g. in a binary weight matrix the observations which lie within a certain range of autocorrelation receive a value of one else zero). The optimal range of autocorrelation can be decided based on spatial correlogram. A spatial correlogram[24] is a 2D plot where the spatial autocorrelation index (Moran's I) is plotted as a function of lag distance where a positive value indicates the presence of autocorrelation within a certain distance range. To create a LISA map of a drug ion image the following steps were performed:

- the optimal spatial weight matrix was created on the bases of the spatial correlogram plot (Figure 1b).
- the original variable was converted into its spatially lagged version (Figure 1c).
- Moran's I scatter plot was created by regressing the original variable against its spatially lagged version where pixels are grouped into four different zones usually called high-high, low-low, high-low and low-high (Figure 1d).
- Finally, a LISA map was constructed which is a two-dimensional image where pixels are labeled according to their class in Moran's I scatter plot (Figure 1e).

Figure 1. A schematic workflow of drug LISA map creation. a) Original drug ion image. b) Spatial correlogram of the drug-ion image where Moran's I values (Ac1) are plotted against the lag distance (dists). c) spatially lagged image of the drug-ion. d) Moran's I scatter plot where the drug signal and its spatially lagged version are regressed against each other. e) LISA clustered map of the drug ion where pixels falling in the same quadrant of the Moran scatter plot are grouped.

In a LISA map or Moran's I scatter plot, the high-high zone contains pixels that have a high intensity or above-average value and surrounded by a similar type of high-intensity pixels. The low-low zone contains pixels that have a lower intensity or below-average value and surrounded by a similar type of low-intensity pixels. The high-low zone contains pixels that have above-average value for themselves but surrounded by neighbors with below-average value. The reverse rule applies to the pixels fall in the low-high zone. Note, in a LISA map pixels falling in high-high and low-low zones show positive spatial autocorrelation and are spatially smooth. Therefore, a single zone of a LISA map may contain multiple clusters with approximately similar profiles.

Association between clustered image and drug LISA map

The obtained drug LISA maps were analyzed for their association with the unsupervised clusters obtained using unsupervised clustering. A quantitative analysis was performed to understand which cluster subtypes overlap most with which zone of the LISA map, for which the fraction of pixels in different zones and clusters in each tumor model was calculated. Further, the strength of association between those two vector classes (LISA map zones and unsupervised clusters) was estimated using Cramer's V[24] method. Cramer's V is a statistical measure similar to the Pearson correlation to find the correlation between two nominal variables and returns a correlation value within the range of 0-1.

Representative ion signals selection from the identified clusters

The method used to select representative ion signals from the identified clusters in the MSI data was first validated on synthetic spatially autocorrelated data. Two spatial approaches (spatial error model (SE), and spatial lag model (SL)) were compared with a standard non-spatial approach (ordinary least square (OLS)). Both spatial models[24,44] are modified versions of an OLS model and include spatial autocorrelation in a different component of the OLS model. The comparison of the performance of the above mentioned statistical methods with complete description of the synthetic data generation process is given in Additional File 3.

All spatial models were fitted with the spdep[24] R package. Similar to what was done in the case of the LISA map, the right threshold for the spatial weight matrix was decided based on the spatial correlogram.

In MSI data, the selection of ion signals from identified clusters was performed using the method which gives the best performance on our synthetic data. In order to do that, the outcomes of the original clustering were converted into a set of two-class images where each cluster is, in turn, compared with all the others. As the variables selection using the spatial method is computationally intensive, we only used a few tumor models in which the cluster of interest was present. Thus, per cluster five different tumor slices were selected, i.e. MSI data from 4 - 5 different tumor models. If a particular ion was found to be important in all five datasets, then it was considered as a significant ion signal for the respective cluster. The important ions were selected on the bases of the model p-values corrected for multiple testing by using the procedure of Benjamini & Hochberg[45].

Results

Unsupervised clustering of MSI data

a) A2780 cell-line based MSI data

The clustering method identified five unique clusters in the combined set of A2780 xenograft models (Figure 2 left). The majority of the replicates possess those five clusters in different ratios where clusters 1 and 2 were predominant in all tumor models in both treatment conditions. The relative contribution of cluster 3 is reduced and cluster 4 is enhanced in the presence of bevacizumab (Figure 2, top right). Cluster 3 showed a high overlap with the necrosis area[12] and noticed to be present in a relatively higher fraction among the tumor models not pretreated with bevacizumab. The small fraction of cluster 5 is present in all tumor models.

Similar to the number of pixels, the homogeneity of clusters (parameter b) for the individual tumors was calculated using the modified version of our DHI and it is shown in Figure 2, bottom right. The figure highlights the clear difference in clusters homogeneity under the two treatment conditions, especially for

cluster 2. The homogeneity of cluster 2 in the presence of bevacizumab treatment is much higher than the homogeneity of any other cluster in two treatment conditions. Without bevacizumab treatment also tumor models show high homogeneity for cluster 2 followed by cluster 3.

Figure 2: Cluster analysis of A2780 tumor MSI data generated in the presence and absence of bevacizumab treatment. Left: Representation of clusters detected by the k-means method. Right: Ratio of a) pixels and b) homogeneity calculated from individual clusters under two treatment conditions. The red horizontal line is the global mean value of pixel and homogeneity ratio. Here, Pixel ratio = Number of pixels in individual clusters/Total number of pixels from all tumor MSI data under particular treatment conditions. Homogeneity ratio =Size-zone of individual clusters for a given tumor model/Total number of pixels in that particular tumor model. With Beva = pretreated with bevacizumab and Without Beva = without bevacizumab pretreatment.

b) HCT116 cell-line based MSI data

Five clusters were identified in the HCT116 tumor cell line MSI data (see Additional File 4). Similar to the A2780-1A9 tumor MSI data, there was not a large observed difference in clusters population under the two treatment conditions. Cluster 2 and 3 were predominantly present in all tumor models irrespective of the treatment conditions (see Additional File 4 Figure S-1 top right). Cluster 1 was observed in a moderate amount and very small fractions of clusters 4 and 5 were present in all tumor models. The homogeneity assessment of the individual clusters in the two treatment conditions shows that cluster 3 has high homogeneity in the case of bevacizumab treatment. In the absence of bevacizumab treatment, clusters 2 and 3 show more homogeneity (see Additional File 4 Figure S-1 bottom right).

The statistical analysis of pixel and homogeneity ratio values was performed using a linear mixed model approach where range and p-value from both tumor models are given in Additional File 4 Table S-1. For the A2780-1A9 MSI data, the pixel ratios are not significantly different in the two treatment conditions. The homogeneity value of cluster 2 is close to significant which is derived with parameter $Nu = 5$ in our

homogeneity formula. In HCT116 MSI data, the number of pixels in cluster 1 and homogeneity ratio for cluster 2 is statistically significant in two treatment conditions.

Association between clustered image and drug LISA map

A visual comparison of the clustered images and drug LISA maps confirms the link between the drug distribution profile and the underlying clusters (Figure 3). For example, homogeneously high drug distribution areas (high-high (HH) zone in the LISA map) are mostly associated to cluster type 1 and 2, while, homogeneously low drug distribution areas (low-low (LL) zone in the LISA map) correspond to cluster type 3 and 5 (Table 1). The observed association between cluster types and the different zones of the LISA map is irrespective of treatment condition across all tumor models (Figure 4).

Similar observations were made from HCT116 tumor MSI data (see Additional File 5). In HCT116 MSI data, clusters 1,2 and 3 show clear overlapping with spatially homogeneous zones of the LISA maps. The cluster 1 and 3 overlapped with high drug concentration areas in the tissue and cluster 2 with low drug concentration areas (see Additional File 5). The association between cluster 2 and low drug concentration areas in the LISA maps is more clear for HCT116 data than for A2780-1A9 data (Additional File 5 Table S-1).

The statistical correlation between the clustered image and drug LISA map was calculated using Cramer's V method. A very small fraction of pixels falls within high-low and low-high zones of the LISA map. Therefore, the Cramer's V is calculated between HH, LL zones of LISA map with unsupervised clustered classes. For both tumor MSI data, the Cramer's value across all tumor models was found to be within the range of 0.5 -0.8 (see Additional File 5 Table S-1) that confirmed the dependency of the drug on different tumor tissue areas.

Figure 3: Individual clustered image (first column), LISA map (second column) and their combination are shown for few tumor models from A2780-1A9 MSI data. The clusters found in high-high (HH), low-

low (LL), high-low (HL), and low-high (LH) zones of LISA map are highlighted. In LISA map, HH, LL, HL, and LH are zones identified in Moran's I scatter plot.

Table 1: The percentages of pixels belonging to different cluster classes falling into HH, LL, HL and LH zones of the LISA map for tumor MSI data shown in Figure 3.

		Cluster 1	Cluster 2	Cluster 3	Cluster 4	Cluster 5	Cramer's V*
Image 1	HH	**8.28%**	**23.5%**	0.58%	5.75%	3.5%	0.501
	LL	4.48%	4.92%	**7.9%**	5.26%	3.46%	
	HL	3.36%	7.85%	0.87%	2.68%	1.22%	
	LH	2.87%	5.46%	2.44%	2.58%	2.88%	
Image 2	HH	**18.15%**	**13.34%**	0.31%	2.98%	1.94%	0.643
	LL	0.86%	9.39%	0.8%	7.49%	**15.93%**	
	HL	2.83%	8.5%	0.09%	1.6%	3.76%	
	LH	2.89%	3.57%	0.43%	2.1%	3.01%	
Image 3	HH	**14.69%**	**13.55%**	0.05%	5.77%	2.19%	0.463
	LL	4.2%	11.57%	0.66%	9.22%	**14.78%**	
	HL	4.02%	7.19%	0.036%	1.56%	1.69%	
	LH	1.38%	2.65%	0.29%	1.79%	2.69%	
Image 4	HH	**21.55%**	**17.09%**	0.094%	1.95%	3.89%	0.69
	LL	3.67%	1.91%	2.29%	6.2%	**18.62%**	
	HL	3.12%	2.20%	0.21%	1.93%	3.16%	
	LH	3.30%	3.44%	0.67%	1.4%	3.29%	
Image 5	HH	**16.41%**	**20.53%**	2.49%	4.2%	2.95%	0.601
	LL	2.20%	3.10%	**11.89%**	2.33%	2.15%	

	HL	3.51%	4.43%	2.89%	1.43%	1.79%	
	LH	3.36%	5.99%	3.13%	2.74%	2.44%	
Image 6	HH	**15.04%**	**23.57%**	1.53%	0.99%	3.23%	0.62
	LL	4.04%	4.12%	**13.44%**	2.05%	**4.37%**	
	HL	3.71%	6.59%	2.12%	1.19%	2.23%	
	LH	2.85%	3.64%	2.83%	0.98%	1.44%	

*Cramer's Correlation is calculated between HH, LL zones of LISA map and unsupervised clusters.

Figure 4: Quantitative analysis to find the association between drug LISA maps and identified clusters from complete A2780-1A9 tumor MSI data. Here, each subplot highlights the fraction of pixels present in different zones of the LISA map under two treatment conditions. The red horizontal line in each subplot is a global mean value for pixel ratio for that particular zone. With Beva: pre-treated with bevacizumab and without Beva: without pre-treatment with bevacizumab

Ion signals selection from the identified clusters

For variables selection, the performances of spatial (SE and SL) and non-spatial (OLS) methods were tested on synthetic spatially autocorrelated data. The SL method completely outperformed the other two methods (see Additional File 3); therefore, used for m/z values selection from unsupervised clusters identified in two MSI cancer datasets (Table 2). Note, cluster 4 identified in A2780-1A9 tumor data was present in a single tumor model and cluster 4 and 5 for HCT116 was present in very small fraction and did not follow any proper spatial structure , therefore those clusters were excluded from the ions selection step. The list of important ions is given in Additional file 9. In the A2780 cancer data, cluster 2 had 28 ions showing a significant difference. In particular, the ion at m/z=335.41 had high intensity in all tumor models. The ion image of this particular ion showed a homogeneous distribution in the cluster (see Additional File 6 Figure S-1 top row). A larger number of significantly different ions was identified for cluster 3, mostly with low signal intensity.

For the HCT116 MSI data, a large number of significant ions was identified in cluster 2 and 3. In cluster 3, the majority of ions had a high signal or positive regression coefficients in the spatial model, with the ion at m/z =281.315 showing the highest value. In cluster 2, ions with both high and low signals were present in equal portions (see Additional File 6 Figure S-1 bottom row).

Table 2: The number of ion signals selected from different clusters in two tumor MSI data

Cluster type	A2780	HCT116
1	26	22
2	28	83
3	91	70
5	35	

Discussion

Several studies have shown that the tissue spatial heterogeneity within a solid tumor impacts on the drug distribution[2,10,12,30,46,47]. Different tissue characteristics and tumor microenvironment affect the drug distribution which means that the concentration of a drug at a given spatial location could be related to the tissue composition at that point. It is also true that the presence of the drug can induce a modification of the tumor structure[31], so in general, it is impossible to disentangle the two phenomena. In our case, the situation was fortunate since a part of the tumor-bearing mice had received bevacizumab treatment before drug (paclitaxel) treatment. Therefore, we assume that the observed differences in the spatial organization of tissue areas characterized by similar metabolic fingerprints can be interpreted as a direct or indirect effect of bevacizumab treatment which was given twice before drug injection. This was also suggested by our previous study showing an increase in drug homogeneity in samples treated with bevacizumab[30]. The main goal of this research was to show that computational methods can be used to

explore and quantify spatial heterogeneity within tumors and link the observed homo-/heterogeneous drug distribution to the alteration in microenvironment due to applied therapeutic strategy. To achieve these objectives, our data analysis involved a combination of methods from different research streams. First, the clustering of combined MSI data was performed using K-means with correlation distance and then those clusters were linked with drug distribution patterns obtained using the LISA method. As stated in the introduction, K-means clustering is efficiently being used in several studies performed on MSI data for the selection of relevant clusters[25–27]. Moreover, in another study, the authors also tested this method and compared it with several spatial methods, i.e., using simulated and real data and came to the same conclusion (unpublished observation). In the spatial data analysis field, a LISA map is a commonly known technique that identifies the spatially relevant clusters in a single two-dimensional image[21,22,43]. The LISA map provides an automated way to find spatially homogeneous clusters that are difficult to generate using a simple thresholding approach (see Additional File 8). Simple binary images were created using different threshold values. The images constructed with a threshold value of four show some resemblance with the LISA map (see Additional File 8). The binary images are not fully able to mimic the drug distribution profile. Moreover, the selection of the right threshold value from the drug ion image histogram is not very straightforward. In contrast, the LISA map is able to highlight the observed high and low intensity spatially homogeneous areas in drug ion images efficiently where for spatial weight matrix can be selected based on spatial correlogram.

The clustering of MSI data was able to identify metabolic separated regions which cannot be observed in H&E stained tissue images[25]. This is clear if one compares the images shown in Figure 5. The segmented image shows tissue subtypes in addition to the one which can be associated to the necrotic and fibrous regions on H&E stained image. The effect of the treatment with bevacizumab was visible in the segmented images. In particular, the antiangiogenic compound was increasing homogeneity (both in the pattern of clusters and in the drug) even if no anti-angiogenesis treatment specific cluster was identified (Figure 2 and Additional File 4) Interestingly, these changes in distribution were not affecting drug total concentration, which was approximately equal in the two treatment conditions (see Additional File 7).

This confirmed the results of our previous publication[17] and our homogeneity assessment of clustered images (Figure 2 bottom right) that the tumor tissues from bevacizumab treatment are more homogeneous.

Quantitative analysis of the LISA maps highlights the association between the drug distribution and the tissue metabolic fingerprint (Figure 4 and Additional File 5). In A2780-1A9 tumors, clusters 1 and 2 overlap with the high-high homogeneous region of drug LISA map and cluster 2 was the most homogeneous (Figure 2- bottom right). For HCT116 tumors, clusters 1 and 3 showed some overlap with high-high zone, and cluster 3 was the one showing the higher homogeneity (see Additional File 5). Remarkably, the clusters associated with the HH and LL drug distribution regions are always the same, regardless of the pretreatment with bevacizumab, which is instead affecting the arrangement of the tissue subpopulations.

In summary, in this paper, we provided a computational approach to understand the problem of drug homogeneity in association with tumor heterogeneity which validates the few conclusions made in previous studies. In addition, the proposed framework allowed to robustly select molecular signals which characterize the different tissue subpopulations which can be used to monitor the effects of therapeutic strategies on the tumor spatial heterogeneity. Considering that the focus of our investigation was methodological, we have not dealt with the identification of the characteristic ions, and this would require additional (and extensive) experimental efforts.

Figure 5: Comparison of tumor tissue optical image (left column) with its clustered (middle column) and drug LISA (right column) image from two tumors MSI data (A2780-1A9, HCT116). The black and red dots in H&E stained image represent the necrotic and fibrotic area, respectively. In the above figure, the optical images are adapted from scientific journal[10] published under CC BY license[48]. In the above LISA map HH (high-high), LL (low-low), HL (high-low), and LH (low-high) are zones identified in Moran's I scatter plot.

Conclusions

In cancer research, one of the causes of drug therapy failure is tumor drug resistance often induced by scare drug penetration. This phenomenon is supposed to be linked to the presence of diverse tumor microenvironments which are difficult to identify with established histological techniques. In this work, we show that a molecular imaging technique like MSI, coupled with advanced data analysis strategies, offers a great opportunity to investigate the link between drug distribution and tissue heterogeneity. Our approach allowed to simultaneously investigate tissue histology and drug distribution and it was capable of detecting the effects on the tumor heterogeneity induced by a specific intervention (treatment with bevacizumab). We hope that the unsupervised approach proposed here will help oncologists to quantitatively evaluate the efficacy of therapeutic strategies.

ASSOCIATED CONTENT

Availability of supporting data

The datasets and R-script used to generate the results of this article are available on the DNAS-KNAW repository (https://doi.org/10.17026/dans26h-kstf).

Additional files

Additional File 1: Figures show workflow of adaptive bins creation and maximum intensity spectrum from two tumor models.

Additional File 2: Description of the statistical methods used in this manuscript.

Additional File 3: Synthetic spatially autocorrelated data generation steps and comparison of spatial and non-spatial methods for variables selection on synthetic data.

Additional File 4: Cluster analysis of HCT116 MSI. Range and significance values for pixels and homogeneity ratio from different clusters under two treatment condition.

Additional File 5: Overlay of clustered image and LISA map and contingency table from HCT116 MSI data. And, the quantitative analysis of LISA maps for HCT116 tumor MSI data.

Additional File 6: MS images of selected molecular ions from different clusters from tumor MSI data.

Additional File 7: Plot of drug concentration in different clusters.

Additional File 8: Comparison of drug binary image created by selecting manual threshold value based on histogram and LISA method.

Additional file 9: Excel sheet contains selected important ion signals from identified clusters of our cancer MSI data.

AUTHOR INFORMATION

Corresponding Author

* E-mail: chemometrics@science.ru.nl

Competing Interests

The authors declare no competing interests.

Author Contributions

MP analyzed MSI data and wrote first draft of the paper under supervision of GP, PF, JJ and LB. GP, PF, and JJ edit the manuscript. ED, LM, SG, FF and RG did MSI experiment and review the manuscript. All authors read and approved the final manuscript.

REFERENCES

(1) Minchinton, A. I.; Tannock, I. F. Drug Penetration in Solid Tumours. *Nat. Rev. Cancer* **2006**, *6* (8), 583–592. https://doi.org/10.1038/nrc1893.

(2) Trédan, O.; Galmarini, C. M.; Patel, K.; Tannock, I. F. Drug Resistance and the Solid Tumor Microenvironment. *J. Natl. Cancer Inst.* **2007**, *99* (19), 1441–1454. https://doi.org/10.1093/jnci/djm135.

(3) Zhao, B.; Hemann, M. T.; Lauffenburger, D. A. Intratumor Heterogeneity Alters Most Effective Drugs in Designed Combinations. *Proc. Natl. Acad. Sci. U. S. A.* **2014**, *111* (29), 10773–10778. https://doi.org/10.1073/pnas.1323934111.

(4) Heindl, A.; Nawaz, S.; Yuan, Y. Mapping Spatial Heterogeneity in the Tumor Microenvironment: A New Era for Digital Pathology. *Lab. Investig.* **2015**, *95* (4), 377–384. https://doi.org/10.1038/labinvest.2014.155.

(5) Wiseman, J. M.; Ifa, D. R.; Zhu, Y.; Kissinger, C. B.; Manicke, N. E.; Kissinger, P. T.; Cooks, R. G. Desorption Electrospray Ionization Mass Spectrometry: Imaging Drugs and Metabolites in Tissues. *Proc. Natl. Acad. Sci.* **2008**, *105* (47), 18120–18125. https://doi.org/10.1073/pnas.0801066105.

(6) Prideaux, B.; Stoeckli, M. Mass Spectrometry Imaging for Drug Distribution Studies. *J. Proteomics* **2012**, *75* (16), 4999–5013. https://doi.org/10.1016/j.jprot.2012.07.028.

(7) Jones, E. A.; van Remoortere, A.; van Zeijl, R. J. M.; Hogendoorn, P. C. W.; Bovée, J. V. M. G.; Deelder, A. M.; McDonnell, L. A. Multiple Statistical Analysis Techniques Corroborate Intratumor Heterogeneity in Imaging Mass Spectrometry Datasets of Myxofibrosarcoma. *PLoS One* **2011**, *6* (9), e24913. https://doi.org/10.1371/journal.pone.0024913.

(8) Abdelmoula, W. M.; Balluff, B.; Englert, S.; Dijkstra, J.; Reinders, M. J. T.; Walch, A. Data-Driven Identification of Prognostic Tumor Subpopulations Using Spatially Mapped t-SNE of Mass Spectrometry Imaging Data. *PNAS* **2016**, *113* (43), 12244–12249. https://doi.org/10.1073/pnas.1510227113.

(9) Inglese, P.; McKenzie, J. S.; Mroz, A.; Kinross, J.; Veselkov, K.; Holmes, E.; Takats, Z.; Nicholson, J. K.; Glen, R. C. Deep Learning and 3D-DESI Imaging Reveal the Hidden Metabolic Heterogeneity of Cancer. *Chem. Sci.* **2017**, *8* (5), 3500–3511. https://doi.org/10.1039/c6sc03738k.

(10) Giordano, S.; Morosi, L.; Veglianese, P.; Licandro, S. A.; Frapolli, R.; Zucchetti, M.; Cappelletti, G.; Falciola, L.; Pifferi, V.; Visentin, S.; et al. 3D Mass Spectrometry Imaging Reveals a Very Heterogeneous Drug Distribution in Tumors. *Sci. Rep.* **2016**, *6* (1), 37027. https://doi.org/10.1038/srep37027.
(11) Thompson, C. G.; Bokhart, M. T.; Sykes, C.; Adamson, L.; Fedoriw, Y.; Luciw, P. A.; Muddiman, D. C.; Kashuba, A. D. M.; Rosen, E. P. Mass Spectrometry Imaging Reveals Heterogeneous Efavirenz Distribution within Putative HIV Reservoirs. *Antimicrob. Agents Chemother.* **2015**, *59* (5), 2944–2948. https://doi.org/10.1128/AAC.04952-14.
(12) Giordano, S.; Zucchetti, M.; Decio, A.; Cesca, M.; Fuso Nerini, I.; Maiezza, M.; Ferrari, M.; Licandro, S. A.; Frapolli, R.; Giavazzi, R.; et al. Heterogeneity of Paclitaxel Distribution in Different Tumor Models Assessed by MALDI Mass Spectrometry Imaging. *Sci. Rep.* **2016**, *6* (1), 39284. https://doi.org/10.1038/srep39284.
(13) Walch, A.; Rauser, S.; Deininger, S.-O.; Höfler, H. MALDI Imaging Mass Spectrometry for Direct Tissue Analysis: A New Frontier for Molecular Histology. *Histochem. Cell Biol.* **2008**, *130* (3), 421–434. https://doi.org/10.1007/s00418-008-0469-9.
(14) Balluff, B.; Frese, C. K.; Maier, S. K.; Schöne, C.; Kuster, B.; Schmitt, M.; Aubele, M.; Höfler, H.; Deelder, A. M.; Heck, A. J.; et al. De Novo Discovery of Phenotypic Intratumour Heterogeneity Using Imaging Mass Spectrometry. *J. Pathol.* **2015**, *235* (1), 3–13. https://doi.org/10.1002/path.4436.
(15) Cassese, A.; Ellis, S. R.; Ogrinc Potočnik, N.; Burgermeister, E.; Ebert, M.; Walch, A.; van den Maagdenberg, A. M. J. M.; McDonnell, L. A.; Heeren, R. M. A.; Balluff, B. Spatial Autocorrelation in Mass Spectrometry Imaging. *Anal. Chem.* **2016**, *88* (11), 5871–5878. https://doi.org/10.1021/acs.analchem.6b00672.
(16) Inglese, P.; McKenzie, J. S.; Mroz, A.; Kinross, J.; Veselkov, K.; Holmes, E.; Takats, Z.; Nicholson, J. K.; Glen, R. C. Deep Learning and 3D-DESI Imaging Reveal the Hidden Metabolic Heterogeneity of Cancer. *Chem. Sci.* **2017**, *8* (5), 3500–3511. https://doi.org/10.1039/c6sc03738k.
(17) Prasad, M.; Postma, G.; Morosi, L.; Giordano, S.; Giavazzi, R.; D'Incalci, M.; Falcetta, F.; Davoli, E.; Jansen, J.; Franceschi, P. Drug-Homogeneity Index in Mass-Spectrometry Imaging. *Anal. Chem.* **2018**, *90* (22), 13257–13264. https://doi.org/10.1021/acs.analchem.8b01870.
(18) Trede, D.; Schiffler, S.; Becker, M.; Wirtz, S.; Steinhorst, K.; Strehlow, J.; Aichler, M.; Kobarg, J. H.; Oetjen, J.; Dyatlov, A.; et al. Exploring Three-Dimensional Matrix-Assisted Laser Desorption/Ionization Imaging Mass Spectrometry Data: Three-Dimensional Spatial Segmentation of Mouse Kidney. *Anal. Chem.* **2012**, *84* (14), 6079–6087. https://doi.org/10.1021/ac300673y.
(19) Calligaris, D.; Norton, I.; Feldman, D. R.; Ide, J. L.; Dunn, I. F.; Eberlin, L. S.; Cooks, R. G.; Jolesz, F. A.; Golby, A. J.; Agar, N. Y. Mass Spectrometry Imaging as a Tool for Surgical Decision- Making. *J. Mass Spectrom.* **2013**, *48* (11), 1178–1187. https://doi.org/10.1002/jms.3295.
(20) Franceschi, P.; Wehrens, R. Self-Organizing Maps: A Versatile Tool for the Automatic Analysis of Untargeted Imaging Datasets. *Proteomics* **2014**, *14* (7–8), 853–861. https://doi.org/10.1002/pmic.201300308.
(21) Anselin, L. Local Indicators of Spatial Association-LISA. *Geogr. Anal.* **2010**, *27* (2), 93–115. https://doi.org/10.1111/j.1538-4632.1995.tb00338.x.
(22) Lin, W. C.; Lin, Y. P.; Wang, Y. C.; Chang, T. K.; Chiang, L. C. Assessing and Mapping Spatial Associations among Oral Cancer Mortality Rates, Concentrations of Heavy Metals in Soil, and Land Use Types Based on Multiple Scale Data. *Int. J. Environ. Res. Public Health* **2014**, *11* (2), 2148–2168. https://doi.org/10.3390/ijerph110202148.
(23) Sheskin, D. J. *Handbook of Parametric and Nonparametric Statistical Procedures*;

Chapman and Hall/CRC, 2007; Vol. 51. https://doi.org/10.2307/2685909.
(24) Bivand, R. S.; Pebesma, E. J.; Gomez-Rubio, V. *Applied Spatial Data Analysis with R*; Springer New York: New York, NY, 2008. https://doi.org/10.1007/978-0-387-78171-6.
(25) Trede, D.; Schiffler, S.; Becker, M.; Wirtz, S.; Steinhorst, K.; Strehlow, J.; Aichler, M.; Kobarg, J. H.; Oetjen, J.; Dyatlov, A.; et al. Exploring Three-Dimensional Matrix-Assisted Laser Desorption/Ionization Imaging Mass Spectrometry Data: Three-Dimensional Spatial Segmentation of Mouse Kidney. *Anal. Chem.* **2012**, *84* (14), 6079–6087. https://doi.org/10.1021/ac300673y.
(26) Widlak, P.; Mrukwa, G.; Kalinowska, M.; Pietrowska, M.; Chekan, M.; Wierzgon, J.; Gawin, M.; Drazek, G.; Polanska, J. Detection of Molecular Signatures of Oral Squamous Cell Carcinoma and Normal Epithelium - Application of a Novel Methodology for Unsupervised Segmentation of Imaging Mass Spectrometry Data. *Proteomics* **2016**, *16* (11–12), 1613–1621. https://doi.org/10.1002/pmic.201500458.
(27) Louie, K. B.; Bowen, B. P.; McAlhany, S.; Huang, Y.; Price, J. C.; Mao, J.; Hellerstein, M.; Northen, T. R. Mass Spectrometry Imaging for in Situ Kinetic Histochemistry. *Sci. Rep.* **2013**, *3* (1), 1656. https://doi.org/10.1038/srep01656.
(28) Ng, N.; Sandberg, M.; Ahlström, G. Prevalence of Older People with Intellectual Disability in Sweden: A Spatial Epidemiological Analysis. *J. Intellect. Disabil. Res.* **2015**, *59* (12), 1155–1167. https://doi.org/10.1111/jir.12219.
(29) Wang, T.; Wang, X.; Tie, P.; Bai, Y.; Zheng, Y.; Yan, C.; Chai, Z.; Chen, J.; Rao, H.; Zeng, L.; et al. Spatio-Temporal Cluster and Distribution of Human Brucellosis in Shanxi Province of China between 2011 and 2016. *Sci. Rep.* **2018**, *8* (1), 1–10. https://doi.org/10.1038/s41598-018-34975-7.
(30) Cesca, M.; Morosi, L.; Berndt, A.; Fuso Nerini, I.; Frapolli, R.; Richter, P.; Decio, A.; Dirsch, O.; Micotti, E.; Giordano, S.; et al. Bevacizumab-Induced Inhibition of Angiogenesis Promotes a More Homogeneous Intratumoral Distribution of Paclitaxel, Improving the Antitumor Response. *Mol. Cancer Ther.* **2016**, *15* (1), 125–135. https://doi.org/10.1158/1535-7163.MCT-15-0063.
(31) Bai, J.; Wang, M. X.; Chowbay, B.; Ching, C. B.; Chen, W. N. Metabolic Profiling of HepG2 Cells Incubated with S(-) and R(+) Enantiomers of Anti-Coagulating Drug Warfarin. *Metabolomics* **2011**, *7* (3), 353–362. https://doi.org/10.1007/s11306-010-0262-3.
(32) Team, R. C. R: A Language and Environment for Statistical Computing. **2017**.
(33) Gibb, S.; Strimmer, K. Maldiquant: A Versatile R Package for the Analysis of Mass Spectrometry Data. *Bioinformatics* **2012**, *28* (17), 2270–2271. https://doi.org/10.1093/bioinformatics/bts447.
(34) Gong, L.; Constantine, W.; Chen, Y. A. MsProcess: Protein Mass Spectra Processing. **2012**, *R package*.
(35) McDonnell, L. A.; van Remoortere, A.; van Zeijl, R. J. M.; Deelder, A. M. Mass Spectrometry Image Correlation: Quantifying Colocalization. *J. Proteome Res.* **2008**, *7* (8), 3619–3627. https://doi.org/10.1021/pr800214d.
(36) Rocke D, Lee GC, Tillinghast J, Durbin-Johnson B, W. S. LMGene: LMGene Software for Data Transformation and Identification of Differentially Expressed Genes in Gene Expression Arrays. **2018**, *R package*.
(37) Smyth, G. K.; Ritchie, M.; Thorne, N. Linear Models for Microarray and RNA-Seq Data User ' s Guide. **2018**, No. April.
(38) Steinbach, M.; Karypis, G.; Kumar, V. A Comparison of Document Clustering Techniques. In *In KDD Workshop on Text Mining*; 2000. https://doi.org/10.1109/ICCCYB.2008.4721382.
(39) Calinski, T.; Harabasz, J. A Dendrite Method for Cluster Analysis. *Commun. Stat. -*

Theory Methods **1974**, *3* (1), 1–27. https://doi.org/10.1080/03610927408827101.
(40) Lucas, M. A. Amap: Another Multidimensional Analysis Package. **2018**, No. R package version 0.8-16.
(41) Henning, C. Fpc: Flexible Procedures for Clustering. **2018**, No. R package version 2.1-11.1.
(42) Pinheiro J, Bates D, DebRoy S, S. D. and R. C. T. (2017). Nlme: Linear and Nonlinear Mixed Effects Models. **2017**, *R package*.
(43) Plant, R. E. *Spatial Data Analysis in Ecology and Agriculture Using R*; Intergovernmental Panel on Climate Change, Ed.; Cambridge University Press: Cambridge, 2012. https://doi.org/10.1017/CBO9781107415324.004.
(44) Kissling, W. D.; Carl, G. Spatial Autocorrelation and the Selection of Simultaneous Autoregressive Models. **2008**, 59–71. https://doi.org/10.1111/j.1466-8238.2007.00334.x.
(45) Franceschi, P.; Giordan, M.; Wehrens, R. Multiple Comparisons in Mass-Spectrometry-Based -Omics Technologies. *TrAC Trends Anal. Chem.* **2013**, *50*. https://doi.org/https://doi.org/10.1016/j.trac.2013.04.011.
(46) Fuso Nerini, I.; Morosi, L.; Zucchetti, M.; Ballerini, A.; Giavazzi, R.; D'incalci, M. Intratumor Heterogeneity and Its Impact on Drug Distribution and Sensitivity. *Clin. Pharmacol. Ther.* **2014**, *96* (2), 224–238. https://doi.org/10.1038/clpt.2014.105.
(47) Thompson, C. G.; Bokhart, M. T.; Sykes, C.; Adamson, L.; Fedoriw, Y.; Luciw, P. A.; Muddiman, D. C.; Kashuba, A. D. M.; Rosen, E. P. Mass Spectrometry Imaging Reveals Heterogeneous Efavirenz Distribution within Putative HIV Reservoirs. *Antimicrob. Agents Chemother.* **2015**, *59* (5), 2944–2948. https://doi.org/10.1128/AAC.04952-14.
(48) Creative Commons Attribution 4.0 International License.

Click here to download Figure Figure1.docx

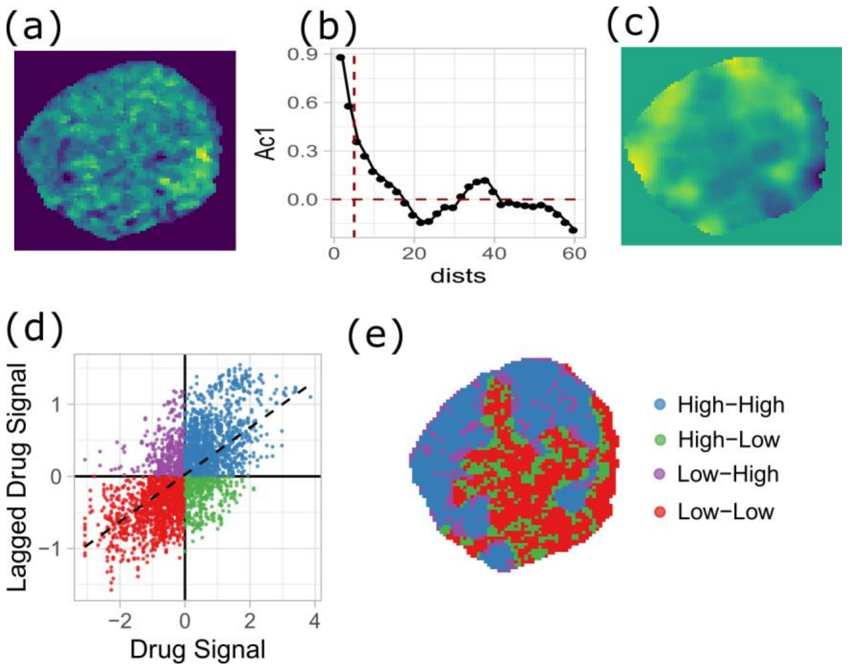

Click here to download Figure Figure2.docx

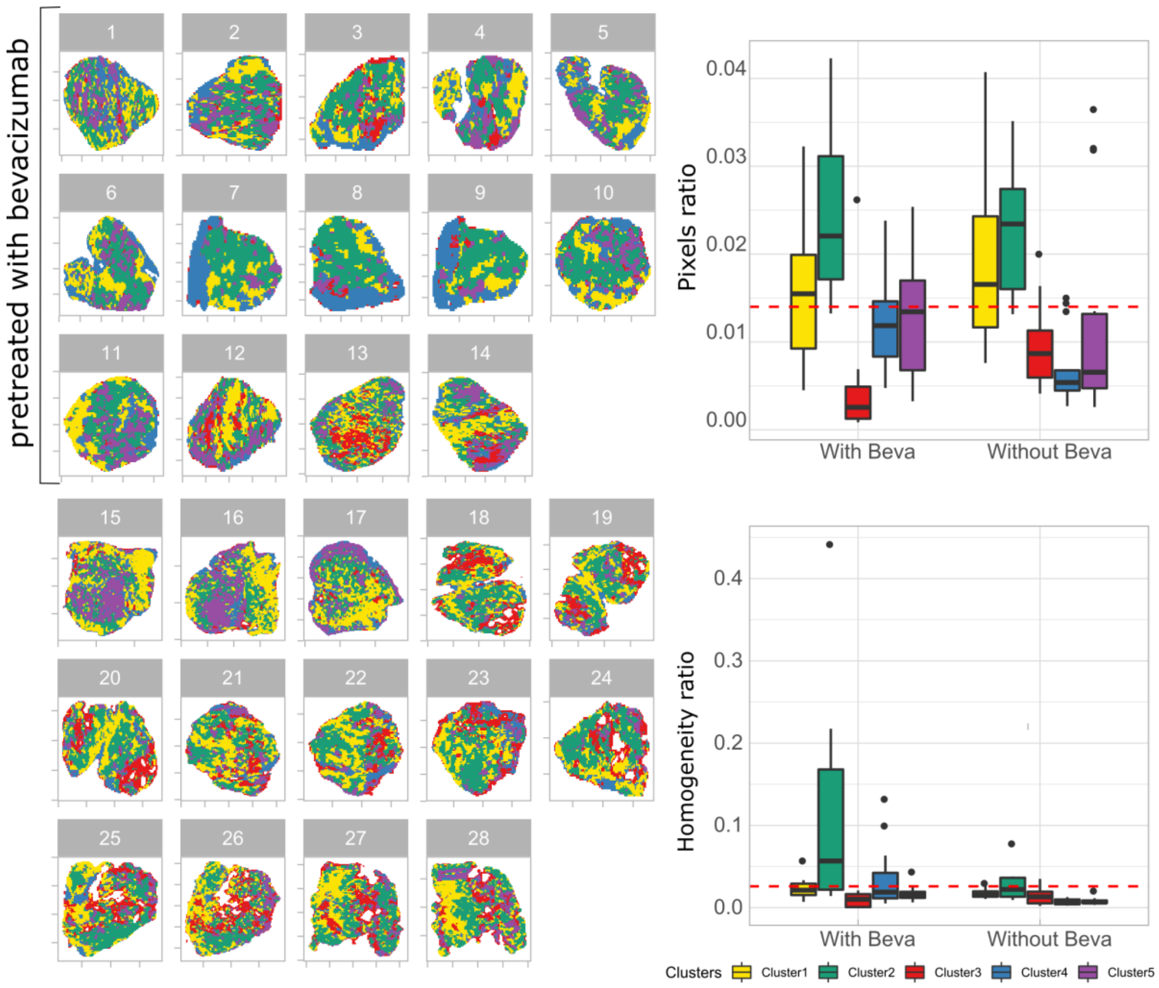

Click here to download Figure Figure3.docx

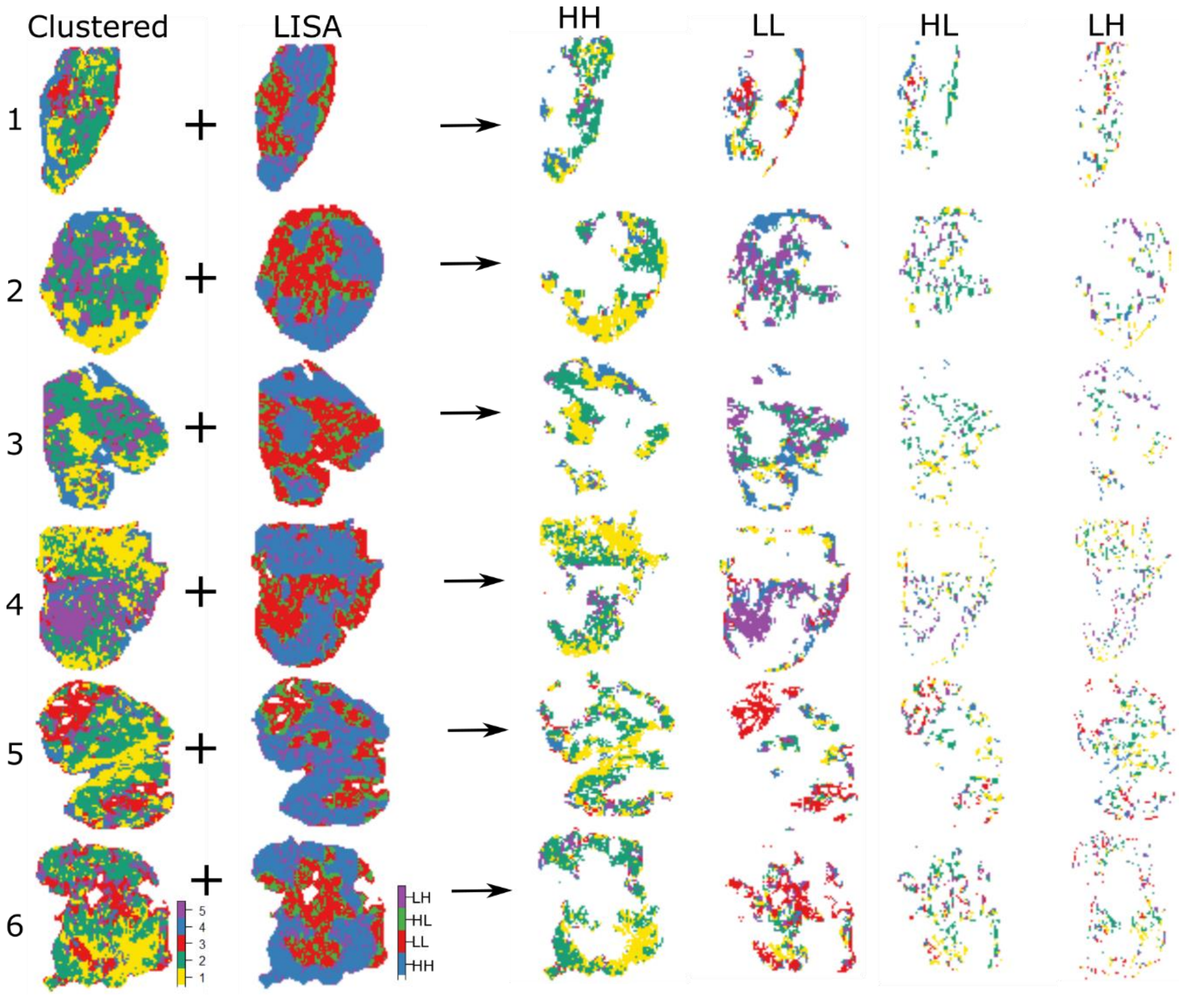

Click here to download Figure Figure4.docx

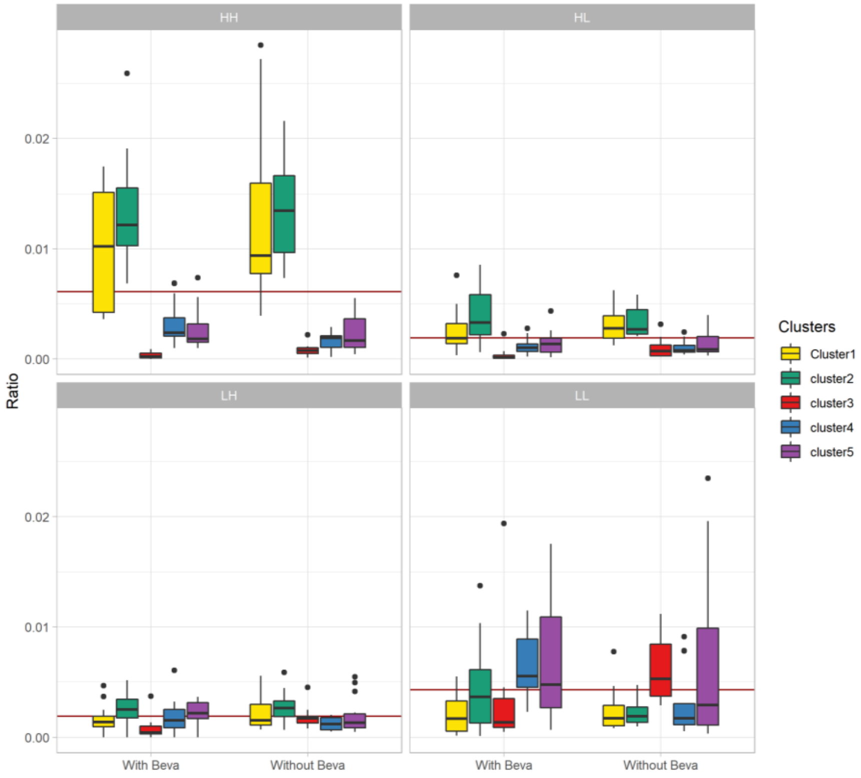

Click here to download Figure Figure5.docx

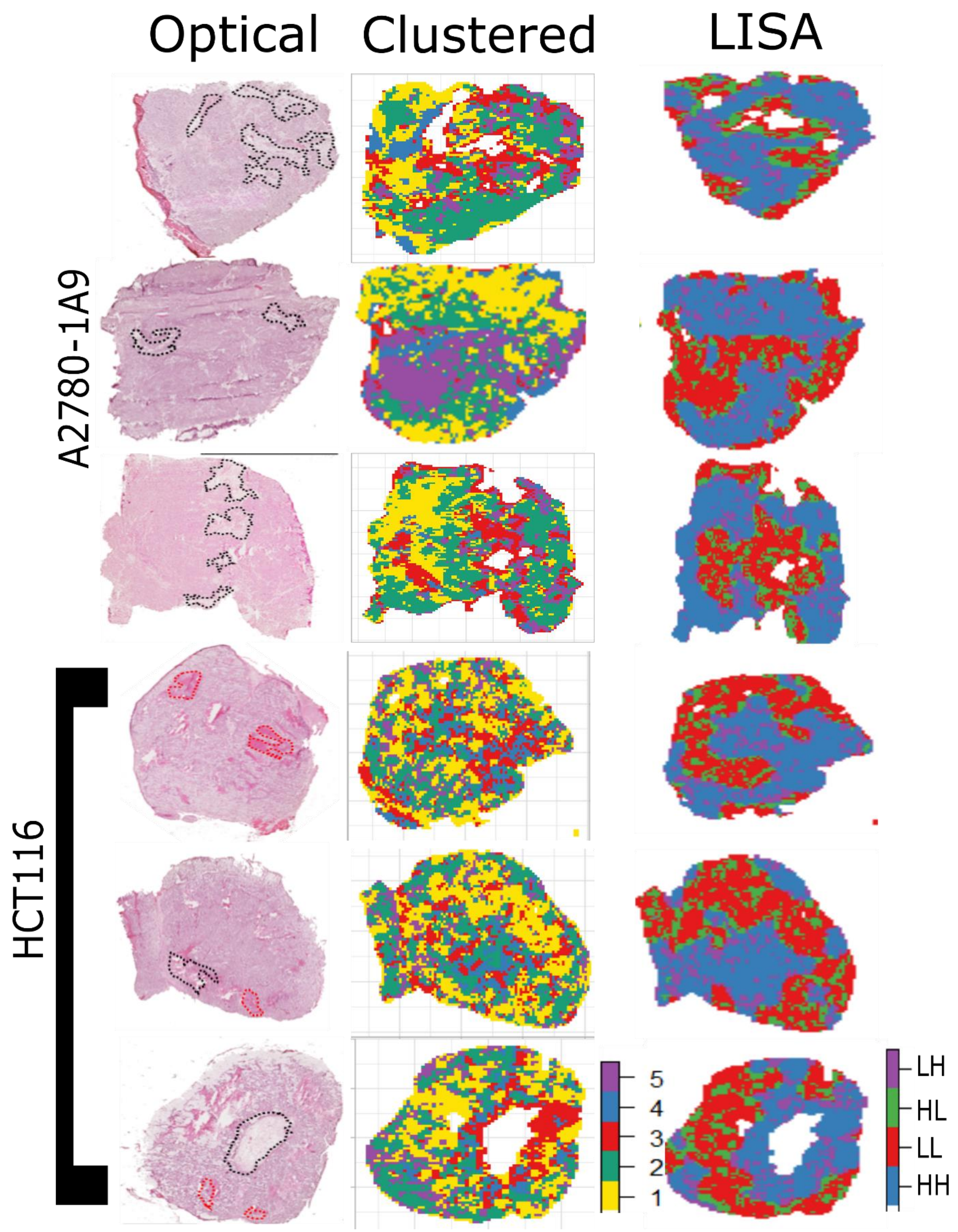

Click here to access/download
Supplementary Material
AdditionalFile1.docx

Click here to access/download
Supplementary Material
AdditionalFile3.docx

Click here to access/download
Supplementary Material
AdditionalFile4.docx

Click here to access/download
Supplementary Material
AdditionalFile5.docx

Click here to access/download
Supplementary Material
AdditionalFile6.docx

Click here to access/download
Supplementary Material
AdditionalFile7.docx

Click here to access/download
Supplementary Material
AdditionalFile8.docx

Click here to access/download
Supplementary Material
AdditionalFile9.xlsx

Click here to download Personal Cover AdditionalFile2.docx

Text S-1

Moran index

Moran's I provides a global measure of spatial autocorrelation which is computed using the following formula[1]:

$$I = \frac{n}{\sum_{i=1}^{n}(y_i - \bar{y})^2} \frac{\sum_{i=1}^{n}\sum_{j=1}^{n} w_{ij}(y_i - \bar{y})(y_j - \bar{y})}{\sum_{i=1}^{n}\sum_{j=1}^{n} w_{ij}}$$

where n equals the total number of observations in the study area, y_i is the value of the variable at site i, $\underline{y}$ is the mean value of the variable, w_{ij} represents the spatial weights. Moran's I value is highly dependent upon spatial weight matrix w_{ij} description. One possibility of defining the neighbors (w_{ij}=1) is to use a distance threshold, for example, two sites are considered as neighbors if the distance between them is smaller than or equal to some threshold. If most similar observation lies next to each other then Moran's I returns high spatial autocorrelation value which is close to 1 and vice-versa.

Moran scatter plot

Moran scatter plot provides a visual representation of spatial associations in the neighborhood around each observation[1]. It plots the original value of the observations on the horizontal axis and it's spatially lagged or weighted version on the y-axis. Moran scatters plot contains four spatial regions or quadrants defined by the horizontal line y=0 and the vertical line x=0. Points in the upper right (or high-high) and lower left (or low-low) quadrants indicate positive spatial association that are the higher and lower than the sample mean, respectively. The lower right (or high-low) and upper left (or low-high) quadrants include observations that exhibit negative spatial association, i.e. little similarity to their neighboring ones. Identify map regions based on Moran scatter plot collectively called as local indicators of spatial association (LISA)[2].

Homogeneity index (HI)

Homogeneity index (HI) formula, used to measure the homogeneous distribution of different tissue types in our clustered image, is based on our drug homogeneity index (DHI)[3] formula. Instead of summing the value across all grey-levels (as in original formula) we calculate the value for individual grey-level or cluster class using below formula:

$$HI = \frac{[\sum_{j=N_u}^{N_z} jP(i,j)]_{i=1,..,n}}{\sum_{i=1}^{n}\sum_{j=N_u}^{N_z} P(i,j)}$$

Here, $P(i,j)$ is the gray-level size-zone matrix (GLSZM) contains gray-levels or number of clusters (n) for this particular study as rows and the size zones (N_z) as columns. Numerator of HI shows homogeneity value for individual cluster class which is further normalized based on the size of GLSZM to get the contribution of the particular cluster in a given image.

References

(1) Bivand, R. S.; Pebesma, E. J.; Gomez-Rubio, V. *Applied Spatial Data Analysis with R*; Springer New York: New York, NY, **2008**. https://doi.org/10.1007/978-0-387-78171-6.

(2) Anselin, L. Local Indicators of Spatial Association-LISA. *Geogr. Anal.* **2010**, *27* (2), 93–115. https://doi.org/10.1111/j.1538-4632.1995.tb00338.x.

(3) Prasad, M.; Postma, G.; Morosi, L.; Giordano, S.; Giavazzi, R.; D'Incalci, M.; Falcetta, F.; Davoli, E.; Jansen, J.; Franceschi, P. Drug-Homogeneity Index in Mass-Spectrometry Imaging. *Anal. Chem.* **2018**, *90* (22), 13257–13264. https://doi.org/10.1021/acs.analchem.8b01870.
