# Supplementary material for: A methodological approach to correlate tumor heterogeneity with drug distribution profile in mass spectrometry imaging data
Source: Gigascience. 2020 Nov 25;9(11):giaa131. doi: 10.1093/gigascience/giaa131 (PMC7688471; doi:10.1093/gigascience/giaa131)
Supplement: giaa131_GIGA-D-20-00114_Revision_1 [file giaa131_giga-d-20-00114_revision_1.pdf]

GigaScience

A methodological approach to correlate tumor heterogeneity with drug distribution profile in mass spectrometry imaging (MSI) data

--Manuscript Draft--

**Manuscript Number:**	GIGA-D-20-00114R1
**Full Title:**	A methodological approach to correlate tumor heterogeneity with drug distribution profile in mass spectrometry imaging (MSI) data
**Article Type:**	Research
**Funding Information:**	
**Abstract:**	Mass spectrometry imaging (MSI) has become a valuable tool in drug imaging because of its ability to provide a simultaneous spatial distribution of the drug and several other molecular ions present in the biological sample. An important application is the evaluation of homogeneity/heterogeneity of drug distribution in solid tumors. Solid tumors are known to be made up of different tissue subpopulations and their heterogeneity is supposed to have a direct and/or indirect influence on drug distribution. Hence, for further enhancement of penetration therapy performance, it is important to link a characterization of the tumor microenvironment with drug homogeneity. In this study, untargeted MSI data were used to understand the spatial heterogeneity within solid tumors, assessing its impact on the drug (paclitaxel) distribution. The proposed approach was applied on MSI datasets already analyzed, focusing on tumor drug distribution. Untargeted MSI datasets were collected on different tumor xenograft models (ovarian and colon cancer cell lines) pre-treated or not with anti-angiogenesis compound (bevacizumab). Our main data analysis steps involved: a) pre-processing of MSI data to make all biological samples directly comparable, b) unsupervised data clustering to find different tissue subtypes, c) quantification of drug heterogeneity using local indicators of spatial association (LISA) map and d) selection of important ion signals from identified clusters of interest using the spatial -aware statistical tools. Our clustering results show variation in tumor subpopulations and less spatial heterogeneity in the MSI data collected on samples treated with the anti-angiogenesis compound consistently with our previous data. The local spatial structures identified in drug ion LISA maps show a correlation with clusters identified using a clustering method. Using the right spatial method, we were able to reduce the number of false-positive ions selected and identified the one that shows relevant spatial patterns in different tissue subtypes. Finally, our overall study shows that there is a direct association in drug homogeneity and spatial arrangement of different tissue subtypes in a solid tumor.
**Corresponding Author:**	Geert Postma<br>Radboud Universiteit<br>Nijmegen, NETHERLANDS
**Corresponding Author Secondary Information:**	
**Corresponding Author's Institution:**	Radboud Universiteit
**Corresponding Author's Secondary Institution:**	
**First Author:**	Mridula Prasad, M.Sc.
**First Author Secondary Information:**	
**Order of Authors:**	Mridula Prasad, M.Sc.
	Geert Postma
	Pietro Franceschi
	Lavinia Morosi
	Silvia Giordano
	Francesca Falcetta
	Raffaella Giavazzi

Powered by Editorial Manager® and ProduXion Manager® from Aries Systems Corporation

	Enrico Davoli
	Lutgarde M.C. Buydens
	Jeroen Jansen
**Order of Authors Secondary Information:**	
**Response to Reviewers:**	Dear Editor.<br>We would like to thank the reviewers' for their constructive comments and suggestions. We are including a point-by-point discussion of their comments hereafter. We also revised our manuscript accordingly wherever it was required. The R package from our paper is online available and registered at bio.tools and Scicrunch (bio.tools: corrdrugtumormsi, RRID : SCR_018962). We believe all these changes increase the reproducibility of our paper and now is suitable to publish in the Gigascience journal.<br><br>Please consult the uploaded Personal Cover file 'EditorLetter.docx' for the response to the reviewers with figures as additional support of our response and the Peronal Cover file 'Fequency ofmolecularIonsInMSIdata.xlsx' with some data mentioned in our repose to the reviewers.<br><br>Yours sincerely<br>Dr. Geert Postma<br><br>Reviewer #1:<br>Question 1.1 The authors present a computational method for identifying spatial regions with molecularly distinct regions between control and drug therapy using previously published data. The method is well described and paper is somewhat easy to follow. The code and attached data was reviewed as well and appears clear and would be easily translatable to other projects. A more formal implementation as an R package would be desirable as the workflow is quite complex it would benefit to make a more accessible API so less experienced users wouldn't get lost.<br>Answer: A R package is developed that includes all main methods from our paper (bio.tools: corrdrugtumormsi, RRID : SCR_018962). Interested users can install our R-package from the Github website and refer to vignette for the usage of different functions in our package.<br><br>Question 1.2 For step 1 in the processing: How are 'drug' related peaks guaranteed to be removed from the 'microenvironment' segmentation? In processing, it is mentioned that ion peaks with correlation > 0.5 with the drug compound were removed. This seems like it would bias the segmentation if the drug had a very discrete distribution in a very particular histological region. One can imagine a scenario where a drug is distributed in area "A" exclusively along with other endogenous compounds. These endogenous compounds would be then be removed from the segmentation pipeline simply because the drug was highly partitioned into this region. Could the peaks be derived solely from undosed control tissue? Otherwise the authors statement may be misleading.<br>Answer: We agree with the reviewer's comment that the selection of drug-related peaks based on the correlation approach could give biased results. Unfortunately, in our study, we only had one control MSI data set per cell line, and the selection and removal of drug-related peaks based on a single animal also seem quite biased approach. Therefore, we used a heuristic approach where we removed the peaks that show more than 0.5 correlation with drug ion peak and also the peaks present in less than 20% tumor tissue area. In this way, we expected to have not too many ions with discrete distribution and ions with a high correlation with drug ion peak removed. We have validated this approach, see below.<br>Drug-related peaks in control animal:<br>The ion intensity distribution of removed peaks in a control tissue from two tumor MSI data (A2780-1A9, HCT116) is shown in Figure 1 (see Personal Cover file 'EditorLetter.docx'). Since the control animal has not been treated with drug compound we do not expect those removed peaks to be present in MSI data. But, unfortunately, that is not the case, in the figure, the drug (284.12) and its isotopic peaks (285.155) are completely absent in the control animal and other removed peaks are visible. Therefore, it seems that the threshold of 0.5 we used in our study is quite low and we need a much higher threshold to avoid removing non-drug-related peaks. On using a correlation threshold of 0.9 only the drug isotopic peak is removed from our data,

Powered by Editorial Manager® and ProduXion Manager® from Aries Systems Corporation

therefore instead of 0.5, we will recommend the minimum correlation threshold of 0.9 in our paper.
Apart from the above analysis, we also checked whether specific peaks which could be considered as drug-related peaks were absent in the control tumor MSI data but present in the treated animal data. We did not notice such peaks. There were few empty bins in the control MSI data but those bins are also empty in the majority of treated animals. This observation was made for both tumor MSI datasets. See added file 'FrequencyofmoelcularIonsInMSIdata.xls' where the count of the pixel with non-zero ion-intensity value across all tumor models is given.
We also looked at the impact of the removed peaks on the segmentation results. An impact of the removed peaks on the segmentation results is expected if the removed peaks are related to a unique spatial structure. The correlation between removed and other remaining peaks was calculated. All removed peaks (except for drug and its isotopic peaks) show a high correlation with many other peaks. Therefore, we don't expect our segmentation results to be biased with our approach. This statement is confirmed by the segmentation of the control tissue. The clustering of control MSI data was performed in both scenarios: with and without drug-related peaks (Figure 2, see Personal Cover file 'EditorLetter.docx') similar cluster structures were derived.

Figure 1, Figure 2, see Personal Cover file 'EditorLetter.docx'

Based on the analysis on control MSI data we agree with the reviewer that our statement regarding the removal of drug-related peaks based on correlation >0.5 is not sufficient therefore in the absence of control tissue a high correlation threshold (0.9) should be used.

Question 1.3 The authors note that mass spectral validation of model-identified differential ions is not possible and that is reasonable. In general, the spatial models presented in the findings are compelling. However, as this paper deals with spatial characterization of tissue, there appears to be no spatial validation. Indeed the obvious choice of the gold standard in pathology, H&E microscopy, is present in Figure 5 but the size of the images is so small it is negligible for spatial validation. Secondly, there are numerous published MSI examples(DOI: 10.1021/jasms.8b04879, doi:10.1074/mcp.O115.053918, https://doi.org/10.1038/srep36814) where there are clean and distinct, immediate visual association of segmented MSI images to histological regions in H&E, but here the segmentation doesn't seem to replicate much of the structure visible in Figure 5, at least AS PRESENTED. This comment isn't to push for models integrating H&E as an input but to have some qualitative result describing
the types of cells present in the tumor regions associated with the major clusters. While molecular histology is valid, it is unusual for it to not mimic classical histology.
Minor comments
All figures containing should have a scale bar indicating the physical dimension of the images.

Answer: We agree with the reviewer's comment that there needs to be more evidence regarding clusters identity and tissue types present in our dataset. However, the MSI data used in this study was initially generated to understand drug homogeneity in different types of solid tumors, and there was little histology-related work done to identify the tissue types. At this stage histological informations available, like proliferation/necrosis, vessels1 allowed us to correlate only what has been discussed in the text. Future studies will be aimed at the validation of different clustering features presented in this work with more specific, dedicated histological studies.
The reviewer's comment is correct that the optical image in Figure 5 does not show proper correlation with our clustered images, therefore we will remove all images except images 2 and 5 were necrosis region is visible both in the optical image and our clustered images.

Small errors:
Introduction
* Techniques of election <- is not proper English. Perhaps 'A valuable technique' would be less awkward.
Answer: modified accordingly.

Powered by Editorial Manager® and ProduXion Manager® from Aries Systems Corporation

Reviewer #2:
This is an interesting paper addressing how MSI can be effectively used to better understand the link between drug and the characteristics of malignant tissue. While it is not surprising that the physicochemical properties of both the tissue and the drug are important to passive tumor penetration and local exposure, MSI provides an important opportunity to understand the spatial and temporal dynamics of this process, and the development of effective computational workflows is vital. A few questions/suggestions for the authors follow:

Question 2.1 To what extent can the most prominent histochemical changes occurring post-bevacizumab treatment be captured by m/z range and other experimental settings studied in this untargeted MSI experiment? Since it used a limited mass range, certain important changes (e.g., in cell-surface protein expression, lipid membrane composition, etc.) may not have been measured. The authors may have addressed this question in their cited previous work, but it would be helpful to provide some additional context.
Answer: We agree with the reviewer, our MSI data cover a limited mass range where we cannot see certain changes. However, the MSI data used in this study was initially generated to understand drug homogeneity in different types of solid tumors, and there was little histology-related work done to identify the tissue types. Our major focus, at this stage was not in a comprehensive metabolite study, but in methodology development. Future studies will be focussed, with more specific histology, on local metabolic effects of drugs and larger mass range will be used¬¬¬

Question 2.2 Since the focus of this paper is methodology, evaluation of the approach against known ground-truths is critical. In that regard, the efforts of the authors in developing a synthetic dataset and evaluating the methods on it is appreciated. There are a few ways that this assessment could be expanded to provide additional information about the robustness of the workflow. For example, Additional File 3 includes plots showing the synthetic data and some of its characteristics, but to what extent do the statistical properties of the synthetic data compare with those of real MSI datasets? The SL method was recommended, but how sensitive is it to the selection of the weight matrix? If it is sensitive, are there any recommendations for selecting the weight matrix based on data characteristics? When bridging to the experimental data, has the method been tested on MSI datasets (including synthetic ones) with available complementary ground-truth labeling to help evaluate the extent to which identified clusters map to known differences? It is mentioned that peaks that were present in less than 20% of the tissue were removed to focus on more common ions. To place this 20% cutoff in context, what was the coverage area of the clusters identified? It seems possible that this step may omit significant portions of tissue heterogeneity. For future applications of this workflow, how should this cutoff threshold be selected? Overall, how robust are the results/workflow recommendations to the choices of distance metric and clustering index?
Answer:
a)MSI data is a type of spatial data where nearby observations are highly correlated with each other. In our study, we had used the spatial autocorrelation function to generate similar spatially autocorrelated synthetic data. In additional File 3, a spatial correlogram plot from our synthetic data is presented, which is quite comparable with the spatial correlogram of drug molecular ions from different tumor MSI data shown in Additional File 5.
b)Yes, the results from spatial methods depend upon the selection of the spatial weight matrix. We performed a small simulation study with MSI data. And, we included our conclusion and recommendation in the discussion section, page 17.
c)In our study, we don't have ground-truth labeling. Therefore it was not possible to completely validate the identify clusters. The validation of identified clusters was performed with H&E stained images shown in Additional File 5 where the necrosis tissue present in the optical image shows similarity with clusters in the A2780-1A9 and HCT116 data.
d)In our study, we discarded peaks with less than 20% coverage area, assuming that they represent the noisy peaks and could influence our clustering algorithm. The decision of discarding those peaks was based on multiple trials. For the removal of peaks, we tried a threshold of 10%, 20%, and 30%. With a threshold of 10%, we missed some noisy peaks and with 30% we excluded a few extra peaks in our data. Therefore, a threshold of 20% seems a reasonable choice. Moreover, this step of peak

Powered by Editorial Manager® and ProduXion Manager® from Aries Systems Corporation

removal was performed on each tumor MSI data separately. Therefore, unless particular peaks were present as noise in all datasets, we do not expect them to be completely removed from our input data for the clustering.

The range of different clusters size derived across all tumor models is shown in Table 1 below. In our MSI data, we have identified the clusters of size smaller than 20% of total tumor tissue area which means after removing a fraction of peaks (which we assume noise-related) we are still able to find spatially relevant smaller clusters.

Table 1. Range of different clusters size in two tumor MSI data.
Cluster 1Cluster 2Cluster 3Cluster 4Cluster 5
A2780Minimum size21262440224153
Maximum size34292959169112703078
HCT116Minimum size337734558208186
Maximum size83316741297682605

Question 2.3 Given the dose of the drug administered, how much exposure within the tumor is expected based on pharmacology, and how might this affect the output? It would also help to provide more explanation of Figure S1 in Additional File 7. In it, the concentration (units undefined) of the drug in each cluster appears to be very similar, across both cell lines and treatment arms; however, the comparison between the clusters and the LISA maps appears to suggest differently. Also, interpretation of the LISA maps of drug exposure in Figure 3 and the data in Table 1 in terms of histology is briefly in the Discussion, but it would be helpful to expand on this.
Answer: For drug MSI data generation, the solid tumors were explanted from mice 6 hours after drug treatment. Therefore we do not expect dramatic changes in tissue architecture due to the drug compound. Moreover, all tumor models have received drug treatment, so if any metabolic change occurs, we expect it to be consistent across all tumor MSI data.
Additional File 7, Figure 1, shows the average amount of drug in each cluster-type under two treatment conditions. The drug average value in each cluster is further normalized with the sum of the average drug in a particular treatment condition. Note, this second step is performed simply to show the drug concentration in the range of 0-1. Apart from that the average drug concentration profile from steps 1 and 2 is similar.

References
1.Cesca, M.; Morosi, L.; Berndt, A.; Fuso Nerini, I.; Frapolli, R.; Richter, P.; Decio, A.; Dirsch, O.; Micotti, E.; Giordano, S.; et al. Bevacizumab-Induced Inhibition of Angiogenesis Promotes a More Homogeneous Intratumoral Distribution of Paclitaxel, Improving the Antitumor Response. Mol. Cancer Ther. 2016, 15 (1), 125–135. https://doi.org/10.1158/1535-7163.MCT-15-0063.

Additional Information:

**Question**	**Response**
Are you submitting this manuscript to a special series or article collection?	No
**Experimental design and statistics**<br><br>Full details of the experimental design and statistical methods used should be given in the Methods section, as detailed in our Minimum Standards Reporting Checklist. Information essential to interpreting the data presented should be made available in the figure legends.	Yes

Powered by Editorial Manager® and ProduXion Manager® from Aries Systems Corporation

Have you included all the information requested in your manuscript?	
**Resources**<br><br>A description of all resources used, including antibodies, cell lines, animals and software tools, with enough information to allow them to be uniquely identified, should be included in the Methods section. Authors are strongly encouraged to cite Research Resource Identifiers (RRIDs) for antibodies, model organisms and tools, where possible.<br><br>Have you included the information requested as detailed in our Minimum Standards Reporting Checklist?	Yes
**Availability of data and materials**<br><br>All datasets and code on which the conclusions of the paper rely must be either included in your submission or deposited in publicly available repositories (where available and ethically appropriate), referencing such data using a unique identifier in the references and in the “Availability of Data and Materials” section of your manuscript.<br><br>Have you have met the above requirement as detailed in our Minimum Standards Reporting Checklist?	Yes

Manuscript

Click here to access/download;Manuscript;Manuscript withoutTrackChanges.docx

A methodological approach to correlate tumor heterogeneity with drug distribution profile in mass spectrometry imaging (MSI) data

Mridula Prasad[1,2], Geert Postma[1*], Pietro Franceschi[2], Lavinia Morosi[3], Silvia Giordano[4], Francesca Falcetta[3], Raffaella Giavazzi[3], Enrico Davoli[4], Lutgarde M.C. Buydens[1], Jeroen Jansen[1]

[1]IMM/ Analytical Chemistry, Radboud University, Heyendaalseweg, 6525 AJ Nijmegen, Netherlands.

[2]Unit of Computational Biology, Research and Innovation Center, Fondazione Edmund Mach, 38010, San Michele all' Adige, Italy.

[3]Department of Oncology, Istituto di Ricerche Farmacologiche Mario Negri IRCCS, Via La Masa, 19-20156 Milan Italy.

[4]Mass Spectrometry Laboratory, Istituto di Ricerche Farmacologiche Mario Negri IRCCS, Via La Masa, 19-20156 Milan Italy,

ABSTRACT: Mass spectrometry imaging (MSI) has become a valuable tool in drug imaging because of its ability to provide a simultaneous spatial distribution of the drug and several other molecular ions present in the biological sample. An important application is the evaluation of homogeneity/heterogeneity of drug distribution in solid tumors. Solid tumors are known to be made up of different tissue subpopulations and their heterogeneity is supposed to have a direct and/or indirect influence on drug distribution. Hence, for further enhancement of penetration therapy performance, it is important to link a characterization of the tumor microenvironment with drug homogeneity. In this study, untargeted MSI data were used to understand the spatial heterogeneity within solid tumors, assessing its impact on the drug (paclitaxel) distribution. The proposed approach was applied on MSI datasets already analyzed, focusing on tumor drug distribution. Untargeted MSI datasets were collected on different tumor xenograft

models (ovarian and colon cancer cell lines) pre-treated or not with anti-angiogenesis compound (bevacizumab). Our main data analysis steps involved: a) pre-processing of MSI data to make all biological samples directly comparable, b) unsupervised data clustering to find different tissue subtypes, c) quantification of drug heterogeneity using local indicators of spatial association (LISA) map and d) selection of important ion signals from identified clusters of interest using the spatial -aware statistical tools. Our clustering results show variation in tumor subpopulations and less spatial heterogeneity in the MSI data collected on samples treated with the anti-angiogenesis compound consistently with our previous data. The local spatial structures identified in drug ion LISA maps show a correlation with clusters identified using a clustering method. Using the right spatial method, we were able to reduce the number of false-positive ions selected and identified the one that shows relevant spatial patterns in different tissue subtypes. Finally, our overall study shows that there is a direct association in drug homogeneity and spatial arrangement of different tissue subtypes in a solid tumor.

Introduction

Heterogeneity of the tumor microenvironment directly affects drug penetration reducing the therapeutic efficacy and contributing to the development of resistance[1,2]. For the above-mentioned reasons, knowledge about tumor spatial heterogeneity is then of paramount importance to optimize therapeutic outcomes[3]. In order to understand the relation between drug distribution and tissue homogeneity/heterogeneity, an imaging technique able to investigate both phenomena simultaneously would be highly desirable[4].

Mass spectrometry imaging (MSI) is a molecular imaging technique that provides simultaneous information about spatial localization of drugs and other small molecules present in the biological sample[5,6]. It is a valuable technique to characterize tumor tissue subtypes[7–9]and it is also applied to map the distribution of drugs within the tissue[10–13]. Untargeted MSI datasets provide unprecedented opportunities to understand the drug distribution in association with tissue heterogeneity. Several computational algorithms have been implemented on MSI data for the identification of tumor tissue

areas[7,8,14]and important molecular ions selection[8,15,16]. As far as our knowledge there is no work done that combines the tumor heterogeneity with a drug distribution profile. In a recent paper, we have proposed a new objective index (DHI)[17] to measure drug homogeneity in different MSI datasets and we aim at extending this work proposing a framework that can be used to investigate the relation between drug homogeneity and the observed tumor heterogeneity. The proposed approach encompasses the following steps.

1. Identification of the different tissue sub-populations from an untargeted multi-sample MSI dataset (segmentation).
2. Quantitative analysis of the spatial arrangement of the tissue subtypes across different samples.
3. Extraction of discrete drug distribution maps.
4. Matching of the segmented MSI dataset with the drug distribution maps.
5. Selection of ion signals that can be used to differentiate specific tissue areas.

Since the accessibility of a drug to a particular spatial location is expected to be linked to the metabolic and histological characteristics of the underlying tissues, multivariate unsupervised clustering was used to group the pixels into a limited number of groups which represent the different tissue subtypes[8,9,14,18–20] (Step 1). This step was performed after removing the drug-related peaks to avoid their influence in groups formation. The quantitative assessment of the spatial arrangement of the different clusters (Step 2) was performed by using a modified version of our DHI[17] index, which was optimized to characterize the spatial arrangement of the pixels on the clustered images. The *local indicators of spatial association (LISA)*[21,22] method was instead applied to create discrete drug distribution maps (Step 3). The association between LISA maps and identified segments was derived using Cramer's[23] V method (Step 4). The identification of important ion signals able to differentiate the tissue subtypes was finally performed by using a spatial-aware statistical method that corrects for spatial autocorrelation[24] (Step 5). Since the application of these models in MSI is still uncommon, the optimal spatial model for biomarker selection was tested on a synthetic autocorrelated spatial dataset.

In our study, the identification of tissue subpopulations was performed using K-means clustering with correlation distance. This non-spatial clustering method has already been used in many other MSI

research[25–27] and was able to efficiently extract relevant structures. The spatially relevant clusters in drug ion image were detected based on Moran's scatter plot or LISA map. LISA map is a commonly used method in spatial statistics where pixels are grouped into different clusters (zones) based on their similar or different behavior with neighboring pixels[28,29]. The potential of the proposed approach was tested on the same datasets that we used in our previous work[17,30] which consisted of the MSI untargeted analysis of a set of different tumor xenograft models from ovarian (A2780) and colon (HCT116) cancer cell lines. The study was designed to assess the effect of an anti-angiogenesis compound (bevacizumab) on the drug (paclitaxel) distribution[30]. For this purpose, half of the animals were pretreated twice (5 and 1 days before excision) with bevacizumab, before being administered with paclitaxel a few hours (six) before excision. Even if it is impossible to exclude that the anticancer drug could be responsible for the observed spatial heterogeneity in metabolic profile[31], any relevant difference between the two treatment groups is likely to be associated with the direct or indirect effect of bevacizumab.

EXPERIMENTAL SECTION

MSI datasets

MSI data were derived from the tumor-bearing mice, treated with paclitaxel (60 mg/kg) alone or in combination with bevacizumab (two intraperitoneal injections at 150 μg per mouse). For mice experiment, IRFMN adheres to the principles set out in the following laws, regulations, and policies governing the care and use of laboratory animals: Italian Governing Law (D.lgs 26/2014; Authorization n.19/2008-A issued March 6, 2008 by Ministry of Health); Mario Negri Institutional Regulations and Policies providing internal authorization for persons conducting animal experiments (Quality Management System Certificate–UNI EN ISO 9001:2008 –Reg. N° 6121); the NIH Guide for the Care and Use of Laboratory Animals (2011 edition) and EU directives and guidelines (EEC Council Directive 2010/63/UE).

The complete details of the experiment experiments described here.[12,17,30]. Briefly, tumors were collected, frozen in liquid nitrogen and prepared for MSI analysis. A MALDI 4800 TOF-TOF (AB

SCIEX, Old Connecticut Path, Framingham, MA) was used. And, mass spectra were recorded in full-scan-profile mode over a limited mass range (*m*/*z* 199–500). Images of tissue sections were acquired using the 4800 Imaging Tool software with an imaging raster of 100×100 μm (pixel dimension of ca. 0.01 mm^2). The obtained dataset consisted of 131 349 (A2780) and 59 652 (HCT116) raw spectra from the tissue areas of all tumor models.

MSI data pre-processing

The initial data files in Analyze 7.5 format were opened in R[32] free software version 3.4.3 using the MALDIquant[33] package. Each MSI datafile contains mass spectra collected from both tumor tissue and a glass slide. To avoid any bias in pre-processing and data analysis steps, the mass spectra belonging to tumor tissue only were used. The identification of tumor tissue was performed by constructing a mask of the ion signal detected in the m/z = 281.1-281.44 range. This ion was confirmed to be a reliable tissue marker by visually comparing all MS images with their optical counterparts. To correct for possible spectral misalignments across the different datasets, an adaptive binning approach was applied. To do this, first, a reference spectrum was created, which is a single maximum intensity spectrum of all the spectra. The main reasoning behind it is to acquire knowledge about the location and shape of all the ion peaks in our data and then optimize the bin size accordingly. Smoothing of the reference spectrum was performed by applying discrete wavelet transformation (Daubechies least asymmetric 8-tap filter with hard shrinkage) using a msDenoiseWavelet function from msProcess[34] R package. Peaks in the reference spectrum were identified by local maxima search above a certain threshold[17]. Initial bins were created based on those identified peaks. The bin size was further optimized on the bases of instrument characteristics merging together peaks showing a difference of less than 0.05m/z. To avoid that peaks are missed in low-intensity spectra, an extra bin of size 0.5 was added in the presence of a large gap between two identified peaks (especially towards the end of the spectra). The complete workflow of bin creation and maximum intensity spectra from two tumor MSI data are shown in Additional file 1.

After bin identification, each MSI data file was reopened and peak picking was performed using an approach similar to the one applied to the reference spectrum and the identified peaks were assigned to their corresponding bins. To focus only on the more common ions, peaks that were present in less than 20% of the tissue area were removed. Peaks only present in a single tumor model were rejected. To remove spatial noise, median filtering with a window size of 3x3 pixels was performed on each extracted ion image. We also performed edge correction in the individual dataset, as marginal pixels have enormously high intensity due to the surface difference between tissue and glass slide[35]. image. To make the spectra collected on the different pixels comparable median normalization was performed. Generalized log transformation was performed as a variance stabilization step using LMGene[36] in R. A plausible batch effect between the slices of certain mouse models was removed using the removeBatchEffect function from R limma[37] package. Ion peaks with correlation with the drug compound (paclitaxel) (>0.9) were removed before cluster analysis. The overall dimension of the final data matrix belonging to A2780 and HCT116 is equal to 131 349 x 173 and 59 652 x 155, respectively.

MSI data analysis

Unsupervised data clustering

Segmentation of MSI data collected on the complete set of sections from a specific cell-line was performed by unsupervised bisect k-means clustering[38] using correlation as a similarity measure. The individual clusters were allowed to split further until the largest cluster contains 40% of the pixels included in the initial data matrix. The number of clusters at each step was selected based on the calinhara internal validity index[39]. The clustering and validation were implemented using R-packages amap[40] and fpc[41], respectively. The outcomes of segmentation for the different sample groups (cell lines and bevacizumab treatment) were analyzed based on the following parameters: a) size and b) homogeneity of the individual clusters. The homogeneity of the individual clusters was assessed by using a slightly modified version of the drug homogeneity index (DHI) (see Additional File 2). Higher cluster

homogeneity means the more continuous/homogeneous distribution of a particular tissue-type. All these parameter values were normalized by the total number of pixels from the tumor models of each treatment condition. Statistical significance of pixel and homogeneity ratio under two treatment conditions was calculated using linear mixed models with the nlme[42] R-package.

Quantification assessment of the drug distribution

Spatial quantification of the drug heterogeneity was performed using the Moran's I scatter plot[24], also known as the local indicators of spatial autocorrelation (LISA) map (see Additional File 2). This spatial-aware method was selected because is expected to yield more robust results in the presence of the spatially autocorrelated drug signal.

To create a Moran's I scatter plot and/or LISA map required inputs are: original variable, spatially lagged variable and spatial weight matrix. The original variable in our case it is the ion intensity map of the drug peak. The spatially lagged variable is constructed by multiplying the autoscaled version of the original variable with the help of the spatial weight matrix[24,43]. This weight matrix stores the connections between nearby observations (e.g. in a binary weight matrix the observations which lie within a certain range of autocorrelation receive a value of one else zero). The optimal range of autocorrelation can be decided based on spatial correlogram. A spatial correlogram[24] is a 2D plot where the spatial autocorrelation index (Moran's I) is plotted as a function of lag distance where a positive value indicates the presence of autocorrelation within a certain distance range. To create a LISA map of a drug ion image the following steps were performed:

- the optimal spatial weight matrix was created on the bases of the spatial correlogram plot (Figure 1b).
- the original variable was converted into its spatially lagged version (Figure 1c).
- Moran's I scatter plot was created by regressing the original variable against its spatially lagged version where pixels are grouped into four different zones usually called high-high, low-low, high-low and low-high (Figure 1d).
- Finally, a LISA map was constructed which is a two-dimensional image where pixels are labeled according to their class in Moran's I scatter plot (Figure 1e).

Figure 1. A schematic workflow of drug LISA map creation. a) Original drug ion image. b) Spatial correlogram of the drug-ion image where Moran's I values (Ac1) are plotted against the lag distance (dists). c) spatially lagged image of the drug-ion. d) Moran's I scatter plot where the drug signal and its spatially lagged version are regressed against each other. e) LISA clustered map of the drug ion where pixels falling in the same quadrant of the Moran scatter plot are grouped.

In a LISA map or Moran's I scatter plot, the high-high zone contains pixels that have a high intensity or above-average value and surrounded by a similar type of high-intensity pixels. The low-low zone contains pixels that have a lower intensity or below-average value and surrounded by a similar type of low-intensity pixels. The high-low zone contains pixels that have above-average value for themselves but surrounded by neighbors with below-average value. The reverse rule applies to the pixels fall in the low-high zone. Note, in a LISA map pixels falling in high-high and low-low zones show positive spatial autocorrelation and are spatially smooth. Therefore, a single zone of a LISA map may contain multiple clusters with approximately similar profiles.

Association between clustered image and drug LISA map

The obtained drug LISA maps were analyzed for their association with the unsupervised clusters obtained using unsupervised clustering. A quantitative analysis was performed to understand which cluster subtypes overlap most with which zone of the LISA map, for which the fraction of pixels in different zones and clusters in each tumor model was calculated. Further, the strength of association between those two vector classes (LISA map zones and unsupervised clusters) was estimated using Cramer's V[24] method. Cramer's V is a statistical measure similar to the Pearson correlation to find the correlation between two nominal variables and returns a correlation value within the range of 0-1.

Representative ion signals selection from the identified clusters

The method used to select representative ion signals from the identified clusters in the MSI data was first validated on synthetic spatially autocorrelated data. Two spatial approaches (spatial error model (SE), and spatial lag model (SL)) were compared with a standard non-spatial approach (ordinary least square (OLS)). Both spatial models[24,44] are modified versions of an OLS model and include spatial autocorrelation in a different component of the OLS model. The comparison of the performance of the above mentioned statistical methods with complete description of the synthetic data generation process is given in Additional File 3.

All spatial models were fitted with the spdep[24] R package. Similar to what was done in the case of the LISA map, the right threshold for the spatial weight matrix was decided based on the spatial correlogram.

In MSI data, the selection of ion signals from identified clusters was performed using the method which gives the best performance on our synthetic data. In order to do that, the outcomes of the original clustering were converted into a set of two-class images where each cluster is, in turn, compared with all the others. As the variables selection using the spatial method is computationally intensive, we only used a few tumor models in which the cluster of interest was present. Thus, per cluster five different tumor slices were selected, i.e. MSI data from 4 - 5 different tumor models. If a particular ion was found to be important in all five datasets, then it was considered as a significant ion signal for the respective cluster. The important ions were selected on the bases of the model p-values corrected for multiple testing by using the procedure of Benjamini & Hochberg[45].

Results

Unsupervised clustering of MSI data

a) A2780 cell-line based MSI data

The clustering method identified five unique clusters in the combined set of A2780 xenograft models (Figure 2 left). The majority of the replicates possess those five clusters in different ratios where clusters 1 and 2 were predominant in all tumor models in both treatment conditions. The relative contribution of

cluster 3 is reduced and cluster 4 is enhanced in the presence of bevacizumab (Figure 2, top right). Cluster 3 showed a high overlap with the necrosis area[12] and noticed to be present in a relatively higher fraction among the tumor models not pretreated with bevacizumab. The small fraction of cluster 5 is present in all tumor models.

Similar to the number of pixels, the homogeneity of clusters (parameter b) for the individual tumors was calculated using the modified version of our DHI and it is shown in Figure 2, bottom right. The figure highlights the clear difference in clusters homogeneity under the two treatment conditions, especially for cluster 2. The homogeneity of cluster 2 in the presence of bevacizumab treatment is much higher than the homogeneity of any other cluster in two treatment conditions. Without bevacizumab treatment also tumor models show high homogeneity for cluster 2 followed by cluster 3.

Figure 2: Cluster analysis of A2780 tumor MSI data generated in the presence and absence of bevacizumab treatment. Left: Representation of clusters detected by the k-means method. Right: Ratio of a) pixels and b) homogeneity calculated from individual clusters under two treatment conditions. The red horizontal line is the global mean value of pixel and homogeneity ratio. Here, Pixel ratio = Number of pixels in individual clusters/Total number of pixels from all tumor MSI data under particular treatment conditions. Homogeneity ratio =Size-zone of individual clusters for a given tumor model/Total number of pixels in that particular tumor model. With Beva = pretreated with bevacizumab and Without Beva = without bevacizumab pretreatment.

b) HCT116 cell-line based MSI data

Five clusters were identified in the HCT116 tumor cell line MSI data (see Additional File 4). Similar to the A2780-1A9 tumor MSI data, there was not a large observed difference in clusters population under the two treatment conditions. Cluster 2 and 3 were predominantly present in all tumor models irrespective of the treatment conditions (see Additional File 4 Figure S-1 top right). Cluster 1 was observed in a moderate amount and very small fractions of clusters 4 and 5 were present in all tumor models. The

homogeneity assessment of the individual clusters in the two treatment conditions shows that cluster 3 has high homogeneity in the case of bevacizumab treatment. In the absence of bevacizumab treatment, clusters 2 and 3 show more homogeneity (see Additional File 4 Figure S-1 bottom right).

The statistical analysis of pixel and homogeneity ratio values was performed using a linear mixed model approach where range and p-value from both tumor models are given in Additional File 4 Table S-1. For the A2780-1A9 MSI data, the pixel ratios are not significantly different in the two treatment conditions. The homogeneity value of cluster 2 is close to significant which is derived with parameter $Nu = 5$ in our homogeneity formula. In HCT116 MSI data, the number of pixels in cluster 1 and homogeneity ratio for cluster 2 is statistically significant in two treatment conditions.

Association between clustered image and drug LISA map

The spatially distinct regions based on observed drug distribution profiles were identified using LISA maps in all tumor models. The LISA map was created using the spatial weight matrix with an autocorrelation value equal to five since in spatial correlograms high positive spatial autocorrelation was observed within this range (see Additional File 5 S-1).

A visual comparison of the clustered images and drug LISA maps confirms the link between the drug distribution profile and the underlying clusters (Figure 3). For example, homogeneously high drug distribution areas (high-high (HH) zone in the LISA map) are mostly associated to cluster type 1 and 2, while, homogeneously low drug distribution areas (low-low (LL) zone in the LISA map) correspond to cluster type 3 and 5 (Table 1). The observed association between cluster types and the different zones of the LISA map is irrespective of treatment condition across all tumor models (Figure 4).

Similar observations were made from HCT116 tumor MSI data (see Additional File 5). In HCT116 MSI data, clusters 1,2 and 3 show clear overlapping with spatially homogeneous zones of the LISA maps. The cluster 1 and 3 overlapped with high drug concentration areas in the tissue and cluster 2 with low drug concentration areas (see Additional File 5). The association between cluster 2 and low drug concentration

areas in the LISA maps is more clear for HCT116 data than for A2780-1A9 data (Additional File 5 Table S-1).

The statistical correlation between the clustered image and drug LISA map was calculated using Cramer's V method. A very small fraction of pixels falls within high-low and low-high zones of the LISA map. Therefore, the Cramer's V is calculated between HH, LL zones of LISA map with unsupervised clustered classes. For both tumor MSI data, the Cramer's value across all tumor models was found to be within the range of 0.5 -0.8 (see Additional File 5 Table S-1) that confirmed the dependency of the drug on different tumor tissue areas.

Figure 3: Individual clustered image (first column), LISA map (second column) and their combination are shown for few tumor models from A2780-1A9 MSI data. The clusters found in high-high (HH), low-low (LL), high-low (HL), and low-high (LH) zones of LISA map are highlighted. In LISA map, HH, LL, HL, and LH are zones identified in Moran's I scatter plot.

Table 1: The percentages of pixels belonging to different cluster classes falling into HH, LL, HL and LH zones of the LISA map for tumor MSI data shown in Figure 3.

		Cluster 1	Cluster 2	Cluster 3	Cluster 4	Cluster 5	Cramer's V*
Image 1	HH	**8.28%**	**23.5%**	0.58%	5.75%	3.5%	0.501
	LL	4.48%	4.92%	**7.9%**	5.26%	3.46%	
	HL	3.36%	7.85%	0.87%	2.68%	1.22%	
	LH	2.87%	5.46%	2.44%	2.58%	2.88%	
Image 2	HH	**18.15%**	**13.34%**	0.31%	2.98%	1.94%	0.643

	LL	0.86%	9.39%	0.8%	7.49%	**15.93%**	
	HL	2.83%	8.5%	0.09%	1.6%	3.76%	
	LH	2.89%	3.57%	0.43%	2.1%	3.01%	
Image 3	HH	**14.69%**	**13.55%**	0.05%	5.77%	2.19%	0.463
	LL	4.2%	11.57%	0.66%	9.22%	**14.78%**	
	HL	4.02%	7.19%	0.036%	1.56%	1.69%	
	LH	1.38%	2.65%	0.29%	1.79%	2.69%	
Image 4	HH	**21.55%**	**17.09%**	0.094%	1.95%	3.89%	0.69
	LL	3.67%	1.91%	2.29%	6.2%	**18.62%**	
	HL	3.12%	2.20%	0.21%	1.93%	3.16%	
	LH	3.30%	3.44%	0.67%	1.4%	3.29%	
Image 5	HH	**16.41%**	**20.53%**	2.49%	4.2%	2.95%	0.601
	LL	2.20%	3.10%	**11.89%**	2.33%	2.15%	
	HL	3.51%	4.43%	2.89%	1.43%	1.79%	
	LH	3.36%	5.99%	3.13%	2.74%	2.44%	
Image 6	HH	**15.04%**	**23.57%**	1.53%	0.99%	3.23%	0.62
	LL	4.04%	4.12%	**13.44%**	2.05%	**4.37%**	
	HL	3.71%	6.59%	2.12%	1.19%	2.23%	
	LH	2.85%	3.64%	2.83%	0.98%	1.44%	

*Cramer's Correlation is calculated between HH, LL zones of LISA map and unsupervised clusters.

Figure 4: Quantitative analysis to find the association between drug LISA maps and identified clusters from complete A2780-1A9 tumor MSI data. Here, each subplot highlights the fraction of pixels present in different zones of the LISA map under two treatment conditions. The red horizontal line in each subplot is a global mean value for pixel ratio for that particular zone. With Beva: pre-treated with bevacizumab and without Beva: without pre-treatment with bevacizumab

Ion signals selection from the identified clusters

For variables selection, the performances of spatial (SE and SL) and non-spatial (OLS) methods were tested on synthetic spatially autocorrelated data. The SL method completely outperformed the other two methods (see Additional File 3); therefore, used for m/z values selection from unsupervised clusters identified in two MSI cancer datasets (Table 2). Note, cluster 4 identified in A2780-1A9 tumor data was present in a single tumor model and cluster 4 and 5 for HCT116 was present in very small fraction and did not follow any proper spatial structure , therefore those clusters were excluded from the ions selection step. The list of important ions is given in Additional file 9. In the A2780 cancer data, cluster 2 had 28 ions showing a significant difference. In particular, the ion at m/z=335.41 had high intensity in all tumor models. The ion image of this particular ion showed a homogeneous distribution in the cluster (see Additional File 6 Figure S-1 top row). A larger number of significantly different ions was identified for cluster 3, mostly with low signal intensity.

For the HCT116 MSI data, a large number of significant ions was identified in cluster 2 and 3. In cluster 3, the majority of ions had a high signal or positive regression coefficients in the spatial model, with the ion at m/z =281.315 showing the highest value. In cluster 2, ions with both high and low signals were present in equal portions (see Additional File 6 Figure S-1 bottom row).

Table 2: The number of ion signals selected from different clusters in two tumor MSI data

Cluster type	A2780	HCT116
1	26	22
2	28	83
3	91	70

5	35

Discussion

Several studies have shown that the tissue spatial heterogeneity within a solid tumor impacts on the drug distribution[2,10,12,30,46,47]. Different tissue characteristics and tumor microenvironment affect the drug distribution which means that the concentration of a drug at a given spatial location could be related to the tissue composition at that point. It is also true that the presence of the drug can induce a modification of the tumor structure[31], so in general, it is impossible to disentangle the two phenomena. In our case, the situation was fortunate since a part of the tumor-bearing mice had received bevacizumab treatment before drug (paclitaxel) treatment. Therefore, we assume that the observed differences in the spatial organization of tissue areas characterized by similar metabolic fingerprints can be interpreted as a direct or indirect effect of bevacizumab treatment which was given twice before drug injection. This was also suggested by our previous study showing an increase in drug homogeneity in samples treated with bevacizumab[30]. The main goal of this research was to show that computational methods can be used to explore and quantify spatial heterogeneity within tumors and link the observed homo-/heterogeneous drug distribution to the alteration in microenvironment due to applied therapeutic strategy. To achieve these objectives, our data analysis involved a combination of methods from different research streams. First, the clustering of combined MSI data was performed using K-means with correlation distance and then those clusters were linked with drug distribution patterns obtained using the LISA method. As stated in the introduction, K-means clustering is efficiently being used in several studies performed on MSI data for the selection of relevant clusters[25–27]. Moreover, in another study, the authors also tested this method and compared it with several spatial methods, i.e., using simulated and real data and came to the same conclusion (unpublished observation). In the spatial data analysis field, a LISA map is a commonly known technique that identifies the spatially relevant clusters in a single two-dimensional

image[21,22,43]. The LISA map provides an automated way to find spatially homogeneous clusters that are difficult to generate using a simple thresholding approach (see Additional File 8). Simple binary images were created using different threshold values. The images constructed with a threshold value of four show some resemblance with the LISA map (see Additional File 8). The binary images are not fully able to mimic the drug distribution profile. Moreover, the selection of the right threshold value from the drug ion image histogram is not very straightforward. In contrast, the LISA map is able to highlight the observed high and low intensity spatially homogeneous areas in drug ion images efficiently where for spatial weight matrix can be selected based on spatial correlogram.

The clustering of MSI data was able to identify metabolic separated regions which cannot be observed in H&E stained tissue images[25]. This is clear if one compares the images shown in Figure 5. The segmented image shows tissue subtypes in addition to the one which can be associated to the necrotic and fibrous regions on H&E stained image. The effect of the treatment with bevacizumab was visible in the segmented images. In particular, the antiangiogenic compound was increasing homogeneity (both in the pattern of clusters and in the drug) even if no anti-angiogenesis treatment specific cluster was identified (Figure 2 and Additional File 4). Interestingly, the average drug concentration in the individual clusters under the two treatment conditions is approximately equal (see Additional File 7). This confirmed the results of our previous publication[17] and our homogeneity assessment of clustered images (Figure 2 bottom right) that the tumor tissues from bevacizumab treatment are more homogeneous.

Quantitative analysis of the LISA maps shows the dependency of the observed drug distribution profiles and the underlying tissue-types (Figure 4 and Additional File 5). In A2780-1A9 tumors, clusters 1 and 2 have a high affinity for the drug and while clusters 3 and 5 show a lower than the average drug concentration (Figure 4 and Table 1).

For HCT116 tumors, clusters 1 and 3 showed some overlap with the high-high zone, and cluster 2 with the low-low zone (see Additional File 5). Remarkably, the clusters associated with the HH and LL drug

distribution regions are always the same, regardless of the pretreatment with bevacizumab, which is instead affecting the arrangement of the tissue subpopulations.

The dependency of spatial methods on the spatial weight matrix

Two commonly known spatial methods (LISA map and spatial lagged regression) were used in our study to find the spatially homogeneous clusters and spatially relevant ions from different clusters, respectively. Both spatial methods required the spatial weight matrix as an input. Therefore, the dependency of results on the spatial weight matrix was tested (see Additional File 10 and 11). The variables selection was performed using the SL method for a range of autocorrelation or lag distance values (1-15) in the spatial weight matrix in tumor MSI data (see Additional File 10). The SL method selects mostly the same molecular ions at different lag distance values. All variables selected have positive Moran's I value at the above-mentioned autocorrelation range. Therefore, based on our analysis of MSI data we had not noticed the dependency of the SL method on the spatial weight matrix. A similar observation was made when this analysis was performed on synthetic data (see Additional File 3). In the case of our synthetic data, the accuracy of SL starts decreasing after a lag distance of 6 since some false positive selection was observed, but sensitivity remains constant as the true variables were all selected. However, in the case of real MSI data, this type of plot is not feasible. Therefore we looked at Moran's spatial autocorrelation value of all selected variables (see Additional File 10). After the lag distance of 5 the Moran's I value of the selected variables starts to approach zero. Therefore, even though in our analysis the list of molecular ions was mostly consistent we will not recommend going beyond the lag distance of 5, as at such large autocorrelation range we may start including some noisy variables in our list.

A similar approach is used to test the dependency of a LISA map on the spatial weight matrix. The LISA map of the drug ion image for a particular tumor tissue was constructed with different spatial weight matrices (see Additional File 10). The LISA map created with the lag distance of one contains large homogenous areas (LL and HH). On the increasing the lag distance, the pixels from different areas start

mixing, i.e. the size of HL and LH regions gradually start increasing and the LISA map becomes less reliable. Therefore similar to the spatial regression method, the spatial weight matrix with a maximum lag distance of 5 is preferable for a LISA map.

In summary, in this paper, we provide a computational approach to understand the problem of drug homogeneity in association with tumor heterogeneity which validates the few conclusions made in previous studies. We provide a complete workflow of data pre-processing of MSI data, the association between the drug and identified clusters, and the selection of molecular from identified clusters. In our paper, we have used different methods to get different pieces of information. The dependency of spatial methods on the spatial weight matrix is discussed above. Apart from that, if the user wants to use our approach on their MSI data, they also need to consider the parameters used in our studies, such as peak removal with less than 20% coverage area and the clustering index. In our case, before setting a 20% threshold we tried a 10 -30% threshold to be sure if the majority of noisy peaks have been removed and no other important peaks. A similar task was performed during the clustering of MSI data. The number of clusters was selected using the internal clustering index method but the stopping criteria in bisect clustering (40%) was set according to our data dimension and the number of expected clusters in our MSI data. If the MSI data contain very small spatial structures than this threshold needs to be reduced. In general, we believe that our paper completes the pipeline for the analysis of untargeted drug MSI data and will be useful for the groups working with a similar problem. A R pipeline includes all the methods from our papers is online available (RRID: SCR 018962, bio.tools: corrdrugtumormsi)

Figure 5: Comparison of tumor tissue optical images (rightcolumn) with its clustered image (left column) from two tumor MSI data (A2780-1A9, HCT116). The black and red dots in the H&E stained images

represent the necrotic and fibrotic area, respectively. The optical images are adapted from a scientific journal[10] published under CC BY license[48].

Conclusions

In cancer research, one of the causes of drug therapy failure is tumor drug resistance often induced by scare drug penetration. This phenomenon is supposed to be linked to the presence of diverse tumor microenvironments which are difficult to identify with established histological techniques. In this work, we show that a molecular imaging technique like MSI, coupled with advanced data analysis strategies, offers a great opportunity to investigate the link between drug distribution and tissue heterogeneity. Our approach allowed to simultaneously investigate tissue histology and drug distribution and it was capable of detecting the effects on the tumor heterogeneity induced by a specific intervention (treatment with bevacizumab). We hope that the unsupervised approach proposed here will help oncologists to quantitatively evaluate the efficacy of therapeutic strategies.

ASSOCIATED CONTENT

Availability of supporting data

The datasets and R-script used to generate the results of this article are available on the DNAS-KNAW repository (https://doi.org/10.17026/dans26h-kstf).

Additional files

Additional File 1: Figures show workflow of adaptive bins creation and maximum intensity spectrum from two tumor models.

Additional File 2: Description of the statistical methods used in this manuscript.

Additional File 3: Synthetic spatially autocorrelated data generation steps and comparison of spatial and non-spatial methods for variables selection on synthetic data.

Additional File 4: Cluster analysis of HCT116 MSI. Range and significance values for pixels and homogeneity ratio from different clusters under two treatment condition.

Additional File 5: Overlay of clustered image and LISA map and contingency table from HCT116 MSI data. And, the quantitative analysis of LISA maps for HCT116 tumor MSI data.

Additional File 6: MS images of selected molecular ions from different clusters from tumor MSI data.

Additional File 7: Plot of drug concentration in different clusters.

Additional File 8: Comparison of drug binary image created by selecting manual threshold value based on histogram and LISA method.

Additional file 9: Excel sheet contains selected important ion signals from identified clusters of our cancer MSI data.

Additional file 10: Sensitivity analysis for spatial weight matrix in spatial methods

Additional file 11:List of molecular ions selected for clustered image shown in Additional file 10

AUTHOR INFORMATION

Corresponding Author

* E-mail: chemometrics@science.ru.nl

Competing Interests

The authors declare no competing interests.

Author Contributions

MP analyzed MSI data and wrote first draft of the paper under supervision of GP, PF, JJ and LB. GP, PF, and JJ edit the manuscript. ED, LM, SG, FF and RG did MSI experiment and review the manuscript. All authors read and approved the final manuscript.

REFERENCES

(1) Minchinton, A. I.; Tannock, I. F. Drug Penetration in Solid Tumours. *Nat. Rev. Cancer* **2006**, *6* (8), 583–592. https://doi.org/10.1038/nrc1893.

(2) Trédan, O.; Galmarini, C. M.; Patel, K.; Tannock, I. F. Drug Resistance and the Solid Tumor Microenvironment. *J. Natl. Cancer Inst.* **2007**, *99* (19), 1441–1454. https://doi.org/10.1093/jnci/djm135.

(3) Zhao, B.; Hemann, M. T.; Lauffenburger, D. A. Intratumor Heterogeneity Alters Most Effective Drugs in Designed Combinations. *Proc. Natl. Acad. Sci. U. S. A.* **2014**, *111* (29), 10773–10778. https://doi.org/10.1073/pnas.1323934111.

(4) Heindl, A.; Nawaz, S.; Yuan, Y. Mapping Spatial Heterogeneity in the Tumor Microenvironment: A New Era for Digital Pathology. *Lab. Investig.* **2015**, *95* (4), 377–384. https://doi.org/10.1038/labinvest.2014.155.

(5) Wiseman, J. M.; Ifa, D. R.; Zhu, Y.; Kissinger, C. B.; Manicke, N. E.; Kissinger, P. T.; Cooks, R. G. Desorption Electrospray Ionization Mass Spectrometry: Imaging Drugs and Metabolites in Tissues. *Proc. Natl. Acad. Sci.* **2008**, *105* (47), 18120–18125. https://doi.org/10.1073/pnas.0801066105.

(6) Prideaux, B.; Stoeckli, M. Mass Spectrometry Imaging for Drug Distribution Studies. *J. Proteomics* **2012**, *75* (16), 4999–5013. https://doi.org/10.1016/j.jprot.2012.07.028.

(7) Jones, E. A.; van Remoortere, A.; van Zeijl, R. J. M.; Hogendoorn, P. C. W.; Bovée, J. V. M. G.; Deelder, A. M.; McDonnell, L. A. Multiple Statistical Analysis Techniques Corroborate Intratumor Heterogeneity in Imaging Mass Spectrometry Datasets of Myxofibrosarcoma. *PLoS One* **2011**, *6* (9), e24913. https://doi.org/10.1371/journal.pone.0024913.

(8) Abdelmoula, W. M.; Balluff, B.; Englert, S.; Dijkstra, J.; Reinders, M. J. T.; Walch, A.

Data-Driven Identification of Prognostic Tumor Subpopulations Using Spatially Mapped t-SNE of Mass Spectrometry Imaging Data. *PNAS* **2016**, *113* (43), 12244–12249. https://doi.org/10.1073/pnas.1510227113.
(9) Inglese, P.; McKenzie, J. S.; Mroz, A.; Kinross, J.; Veselkov, K.; Holmes, E.; Takats, Z.; Nicholson, J. K.; Glen, R. C. Deep Learning and 3D-DESI Imaging Reveal the Hidden Metabolic Heterogeneity of Cancer. *Chem. Sci.* **2017**, *8* (5), 3500–3511. https://doi.org/10.1039/c6sc03738k.
(10) Giordano, S.; Morosi, L.; Veglianese, P.; Licandro, S. A.; Frapolli, R.; Zucchetti, M.; Cappelletti, G.; Falciola, L.; Pifferi, V.; Visentin, S.; et al. 3D Mass Spectrometry Imaging Reveals a Very Heterogeneous Drug Distribution in Tumors. *Sci. Rep.* **2016**, *6* (1), 37027. https://doi.org/10.1038/srep37027.
(11) Thompson, C. G.; Bokhart, M. T.; Sykes, C.; Adamson, L.; Fedoriw, Y.; Luciw, P. A.; Muddiman, D. C.; Kashuba, A. D. M.; Rosen, E. P. Mass Spectrometry Imaging Reveals Heterogeneous Efavirenz Distribution within Putative HIV Reservoirs. *Antimicrob. Agents Chemother.* **2015**, *59* (5), 2944–2948. https://doi.org/10.1128/AAC.04952-14.
(12) Giordano, S.; Zucchetti, M.; Decio, A.; Cesca, M.; Fuso Nerini, I.; Maiezza, M.; Ferrari, M.; Licandro, S. A.; Frapolli, R.; Giavazzi, R.; et al. Heterogeneity of Paclitaxel Distribution in Different Tumor Models Assessed by MALDI Mass Spectrometry Imaging. *Sci. Rep.* **2016**, *6* (1), 39284. https://doi.org/10.1038/srep39284.
(13) Walch, A.; Rauser, S.; Deininger, S.-O.; Höfler, H. MALDI Imaging Mass Spectrometry for Direct Tissue Analysis: A New Frontier for Molecular Histology. *Histochem. Cell Biol.* **2008**, *130* (3), 421–434. https://doi.org/10.1007/s00418-008-0469-9.
(14) Balluff, B.; Frese, C. K.; Maier, S. K.; Schöne, C.; Kuster, B.; Schmitt, M.; Aubele, M.; Höfler, H.; Deelder, A. M.; Heck, A. J.; et al. De Novo Discovery of Phenotypic Intratumour Heterogeneity Using Imaging Mass Spectrometry. *J. Pathol.* **2015**, *235* (1), 3–13. https://doi.org/10.1002/path.4436.
(15) Cassese, A.; Ellis, S. R.; Ogrinc Potočnik, N.; Burgermeister, E.; Ebert, M.; Walch, A.; van den Maagdenberg, A. M. J. M.; McDonnell, L. A.; Heeren, R. M. A.; Balluff, B. Spatial Autocorrelation in Mass Spectrometry Imaging. *Anal. Chem.* **2016**, *88* (11), 5871–5878. https://doi.org/10.1021/acs.analchem.6b00672.
(16) Inglese, P.; McKenzie, J. S.; Mroz, A.; Kinross, J.; Veselkov, K.; Holmes, E.; Takats, Z.; Nicholson, J. K.; Glen, R. C. Deep Learning and 3D-DESI Imaging Reveal the Hidden Metabolic Heterogeneity of Cancer. *Chem. Sci.* **2017**, *8* (5), 3500–3511. https://doi.org/10.1039/c6sc03738k.
(17) Prasad, M.; Postma, G.; Morosi, L.; Giordano, S.; Giavazzi, R.; D'Incalci, M.; Falcetta, F.; Davoli, E.; Jansen, J.; Franceschi, P. Drug-Homogeneity Index in Mass-Spectrometry Imaging. *Anal. Chem.* **2018**, *90* (22), 13257–13264. https://doi.org/10.1021/acs.analchem.8b01870.
(18) Trede, D.; Schiffler, S.; Becker, M.; Wirtz, S.; Steinhorst, K.; Strehlow, J.; Aichler, M.; Kobarg, J. H.; Oetjen, J.; Dyatlov, A.; et al. Exploring Three-Dimensional Matrix-Assisted Laser Desorption/Ionization Imaging Mass Spectrometry Data: Three-Dimensional Spatial Segmentation of Mouse Kidney. *Anal. Chem.* **2012**, *84* (14), 6079–6087. https://doi.org/10.1021/ac300673y.
(19) Calligaris, D.; Norton, I.; Feldman, D. R.; Ide, J. L.; Dunn, I. F.; Eberlin, L. S.; Cooks, R. G.; Jolesz, F. A.; Golby, A. J.; Agar, N. Y. Mass Spectrometry Imaging as a Tool for Surgical Decision- Making. *J. Mass Spectrom.* **2013**, *48* (11), 1178–1187. https://doi.org/10.1002/jms.3295.
(20) Franceschi, P.; Wehrens, R. Self-Organizing Maps: A Versatile Tool for the Automatic Analysis of Untargeted Imaging Datasets. *Proteomics* **2014**, *14* (7–8), 853–861. https://doi.org/10.1002/pmic.201300308.

(21) Anselin, L. Local Indicators of Spatial Association-LISA. *Geogr. Anal.* **2010**, *27* (2), 93–115. https://doi.org/10.1111/j.1538-4632.1995.tb00338.x.

(22) Lin, W. C.; Lin, Y. P.; Wang, Y. C.; Chang, T. K.; Chiang, L. C. Assessing and Mapping Spatial Associations among Oral Cancer Mortality Rates, Concentrations of Heavy Metals in Soil, and Land Use Types Based on Multiple Scale Data. *Int. J. Environ. Res. Public Health* **2014**, *11* (2), 2148–2168. https://doi.org/10.3390/ijerph110202148.

(23) Sheskin, D. J. *Handbook of Parametric and Nonparametric Statistical Procedures*; Chapman and Hall/CRC, 2007; Vol. 51. https://doi.org/10.2307/2685909.

(24) Bivand, R. S.; Pebesma, E. J.; Gomez-Rubio, V. *Applied Spatial Data Analysis with R*; Springer New York: New York, NY, 2008. https://doi.org/10.1007/978-0-387-78171-6.

(25) Trede, D.; Schiffler, S.; Becker, M.; Wirtz, S.; Steinhorst, K.; Strehlow, J.; Aichler, M.; Kobarg, J. H.; Oetjen, J.; Dyatlov, A.; et al. Exploring Three-Dimensional Matrix-Assisted Laser Desorption/Ionization Imaging Mass Spectrometry Data: Three-Dimensional Spatial Segmentation of Mouse Kidney. *Anal. Chem.* **2012**, *84* (14), 6079–6087. https://doi.org/10.1021/ac300673y.

(26) Widlak, P.; Mrukwa, G.; Kalinowska, M.; Pietrowska, M.; Chekan, M.; Wierzgon, J.; Gawin, M.; Drazek, G.; Polanska, J. Detection of Molecular Signatures of Oral Squamous Cell Carcinoma and Normal Epithelium - Application of a Novel Methodology for Unsupervised Segmentation of Imaging Mass Spectrometry Data. *Proteomics* **2016**, *16* (11–12), 1613–1621. https://doi.org/10.1002/pmic.201500458.

(27) Louie, K. B.; Bowen, B. P.; McAlhany, S.; Huang, Y.; Price, J. C.; Mao, J.; Hellerstein, M.; Northen, T. R. Mass Spectrometry Imaging for in Situ Kinetic Histochemistry. *Sci. Rep.* **2013**, *3* (1), 1656. https://doi.org/10.1038/srep01656.

(28) Ng, N.; Sandberg, M.; Ahlström, G. Prevalence of Older People with Intellectual Disability in Sweden: A Spatial Epidemiological Analysis. *J. Intellect. Disabil. Res.* **2015**, *59* (12), 1155–1167. https://doi.org/10.1111/jir.12219.

(29) Wang, T.; Wang, X.; Tie, P.; Bai, Y.; Zheng, Y.; Yan, C.; Chai, Z.; Chen, J.; Rao, H.; Zeng, L.; et al. Spatio-Temporal Cluster and Distribution of Human Brucellosis in Shanxi Province of China between 2011 and 2016. *Sci. Rep.* **2018**, *8* (1), 1–10. https://doi.org/10.1038/s41598-018-34975-7.

(30) Cesca, M.; Morosi, L.; Berndt, A.; Fuso Nerini, I.; Frapolli, R.; Richter, P.; Decio, A.; Dirsch, O.; Micotti, E.; Giordano, S.; et al. Bevacizumab-Induced Inhibition of Angiogenesis Promotes a More Homogeneous Intratumoral Distribution of Paclitaxel, Improving the Antitumor Response. *Mol. Cancer Ther.* **2016**, *15* (1), 125–135. https://doi.org/10.1158/1535-7163.MCT-15-0063.

(31) Bai, J.; Wang, M. X.; Chowbay, B.; Ching, C. B.; Chen, W. N. Metabolic Profiling of HepG2 Cells Incubated with S(-) and R(+) Enantiomers of Anti-Coagulating Drug Warfarin. *Metabolomics* **2011**, *7* (3), 353–362. https://doi.org/10.1007/s11306-010-0262-3.

(32) Team, R. C. R: A Language and Environment for Statistical Computing. **2017**.

(33) Gibb, S.; Strimmer, K. Maldiquant: A Versatile R Package for the Analysis of Mass Spectrometry Data. *Bioinformatics* **2012**, *28* (17), 2270–2271. https://doi.org/10.1093/bioinformatics/bts447.

(34) Gong, L.; Constantine, W.; Chen, Y. A. MsProcess: Protein Mass Spectra Processing. **2012**, *R package*.

(35) McDonnell, L. A.; van Remoortere, A.; van Zeijl, R. J. M.; Deelder, A. M. Mass Spectrometry Image Correlation: Quantifying Colocalization. *J. Proteome Res.* **2008**, *7* (8), 3619–3627. https://doi.org/10.1021/pr800214d.

(36) Rocke D, Lee GC, Tillinghast J, Durbin-Johnson B, W. S. LMGene: LMGene Software for Data Transformation and Identification of Differentially Expressed Genes in Gene

Expression Arrays. **2018**, *R package*.
(37) Smyth, G. K.; Ritchie, M.; Thorne, N. Linear Models for Microarray and RNA-Seq Data User ’ s Guide. **2018**, No. April.
(38) Steinbach, M.; Karypis, G.; Kumar, V. A Comparison of Document Clustering Techniques. In *In KDD Workshop on Text Mining*; 2000. https://doi.org/10.1109/ICCCYB.2008.4721382.
(39) Calinski, T.; Harabasz, J. A Dendrite Method for Cluster Analysis. *Commun. Stat. - Theory Methods* **1974**, *3* (1), 1–27. https://doi.org/10.1080/03610927408827101.
(40) Lucas, M. A. Amap: Another Multidimensional Analysis Package. **2018**, No. R package version 0.8-16.
(41) Henning, C. Fpc: Flexible Procedures for Clustering. **2018**, No. R package version 2.1-11.1.
(42) Pinheiro J, Bates D, DebRoy S, S. D. and R. C. T. (2017). Nlme: Linear and Nonlinear Mixed Effects Models. **2017**, *R package*.
(43) Plant, R. E. *Spatial Data Analysis in Ecology and Agriculture Using R*; Intergovernmental Panel on Climate Change, Ed.; Cambridge University Press: Cambridge, 2012. https://doi.org/10.1017/CBO9781107415324.004.
(44) Kissling, W. D.; Carl, G. Spatial Autocorrelation and the Selection of Simultaneous Autoregressive Models. **2008**, 59–71. https://doi.org/10.1111/j.1466-8238.2007.00334.x.
(45) Franceschi, P.; Giordan, M.; Wehrens, R. Multiple Comparisons in Mass-Spectrometry-Based -Omics Technologies. *TrAC Trends Anal. Chem.* **2013**, *50*. https://doi.org/https://doi.org/10.1016/j.trac.2013.04.011.
(46) Fuso Nerini, I.; Morosi, L.; Zucchetti, M.; Ballerini, A.; Giavazzi, R.; D’incalci, M. Intratumor Heterogeneity and Its Impact on Drug Distribution and Sensitivity. *Clin. Pharmacol. Ther.* **2014**, *96* (2), 224–238. https://doi.org/10.1038/clpt.2014.105.
(47) Thompson, C. G.; Bokhart, M. T.; Sykes, C.; Adamson, L.; Fedoriw, Y.; Luciw, P. A.; Muddiman, D. C.; Kashuba, A. D. M.; Rosen, E. P. Mass Spectrometry Imaging Reveals Heterogeneous Efavirenz Distribution within Putative HIV Reservoirs. *Antimicrob. Agents Chemother.* **2015**, *59* (5), 2944–2948. https://doi.org/10.1128/AAC.04952-14.
(48) Creative Commons Attribution 4.0 International License.

Manuscript

Click here to access/download;Manuscript;Manuscript withTrackChanges.docx

A methodological approach to correlate tumor heterogeneity with drug distribution profile in mass spectrometry imaging (MSI) data

Mridula Prasad[1,2], Geert Postma[1*], Pietro Franceschi[2], Lavinia Morosi[3], Silvia Giordano[4], Francesca Falcetta[3], Raffaella Giavazzi[3], Enrico Davoli[4], Lutgarde M.C. Buydens[1], Jeroen Jansen[1]

[1]IMM/ Analytical Chemistry, Radboud University, Heyendaalseweg, 6525 AJ Nijmegen, Netherlands.

[2]Unit of Computational Biology, Research and Innovation Center, Fondazione Edmund Mach, 38010, San Michele all' Adige, Italy.

[3]Department of Oncology, Istituto di Ricerche Farmacologiche Mario Negri IRCCS, Via La Masa, 19-20156 Milan Italy.

[4]Mass Spectrometry Laboratory, Istituto di Ricerche Farmacologiche Mario Negri IRCCS, Via La Masa, 19-20156 Milan Italy,

ABSTRACT: Mass spectrometry imaging (MSI) has become a valuable tool in drug imaging because of its ability to provide a simultaneous spatial distribution of the drug and several other molecular ions present in the biological sample. An important application is the evaluation of homogeneity/heterogeneity of drug distribution in solid tumors. Solid tumors are known to be made up of different tissue subpopulations and their heterogeneity is supposed to have a direct and/or indirect influence on drug distribution. Hence, for further enhancement of penetration therapy performance, it is important to link a characterization of the tumor microenvironment with drug homogeneity. In this study, untargeted MSI data were used to understand the spatial heterogeneity within solid tumors, assessing its impact on the drug (paclitaxel) distribution. The proposed approach was applied on MSI datasets already analyzed, focusing on tumor drug distribution. Untargeted MSI datasets were collected on different tumor xenograft

models (ovarian and colon cancer cell lines) pre-treated or not with anti-angiogenesis compound (bevacizumab). Our main data analysis steps involved: a) pre-processing of MSI data to make all biological samples directly comparable, b) unsupervised data clustering to find different tissue subtypes, c) quantification of drug heterogeneity using local indicators of spatial association (LISA) map and d) selection of important ion signals from identified clusters of interest using the spatial -aware statistical tools. Our clustering results show variation in tumor subpopulations and less spatial heterogeneity in the MSI data collected on samples treated with the anti-angiogenesis compound consistently with our previous data. The local spatial structures identified in drug ion LISA maps show a correlation with clusters identified using a clustering method. Using the right spatial method, we were able to reduce the number of false-positive ions selected and identified the one that shows relevant spatial patterns in different tissue subtypes. Finally, our overall study shows that there is a direct association in drug homogeneity and spatial arrangement of different tissue subtypes in a solid tumor.

Introduction

Heterogeneity of the tumor microenvironment directly affects drug penetration reducing the therapeutic efficacy and contributing to the development of resistance[1,2]. For the above-mentioned reasons, knowledge about tumor spatial heterogeneity is then of paramount importance to optimize therapeutic outcomes[3]. In order to understand the relation between drug distribution and tissue homogeneity/heterogeneity, an imaging technique able to investigate both phenomena simultaneously would be highly desirable[4].

Mass spectrometry imaging (MSI) is a molecular imaging technique that provides simultaneous information about spatial localization of drugs and other small molecules present in the biological sample[5,6]. It is a valuable technique ~~one of the techniques of election~~ to characterize tumor tissue subtypes[7–9]and it is also applied to map the distribution of drugs within the tissue[10–13]. Untargeted MSI datasets provide unprecedented opportunities to understand the drug distribution in association with tissue heterogeneity. Several computational algorithms have been implemented on MSI data for the

identification of tumor tissue areas[7,8,14]and important molecular ions selection[8,15,16]. As far as our knowledge there is no work done that combines the tumor heterogeneity with a drug distribution profile. In a recent paper, we have proposed a new objective index (DHI)[17] to measure drug homogeneity in different MSI datasets and we aim at extending this work proposing a framework that can be used to investigate the relation between drug homogeneity and the observed tumor heterogeneity. The proposed approach encompasses the following steps.

1. Identification of the different tissue sub-populations from an untargeted multi-sample MSI dataset (segmentation).
2. Quantitative analysis of the spatial arrangement of the tissue subtypes across different samples.
3. Extraction of discrete drug distribution maps.
4. Matching of the segmented MSI dataset with the drug distribution maps.
5. Selection of ion signals that can be used to differentiate specific tissue areas.

Since the accessibility of a drug to a particular spatial location is expected to be linked to the metabolic and histological characteristics of the underlying tissues, multivariate unsupervised clustering was used to group the pixels into a limited number of groups which represent the different tissue subtypes[8,9,14,18–20] (Step 1). This step was performed after removing the drug-related peaks to avoid their influence in groups formation. The quantitative assessment of the spatial arrangement of the different clusters (Step 2) was performed by using a modified version of our DHI[17] index, which was optimized to characterize the spatial arrangement of the pixels on the clustered images. The *local indicators of spatial association (LISA)*[21,22] method was instead applied to create discrete drug distribution maps (Step 3). The association between LISA maps and identified segments was derived using Cramer's[23] V method (Step 4). The identification of important ion signals able to differentiate the tissue subtypes was finally performed by using a spatial-aware statistical method that corrects for spatial autocorrelation[24] (Step 5). Since the application of these models in MSI is still uncommon, the optimal spatial model for biomarker selection was tested on a synthetic autocorrelated spatial dataset.

In our study, the identification of tissue subpopulations was performed using K-means clustering with correlation distance. This non-spatial clustering method has already been used in many other MSI

research[25–27] and was able to efficiently extract relevant structures. The spatially relevant clusters in drug ion image were detected based on Moran's scatter plot or LISA map. LISA map is a commonly used method in spatial statistics where pixels are grouped into different clusters (zones) based on their similar or different behavior with neighboring pixels[28,29]. The potential of the proposed approach was tested on the same datasets that we used in our previous work[17,30] which consisted of the MSI untargeted analysis of a set of different tumor xenograft models from ovarian (A2780) and colon (HCT116) cancer cell lines. The study was designed to assess the effect of an anti-angiogenesis compound (bevacizumab) on the drug (paclitaxel) distribution[30]. For this purpose, half of the animals were pretreated twice (5 and 1 days before excision) with bevacizumab, before being administered with paclitaxel a few hours (six) before excision. Even if it is impossible to exclude that the anticancer drug could be responsible for the observed spatial heterogeneity in metabolic profile[31], any relevant difference between the two treatment groups is likely to be associated with the direct or indirect effect of bevacizumab.

EXPERIMENTAL SECTION

MSI datasets

MSI data were derived from the tumor-bearing mice, treated with paclitaxel (60 mg/kg) alone or in combination with bevacizumab (two intraperitoneal injections at 150 μg per mouse). For mice experiment, IRFMN adheres to the principles set out in the following laws, regulations, and policies governing the care and use of laboratory animals: Italian Governing Law (D.lgs 26/2014; Authorization n.19/2008-A issued March 6, 2008 by Ministry of Health); Mario Negri Institutional Regulations and Policies providing internal authorization for persons conducting animal experiments (Quality Management System Certificate–UNI EN ISO 9001:2008 –Reg. N° 6121); the NIH Guide for the Care and Use of Laboratory Animals (2011 edition) and EU directives and guidelines (EEC Council Directive 2010/63/UE).

The complete details of the experiment experiments described here.[12,17,30]. Briefly, tumors were collected, frozen in liquid nitrogen and prepared for MSI analysis. A MALDI 4800 TOF-TOF (AB

SCIEX, Old Connecticut Path, Framingham, MA) was used. And, mass spectra were recorded in full-scan-profile mode over a limited mass range (*m*/*z* 199–500). Images of tissue sections were acquired using the 4800 Imaging Tool software with an imaging raster of 100 × 100 µm (pixel dimension of ca. 0.01 mm^2). The obtained dataset consisted of 131 349 (A2780) and 59 652 (HCT116) raw spectra from the tissue areas of all tumor models.

MSI data pre-processing

The initial data files in Analyze 7.5 format were opened in R[32] free software version 3.4.3 using the MALDIquant[33] package. Each MSI datafile contains mass spectra collected from both tumor tissue and a glass slide. To avoid any bias in pre-processing and data analysis steps, the mass spectra belonging to tumor tissue only were used. The identification of tumor tissue was performed by constructing a mask of the ion signal detected in the m/z = 281.1-281.44 range. This ion was confirmed to be a reliable tissue marker by visually comparing all MS images with their optical counterparts. To correct for possible spectral misalignments across the different datasets, an adaptive binning approach was applied. To do this, first, a reference spectrum was created, which is a single maximum intensity spectrum of all the spectra. The main reasoning behind it is to acquire knowledge about the location and shape of all the ion peaks in our data and then optimize the bin size accordingly. Smoothing of the reference spectrum was performed by applying discrete wavelet transformation (Daubechies least asymmetric 8-tap filter with hard shrinkage) using a msDenoiseWavelet function from msProcess[34] R package. Peaks in the reference spectrum were identified by local maxima search above a certain threshold[17]. Initial bins were created based on those identified peaks. The bin size was further optimized on the bases of instrument characteristics merging together peaks showing a difference of less than 0.05m/z. To avoid that peaks are missed in low-intensity spectra, an extra bin of size 0.5 was added in the presence of a large gap between two identified peaks (especially towards the end of the spectra). The complete workflow of bin creation and maximum intensity spectra from two tumor MSI data are shown in Additional file 1.

After bin identification, each MSI data file was reopened and peak picking was performed using an approach similar to the one applied to the reference spectrum and the identified peaks were assigned to their corresponding bins. To focus only on the more common ions, peaks that were present in less than 20% of the tissue area were removed. Peaks only present in a single tumor model were rejected. To remove spatial noise, median filtering with a window size of 3x3 pixels was performed on each extracted ion image. We also performed edge correction in the individual dataset, as marginal pixels have enormously high intensity due to the surface difference between tissue and glass slide[35]. image. To make the spectra collected on the different pixels comparable median normalization was performed. Generalized log transformation was performed as a variance stabilization step using LMGene[36] in R. A plausible batch effect between the slices of certain mouse models was removed using the removeBatchEffect function from R limma[37] package. Ion peaks with correlation with the drug compound (paclitaxel) (>0.9 ~~0.5~~) were removed before cluster analysis. The overall dimension of the final data matrix belonging to A2780 and HCT116 is equal to 131 349 x 173 and 59   652 x 155, respectively.

MSI data analysis

Unsupervised data clustering

Segmentation of MSI data collected on the complete set of sections from a specific cell-line was performed by unsupervised bisect k-means clustering[38] using correlation as a similarity measure. The individual clusters were allowed to split further until the largest cluster contains 40% of the pixels included in the initial data matrix. The number of clusters at each step was selected based on the calinhara internal validity index[39]. The clustering and validation were implemented using R-packages amap[40] and fpc[41], respectively. The outcomes of segmentation for the different sample groups (cell lines and bevacizumab treatment) were analyzed based on the following parameters: a) size and b) homogeneity of the individual clusters. The homogeneity of the individual clusters was assessed by using a slightly modified version of the drug homogeneity index (DHI) (see Additional File 2). Higher cluster

homogeneity means the more continuous/homogeneous distribution of a particular tissue-type. All these parameter values were normalized by the total number of pixels from the tumor models of each treatment condition. Statistical significance of pixel and homogeneity ratio under two treatment conditions was calculated using linear mixed models with the nlme[42] R-package.

Quantification assessment of the drug distribution

Spatial quantification of the drug heterogeneity was performed using the Moran's I scatter plot[24], also known as the local indicators of spatial autocorrelation (LISA) map (see Additional File 2). This spatial-aware method was selected because is expected to yield more robust results in the presence of the spatially autocorrelated drug signal.

To create a Moran's I scatter plot and/or LISA map required inputs are: original variable, spatially lagged variable and spatial weight matrix. The original variable in our case it is the ion intensity map of the drug peak. The spatially lagged variable is constructed by multiplying the autoscaled version of the original variable with the help of the spatial weight matrix[24,43]. This weight matrix stores the connections between nearby observations (e.g. in a binary weight matrix the observations which lie within a certain range of autocorrelation receive a value of one else zero). The optimal range of autocorrelation can be decided based on spatial correlogram. A spatial correlogram[24] is a 2D plot where the spatial autocorrelation index (Moran's I) is plotted as a function of lag distance where a positive value indicates the presence of autocorrelation within a certain distance range. To create a LISA map of a drug ion image the following steps were performed:

- the optimal spatial weight matrix was created on the bases of the spatial correlogram plot (Figure 1b).
- the original variable was converted into its spatially lagged version (Figure 1c).
- Moran's I scatter plot was created by regressing the original variable against its spatially lagged version where pixels are grouped into four different zones usually called high-high, low-low, high-low and low-high (Figure 1d).
- Finally, a LISA map was constructed which is a two-dimensional image where pixels are labeled according to their class in Moran's I scatter plot (Figure 1e).

Figure 1. A schematic workflow of drug LISA map creation. a) Original drug ion image. b) Spatial correlogram of the drug-ion image where Moran's I values (Ac1) are plotted against the lag distance (dists). c) spatially lagged image of the drug-ion. d) Moran's I scatter plot where the drug signal and its spatially lagged version are regressed against each other. e) LISA clustered map of the drug ion where pixels falling in the same quadrant of the Moran scatter plot are grouped.

In a LISA map or Moran's I scatter plot, the high-high zone contains pixels that have a high intensity or above-average value and surrounded by a similar type of high-intensity pixels. The low-low zone contains pixels that have a lower intensity or below-average value and surrounded by a similar type of low-intensity pixels. The high-low zone contains pixels that have above-average value for themselves but surrounded by neighbors with below-average value. The reverse rule applies to the pixels fall in the low-high zone. Note, in a LISA map pixels falling in high-high and low-low zones show positive spatial autocorrelation and are spatially smooth. Therefore, a single zone of a LISA map may contain multiple clusters with approximately similar profiles.

Association between clustered image and drug LISA map

The obtained drug LISA maps were analyzed for their association with the unsupervised clusters obtained using unsupervised clustering. A quantitative analysis was performed to understand which cluster subtypes overlap most with which zone of the LISA map, for which the fraction of pixels in different zones and clusters in each tumor model was calculated. Further, the strength of association between those two vector classes (LISA map zones and unsupervised clusters) was estimated using Cramer's V[24] method. Cramer's V is a statistical measure similar to the Pearson correlation to find the correlation between two nominal variables and returns a correlation value within the range of 0-1.

Representative ion signals selection from the identified clusters

The method used to select representative ion signals from the identified clusters in the MSI data was first validated on synthetic spatially autocorrelated data. Two spatial approaches (spatial error model (SE), and spatial lag model (SL)) were compared with a standard non-spatial approach (ordinary least square (OLS)). Both spatial models[24,44] are modified versions of an OLS model and include spatial autocorrelation in a different component of the OLS model. The comparison of the performance of the above mentioned statistical methods with complete description of the synthetic data generation process is given in Additional File 3.

All spatial models were fitted with the spdep[24] R package. Similar to what was done in the case of the LISA map, the right threshold for the spatial weight matrix was decided based on the spatial correlogram.

In MSI data, the selection of ion signals from identified clusters was performed using the method which gives the best performance on our synthetic data. In order to do that, the outcomes of the original clustering were converted into a set of two-class images where each cluster is, in turn, compared with all the others. As the variables selection using the spatial method is computationally intensive, we only used a few tumor models in which the cluster of interest was present. Thus, per cluster five different tumor slices were selected, i.e. MSI data from 4 - 5 different tumor models. If a particular ion was found to be important in all five datasets, then it was considered as a significant ion signal for the respective cluster. The important ions were selected on the bases of the model p-values corrected for multiple testing by using the procedure of Benjamini & Hochberg[45].

Results

Unsupervised clustering of MSI data

a) A2780 cell-line based MSI data

The clustering method identified five unique clusters in the combined set of A2780 xenograft models (Figure 2 left). The majority of the replicates possess those five clusters in different ratios where clusters 1 and 2 were predominant in all tumor models in both treatment conditions. The relative contribution of

cluster 3 is reduced and cluster 4 is enhanced in the presence of bevacizumab (Figure 2, top right). Cluster 3 showed a high overlap with the necrosis area[12] and noticed to be present in a relatively higher fraction among the tumor models not pretreated with bevacizumab. The small fraction of cluster 5 is present in all tumor models.

Similar to the number of pixels, the homogeneity of clusters (parameter b) for the individual tumors was calculated using the modified version of our DHI and it is shown in Figure 2, bottom right. The figure highlights the clear difference in clusters homogeneity under the two treatment conditions, especially for cluster 2. The homogeneity of cluster 2 in the presence of bevacizumab treatment is much higher than the homogeneity of any other cluster in two treatment conditions. Without bevacizumab treatment also tumor models show high homogeneity for cluster 2 followed by cluster 3.

Figure 2: Cluster analysis of A2780 tumor MSI data generated in the presence and absence of bevacizumab treatment. Left: Representation of clusters detected by the k-means method. Right: Ratio of a) pixels and b) homogeneity calculated from individual clusters under two treatment conditions. The red horizontal line is the global mean value of pixel and homogeneity ratio. Here, Pixel ratio = Number of pixels in individual clusters/Total number of pixels from all tumor MSI data under particular treatment conditions. Homogeneity ratio =Size-zone of individual clusters for a given tumor model/Total number of pixels in that particular tumor model. With Beva = pretreated with bevacizumab and Without Beva = without bevacizumab pretreatment.

b) HCT116 cell-line based MSI data

Five clusters were identified in the HCT116 tumor cell line MSI data (see Additional File 4). Similar to the A2780-1A9 tumor MSI data, there was not a large observed difference in clusters population under the two treatment conditions. Cluster 2 and 3 were predominantly present in all tumor models irrespective of the treatment conditions (see Additional File 4 Figure S-1 top right). Cluster 1 was observed in a moderate amount and very small fractions of clusters 4 and 5 were present in all tumor models. The

homogeneity assessment of the individual clusters in the two treatment conditions shows that cluster 3 has high homogeneity in the case of bevacizumab treatment. In the absence of bevacizumab treatment, clusters 2 and 3 show more homogeneity (see Additional File 4 Figure S-1 bottom right).

The statistical analysis of pixel and homogeneity ratio values was performed using a linear mixed model approach where range and p-value from both tumor models are given in Additional File 4 Table S-1. For the A2780-1A9 MSI data, the pixel ratios are not significantly different in the two treatment conditions. The homogeneity value of cluster 2 is close to significant which is derived with parameter $Nu = 5$ in our homogeneity formula. In HCT116 MSI data, the number of pixels in cluster 1 and homogeneity ratio for cluster 2 is statistically significant in two treatment conditions.

Association between clustered image and drug LISA map

The spatially distinct regions based on observed drug distribution profiles were identified using LISA maps in all tumor models. The LISA map was created using the spatial weight matrix with an autocorrelation value equal to five since in spatial correlogram derived from different tumor MSI data, high positive spatial autocorrelation was observed within this range (see Additional File 5 S-1).

A visual comparison of the clustered images and drug LISA maps confirms the link between the drug distribution profile and the underlying clusters (Figure 3). For example, homogeneously high drug distribution areas (high-high (HH) zone in the LISA map) are mostly associated to cluster type 1 and 2, while, homogeneously low drug distribution areas (low-low (LL) zone in the LISA map) correspond to cluster type 3 and 5 (Table 1). The observed association between cluster types and the different zones of the LISA map is irrespective of treatment condition across all tumor models (Figure 4).

Similar observations were made from HCT116 tumor MSI data (see Additional File 5). In HCT116 MSI data, clusters 1,2 and 3 show clear overlapping with spatially homogeneous zones of the LISA maps. The cluster 1 and 3 overlapped with high drug concentration areas in the tissue and cluster 2 with low drug concentration areas (see Additional File 5). The association between cluster 2 and low drug concentration

areas in the LISA maps is more clear for HCT116 data than for A2780-1A9 data (Additional File 5 Table S-1).

The statistical correlation between the clustered image and drug LISA map was calculated using Cramer's V method. A very small fraction of pixels falls within high-low and low-high zones of the LISA map. Therefore, the Cramer's V is calculated between HH, LL zones of LISA map with unsupervised clustered classes. For both tumor MSI data, the Cramer's value across all tumor models was found to be within the range of 0.5 -0.8 (see Additional File 5 Table S-1) that confirmed the dependency of the drug on different tumor tissue areas.

Figure 3: Individual clustered image (first column), LISA map (second column) and their combination are shown for few tumor models from A2780-1A9 MSI data. The clusters found in high-high (HH), low-low (LL), high-low (HL), and low-high (LH) zones of LISA map are highlighted. In LISA map, HH, LL, HL, and LH are zones identified in Moran's I scatter plot.

Table 1: The percentages of pixels belonging to different cluster classes falling into HH, LL, HL and LH zones of the LISA map for tumor MSI data shown in Figure 3.

		Cluster 1	Cluster 2	Cluster 3	Cluster 4	Cluster 5	Cramer's V*
Image 1	HH	**8.28%**	**23.5%**	0.58%	5.75%	3.5%	0.501
	LL	4.48%	4.92%	**7.9%**	5.26%	3.46%	
	HL	3.36%	7.85%	0.87%	2.68%	1.22%	
	LH	2.87%	5.46%	2.44%	2.58%	2.88%	
Image 2	HH	**18.15%**	**13.34%**	0.31%	2.98%	1.94%	0.643

	LL	0.86%	9.39%	0.8%	7.49%	**15.93%**	
	HL	2.83%	8.5%	0.09%	1.6%	3.76%	
	LH	2.89%	3.57%	0.43%	2.1%	3.01%	
Image 3	HH	**14.69%**	**13.55%**	0.05%	5.77%	2.19%	0.463
	LL	4.2%	11.57%	0.66%	9.22%	**14.78%**	
	HL	4.02%	7.19%	0.036%	1.56%	1.69%	
	LH	1.38%	2.65%	0.29%	1.79%	2.69%	
Image 4	HH	**21.55%**	**17.09%**	0.094%	1.95%	3.89%	0.69
	LL	3.67%	1.91%	2.29%	6.2%	**18.62%**	
	HL	3.12%	2.20%	0.21%	1.93%	3.16%	
	LH	3.30%	3.44%	0.67%	1.4%	3.29%	
Image 5	HH	**16.41%**	**20.53%**	2.49%	4.2%	2.95%	0.601
	LL	2.20%	3.10%	**11.89%**	2.33%	2.15%	
	HL	3.51%	4.43%	2.89%	1.43%	1.79%	
	LH	3.36%	5.99%	3.13%	2.74%	2.44%	
Image 6	HH	**15.04%**	**23.57%**	1.53%	0.99%	3.23%	0.62
	LL	4.04%	4.12%	**13.44%**	2.05%	**4.37%**	
	HL	3.71%	6.59%	2.12%	1.19%	2.23%	
	LH	2.85%	3.64%	2.83%	0.98%	1.44%	

*Cramer's Correlation is calculated between HH, LL zones of LISA map and unsupervised clusters.

Figure 4: Quantitative analysis to find the association between drug LISA maps and identified clusters from complete A2780-1A9 tumor MSI data. Here, each subplot highlights the fraction of pixels present in different zones of the LISA map under two treatment conditions. The red horizontal line in each subplot is a global mean value for pixel ratio for that particular zone. With Beva: pre-treated with bevacizumab and without Beva: without pre-treatment with bevacizumab

Ion signals selection from the identified clusters

For variables selection, the performances of spatial (SE and SL) and non-spatial (OLS) methods were tested on synthetic spatially autocorrelated data. The SL method completely outperformed the other two methods (see Additional File 3); therefore, used for m/z values selection from unsupervised clusters identified in two MSI cancer datasets (Table 2). Note, cluster 4 identified in A2780-1A9 tumor data was present in a single tumor model and cluster 4 and 5 for HCT116 was present in very small fraction and did not follow any proper spatial structure , therefore those clusters were excluded from the ions selection step. The list of important ions is given in Additional file 9. In the A2780 cancer data, cluster 2 had 28 ions showing a significant difference. In particular, the ion at m/z=335.41 had high intensity in all tumor models. The ion image of this particular ion showed a homogeneous distribution in the cluster (see Additional File 6 Figure S-1 top row). A larger number of significantly different ions was identified for cluster 3, mostly with low signal intensity.

For the HCT116 MSI data, a large number of significant ions was identified in cluster 2 and 3. In cluster 3, the majority of ions had a high signal or positive regression coefficients in the spatial model, with the ion at m/z =281.315 showing the highest value. In cluster 2, ions with both high and low signals were present in equal portions (see Additional File 6 Figure S-1 bottom row).

Table 2: The number of ion signals selected from different clusters in two tumor MSI data

Cluster type	A2780	HCT116
1	26	22
2	28	83
3	91	70

5	35

Discussion

Several studies have shown that the tissue spatial heterogeneity within a solid tumor impacts on the drug distribution[2,10,12,30,46,47]. Different tissue characteristics and tumor microenvironment affect the drug distribution which means that the concentration of a drug at a given spatial location could be related to the tissue composition at that point. It is also true that the presence of the drug can induce a modification of the tumor structure[31], so in general, it is impossible to disentangle the two phenomena. In our case, the situation was fortunate since a part of the tumor-bearing mice had received bevacizumab treatment before drug (paclitaxel) treatment. Therefore, we assume that the observed differences in the spatial organization of tissue areas characterized by similar metabolic fingerprints can be interpreted as a direct or indirect effect of bevacizumab treatment which was given twice before drug injection. This was also suggested by our previous study showing an increase in drug homogeneity in samples treated with bevacizumab[30]. The main goal of this research was to show that computational methods can be used to explore and quantify spatial heterogeneity within tumors and link the observed homo-/heterogeneous drug distribution to the alteration in microenvironment due to applied therapeutic strategy. To achieve these objectives, our data analysis involved a combination of methods from different research streams. First, the clustering of combined MSI data was performed using K-means with correlation distance and then those clusters were linked with drug distribution patterns obtained using the LISA method. As stated in the introduction, K-means clustering is efficiently being used in several studies performed on MSI data for the selection of relevant clusters[25–27]. Moreover, in another study, the authors also tested this method and compared it with several spatial methods, i.e., using simulated and real data and came to the same conclusion (unpublished observation). In the spatial data analysis field, a LISA map is a commonly known technique that identifies the spatially relevant clusters in a single two-dimensional

image[21,22,43]. The LISA map provides an automated way to find spatially homogeneous clusters that are difficult to generate using a simple thresholding approach (see Additional File 8). Simple binary images were created using different threshold values. The images constructed with a threshold value of four show some resemblance with the LISA map (see Additional File 8). The binary images are not fully able to mimic the drug distribution profile. Moreover, the selection of the right threshold value from the drug ion image histogram is not very straightforward. In contrast, the LISA map is able to highlight the observed high and low intensity spatially homogeneous areas in drug ion images efficiently where for spatial weight matrix can be selected based on spatial correlogram.

The clustering of MSI data was able to identify metabolic separated regions which cannot be observed in H&E stained tissue images[25]. This is clear if one compares the images shown in Figure 5. The segmented image shows tissue subtypsubtypees in addition to the one which can be associated to the necrotic and fibrous regions on H&E stained image. The effect of the treatment with bevacizumab was visible in the segmented images. In particular, the antiangiogenic compound was increasing homogeneity (both in the pattern of clusters and in the drug) even if no anti-angiogenesis treatment specific cluster was identified (Figure 2 and Additional File 4). Interestingly, the average drug concentration in the individual clusters under the two treatment conditions is approximately equal these changes in distribution were not affecting drug total concentration, which was approximately equal in the two treatment conditions (see Additional File 7). This confirmed the results of our previous publication[17] and our homogeneity assessment of clustered images (Figure 2 bottom right) that the tumor tissues from bevacizumab treatment are more homogeneous.

Quantitative analysis of the LISA maps shows the dependency of highlights the association between thethe observed drug distribution profiles and the underlying tissue-types metabolic fingerprint (Figure 4

and Additional File 5). In A2780-1A9 tumors, clusters 1 and 2 have a high affinity for the drug and while clusters 3 and 5 show a lower than the average drug concentration (Figure 4 and Table 1).

~~In A2780-1A9 tumors, clusters 1 and 2 overlap with the high-high homogeneous region of drug LISA map and cluster 2 was the most homogeneous (Figure 2- bottom right).~~ For HCT116 tumors, clusters 1 and 3 showed some overlap with the high-high zone, and cluster 2~~3~~ with the low-low zone ~~was the one showing the higher homogeneity~~ (see Additional File 5). Remarkably, the clusters associated with the HH and LL drug distribution regions are always the same, regardless of the pretreatment with bevacizumab, which is instead affecting the arrangement of the tissue subpopulations.

The dependency of spatial methods on the spatial weight matrix

Two commonly known spatial methods (LISA map and spatial lagged regression) were used in our study to find the spatially homogeneous clusters and spatially relevant ions from different clusters, respectively. Both spatial methods required the spatial weight matrix as an input. Therefore, the dependency of results on the spatial weight matrix was tested (see Additional File 10 and 11). The variables selection was performed using the SL method for a range of autocorrelation or lag distance values (1-15) in the spatial weight matrix in tumor MSI data (see Additional File 10). The SL method selects mostly the same molecular ions at different lag distance values. All variables selected have positive Moran's I value at the above-mentioned autocorrelation range. Therefore, based on our analysis of MSI data we had not noticed

the dependency of the SL method on the spatial weight matrix. A similar observation was made when this analysis was performed on synthetic data (see Additional File 3). In the case of our synthetic data, the accuracy of SL starts decreasing after a lag distance of 6 since some false positive selection was observed, but sensitivity remains constant as the true variables were all selected. However, in the case of real MSI data, this type of plot is not feasible. Therefore we looked at Moran's spatial autocorrelation value of all selected variables (see Additional File 10). After the lag distance of 5 the Moran's I value of the selected variables starts to approach zero. Therefore, even though in our analysis the list of molecular ions was mostly consistent we will not recommend going beyond the lag distance of 5, as at such large autocorrelation range we may start including some noisy variables in our list.

A similar approach is used to test the dependency of a LISA map on the spatial weight matrix. The LISA map of the drug ion image for a particular tumor tissue was constructed with different spatial weight matricesx (see Additional File 10). The LISA map created with the lag distance of one contains large homogenous areas (LL and HH). On the increasing the lag distance, the pixels from different areas start mixing, i.e. the size of HL and LH regions gradually start increasing and the LISA map becomes less reliable. Therefore similar to the spatial regression method, the spatial weight matrix with a maximum lag distance of 5 is preferable for a LISA map.

In summary, in this paper, we providedd a computational approach to understand the problem of drug homogeneity in association with tumor heterogeneity which validates the few conclusions made in previous studies. We provide a complete workflow ofdata pre-processing of MSI data, the association between the drug and identified clusters, and the selection of molecular from identified clusters. In our paper, we have used different methods to get- different pieces of information. The dependency of spatial methods on the spatial weight matrix is discussed above. Apart from that, if theuser wants to use our approach on their MSI data, they also need to consider the parameters used in our studies, such as peaks

removal with less than 20% coverage area and the clustering index. In our case, before setting a 20% threshold we tried a 10 -30% threshold to be sure if the majority of noisy peaks have been removed and no other important peaks. A similar task was performed during the clustering of MSI data. The number of clusters was selected using the internal clustering index method but the stopping criteria in bisect clustering (40%) was set according to our data dimension and the number of expected clusters in our MSI data. If the MSI data contain very small spatial structures than this threshold needs to be reduced. In general, we believe that our paper completes the pipeline for the analysis of untargeted drug MSI data and will be useful for the groups working with a similar problem. A R pipeline includes all the methods from our papers is online available (RRID: SCR_018962, bio.tools: corrdrugtumormsi) In addition, the proposed framework allowed to robustly select molecular signals which characterize the different tissue subpopulations which can be used to monitor the effects of therapeutic strategies on the tumor spatial heterogeneity. Considering that the focus of our investigation was methodological, we have not dealt with the identification of the characteristic ions, and this would require additional (and extensive) experimental efforts.

Figure 5: Comparison of tumor tissue optical images (rightleft column) with its clustered image (middle left column) and drug LISA (right column) image from two tumors MSI data (A2780-1A9, HCT116). The black and red dots in the H&E stained images represent the necrotic and fibrotic area, respectively. In the above figure, tThe optical images are adapted from a scientific journal[10] published under CC BY license[48]. In the above LISA map HH (high-high), LL (low-low), HL (high-low), and LH (low-high) are zones identified in Moran's I scatter plot.

Conclusions

In cancer research, one of the causes of drug therapy failure is tumor drug resistance often induced by scare drug penetration. This phenomenon is supposed to be linked to the presence of diverse tumor

microenvironments which are difficult to identify with established histological techniques. In this work, we show that a molecular imaging technique like MSI, coupled with advanced data analysis strategies, offers a great opportunity to investigate the link between drug distribution and tissue heterogeneity. Our approach allowed to simultaneously investigate tissue histology and drug distribution and it was capable of detecting the effects on the tumor heterogeneity induced by a specific intervention (treatment with bevacizumab). We hope that the unsupervised approach proposed here will help oncologists to quantitatively evaluate the efficacy of therapeutic strategies.

ASSOCIATED CONTENT

Availability of supporting data

The datasets and R-script used to generate the results of this article are available on the DNAS-KNAW repository (https://doi.org/10.17026/dans26h-kstf).

Additional files

Additional File 1: Figures show workflow of adaptive bins creation and maximum intensity spectrum from two tumor models.

Additional File 2: Description of the statistical methods used in this manuscript.

Additional File 3: Synthetic spatially autocorrelated data generation steps and comparison of spatial and non-spatial methods for variables selection on synthetic data.

Additional File 4: Cluster analysis of HCT116 MSI. Range and significance values for pixels and homogeneity ratio from different clusters under two treatment condition.

Additional File 5: Overlay of clustered image and LISA map and contingency table from HCT116 MSI data. And, the quantitative analysis of LISA maps for HCT116 tumor MSI data.

Additional File 6: MS images of selected molecular ions from different clusters from tumor MSI data.

Additional File 7: Plot of drug concentration in different clusters.

Additional File 8: Comparison of drug binary image created by selecting manual threshold value based on histogram and LISA method.

Additional file 9: Excel sheet contains selected important ion signals from identified clusters of our cancer MSI data.

Additional file 10: Sensitivity analysis for spatial weight matrix in spatial methods

Additional file 11:List of molecular ions selected for clustered image shown in Additional file 10

AUTHOR INFORMATION

Corresponding Author

* E-mail: chemometrics@science.ru.nl

Competing Interests

The authors declare no competing interests.

Author Contributions

MP analyzed MSI data and wrote first draft of the paper under supervision of GP, PF, JJ and LB. GP, PF, and JJ edit the manuscript. ED, LM, SG, FF and RG did MSI experiment and review the manuscript. All authors read and approved the final manuscript.

REFERENCES

(1) Minchinton, A. I.; Tannock, I. F. Drug Penetration in Solid Tumours. *Nat. Rev. Cancer* **2006**, *6* (8), 583–592. https://doi.org/10.1038/nrc1893.

(2) Trédan, O.; Galmarini, C. M.; Patel, K.; Tannock, I. F. Drug Resistance and the Solid Tumor Microenvironment. *J. Natl. Cancer Inst.* **2007**, *99* (19), 1441–1454. https://doi.org/10.1093/jnci/djm135.

(3) Zhao, B.; Hemann, M. T.; Lauffenburger, D. A. Intratumor Heterogeneity Alters Most Effective Drugs in Designed Combinations. *Proc. Natl. Acad. Sci. U. S. A.* **2014**, *111* (29), 10773–10778. https://doi.org/10.1073/pnas.1323934111.

(4) Heindl, A.; Nawaz, S.; Yuan, Y. Mapping Spatial Heterogeneity in the Tumor Microenvironment: A New Era for Digital Pathology. *Lab. Investig.* **2015**, *95* (4), 377–384. https://doi.org/10.1038/labinvest.2014.155.

(5) Wiseman, J. M.; Ifa, D. R.; Zhu, Y.; Kissinger, C. B.; Manicke, N. E.; Kissinger, P. T.; Cooks, R. G. Desorption Electrospray Ionization Mass Spectrometry: Imaging Drugs and Metabolites in Tissues. *Proc. Natl. Acad. Sci.* **2008**, *105* (47), 18120–18125. https://doi.org/10.1073/pnas.0801066105.

(6) Prideaux, B.; Stoeckli, M. Mass Spectrometry Imaging for Drug Distribution Studies. *J. Proteomics* **2012**, *75* (16), 4999–5013. https://doi.org/10.1016/j.jprot.2012.07.028.

(7) Jones, E. A.; van Remoortere, A.; van Zeijl, R. J. M.; Hogendoorn, P. C. W.; Bovée, J. V. M. G.; Deelder, A. M.; McDonnell, L. A. Multiple Statistical Analysis Techniques Corroborate Intratumor Heterogeneity in Imaging Mass Spectrometry Datasets of Myxofibrosarcoma. *PLoS One* **2011**, *6* (9), e24913. https://doi.org/10.1371/journal.pone.0024913.

(8) Abdelmoula, W. M.; Balluff, B.; Englert, S.; Dijkstra, J.; Reinders, M. J. T.; Walch, A.

Data-Driven Identification of Prognostic Tumor Subpopulations Using Spatially Mapped t-SNE of Mass Spectrometry Imaging Data. *PNAS* **2016**, *113* (43), 12244–12249. https://doi.org/10.1073/pnas.1510227113.
(9) Inglese, P.; McKenzie, J. S.; Mroz, A.; Kinross, J.; Veselkov, K.; Holmes, E.; Takats, Z.; Nicholson, J. K.; Glen, R. C. Deep Learning and 3D-DESI Imaging Reveal the Hidden Metabolic Heterogeneity of Cancer. *Chem. Sci.* **2017**, *8* (5), 3500–3511. https://doi.org/10.1039/c6sc03738k.
(10) Giordano, S.; Morosi, L.; Veglianese, P.; Licandro, S. A.; Frapolli, R.; Zucchetti, M.; Cappelletti, G.; Falciola, L.; Pifferi, V.; Visentin, S.; et al. 3D Mass Spectrometry Imaging Reveals a Very Heterogeneous Drug Distribution in Tumors. *Sci. Rep.* **2016**, *6* (1), 37027. https://doi.org/10.1038/srep37027.
(11) Thompson, C. G.; Bokhart, M. T.; Sykes, C.; Adamson, L.; Fedoriw, Y.; Luciw, P. A.; Muddiman, D. C.; Kashuba, A. D. M.; Rosen, E. P. Mass Spectrometry Imaging Reveals Heterogeneous Efavirenz Distribution within Putative HIV Reservoirs. *Antimicrob. Agents Chemother.* **2015**, *59* (5), 2944–2948. https://doi.org/10.1128/AAC.04952-14.
(12) Giordano, S.; Zucchetti, M.; Decio, A.; Cesca, M.; Fuso Nerini, I.; Maiezza, M.; Ferrari, M.; Licandro, S. A.; Frapolli, R.; Giavazzi, R.; et al. Heterogeneity of Paclitaxel Distribution in Different Tumor Models Assessed by MALDI Mass Spectrometry Imaging. *Sci. Rep.* **2016**, *6* (1), 39284. https://doi.org/10.1038/srep39284.
(13) Walch, A.; Rauser, S.; Deininger, S.-O.; Höfler, H. MALDI Imaging Mass Spectrometry for Direct Tissue Analysis: A New Frontier for Molecular Histology. *Histochem. Cell Biol.* **2008**, *130* (3), 421–434. https://doi.org/10.1007/s00418-008-0469-9.
(14) Balluff, B.; Frese, C. K.; Maier, S. K.; Schöne, C.; Kuster, B.; Schmitt, M.; Aubele, M.; Höfler, H.; Deelder, A. M.; Heck, A. J.; et al. De Novo Discovery of Phenotypic Intratumour Heterogeneity Using Imaging Mass Spectrometry. *J. Pathol.* **2015**, *235* (1), 3–13. https://doi.org/10.1002/path.4436.
(15) Cassese, A.; Ellis, S. R.; Ogrinc Potočnik, N.; Burgermeister, E.; Ebert, M.; Walch, A.; van den Maagdenberg, A. M. J. M.; McDonnell, L. A.; Heeren, R. M. A.; Balluff, B. Spatial Autocorrelation in Mass Spectrometry Imaging. *Anal. Chem.* **2016**, *88* (11), 5871–5878. https://doi.org/10.1021/acs.analchem.6b00672.
(16) Inglese, P.; McKenzie, J. S.; Mroz, A.; Kinross, J.; Veselkov, K.; Holmes, E.; Takats, Z.; Nicholson, J. K.; Glen, R. C. Deep Learning and 3D-DESI Imaging Reveal the Hidden Metabolic Heterogeneity of Cancer. *Chem. Sci.* **2017**, *8* (5), 3500–3511. https://doi.org/10.1039/c6sc03738k.
(17) Prasad, M.; Postma, G.; Morosi, L.; Giordano, S.; Giavazzi, R.; D'Incalci, M.; Falcetta, F.; Davoli, E.; Jansen, J.; Franceschi, P. Drug-Homogeneity Index in Mass-Spectrometry Imaging. *Anal. Chem.* **2018**, *90* (22), 13257–13264. https://doi.org/10.1021/acs.analchem.8b01870.
(18) Trede, D.; Schiffler, S.; Becker, M.; Wirtz, S.; Steinhorst, K.; Strehlow, J.; Aichler, M.; Kobarg, J. H.; Oetjen, J.; Dyatlov, A.; et al. Exploring Three-Dimensional Matrix-Assisted Laser Desorption/Ionization Imaging Mass Spectrometry Data: Three-Dimensional Spatial Segmentation of Mouse Kidney. *Anal. Chem.* **2012**, *84* (14), 6079–6087. https://doi.org/10.1021/ac300673y.
(19) Calligaris, D.; Norton, I.; Feldman, D. R.; Ide, J. L.; Dunn, I. F.; Eberlin, L. S.; Cooks, R. G.; Jolesz, F. A.; Golby, A. J.; Agar, N. Y. Mass Spectrometry Imaging as a Tool for Surgical Decision- Making. *J. Mass Spectrom.* **2013**, *48* (11), 1178–1187. https://doi.org/10.1002/jms.3295.
(20) Franceschi, P.; Wehrens, R. Self-Organizing Maps: A Versatile Tool for the Automatic Analysis of Untargeted Imaging Datasets. *Proteomics* **2014**, *14* (7–8), 853–861. https://doi.org/10.1002/pmic.201300308.

(21) Anselin, L. Local Indicators of Spatial Association-LISA. *Geogr. Anal.* **2010**, *27* (2), 93–115. https://doi.org/10.1111/j.1538-4632.1995.tb00338.x.
(22) Lin, W. C.; Lin, Y. P.; Wang, Y. C.; Chang, T. K.; Chiang, L. C. Assessing and Mapping Spatial Associations among Oral Cancer Mortality Rates, Concentrations of Heavy Metals in Soil, and Land Use Types Based on Multiple Scale Data. *Int. J. Environ. Res. Public Health* **2014**, *11* (2), 2148–2168. https://doi.org/10.3390/ijerph110202148.
(23) Sheskin, D. J. *Handbook of Parametric and Nonparametric Statistical Procedures*; Chapman and Hall/CRC, 2007; Vol. 51. https://doi.org/10.2307/2685909.
(24) Bivand, R. S.; Pebesma, E. J.; Gomez-Rubio, V. *Applied Spatial Data Analysis with R*; Springer New York: New York, NY, 2008. https://doi.org/10.1007/978-0-387-78171-6.
(25) Trede, D.; Schiffler, S.; Becker, M.; Wirtz, S.; Steinhorst, K.; Strehlow, J.; Aichler, M.; Kobarg, J. H.; Oetjen, J.; Dyatlov, A.; et al. Exploring Three-Dimensional Matrix-Assisted Laser Desorption/Ionization Imaging Mass Spectrometry Data: Three-Dimensional Spatial Segmentation of Mouse Kidney. *Anal. Chem.* **2012**, *84* (14), 6079–6087. https://doi.org/10.1021/ac300673y.
(26) Widlak, P.; Mrukwa, G.; Kalinowska, M.; Pietrowska, M.; Chekan, M.; Wierzgon, J.; Gawin, M.; Drazek, G.; Polanska, J. Detection of Molecular Signatures of Oral Squamous Cell Carcinoma and Normal Epithelium - Application of a Novel Methodology for Unsupervised Segmentation of Imaging Mass Spectrometry Data. *Proteomics* **2016**, *16* (11–12), 1613–1621. https://doi.org/10.1002/pmic.201500458.
(27) Louie, K. B.; Bowen, B. P.; McAlhany, S.; Huang, Y.; Price, J. C.; Mao, J.; Hellerstein, M.; Northen, T. R. Mass Spectrometry Imaging for in Situ Kinetic Histochemistry. *Sci. Rep.* **2013**, *3* (1), 1656. https://doi.org/10.1038/srep01656.
(28) Ng, N.; Sandberg, M.; Ahlström, G. Prevalence of Older People with Intellectual Disability in Sweden: A Spatial Epidemiological Analysis. *J. Intellect. Disabil. Res.* **2015**, *59* (12), 1155–1167. https://doi.org/10.1111/jir.12219.
(29) Wang, T.; Wang, X.; Tie, P.; Bai, Y.; Zheng, Y.; Yan, C.; Chai, Z.; Chen, J.; Rao, H.; Zeng, L.; et al. Spatio-Temporal Cluster and Distribution of Human Brucellosis in Shanxi Province of China between 2011 and 2016. *Sci. Rep.* **2018**, *8* (1), 1–10. https://doi.org/10.1038/s41598-018-34975-7.
(30) Cesca, M.; Morosi, L.; Berndt, A.; Fuso Nerini, I.; Frapolli, R.; Richter, P.; Decio, A.; Dirsch, O.; Micotti, E.; Giordano, S.; et al. Bevacizumab-Induced Inhibition of Angiogenesis Promotes a More Homogeneous Intratumoral Distribution of Paclitaxel, Improving the Antitumor Response. *Mol. Cancer Ther.* **2016**, *15* (1), 125–135. https://doi.org/10.1158/1535-7163.MCT-15-0063.
(31) Bai, J.; Wang, M. X.; Chowbay, B.; Ching, C. B.; Chen, W. N. Metabolic Profiling of HepG2 Cells Incubated with S(-) and R(+) Enantiomers of Anti-Coagulating Drug Warfarin. *Metabolomics* **2011**, *7* (3), 353–362. https://doi.org/10.1007/s11306-010-0262-3.
(32) Team, R. C. R: A Language and Environment for Statistical Computing. **2017**.
(33) Gibb, S.; Strimmer, K. Maldiquant: A Versatile R Package for the Analysis of Mass Spectrometry Data. *Bioinformatics* **2012**, *28* (17), 2270–2271. https://doi.org/10.1093/bioinformatics/bts447.
(34) Gong, L.; Constantine, W.; Chen, Y. A. MsProcess: Protein Mass Spectra Processing. **2012**, *R package*.
(35) McDonnell, L. A.; van Remoortere, A.; van Zeijl, R. J. M.; Deelder, A. M. Mass Spectrometry Image Correlation: Quantifying Colocalization. *J. Proteome Res.* **2008**, *7* (8), 3619–3627. https://doi.org/10.1021/pr800214d.
(36) Rocke D, Lee GC, Tillinghast J, Durbin-Johnson B, W. S. LMGene: LMGene Software for Data Transformation and Identification of Differentially Expressed Genes in Gene

Expression Arrays. **2018**, *R package*.
(37) Smyth, G. K.; Ritchie, M.; Thorne, N. Linear Models for Microarray and RNA-Seq Data User ’ s Guide. **2018**, No. April.
(38) Steinbach, M.; Karypis, G.; Kumar, V. A Comparison of Document Clustering Techniques. In *In KDD Workshop on Text Mining*; 2000. https://doi.org/10.1109/ICCCYB.2008.4721382.
(39) Calinski, T.; Harabasz, J. A Dendrite Method for Cluster Analysis. *Commun. Stat. - Theory Methods* **1974**, *3* (1), 1–27. https://doi.org/10.1080/03610927408827101.
(40) Lucas, M. A. Amap: Another Multidimensional Analysis Package. **2018**, No. R package version 0.8-16.
(41) Henning, C. Fpc: Flexible Procedures for Clustering. **2018**, No. R package version 2.1-11.1.
(42) Pinheiro J, Bates D, DebRoy S, S. D. and R. C. T. (2017). Nlme: Linear and Nonlinear Mixed Effects Models. **2017**, *R package*.
(43) Plant, R. E. *Spatial Data Analysis in Ecology and Agriculture Using R*; Intergovernmental Panel on Climate Change, Ed.; Cambridge University Press: Cambridge, 2012. https://doi.org/10.1017/CBO9781107415324.004.
(44) Kissling, W. D.; Carl, G. Spatial Autocorrelation and the Selection of Simultaneous Autoregressive Models. **2008**, 59–71. https://doi.org/10.1111/j.1466-8238.2007.00334.x.
(45) Franceschi, P.; Giordan, M.; Wehrens, R. Multiple Comparisons in Mass-Spectrometry-Based -Omics Technologies. *TrAC Trends Anal. Chem.* **2013**, *50*. https://doi.org/https://doi.org/10.1016/j.trac.2013.04.011.
(46) Fuso Nerini, I.; Morosi, L.; Zucchetti, M.; Ballerini, A.; Giavazzi, R.; D’incalci, M. Intratumor Heterogeneity and Its Impact on Drug Distribution and Sensitivity. *Clin. Pharmacol. Ther.* **2014**, *96* (2), 224–238. https://doi.org/10.1038/clpt.2014.105.
(47) Thompson, C. G.; Bokhart, M. T.; Sykes, C.; Adamson, L.; Fedoriw, Y.; Luciw, P. A.; Muddiman, D. C.; Kashuba, A. D. M.; Rosen, E. P. Mass Spectrometry Imaging Reveals Heterogeneous Efavirenz Distribution within Putative HIV Reservoirs. *Antimicrob. Agents Chemother.* **2015**, *59* (5), 2944–2948. https://doi.org/10.1128/AAC.04952-14.
(48) Creative Commons Attribution 4.0 International License.

Click here to access/download;Figure;Figure1.docx

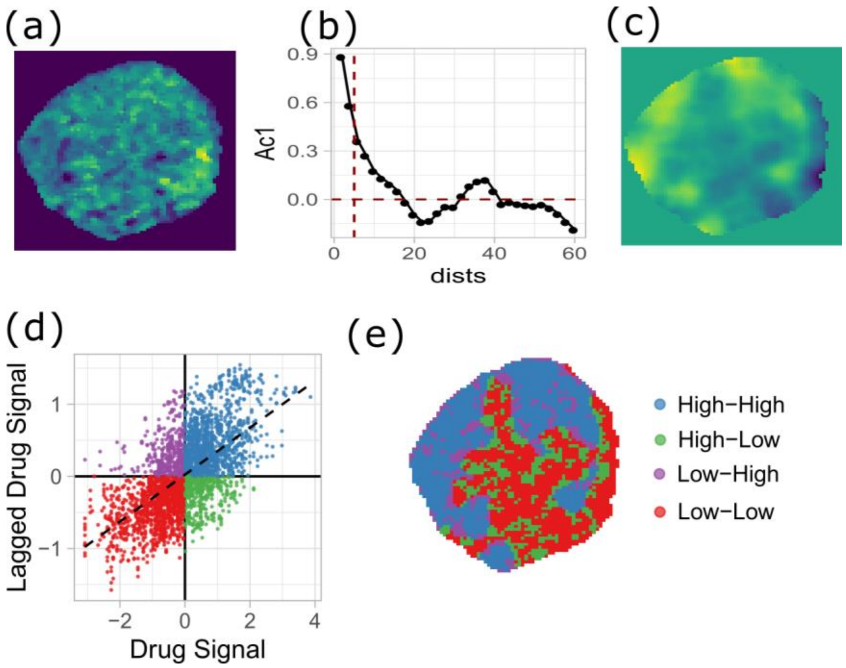

Click here to access/download;Figure;Figure2.docx

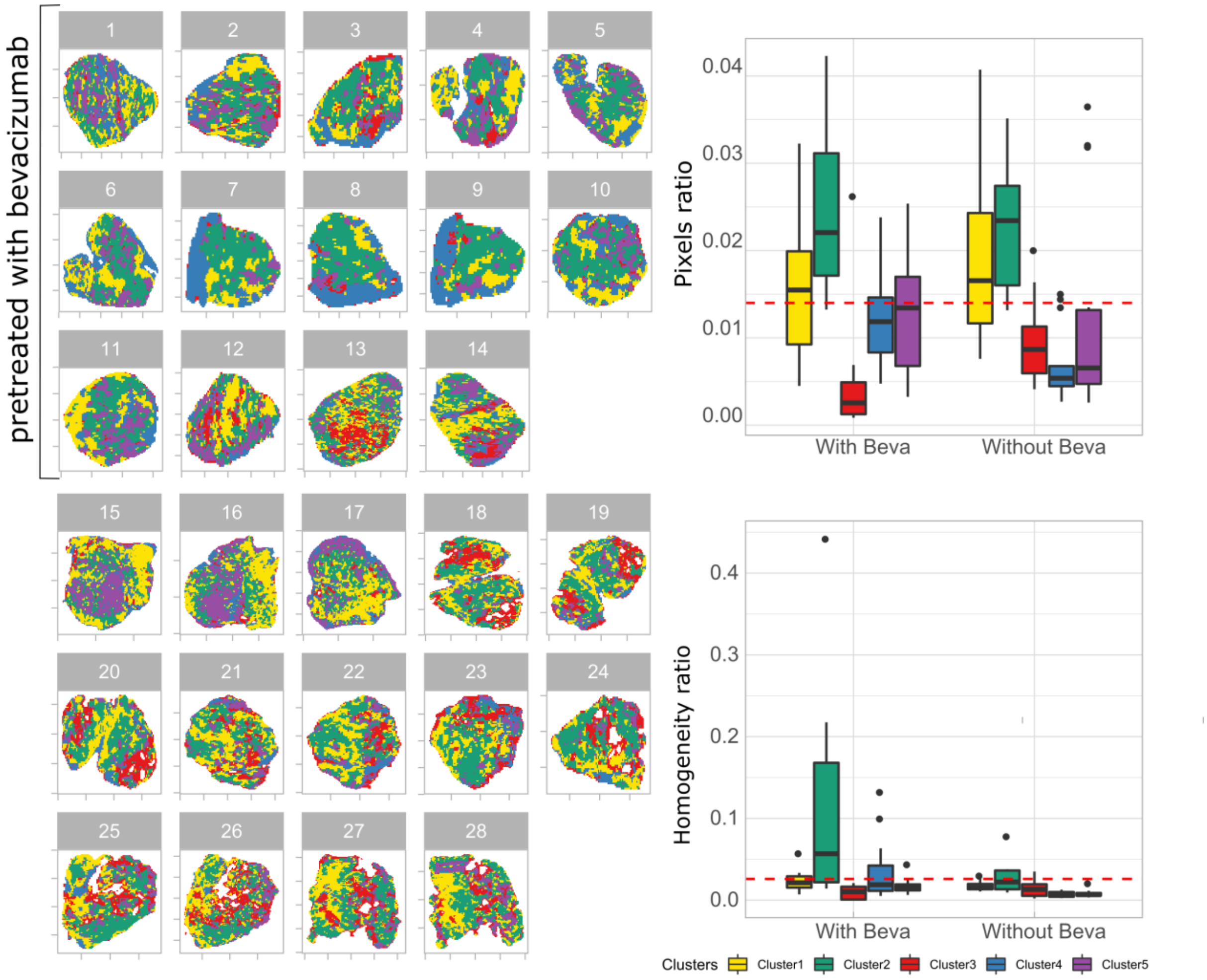

 Click here to access/download;Figure;Figure3.docx

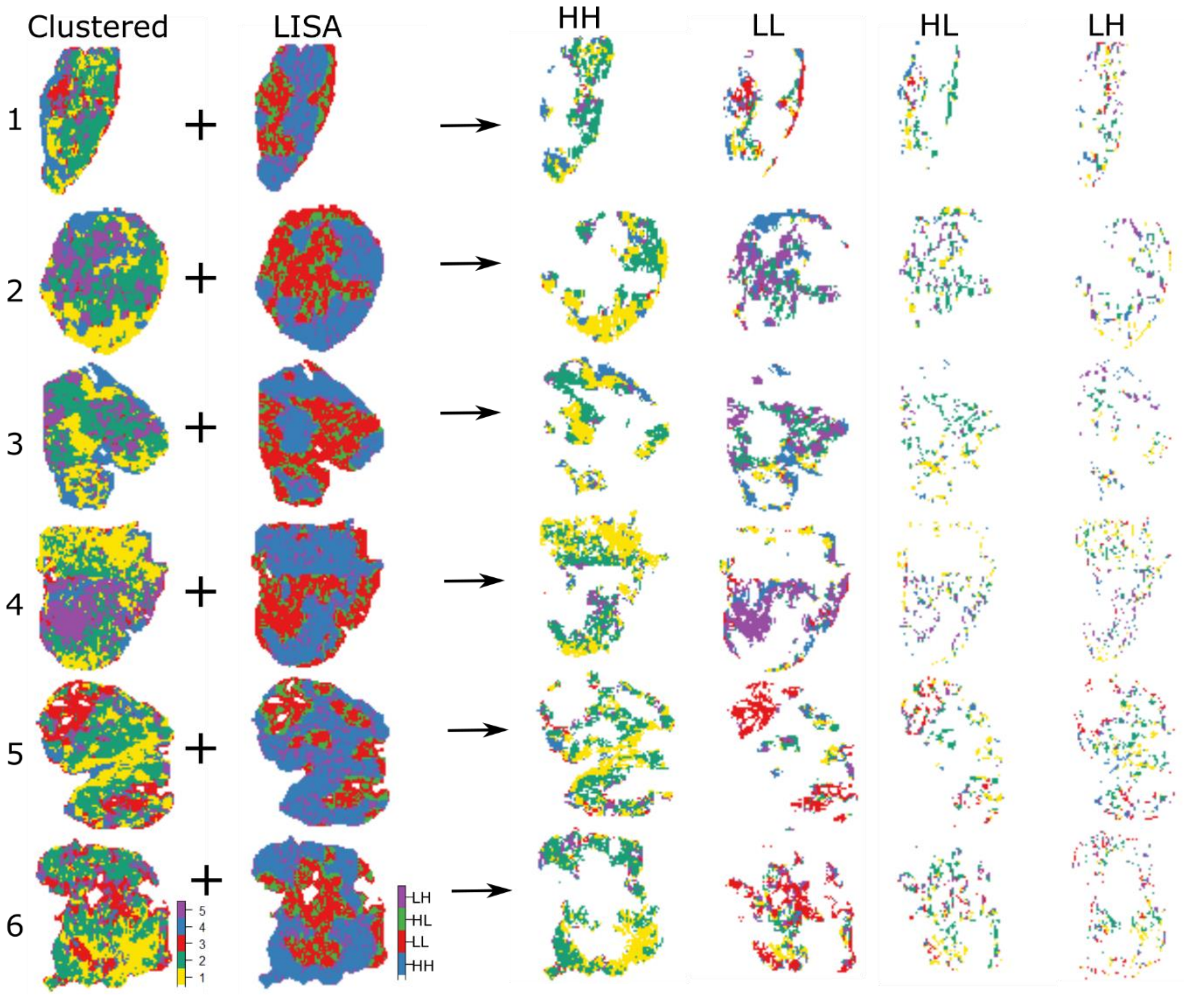

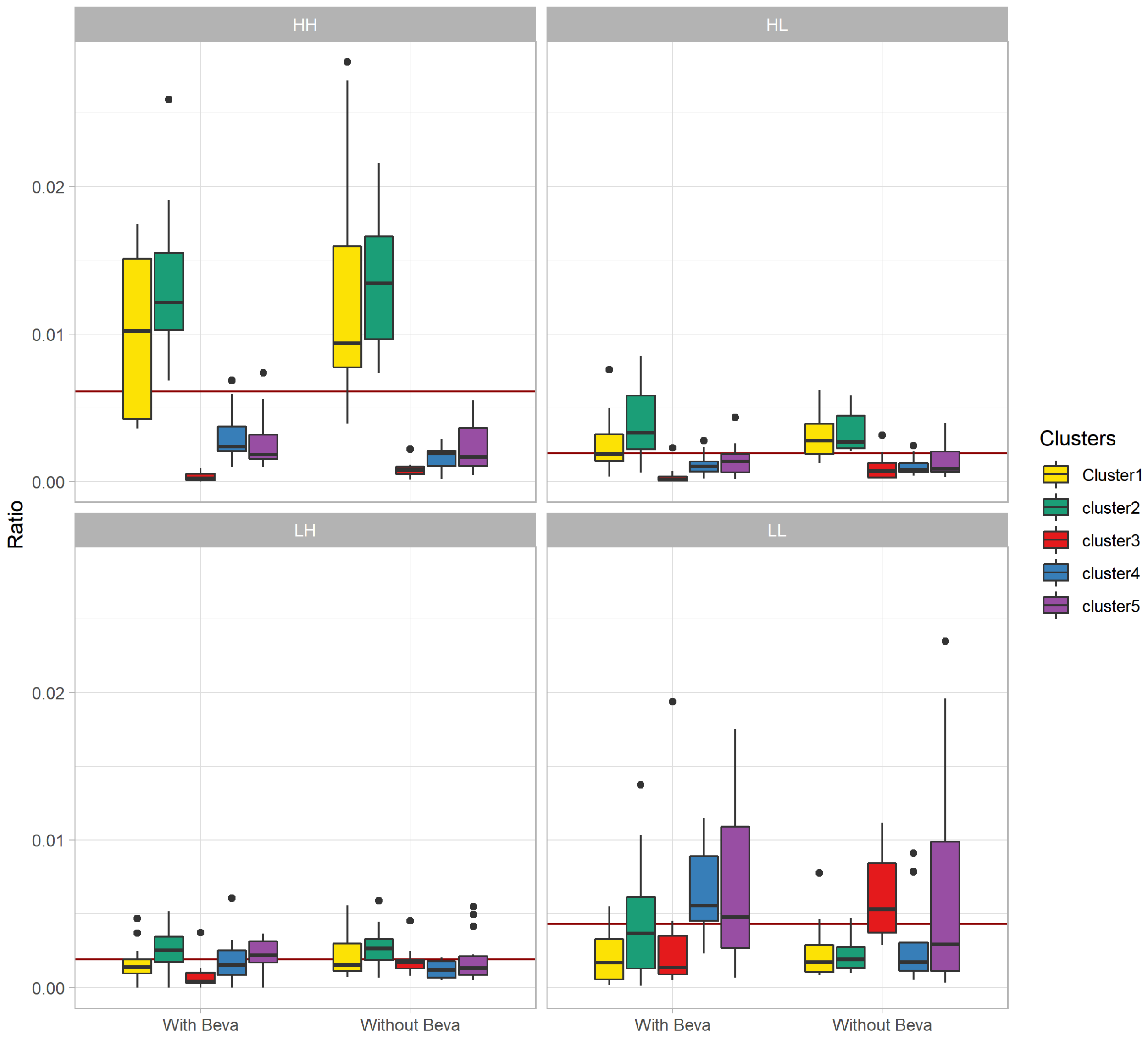
HH
HL
LH
LL
Ratio
0.02
0.01
0.00
With Beva
Without Beva
Clusters
Cluster1
cluster2
cluster3
cluster4
cluster5

Figure
Click here to access/download;Figure;Figure5.docx

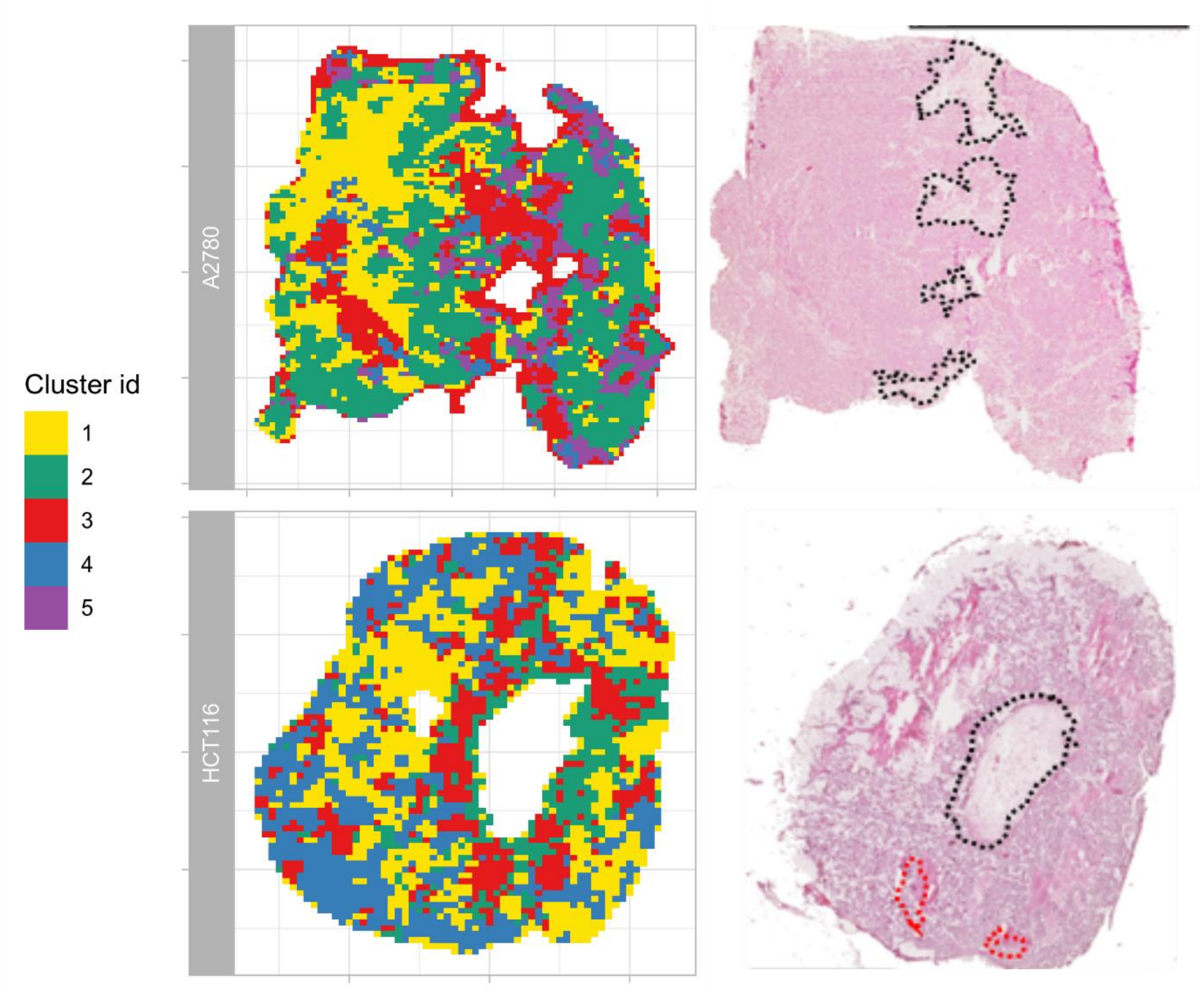

Click here to access/download
Supplementary Material
AdditionalFile1.docx

Click here to access/download
Supplementary Material
AdditionalFile2.docx

Click here to access/download
Supplementary Material
AdditionalFile3.docx

Click here to access/download
Supplementary Material
AdditionalFile4.docx

Click here to access/download
Supplementary Material
AdditionalFile5.docx

Click here to access/download
Supplementary Material
AdditionalFile6.docx

Click here to access/download
Supplementary Material
AdditionalFile7.docx

Click here to access/download
Supplementary Material
AdditionalFile8.docx

Click here to access/download
Supplementary Material
AdditionalFile9.xlsx

Click here to access/download
Supplementary Material
AdditionalFile10.docx

Click here to access/download
Supplementary Material
AdditionalFile 11.xlsx

Personal Cover with response to reviewers including figures Click here to access/download;Personal Cover;EditorLetter.docx

Dear Editor.

We would like to thank the reviewers' for their constructive comments and suggestions. We are including a point-by-point discussion of their comments hereafter. We also revised our manuscript accordingly wherever it was required. The R package from our paper is online available and registered at bio.tools and Scicrunch (bio.tools: corrdrugtumormsi, RRID : SCR_018962). We believe all these changes increase the reproducibility of our paper and now is suitable to publish in the Gigascience journal.

Yours sincerely

Dr. Geert Postma

Reviewer #1:

Question 1.1 The authors present a computational method for identifying spatial regions with molecularly distinct regions between control and drug therapy using previously published data. The method is well described and paper is somewhat easy to follow. The code and attached data was reviewed as well and appears clear and would be easily translatable to other projects. A more formal implementation as an R package would be desirable as the workflow is quite complex it would benefit to make a more accessible API so less experienced users wouldn't get lost.

Answer: A R package is developed that includes all main methods from our paper (bio.tools: corrdrugtumormsi, RRID : SCR_018962). Interested users can install our R-package from the Github website and refer to vignette for the usage of different functions in our package.

Question 1.2 For step 1 in the processing: How are 'drug' related peaks guaranteed to be removed from the 'microenvironment' segmentation? In processing, it is mentioned that ion peaks with correlation > 0.5 with the drug compound were removed. This seems like it would bias the segmentation if the drug had a very discrete distribution in a very particular histological region. One can imagine a scenario where a drug is distributed in area "A" exclusively along with other endogenous compounds. These endogenous compounds would be then be removed from the segmentation pipeline simply because the drug was highly partitioned into this region. Could the peaks be derived solely from undosed control tissue? Otherwise the authors statement may be misleading.

Answer: We agree with the reviewer's comment that the selection of drug-related peaks based on the correlation approach could give biased results. Unfortunately, in our study, we only had one control MSI data set per cell line, and the selection and removal of drug-related peaks based on a single animal also seem quite biased approach. Therefore, we used a heuristic approach where we removed the peaks that show more than 0.5 correlation with drug ion peak and also the peaks present in less than 20% tumor tissue area. In this way, we expected to have not too many ions with discrete distribution and ions with a high correlation with drug ion peak removed. We have validated this approach, see below.

Drug-related peaks in control animal:

The ion intensity distribution of removed peaks in a control tissue from two tumor MSI data (A2780-1A9, HCT116) is shown in Figure 1. Since the control animal has not been treated with drug compound we do not expect those removed peaks to be present in MSI data. But, unfortunately, that is not the case, in the figure, the drug (284.12) and its isotopic peaks (285.155) are completely absent in the control animal and other removed peaks are visible. Therefore, it seems that the threshold of 0.5 we used in our study is quite low and we need a much higher threshold to avoid removing non-drug-related peaks. On using a correlation threshold of 0.9 only the drug isotopic peak is removed from our data, therefore instead of 0.5, we will recommend the minimum correlation threshold of 0.9 in our paper.

Apart from the above analysis, we also checked whether specific peaks which could be considered as drug-related peaks were absent in the control tumor MSI data but present in the treated animal data. We did not notice such peaks. There were few empty bins in the control MSI data but those bins are also empty in the majority of treated animals. This observation was made for both tumor MSI datasets. See added file 'FrequencyofmoelcularIonsInMSIdata.xls' where the count of the pixel with non-zero ion-intensity value across all tumor models is given.

We also looked at the impact of the removed peaks on the segmentation results. An impact of the removed peaks on the segmentation results is expected if the removed peaks are related to a unique spatial structure. The correlation between removed and other remaining peaks was calculated. All removed peaks (except for drug and its isotopic peaks) show a high correlation with many other peaks. Therefore, we don't expect our segmentation results to be biased with our approach. This statement is confirmed by the segmentation of the control tissue. The clustering of control MSI data was performed in both scenarios: with and without drug-related peaks (Figure 2) similar cluster structures were derived.

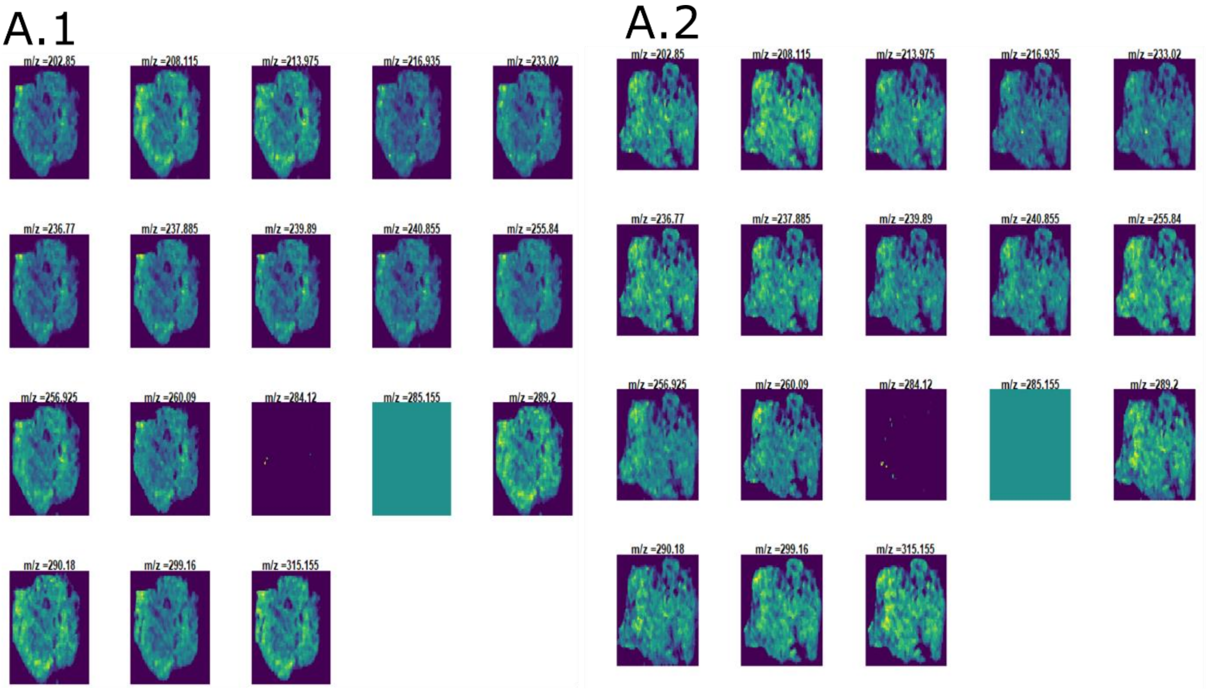

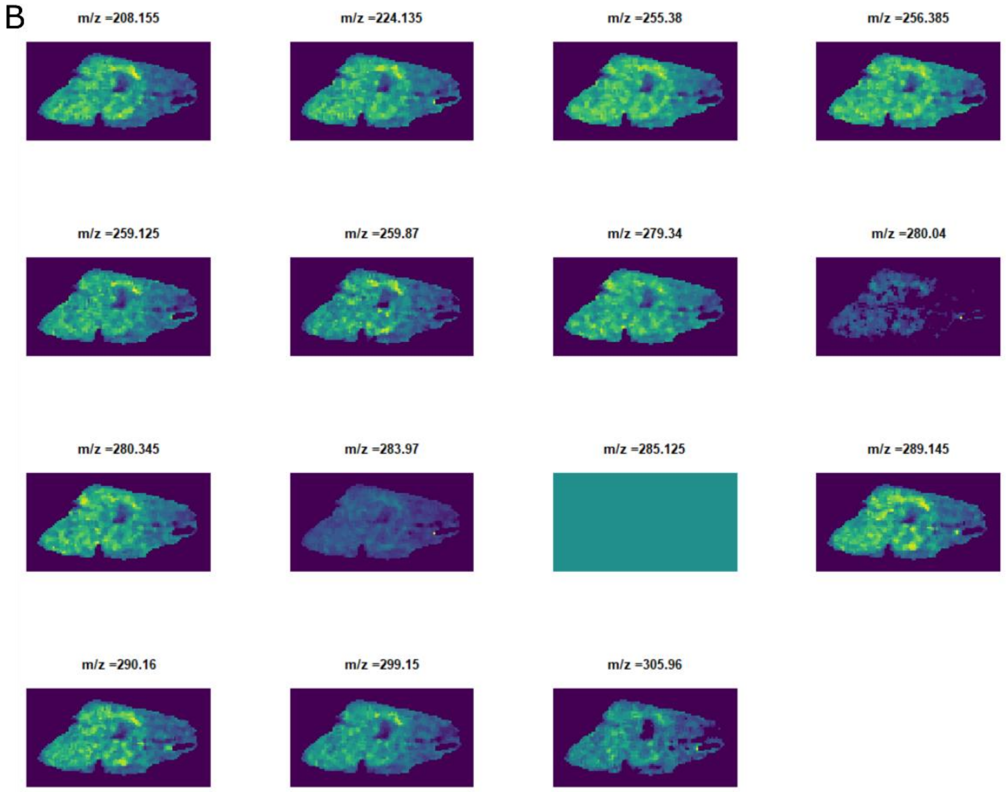

Figure 1: Molecular ion images of peaks removed from our data based on correlation with drug ion peak. A and B: control tumor tissues from A2780-1A9 and HCT116 cell-line respectively (A1, A2: two different slices).

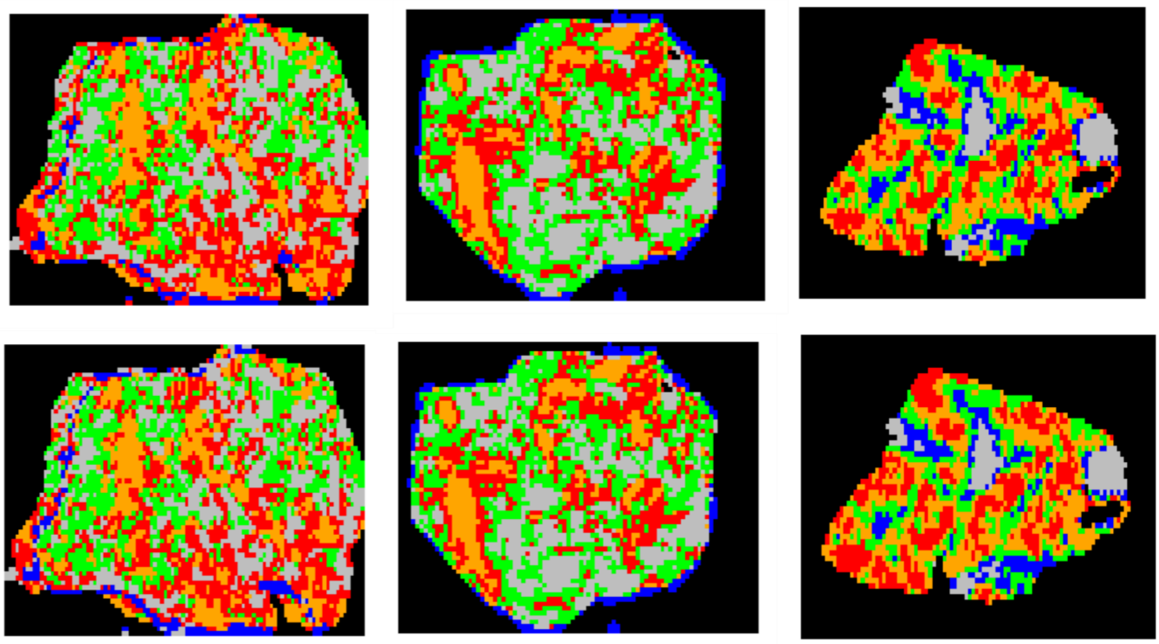

Figure 2: Clustering of control tumor tissue with (Top row) and without (Bottom row) drug correlated peaks.

Based on the analysis on control MSI data we agree with the reviewer that our statement regarding the removal of drug-related peaks based on correlation >0.5 is not sufficient therefore in the absence of control tissue a high correlation threshold (0.9) should be used.

Question 1.3 The authors note that mass spectral validation of model-identified differential ions is not possible and that is reasonable. In general, the spatial models presented in the findings are compelling. However, as this paper deals with spatial characterization of tissue, there appears to be no spatial validation. Indeed the obvious choice of the gold standard in pathology, H&E microscopy, is present in Figure 5 but the size of the images is so small it is negligible for spatial validation. Secondly, there are numerous published MSI examples(DOI: 10.1021/jasms.8b04879, doi:10.1074/mcp.O115.053918, https://doi.org/10.1038/srep36814) where there are clean and distinct, immediate visual association of segmented MSI images to histological regions in H&E, but here the segmentation doesn't seem to replicate much of the structure visible in Figure 5, at least AS PRESENTED. This comment isn't to push for models integrating H&E as an input but to have some qualitative result describing the types of cells present in the tumor regions associated with the major clusters. While molecular histology is valid, it is unusual for it to not mimic classical histology.
Minor comments
All figures containing should have a scale bar indicating the physical dimension of the images.

Answer: We agree with the reviewer's comment that there needs to be more evidence regarding clusters identity and tissue types present in our dataset. However, the MSI data used in this study was initially generated to understand drug homogeneity in different types of solid tumors, and there was little histology-related work done to identify the tissue types. At this stage histological informations available, like proliferation/necrosis, vessels[1] allowed us to correlate only what has been discussed in the text. Future studies will be aimed at the validation of different clustering features presented in this work with more specific, dedicated histological studies.

The reviewer's comment is correct that the optical image in Figure 5 does not show proper correlation with our clustered images, therefore we will remove all images except images 2 and 5 were necrosis region is visible both in the optical image and our clustered images.

Small errors:
Introduction
* Techniques of election <- is not proper English. Perhaps 'A valuable technique' would be less awkward.

Answer: modified accordingly.

Reviewer #2:

This is an interesting paper addressing how MSI can be effectively used to better understand the link between drug and the characteristics of malignant tissue. While it is not surprising that the physicochemical properties of both the tissue and the drug are important to passive tumor penetration and local exposure, MSI provides an important opportunity to understand the spatial and temporal dynamics of this process, and the development of effective computational workflows is vital. A few questions/suggestions for the authors follow:

Question 2.1 To what extent can the most prominent histochemical changes occurring post-bevacizumab treatment be captured by m/z range and other experimental settings studied in this untargeted MSI experiment? Since it used a limited mass range, certain important changes (e.g., in cell-surface protein expression, lipid membrane composition, etc.) may not have been measured. The authors may have addressed this question in their cited previous work, but it would be helpful to provide some additional context.

Answer: We agree with the reviewer, our MSI data cover a limited mass range where we cannot see certain changes. However, the MSI data used in this study was initially generated to understand drug homogeneity in different types of solid tumors, and there was little histology-related work done to identify the tissue types. Our major focus, at this stage was not in a comprehensive metabolite study, but in methodology development. Future studies will be focussed, with more specific histology, on local metabolic effects of drugs and larger mass range will be used

Question 2.2 Since the focus of this paper is methodology, evaluation of the approach against known ground-truths is critical. In that regard, the efforts of the authors in developing a synthetic dataset and evaluating the methods on it is appreciated. There are a few ways that this assessment could be expanded to provide additional information about the robustness of the workflow. For example, Additional File 3 includes plots showing the synthetic data and some of its characteristics, but to what extent do the statistical properties of the synthetic data compare with those of real MSI datasets? The SL method was recommended, but how sensitive is it to the selection of the weight matrix? If it is sensitive, are there any recommendations for selecting the weight matrix based on data characteristics? When bridging to the experimental data, has the method been tested on MSI datasets (including synthetic ones) with available complementary ground-truth labeling to help evaluate the extent to which identified clusters map to known differences? It is mentioned that peaks that were present in less than 20% of the tissue were removed to focus on more common ions. To place this 20% cutoff in context, what was the coverage area of the clusters identified? It seems possible that this step may omit significant portions of tissue heterogeneity. For future applications of this workflow, how should this cutoff threshold be selected? Overall, how robust are the results/workflow recommendations to the choices of distance metric and clustering index?

Answer:

a) MSI data is a type of spatial data where nearby observations are highly correlated with each other. In our study, we had used the spatial autocorrelation function to generate similar spatially autocorrelated synthetic data. In additional File 3, a spatial correlogram plot from our synthetic data is presented, which is quite comparable with the spatial correlogram of drug molecular ions from different tumor MSI data shown in Additional File 5.

b) Yes, the results from spatial methods depend upon the selection of the spatial weight matrix. We performed a small simulation study with MSI data. And, we included our conclusion and recommendation in the discussion section, page 17.
c) In our study, we don't have ground-truth labeling. Therefore it was not possible to completely validate the identify clusters. The validation of identified clusters was performed with H&E stained images shown in Additional File 5 where the necrosis tissue present in the optical image shows similarity with clusters in the A2780-1A9 and HCT116 data.
d) In our study, we discarded peaks with less than 20% coverage area, assuming that they represent the noisy peaks and could influence our clustering algorithm. The decision of discarding those peaks was based on multiple trials. For the removal of peaks, we tried a threshold of 10%, 20%, and 30%. With a threshold of 10%, we missed some noisy peaks and with 30% we excluded a few extra peaks in our data. Therefore, a threshold of 20% seems a reasonable choice. Moreover, this step of peak removal was performed on each tumor MSI data separately. Therefore, unless particular peaks were present as noise in all datasets, we do not expect them to be completely removed from our input data for the clustering.

   The range of different clusters size derived across all tumor models is shown in Table 1 below. In our MSI data, we have identified the clusters of size smaller than 20% of total tumor tissue area which means after removing a fraction of peaks (which we assume noise-related) we are still able to find spatially relevant smaller clusters.

   Table 1. Range of different clusters size in two tumor MSI data.

		Cluster 1	Cluster 2	Cluster 3	Cluster 4	Cluster 5
A2780	Minimum size	212	624	40	224	153
	Maximum size	3429	2959	1691	1270	3078
HCT116	Minimum size	337	734	558	208	186
	Maximum size	833	1674	1297	682	605

Question 2.3 Given the dose of the drug administered, how much exposure within the tumor is expected based on pharmacology, and how might this affect the output? It would also help to provide more explanation of Figure S1 in Additional File 7. In it, the concentration (units undefined) of the drug in each cluster appears to be very similar, across both cell lines and treatment arms; however, the comparison between the clusters and the LISA maps appears to suggest differently. Also, interpretation of the LISA maps of drug exposure in Figure 3 and the data in Table 1 in terms of histology is briefly in the Discussion, but it would be helpful to expand on this.

Answer: For drug MSI data generation, the solid tumors were explanted from mice 6 hours after drug treatment. Therefore we do not expect dramatic changes in tissue architecture due to the drug compound. Moreover, all tumor models have received drug treatment, so if any metabolic change occurs, we expect it to be consistent across all tumor MSI data.

Additional File 7, Figure 1, shows the average amount of drug in each cluster-type under two treatment conditions. The drug average value in each cluster is further normalized with the

sum of the average drug in a particular treatment condition. Note, this second step is performed simply to show the drug concentration in the range of 0-1. Apart from that the average drug concentration profile from steps 1 and 2 is similar.

References

1. Cesca, M.; Morosi, L.; Berndt, A.; Fuso Nerini, I.; Frapolli, R.; Richter, P.; Decio, A.; Dirsch, O.; Micotti, E.; Giordano, S.; et al. Bevacizumab-Induced Inhibition of Angiogenesis Promotes a More Homogeneous Intratumoral Distribution of Paclitaxel, Improving the Antitumor Response. *Mol. Cancer Ther.* **2016**, *15* (1), 125–135. https://doi.org/10.1158/1535-7163.MCT-15-0063.

Personal Cover : table added as proof for reviewers Click here to access/download;Personal Cover;Fequency ofmolecularIonsInMSIdata.xlsx

	X199.945	X200.165	X200.855	X201.145	X201.935	X202.15	X203.145
A2780_CTRL	3525	3124	3550	3085	3592	3019	2853
A2780_CTRL3	4484	4049	4611	4091	4724	3948	3891
./A2780_AVA #304 1	5297	5211	5311	5145	5309	5217	4774
./A2780_AVA #304 2	2254	2217	2263	2200	2259	2213	2134
./A2780_AVA #304 3	2032	1978	2044	1900	2018	1988	1835
./A2780_AVA #315 1	3392	3393	3394	3377	3394	3387	3375
./A2780_AVA #315 2	4460	4428	4460	4331	4460	4454	4359
./A2780_AVA #315 3	5585	5515	5593	5061	5594	5565	4624
./A2780_AVA #326 1	2585	2557	2583	2560	2583	2561	2523
./A2780_AVA #326 2	1816	1793	1816	1806	1814	1801	1796
./A2780_AVA #326 3	1738	1733	1739	1735	1736	1723	1728
./A2780_AVA #335 2	3274	3272	3276	3270	3275	3269	3248
./A2780_AVA #335 3	3243	3204	3246	3128	3246	3196	3054
./A2780_AVA #372 1	2447	2390	2469	2349	2470	2407	2176
./A2780_AVA #372 2	5861	3949	6065	3814	6066	4674	3396
./A2780_AVA #372 3	2763	2801	2836	2791	2836	2804	2721
./A2780_PTX #302 1	9391	8712	9525	7623	9631	8752	7568
./A2780_PTX #302 2	10364	10511	10471	10110	10607	10445	10241
./A2780_PTX #302 3	10743	9642	10793	8369	10815	10412	7708
./A2780_PTX #321 1	4976	3843	5405	3931	5469	3931	3153
./A2780_PTX #321 2	2908	3178	3269	3189	3368	3107	3064
./A2780_PTX #321 3	3224	3647	3663	3638	3818	3497	3420
./A2780_PTX #349 1	5673	4068	5703	3915	5739	4149	2932
./A2780_PTX #349 2	3550	3189	3594	3045	3620	3130	2406
./A2780_PTX #349 3	3845	3373	3945	3200	3972	3587	2805
./A2780_PTX #352 1	2261	2305	2597	2360	2685	2192	2062
./A2780_PTX #352 2	3310	3677	3795	3770	3915	3631	3536
./A2780_PTX #352 3	6707	7115	7428	7169	7656	6847	6927
./A2780_PTX #371 2	5338	4921	5468	4637	5585	5003	3904
./A2780_PTX #371 3	6526	5791	6613	5841	6728	5885	4890

Number of pixels with non-zero value per MSI data

X203.845	X204.145	X204.86	X205.155	X205.815	X206.01	X206.25	X207.04	X208.97
3032	3302	3295	2420	2399	2687	3157	3676	2990
3891	4219	4043	3493	3010	3392	4090	4760	4079
4906	5260	5038	4592	3937	4935	5255	5311	3747
1819	2244	1864	2113	1016	2180	2235	2271	1527
1471	1997	1891	1579	0	1909	1982	2050	1338
3076	3388	3392	3376	574	3092	3386	3394	3257
3935	4425	4458	4235	1279	3370	4405	4460	4451
5374	5409	5571	3957	4596	5122	5501	5594	3980
2535	2571	2582	2487	2230	2545	2565	2588	2186
1619	1798	1788	1776	824	1760	1810	1819	1311
1340	1729	1707	1721	317	1692	1736	1739	1197
3081	3271	3248	3250	2015	3233	3274	3278	2196
3081	3220	3219	2950	1058	3100	3206	3246	1832
1990	2394	1803	2113	630	2344	2424	2470	1340
5454	4467	5535	2587	4387	5313	4916	6051	4597
1778	2796	2578	2640	0	2657	2820	2836	2159
6713	8813	9375	6447	3378	3930	8861	9625	9377
2251	10501	10167	9477	0	1844	10163	10618	10368
7577	9741	10207	5832	7037	2712	10167	10812	8769
4555	4236	4019	2648	3167	2417	4660	5302	3212
2230	3158	2464	2952	0	1744	3202	3319	1906
2451	3562	2971	3274	807	2109	3619	3798	3066
5427	4639	5448	1602	4536	4865	4918	5734	4835
2865	3254	2992	1741	2217	2003	3358	3621	1755
3037	3411	3146	1806	2105	2087	3596	3954	1708
1587	2203	1650	1874	530	1087	2363	2588	1748
1415	3578	2102	3570	0	1605	3719	3890	909
5649	6887	6175	6713	1708	4591	7160	7581	5028
4014	5002	4679	3437	0	3567	5071	5560	1771
5838	6084	5875	4459	4254	5573	6056	6717	4855

X209.2	X209.9	X210.195	X210.995	X211.17	X211.955	X212.765	X212.97	X213.165
3398	2021	3426	1786	2663	3673	3562	3102	2296
4330	1980	4476	2097	3887	4687	3545	3847	3300
5295	0	5305	1091	5010	5308	936	5046	4448
2258	0	2266	0	2190	2266	0	2063	1903
2022	0	2028	216	1862	2033	90	1471	1421
3349	3380	3372	644	3364	3392	836	3356	3369
3857	4448	3423	947	4209	4449	849	4443	4305
5568	4836	5575	0	3552	5571	0	5542	3755
2582	2011	2586	382	2532	2587	1763	2550	2370
1814	938	1819	0	1801	1819	508	1757	1731
1739	839	1739	374	1732	1737	823	1644	1693
3278	1373	3278	0	3267	3278	592	3180	3214
3241	1181	3245	602	3078	3243	838	3211	2907
2470	0	2470	0	2314	2466	0	1718	2003
5678	0	5890	1291	3416	5847	0	5070	2190
2834	0	2835	900	2760	2833	0	2010	2562
7891	9026	7389	1861	6523	9486	3620	9114	6952
8295	10410	6648	0	9876	10610	0	10174	10051
10513	7926	10633	0	5658	10653	3447	10446	7035
5115	0	5213	0	3301	5251	1716	3646	2358
3323	0	3344	0	3120	3355	586	2018	2843
3717	652	3781	933	3507	3813	390	2810	3085
5613	3077	5691	1617	2347	5692	5410	5445	1066
3577	1050	3608	0	2452	3612	2703	3315	1683
3885	661	3926	0	2652	3935	3129	3393	1617
2536	0	2599	0	2180	2604	710	1599	1794
3874	497	3917	0	3634	3904	905	1711	3412
7446	1835	7599	1239	7001	7601	1155	4913	6379
5413	979	5527	0	3832	5507	1874	4294	2847
6544	3026	6634	0	5173	6650	2366	5735	4179

X214.895	X215.89	X216.17	X217.8	X218.165	X219.115	X220.015	X220.185	X220.86
3730	3302	1680	1671	1135	1067	2679	1035	2775
4802	4026	2117	1977	2139	1755	2927	1982	3243
5309	5218	2557	2135	1305	0	4304	1508	4583
2271	2221	1499	261	867	332	2090	742	1170
2050	1926	1039	0	467	296	1613	415	383
3394	3388	3338	1262	2760	2405	3216	2944	2889
4460	4438	4369	1583	701	0	3985	962	4355
5594	5565	4350	2466	0	0	5035	0	5438
2586	2570	1922	1724	874	1023	2479	860	2433
1820	1802	1422	649	942	818	1696	985	1135
1739	1728	1518	529	1130	909	1621	1197	777
3278	3266	3079	631	1851	787	3214	1549	2251
3246	3230	2943	938	787	0	3117	761	2537
2470	2356	1081	0	677	308	1877	643	842
6065	5676	1383	3193	0	0	4301	0	4902
2836	2598	2091	702	1404	677	2543	1234	477
9616	8694	6541	1891	2436	2030	4014	2433	8394
10605	9108	10019	0	5626	5228	4976	4188	8054
10813	9725	6364	0	0	0	2238	0	9894
5384	4187	0	0	0	0	893	0	3622
3325	2173	1235	0	1674	822	1660	1220	575
3769	2655	1713	0	1694	837	2277	1195	1005
5732	5338	0	2252	0	0	3941	0	5175
3615	3117	0	0	0	0	1946	0	3016
3961	3226	808	0	0	0	2027	0	2885
2635	1555	590	0	661	316	0	694	863
3883	1943	2039	0	2286	1098	1170	1976	0
7583	4798	3343	0	3802	2347	2908	3161	1423
5546	4158	1639	0	0	0	1649	0	2534
6700	5703	1681	1727	0	950	3121	0	4804

X221.195	X221.875	X222.015	X222.215	X222.89	X223.025	X223.17	X223.89	X224.195
3516	3445	1389	2532	3505	3061	2096	2136	3580
4555	4387	1483	3769	4540	3698	3439	2907	4696
5164	5301	1044	4834	5308	5118	4479	3125	5309
2265	2239	800	2177	2266	2204	1980	412	2270
2031	1989	358	1819	2036	1884	1621	0	2051
3385	3375	0	3349	3394	3171	3218	1658	3394
4446	4454	931	4099	4460	4417	2333	2597	4456
5509	5574	2267	3730	5594	5466	1796	3618	5594
2576	2573	1252	2381	2586	2544	2102	1690	2588
1817	1811	667	1787	1820	1763	1736	270	1820
1737	1734	496	1720	1739	1648	1716	0	1739
3277	3267	1208	3262	3273	3156	3174	536	3278
3240	3242	1175	2952	3246	3169	2682	523	3246
2327	2462	293	2246	2469	2243	1995	428	2470
4530	5996	1538	3640	6068	5231	2619	3955	6060
2821	2779	455	2758	2836	2344	2639	0	2833
9347	9271	0	7009	9558	8973	2255	6889	9629
10567	10210	0	9178	10533	9369	5493	4508	10623
10473	10762	0	6931	10802	10190	2991	6162	10814
5045	5175	0	3380	5436	4473	2367	3190	5397
3291	3127	0	3114	3320	2502	2911	0	3384
3759	3420	0	3502	3758	2777	3157	559	3862
5511	5665	0	2276	5725	5329	0	4535	5736
3566	3558	0	2125	3603	3279	1221	1401	3624
3865	3901	0	2369	3949	3486	1406	1387	3964
2510	2411	0	2223	2653	1863	1934	552	2666
3838	3535	0	3630	3878	2559	3414	0	3941
7439	6773	0	6896	7521	5058	6429	0	7643
5451	5271	0	3709	5508	4278	2342	0	5585
6486	6275	1185	4462	6536	5462	3092	3599	6714

X224.92	X225.15	X225.87	X226.195	X226.98	X227.12	X227.32	X227.845	X228.165
2902	3150	3610	3272	2107	3045	3562	1267	1033
3211	4391	4536	4329	2225	4114	4547	1343	1516
4336	5276	5294	5280	1977	5224	5308	891	743
1828	2265	2270	2257	565	2218	2264	280	0
833	2047	2042	2004	0	1943	2020	0	182
3387	3392	3394	3380	2042	3359	3361	2819	2702
4460	4459	4460	4268	3582	3523	4434	3654	2176
5579	5543	5594	5361	4776	4248	5535	3869	0
2357	2587	2588	2581	1611	2555	2586	1228	669
1330	1819	1820	1816	532	797	1820	415	539
1344	1739	1739	1736	771	1733	1739	462	795
2899	3277	3278	3276	1197	779	3278	825	787
3097	3218	3246	3233	1836	3183	3246	862	627
639	2463	2435	2458	0	2400	2470	0	0
3607	5834	5919	4911	1380	4009	6040	0	0
624	2832	2805	2828	0	2788	2830	0	757
9333	9229	9628	8095	5391	5933	9585	6014	3793
10242	10527	10618	10183	1907	8699	10586	3971	7169
10582	9816	10810	9208	4745	6158	10776	3189	0
1889	4901	5161	4653	0	4046	5363	0	0
1008	3282	3150	3282	0	3190	3323	0	481
1940	3714	3661	3706	745	3541	3766	0	590
5315	5223	5735	4788	2559	3665	5672	0	0
3174	3462	3613	3437	530	3020	3610	0	0
2990	3740	3949	3653	832	3207	3937	0	0
1074	2443	2562	2483	0	2309	2607	0	0
1368	3808	3795	3851	0	3707	3884	0	936
2422	7311	7390	7415	0	7160	7500	0	1200
3744	5141	5524	5272	0	4744	5498	0	0
5456	6020	6676	6313	2647	0	6655	970	0

X228.985	X229.89	X230.19	X230.93	X231.965	X236.025	X236.2	X237.155	X238.145
2930	2201	1453	3567	1994	1911	980	587	2858
3374	2583	1975	4437	2203	2521	2180	1538	3978
5047	3472	2203	5301	2253	3796	1734	1014	5230
2143	1506	732	2260	712	1728	785	605	2229
1604	782	374	2025	420	774	449	210	1946
3295	3198	3287	3384	1204	2923	2109	0	3357
4400	4339	4083	4451	776	3676	0	0	3593
5458	5289	2278	5588	2066	3880	0	0	4711
2532	2294	1758	2584	1600	2187	832	0	2549
1750	1349	1394	1814	875	1362	985	734	1810
1660	1195	1475	1736	796	1229	1098	882	1735
3186	2563	2677	3270	1334	2677	1454	1011	3268
3178	2583	2395	3240	1360	2375	748	0	3188
1917	735	420	2458	479	773	544	463	2411
4845	2856	0	5954	2070	2374	0	0	4153
2100	1238	1434	2778	765	1464	1364	701	2769
7968	8643	4641	9301	2601	8144	0	0	6710
7111	9450	8346	10238	0	9181	1783	0	8310
9065	9350	3509	10719	1973	8384	0	0	7238
2970	849	0	4981	0	841	0	0	3953
1668	831	1112	2941	402	1189	1398	577	3184
2244	1509	1306	3297	851	1743	1212	548	3553
4661	3629	0	5646	2839	2977	0	0	3528
2570	1539	0	3557	709	1762	0	0	3021
2394	1365	0	3823	662	1225	0	0	3101
882	462	0	2299	274	813	915	317	2316
1250	906	1980	3212	0	1508	2023	1201	3717
2600	2435	3275	6441	1377	2873	3118	1261	7184
3013	1667	0	5230	631	1407	0	0	4425
5097	4039	0	6458	3259	3611	0	0	5499

X238.83	X239.075	X239.185	X240.155	X241.1	X241.915	X242.135	X242.97	X243.19
3676	535	0	2383	3632	3709	3290	1271	2129
4759	1928	0	3689	4732	4785	4170	1924	2226
5311	0	1162	4970	5311	5311	5292	1939	1872
2271	478	854	2141	2271	2271	2245	501	1492
2051	1129	0	1743	2051	2050	2004	0	894
3394	410	0	3283	3394	3394	3274	2668	2554
4460	0	0	1527	4460	4460	4201	4340	675
5594	0	0	2458	5594	5594	5548	4967	1220
2588	0	815	2347	2588	2588	2568	1482	1490
1820	347	1400	1794	1820	1820	1801	730	1259
1739	954	1274	1728	1739	1739	1734	672	1167
3278	0	1934	3252	3278	3277	3265	1469	2332
3246	1139	0	2962	3246	3246	3228	1801	1479
2470	274	550	2293	2470	2470	2455	0	1135
6072	0	0	2855	6073	6066	5851	1941	1925
2836	1521	747	2692	2836	2836	2812	605	1639
9646	0	0	3259	9646	9632	9495	6458	3882
10624	0	0	5724	10629	10615	10526	4437	5849
10818	0	0	3644	10820	10812	10765	2853	3695
5560	0	0	2629	5561	5323	5032	0	0
3411	0	935	3120	3419	3307	3196	0	1641
3894	1038	0	3224	3901	3755	3598	0	1276
5741	0	0	1334	5741	5732	5599	0	1012
3639	626	0	1812	3639	3619	3549	0	869
3988	0	0	1683	3988	3948	3810	0	880
2731	923	0	2090	2734	2619	2454	0	935
3969	1087	1478	3603	3977	3846	3692	0	2421
7746	0	1154	6700	7749	7502	7021	0	3220
5609	1823	0	3096	5608	5538	5336	0	1393
6771	0	0	4472	6776	6703	6305	0	2253

X243.885	X245.95	X247.955	X251.935	X252.21	X252.925	X253.255	X254.305	X254.85
1858	0	2637	3509	1980	1506	3656	2992	3564
1446	0	3021	4370	3245	1121	4705	3753	4509
0	0	2833	5308	4483	0	5309	4835	5288
517	0	2028	2258	1908	0	2271	1862	2218
0	0	1279	2036	1231	0	2046	1324	1938
3376	2152	3137	3393	2823	0	3392	3168	3265
4451	4078	2779	4460	0	2030	4460	4194	4412
5493	4601	5169	5507	1450	2625	5594	5321	5587
1899	453	2488	2563	1652	0	2588	2473	2587
935	297	1734	1810	1675	0	1820	1771	1815
881	278	1644	1718	1659	0	1739	1694	1721
2295	0	3112	3275	3109	0	3278	3228	3271
2588	0	2758	3244	2495	0	3246	3187	3228
0	0	1169	2435	1795	0	2470	1482	2430
0	0	2763	5579	1892	0	6073	3646	5971
0	0	1815	2795	2435	0	2836	2085	2300
8303	3481	4768	8795	0	4572	9646	9224	9468
8506	2348	3946	9993	2231	0	10624	9999	8952
7245	7896	5546	10075	0	4791	10817	10441	10753
0	0	1115	4334	1769	0	5539	4584	5214
0	0	1843	2892	2736	0	3414	3058	2985
0	0	2305	3187	2476	0	3884	3242	3066
1319	0	4421	4992	0	1292	5741	5027	5712
683	0	2428	3222	980	0	3639	3400	3570
0	0	2258	3165	810	0	3985	3595	3884
0	0	801	1773	1817	0	2725	2200	2383
0	0	2015	3048	3405	0	3964	3505	3308
0	0	3729	5537	5565	0	7713	5907	6604
1420	0	2799	4582	1632	0	5601	4554	5314
2484	0	5236	5786	2664	1346	6747	5017	6482

X255.035	X255.27	X255.505	X255.68	X255.995	X256.145	X256.35	X257.835	X258.005
3149	3678	1176	0	0	686	3570	2615	1341
3888	4828	1013	0	0	1045	4704	3442	1323
5249	5311	0	0	0	0	5309	2448	1372
2241	2271	0	0	0	0	2271	365	459
1987	2051	0	0	0	0	2049	0	0
3335	3394	0	0	0	1356	3394	0	0
4433	4460	1550	973	1938	755	4460	0	0
5568	5594	2572	0	2146	0	5594	1480	2378
2514	2588	0	0	0	0	2588	1974	400
1749	1820	0	0	0	0	1820	1009	219
1698	1739	0	0	0	350	1739	389	0
3228	3278	0	0	0	583	3278	1762	856
3240	3246	0	0	0	0	3246	0	761
2331	2470	0	0	0	0	2470	1393	0
5556	6073	0	0	0	0	6064	4761	1091
2697	2836	0	0	0	0	2836	0	0
9336	9646	5638	3921	3971	2118	9644	0	0
10238	10629	4175	2557	2448	3417	10625	0	0
10633	10820	5303	2488	3806	0	10816	2216	0
4580	5568	0	0	0	0	5509	4507	0
2658	3424	0	0	0	0	3393	756	0
3240	3901	0	0	0	0	3863	0	0
5422	5741	0	0	0	0	5741	5242	0
3417	3639	0	0	0	0	3637	1943	0
3739	3991	0	0	0	0	3980	1898	0
2267	2739	0	0	0	0	2722	1059	0
3174	3979	0	0	0	0	3960	0	0
5235	7756	0	0	0	0	7708	3143	0
4729	5609	0	0	0	0	5584	1319	0
5454	6776	0	0	0	0	6744	5663	0

X258.865	X259.115	X260.905	X262.025	X264.04	X264.9	X266.235	X267.235	X268.035
2220	3606	3039	945	1328	1818	1358	0	0
2908	4714	3446	1198	807	1695	2686	0	0
1525	5309	5090	0	1783	3681	3443	1678	0
0	2271	2060	675	1715	720	1371	604	0
0	2050	892	745	991	0	864	0	357
0	3394	3312	0	1451	758	2101	0	0
0	4460	4454	4449	1708	3408	0	0	3352
1068	5594	5565	5198	4104	5116	0	0	0
1630	2588	2297	0	1870	1155	1010	641	0
438	1819	1223	0	1530	0	1392	1087	0
0	1739	1057	0	1497	0	1416	1139	0
0	3278	2602	0	2654	605	2592	1597	0
0	3246	3007	0	1802	1363	1589	593	0
0	2470	1227	414	666	0	1200	668	0
2564	6065	5479	1452	1631	2015	0	0	0
0	2836	1118	1423	1569	0	1826	586	2323
0	9612	9399	0	0	6460	0	0	0
0	10607	10225	0	0	3584	0	0	0
2681	10807	10720	9850	0	8325	0	0	2066
2919	5381	4430	0	0	853	0	0	0
0	3372	1863	0	684	0	2086	631	0
0	3816	2696	0	991	0	1409	0	0
4899	5729	5563	0	0	3360	0	0	0
1261	3625	3506	0	0	1854	0	0	0
644	3969	3747	1731	0	1118	0	0	837
0	2645	2174	491	0	0	1256	0	0
0	3919	2637	0	0	0	2770	1564	825
0	7587	3898	0	0	0	3379	0	0
0	5555	4640	0	0	0	0	0	0
4622	6685	5685	0	1281	3053	1386	0	0

X269.385	X270.86	X276.075	X276.82	X278.94	X279.245	X279.875	X280.29	X281.06
0	1897	2020	3484	1688	3624	2392	3514	0
0	1659	2470	4334	1632	4744	2606	4523	0
1854	3121	2246	5305	3351	5311	2977	5308	0
618	1222	793	2262	407	2271	862	2268	0
0	813	304	2001	0	2051	0	2037	0
0	1287	973	3393	0	3394	1047	3388	0
0	3906	2846	4460	2746	4460	3156	4460	1677
0	5339	3831	5587	4832	5594	3953	5594	2345
0	1169	1002	2580	678	2588	1451	2588	0
534	750	0	1814	0	1820	848	1820	0
542	1239	0	1727	0	1739	715	1739	0
0	993	810	3266	0	3278	863	3276	0
0	2459	1474	3245	887	3246	1007	3246	0
453	404	339	2464	0	2470	0	2470	0
0	1841	0	6033	2148	6073	1355	6066	0
0	1344	0	2803	0	2836	0	2836	0
0	7392	4221	9495	6200	9646	8341	9641	5802
0	2601	0	10398	3200	10629	7524	10627	3298
0	7993	5516	10775	7598	10820	9827	10818	5457
0	0	2044	5099	0	5568	1782	5480	0
0	0	680	2858	0	3424	812	3395	0
0	2248	921	3268	0	3901	633	3829	0
0	1042	2398	5644	3235	5741	3950	5740	0
0	0	1223	3546	1442	3639	2706	3628	0
0	1379	1014	3834	929	3991	2361	3982	0
0	346	0	2330	0	2739	1052	2713	0
0	0	0	3242	0	3979	1089	3951	0
0	0	0	5872	0	7756	0	7681	0
0	0	0	5109	0	5609	1982	5583	0
0	2165	2651	6022	2806	6776	3362	6733	0

X281.3	X281.54	X281.69	X281.785	X281.9	X282.075	X282.4	X282.8	X283.3
3647	3071	2851	2064	3314	3162	3607	2660	3627
4791	3771	3351	2262	4292	3899	4716	3034	4779
5311	5267	5151	4760	5307	5252	5311	4992	5311
2271	2224	1941	1912	2260	2236	2271	1869	2271
2051	1878	1441	1103	2008	1917	2051	451	2051
3394	3174	2960	2351	3349	3323	3394	3119	3394
4456	4382	4313	2271	4440	4436	4460	4405	4460
5594	5364	5220	5115	5553	5555	5594	5537	5594
2588	2478	2053	1892	2564	2232	2588	2434	2588
1820	1741	1435	1285	1807	1640	1820	1496	1820
1739	1677	1411	1276	1720	1631	1739	1271	1739
3278	3236	2927	2797	3269	3189	3278	2562	3278
3246	3175	3027	2820	3245	3191	3246	2830	3246
2470	2448	2013	1938	2467	2359	2470	1164	2470
6073	5813	4808	3633	6046	5289	6073	4904	6073
2836	2729	2154	1411	2833	2587	2836	1103	2836
9646	9368	9135	5028	9513	9457	9646	9416	9646
10629	10243	9867	3925	10484	10439	10629	10191	10629
10820	10635	10293	9916	10767	10656	10820	10723	10820
5568	4228	2431	1618	5233	4263	5565	2974	5568
3424	2834	1617	1101	3210	2772	3424	1039	3424
3900	2889	2150	1031	3600	3050	3901	1458	3901
5741	4824	3480	2000	5559	5306	5741	5462	5741
3639	3337	2925	2548	3557	3444	3639	3470	3639
3991	3523	2929	2429	3866	3628	3991	3517	3991
2739	2178	1697	1188	2491	2259	2738	2131	2739
3979	3411	2067	1736	3708	3280	3977	2261	3979
7756	5432	1660	0	7023	5122	7754	2576	7756
5609	4902	4036	3361	5358	4908	5609	4065	5609
6776	5640	4006	3628	6275	5454	6776	5302	6776

X283.57	X283.86	X284.335	X284.86	X294.815	X297.96	X298.91	X300.9	X302.835
0	1974	3487	0	1080	1672	2967	3097	1496
0	1628	4526	0	982	1933	3525	3864	1901
0	1841	5307	1027	1868	3428	5273	5277	1941
0	339	2271	0	0	1630	2201	2212	0
0	0	2046	0	0	396	1415	1878	0
0	0	3388	716	0	0	3381	3372	0
828	3784	4460	3243	0	0	4459	4456	0
1208	4805	5594	4728	2478	3614	5588	5574	3015
0	0	2588	0	0	1008	2563	2539	537
0	0	1820	0	0	717	1801	1779	0
0	0	1739	0	0	461	1687	1663	0
0	0	3275	0	0	1871	3173	3172	0
0	0	3246	0	0	1638	3221	3218	0
0	0	2470	0	0	657	2014	2390	0
0	0	6067	0	1272	2866	5883	5876	2456
0	0	2836	0	0	405	1981	2379	0
5265	8504	9639	6905	2386	3044	9482	9302	2926
3961	7474	10627	5413	0	0	10351	10098	0
5750	9622	10816	7843	3748	5310	10773	10701	4748
0	0	5513	0	0	0	4606	4849	1737
0	0	3409	0	0	0	2232	2480	0
0	0	3865	0	0	0	2723	2794	0
0	0	5736	0	941	2102	5616	5548	3128
0	1085	3620	0	0	1294	3530	3478	767
0	1108	3971	0	0	1000	3788	3693	0
0	0	2718	0	0	0	2285	2143	0
0	0	3964	0	0	0	2964	2777	0
0	0	7689	0	0	0	4246	4627	0
0	0	5579	0	0	0	4907	4736	0
0	2428	6727	0	1357	3263	5689	5456	2754

X303.04	X303.275	X304.285	X305.41	X306.13	X306.33	X307.385	X308.295	X309.32
1374	3558	3263	3499	3086	2972	3472	2685	3470
1290	4516	4192	4498	3661	3443	4471	2894	4511
1009	5307	5272	5307	5087	5255	5307	5060	5310
374	2269	2242	2269	2250	2192	2268	1909	2268
0	2042	1997	2037	1987	1907	2033	1383	2030
0	3390	3369	3392	3284	3160	3388	1395	3367
0	4460	4459	4460	4303	4378	4458	3887	4448
2848	5594	5587	5594	5405	5436	5594	4908	5590
0	2588	2569	2581	2558	2262	2578	1547	2563
0	1820	1811	1816	1786	1645	1812	1137	1792
0	1739	1731	1735	1711	1619	1721	1009	1719
0	3276	3253	3278	3269	3234	3274	2415	3276
504	3246	3226	3246	3238	3195	3246	2641	3246
0	2470	2389	2470	2410	2431	2470	2279	2470
0	6069	5807	6071	4967	5841	6069	5305	6067
0	2836	2688	2836	2793	2714	2836	2316	2836
2486	9646	9598	9640	5420	9331	9625	8824	9592
0	10629	10530	10622	8817	9877	10620	8738	10595
5443	10819	10798	10814	7957	10444	10814	9730	10792
0	5510	5066	5489	2342	3546	5487	1496	5447
0	3404	3204	3401	2845	2315	3398	965	3373
0	3852	3459	3832	3202	2220	3824	739	3816
2116	5738	5613	5736	3762	4804	5732	3559	5691
940	3632	3562	3621	3151	3276	3619	2728	3611
745	3984	3886	3980	3315	3434	3959	2555	3959
0	2722	2537	2709	1420	2024	2708	1658	2676
0	3964	3683	3933	3017	3052	3914	2026	3900
0	7695	6479	7635	5160	4009	7588	1125	7557
0	5591	5203	5560	4711	4604	5545	3778	5545
2133	6746	5802	6702	4752	4965	6665	4226	6678

X310.495	X314.815	X316.915	X318.91	X321.035	X327.395	X328.105	X329.3	X331.315
1960	3014	1663	3124	0	2357	1579	1067	3298
2150	3609	1854	4100	0	2647	541	906	4230
5094	5292	4126	5299	2303	2169	0	0	5287
1744	2233	1217	2228	652	994	0	0	2253
1212	1881	0	1985	0	468	0	0	2011
1284	3375	1678	3357	0	1778	2025	1295	3379
3206	4457	4142	4443	0	2297	2278	2507	4460
4316	5564	5349	5573	2600	3141	2825	3032	5594
1439	2569	1396	2556	0	1005	0	1101	2576
1104	1798	377	1798	0	1107	0	845	1816
1007	1719	241	1697	0	927	0	718	1736
2512	3223	1185	3250	719	1713	0	1214	3269
2550	3229	1938	3231	926	1536	0	1470	3242
2278	2371	539	2451	473	352	0	0	2457
5325	5917	3428	5961	1292	0	0	0	5938
2373	2501	0	2603	0	0	0	0	2813
6059	9322	7942	9079	0	8677	7715	5311	9596
5442	9983	5799	9595	0	7676	9453	3162	10528
7359	10569	9060	10716	0	9601	9134	4137	10801
0	3685	1315	5077	0	1258	0	0	5147
0	1909	0	2848	0	1232	0	0	3258
0	2263	0	3121	0	1002	0	0	3551
0	4900	3734	5586	0	3169	1151	1490	5696
1319	3132	2100	3480	0	2561	1203	1210	3597
1419	3065	1392	3765	0	2488	0	1041	3919
913	1079	0	2291	0	1425	0	0	2523
1062	1672	0	3203	0	1881	0	0	3768
0	1945	0	6072	0	0	0	0	6785
2745	4036	0	4982	0	1835	0	0	5153
3497	5070	3592	5707	0	3079	1141	0	5811

X332.35	X333.105	X333.405	X335.29	X335.41	X337.475	X344.125	X346.16	X356.86
1126	2145	1180	0	2013	0	3210	2214	2961
1092	2499	1305	0	2434	0	3040	1884	3778
0	3357	0	0	4111	1133	4554	3874	5290
0	1833	803	0	1631	0	2186	1955	2206
0	934	0	0	878	0	1787	1339	1920
1299	0	1596	0	1932	0	3286	2700	3326
2629	2009	3131	0	3383	0	4432	4065	4444
2622	4818	4145	0	4458	2425	5420	3654	5564
0	971	0	853	1345	0	2308	1937	2560
0	992	0	909	956	0	1729	1443	1787
0	986	0	488	904	0	1657	1424	1702
0	2549	2144	512	2631	0	3256	2840	3229
0	2690	2148	0	2695	700	3220	2879	3223
0	966	0	0	1404	0	2065	1501	2421
0	0	0	0	3156	0	3319	1493	5845
0	498	0	0	1150	0	2141	1961	2344
5749	3204	6109	0	6986	0	8116	4147	8917
3740	0	3902	0	6733	0	9225	6741	9146
4891	7406	6420	0	8229	2870	9608	2752	10608
0	0	0	0	2326	0	0	0	4634
0	1094	0	0	1446	0	1604	1792	2342
0	631	0	0	1374	0	1649	1812	2494
1269	939	1252	0	2362	0	2813	0	5366
1004	2084	1225	0	1998	0	2433	1824	3306
0	1835	986	0	2228	0	2357	1831	3347
0	938	0	0	1450	0	0	0	1768
0	1307	0	0	1935	0	1234	1239	2215
0	0	0	0	2231	0	0	0	3787
0	1580	0	0	3059	0	3453	1799	4335
0	2571	0	0	3757	0	4402	0	5397

X359.025	X361.35	X375.325	X378.88	X379.29	X391.29	X394.825	X403.355	X405.31
0	756	0	0	0	2255	0	1121	154
0	1317	0	0	0	3170	0	1022	234
0	1463	3163	0	1960	4437	3514	4902	1972
0	1137	816	0	0	2044	798	1353	0
0	0	0	0	0	1092	0	443	0
0	0	0	0	0	0	1594	0	0
2910	0	1413	2962	0	0	3999	2470	0
3589	0	3661	4540	0	1669	5191	4132	0
0	1470	0	0	0	1571	770	1054	0
0	1655	432	0	702	1727	0	1113	644
0	854	437	0	651	1487	0	1080	622
0	2686	966	0	0	2943	666	1550	0
0	0	1464	0	0	2211	1486	1756	0
0	396	354	0	0	1561	0	1404	0
0	4142	0	0	1564	3382	1801	2643	0
0	1002	446	0	1156	1996	0	1609	1128
6118	0	2456	7863	0	0	7075	5721	0
3494	0	0	6403	0	0	4526	4147	0
5507	0	3404	9131	0	2955	7407	6925	0
0	0	0	0	0	2007	0	0	0
0	0	0	0	0	2243	0	0	0
0	0	0	0	0	1841	0	0	0
0	2429	0	1942	0	1375	0	0	0
0	913	0	1064	0	1926	0	0	0
0	0	0	0	0	1631	0	0	0
0	0	0	0	0	1680	0	465	0
0	0	0	0	0	2407	0	0	0
0	0	0	0	0	1579	0	0	0
0	0	0	0	0	1278	0	0	0
0	2963	0	2023	0	3296	2061	0	0

X415.35	X416.76	X417.34	X418.395	X419.39	X436.335	X437.33	X439.36	X441.345
0	0	2682	1979	3017	3016	0	0	2163
0	0	3660	1372	4059	3516	1062	0	2896
1813	0	5227	3900	5283	5125	1286	1815	5228
0	0	2198	1004	2241	2200	0	0	2133
0	0	1764	0	1996	1807	0	0	1446
0	0	1480	1295	2934	3244	0	0	743
0	772	3244	3955	4209	4415	0	0	1079
0	0	5178	5217	5533	5566	0	0	5112
813	0	2265	1619	2530	2360	640	726	2386
716	0	1790	1439	1820	1704	703	659	1734
577	0	1702	1239	1733	1676	586	576	1540
0	0	3240	2604	3264	3231	599	0	3202
0	0	3118	2611	3236	3197	0	0	3084
0	0	2346	659	2465	2207	0	0	2311
0	0	5426	1971	5921	4244	0	0	5438
0	0	2635	886	2802	2174	0	0	2271
0	6619	7083	8426	9019	9209	0	0	3287
0	4357	5946	6884	8595	9320	0	0	0
0	4971	8838	9190	10222	10423	0	0	7418
0	0	3261	0	4375	2931	0	0	3052
0	0	2653	0	3076	2492	0	0	1518
0	0	2517	0	3298	2218	0	0	965
0	0	3289	0	4768	4738	0	0	4546
0	0	3081	1454	3459	3378	0	0	3068
0	0	2758	1326	3530	3353	0	0	2311
0	0	2118	0	2441	1806	0	0	1482
0	0	3108	0	3663	2845	0	0	1580
0	0	3463	0	6069	1980	0	0	1592
0	0	2892	0	4414	4468	0	0	2707
0	0	4304	2365	5055	4936	0	0	4601

X446.375	X462.345	X464.37	X480.345
0	1922	2597	1196
0	1935	2417	2177
2048	2725	5159	909
0	1520	2124	642
0	443	1663	0
0	0	2366	0
1279	2834	3948	0
3031	4815	5433	0
0	1372	2016	0
0	1139	1512	0
0	1001	1463	0
0	2650	3136	0
0	2548	3123	0
0	501	2344	0
0	0	4811	0
0	0	2317	0
3546	4979	8051	0
0	2615	6285	0
4180	7371	9583	0
0	0	0	0
0	0	630	693
0	0	0	0
0	0	1881	0
0	1405	2354	0
0	1457	2021	0
0	0	1005	0
0	0	1262	499
0	0	0	0
0	0	3449	0
0	2400	4262	0

	X199.935	X200.155	X200.975	X201.92	X203	X203.775	X204.055	X204.795
Ctrl	3196	0	3206	3207	3203	747	2719	2572
./AVA 587	2853	613	2865	2869	2867	696	2503	2416
./AVA 587	2525	560	2579	2579	2575	0	1606	1709
./AVA 587	2204	1069	2231	2233	2229	653	1902	1671
./AVA 593	3846	723	3924	3925	3912	0	1957	2102
./AVA 593	4561	0	4619	4625	4613	0	2404	2659
./AVA 594	3716	1264	3732	3734	3734	0	2623	2154
./AVA 594	3439	1297	3469	3470	3466	0	2348	2100
./AVA 594	3488	895	3531	3536	3533	0	1446	1656
./PTX 576 1	4174	1406	4429	4453	4407	2488	2840	2785
./PTX 576 2	2242	271	2334	2337	2328	504	1469	955
./PTX 576 3	3002	943	3105	3105	3094	1610	2099	2029
./PTX 577 1	4033	1049	4068	4075	4065	1237	3069	3258
./PTX 577 2	4491	0	4578	4584	4580	766	2124	2361
./PTX 577 3	4681	0	4744	4747	4741	1725	2464	2829
./PTX 586 1	2872	612	3019	3023	3008	1553	2341	2466
./PTX 586 2	3036	609	3093	3096	3091	1709	2273	2530
./PTX 586 3	3095	906	3193	3196	3189	2022	2458	2577

X205.145	X205.81	X205.99	X206.17	X206.955	X207.12	X207.835	X208.155	X208.955
0	1717	0	1153	824	2608	1811	3184	1628
497	1618	474	1395	2808	2697	1763	2851	1536
0	616	0	1000	2236	2236	695	2563	728
627	853	0	1559	2077	2092	898	2220	693
0	1293	0	1043	2181	3042	0	3909	1102
0	2288	0	1249	3812	3798	0	4617	1657
1125	2130	0	1717	3637	3420	1293	3728	1811
1084	1698	0	1776	3183	3169	1118	3457	1632
0	1510	0	1176	3115	2577	805	3536	973
0	2616	0	2191	3505	3255	1674	4331	1314
371	525	0	461	1477	1547	474	2315	472
572	2070	0	1647	2322	2673	771	3063	654
0	1880	0	1759	3565	3533	1132	4064	1434
0	1856	0	1079	3911	3162	0	4572	734
0	2890	0	1028	4282	3090	0	4711	903
0	1782	521	1202	2695	2477	1376	2963	1654
0	1795	386	1243	2280	2669	1158	3074	1217
0	2268	342	1674	2703	2845	1409	3179	1136

X209.15	X209.98	X210.15	X211.185	X212.13	X212.69	X212.95	X214.125	X214.725
2937	768	2905	0	2570	2037	1128	3199	3193
2759	1496	2815	740	2713	1205	1850	2850	2861
2361	794	2442	666	2133	707	965	2553	2575
2177	809	2208	1122	2181	944	711	2226	2077
3735	923	3769	908	3262	1199	1305	3886	3906
4389	1726	4371	1058	3732	2021	2068	4611	4579
3575	2197	3566	1303	3241	2615	2802	3725	3722
3320	1876	3376	1379	3160	1484	2240	3454	3456
3148	1570	3211	989	2765	2642	1629	3518	3475
4209	1674	4256	1463	4107	2963	905	4144	4363
2049	730	2112	451	1670	1472	483	2256	2314
3001	995	2967	1243	2849	1452	665	2969	2527
3938	2027	3974	1132	3632	2433	1946	4017	4051
4031	1412	4097	0	3229	1700	1545	4508	4545
4400	1576	4401	0	3729	2280	1148	4607	4679
2866	2115	2865	741	2507	2306	1655	2900	3006
2966	2137	2994	663	2722	2304	1457	3001	3058
3101	2240	3127	1090	2976	2279	1446	3067	3079

X215.125	X216.045	X216.955	X219.835	X220.965	X221.9	X222.165	X222.745	X223.1
0	1310	3158	1699	2935	3169	0	3201	2692
0	1685	2837	2060	2721	2824	445	2863	2668
0	630	2554	1222	2172	2541	586	2579	1673
1594	535	2212	1057	1992	2209	814	2228	2052
0	852	3885	902	3453	3906	757	3923	2821
953	1554	4601	1669	4379	4615	1101	4627	4115
856	1899	3712	2366	3488	3712	1278	3728	3470
846	1682	3447	2118	3297	3452	1332	3460	3203
1365	1295	3515	1351	3161	3530	932	3536	3019
822	1584	4254	1916	4107	4040	1281	4440	3753
273	479	2280	881	1883	2238	0	2336	1588
2232	941	3057	1334	2930	3046	899	3104	2476
650	1384	4052	2043	3715	4032	816	4074	3306
849	1140	4552	1843	4010	4555	0	4582	3387
688	1371	4712	1899	4509	4673	0	4749	3689
0	1730	2948	2010	2932	2399	631	3020	2652
364	1448	3062	1759	2976	3011	581	3095	2620
1007	1731	3151	2034	3129	3092	982	3197	2850

X223.855	X224.135	X224.865	X225.095	X225.98	X226.16	X227.1	X227.3	X231.105
2720	3207	2187	2694	3198	2302	1883	2945	1807
2461	2860	2323	2757	2864	2456	2025	2822	2796
1715	2578	1438	2443	2548	1936	1612	2561	2157
1566	2232	1459	2171	2226	2104	505	2226	2029
2955	3917	1962	3201	3920	2900	2661	3913	3323
4083	4625	2887	3941	4626	3546	2632	4618	4167
2906	3733	3131	3462	3733	3224	2955	3719	3653
2795	3467	2886	3238	3469	3106	2800	3447	3355
2773	3534	2184	2803	3528	2636	1648	3531	3267
3824	4413	2786	3758	4357	3720	2791	4351	2986
1625	2333	1030	1564	2308	1389	705	2296	1639
2820	3092	1947	2504	3020	2544	603	3057	2624
3360	4074	2920	3658	4074	3325	2762	4056	2245
3800	4580	2552	3829	4585	2914	1743	4558	3935
4359	4739	3005	3736	4738	2967	1205	4697	4133
2703	3023	2390	2533	2988	2143	1688	2970	2355
2780	3087	2387	2573	3087	2332	1648	3058	2226
2946	3189	2631	2751	3189	2708	1735	3156	2602

X232.975	X235.97	X236.905	X237.74	X238.095	X238.815	X239.135	X239.73	X240.135
3190	0	3184	3191	1249	3207	2887	3192	0
2856	768	2854	2848	1716	2869	2635	2849	844
2569	575	2572	2571	1438	2581	2406	2577	657
2223	524	2224	2226	1674	2233	2080	2226	1025
3903	0	3910	3921	2048	3930	3754	3920	1142
4607	1368	4623	4621	2959	4627	3873	4623	1842
3723	1963	3729	3725	2743	3732	3652	3723	2091
3456	1757	3459	3462	2686	3475	3353	3464	1872
3532	1581	3533	3536	2333	3536	3417	3534	1544
4357	1778	4344	4393	3145	4478	3530	4423	1619
2317	0	2317	2320	646	2341	1678	2327	0
3086	1162	3095	3101	2071	3106	971	3102	1150
4064	1181	4065	4074	2384	4075	3479	4072	1047
4570	854	4573	4575	2230	4587	4161	4581	1240
4741	937	4725	4733	2112	4750	4060	4746	769
2996	1525	2987	3007	1729	3030	2672	3012	666
3082	1319	3084	3093	1731	3097	2588	3089	607
3175	1648	3177	3180	2300	3197	2798	3185	1163

X240.735	X241.11	X241.97	X242.155	X243.12	X243.86	X248.1	X252.055	X253.315
3189	3208	3208	3108	1186	1820	800	1710	3205
2868	2869	2862	2757	1103	2062	901	2100	2869
2578	2581	2559	2149	589	1133	0	1571	2581
2233	2231	2219	2194	961	941	0	1583	2230
3921	3929	3921	3619	917	1487	0	1392	3927
4621	4627	4627	4551	1878	2489	894	2496	4627
3734	3734	3717	3605	2274	2399	1366	2904	3733
3465	3475	3440	3302	2052	1942	1024	2633	3470
3534	3536	3531	3448	1111	1175	0	2064	3536
4441	4478	4209	3739	716	0	0	2235	4480
2325	2341	2266	2171	0	227	0	0	2341
3102	3104	2951	2692	572	0	0	1655	3106
4068	4075	4054	3866	1062	1928	0	1861	4074
4580	4587	4559	4378	784	2060	0	1976	4587
4746	4752	4680	4471	402	1188	0	1528	4750
3018	3030	2871	2631	635	956	513	1250	3028
3091	3096	3026	2732	419	820	0	1088	3094
3190	3197	3026	2731	839	983	0	1775	3196

X254.02	X254.375	X254.78	X255.09	X255.38	X255.78	X256.385	X256.785	X257.15
0	2765	3191	0	3208	3199	3201	3202	656
0	2739	2868	1409	2863	2868	2869	2869	0
0	2514	2576	1190	2578	2578	2581	2581	0
0	2157	2231	1314	2232	2233	2232	2233	0
0	3892	3920	1854	3930	3926	3929	3930	0
1075	4607	4620	0	4627	4627	4627	4627	1013
1395	3661	3733	2890	3729	3734	3731	3734	1234
873	3405	3466	2728	3475	3469	3473	3472	1009
1000	3503	3531	0	3535	3536	3536	3536	644
0	4179	4435	825	4479	4467	4476	4474	0
0	2212	2333	856	2341	2337	2340	2341	0
317	3013	3104	501	3106	3106	3106	3106	625
0	4013	4069	1297	4069	4073	4075	4075	725
0	4532	4577	0	4586	4584	4587	4586	0
0	4617	4745	0	4751	4744	4752	4752	0
454	2933	3019	1430	3030	3020	3030	3030	0
311	2988	3095	412	3095	3097	3097	3097	0
527	3112	3191	440	3196	3197	3197	3194	0

X257.875	X258.72	X259.125	X259.87	X260.245	X260.92	X262.025	X264.925	X266.28
3188	3137	3198	3024	1069	2655	2377	0	0
2852	2800	2840	1216	1697	2668	1406	0	0
2560	2177	2577	2270	371	2170	902	0	0
2223	2116	2219	1181	1052	1629	765	0	0
3895	3711	3917	3650	0	3567	2064	0	0
4601	4511	4623	4430	0	4481	3574	0	1038
3724	3610	3717	1051	2473	3695	2112	859	926
3446	3283	3451	2939	761	3390	1808	544	820
3530	3453	3535	2976	0	3461	1971	585	728
4393	4286	4293	3485	0	3912	2152	0	0
2306	2245	2309	0	1352	1774	304	0	0
3097	3069	3005	1290	1156	2810	975	0	0
4061	3964	4062	3539	0	3863	1371	0	0
4558	4453	4567	4142	0	4437	2149	0	0
4724	4690	4711	4050	0	4491	2261	0	0
2995	2976	2976	2311	0	2886	1296	373	0
3090	3060	3048	2423	0	2959	1204	0	0
3186	3168	3126	2419	0	3038	1280	0	0

X267.155	X270.995	X276.915	X278.71	X279.34	X279.58	X279.78	X280.04	X280.345
0	922	2996	1234	3207	0	1901	1467	3201
0	515	2779	1321	2869	0	1526	1189	2855
0	355	2451	558	2579	0	2394	652	2581
0	261	2117	0	2233	0	0	1978	2229
0	580	3823	1070	3928	777	3708	678	3928
990	580	4561	3138	4627	1785	0	4438	4627
656	1301	3653	2524	3732	1208	2031	2684	3722
784	1060	3313	2208	3474	948	1931	2226	3465
728	694	3441	2303	3535	911	0	3400	3536
0	0	3623	1908	4479	0	950	2801	4461
0	0	1948	0	2341	0	299	1064	2333
0	0	2802	1128	3105	631	0	2780	3101
0	690	3706	1252	4074	0	2390	1543	4075
0	0	4403	1986	4585	0	0	4135	4581
0	0	4436	2130	4749	0	0	3902	4746
0	617	2455	2108	3026	0	2558	372	3027
0	589	2579	1536	3097	0	0	1740	3097
0	528	2658	2105	3195	0	0	2283	3195

X280.59	X281.05	X281.315	X281.575	X281.865	X282.1	X282.4	X282.8	X283.105
0	2123	3208	2642	3104	2914	3207	2855	0
0	2187	2865	2496	2802	2713	2869	2821	0
0	2262	2570	2314	2570	2522	2581	2556	1367
0	1879	2228	2025	2198	2128	2233	2167	277
0	3464	3926	3830	3906	3854	3930	3890	1451
1121	4332	4622	4618	4619	4601	4625	4616	1913
1129	3509	3728	3614	3710	3703	3734	3713	1541
819	3154	3470	3314	3439	3422	3473	3441	2190
878	3369	3526	3468	3523	3481	3536	3531	1450
0	3547	4470	3207	4236	3742	4478	4278	498
0	716	2337	1378	2275	1501	2341	1976	0
0	1983	3106	2426	3029	2487	3104	3051	0
0	2783	4065	3616	4050	3854	4075	3982	538
0	4107	4579	4360	4559	4411	4587	4532	0
0	3936	4743	4141	4679	4436	4752	4692	0
0	2258	3010	2119	2956	2715	3029	2977	334
0	1857	3093	2276	3033	2641	3097	3056	0
0	2237	3195	2410	3127	2710	3196	3151	0

X283.365	X283.62	X283.97	X284.37	X284.82	X285.125	X288.13	X289.145	X290.16
3208	0	3154	3200	0	0	1795	3208	3201
2868	0	2686	2862	578	1186	1339	2869	2843
2578	526	2551	2581	329	1375	1374	2581	2559
2233	292	2149	2230	0	1020	1300	2233	2224
3929	1223	3889	3927	495	2521	1759	3926	3913
4626	2792	4625	4627	1984	3149	2547	4627	4621
3733	1937	3656	3724	2244	1802	2375	3734	3728
3475	1381	3396	3466	1769	1815	2400	3475	3460
3536	1859	3524	3536	1631	1603	1641	3536	3507
4480	0	4124	4461	0	2606	1429	4474	4189
2341	0	2224	2336	0	653	0	2340	2264
3106	0	2987	3095	512	1937	0	3103	2773
4075	0	3943	4074	0	2060	1026	4075	4051
4587	0	4536	4585	1155	2800	1638	4587	4536
4752	0	4603	4752	757	2581	1163	4751	4627
3030	0	2761	3030	392	1220	0	3029	2913
3097	0	2808	3095	366	1299	493	3097	2984
3197	0	2924	3195	746	1593	1096	3197	3064

X294.995	X298.06	X298.75	X299.15	X300.12	X300.83	X302.975	X303.34	X304.335
0	750	2742	3198	1719	3052	3111	3201	3188
0	1001	2785	2853	1251	2835	2715	2850	2824
0	0	2232	2579	810	2474	2228	2581	2575
0	0	1713	2228	657	2120	1844	2227	2201
0	0	3720	3918	1747	3828	3656	3928	3915
817	2889	4592	4625	3612	4586	4516	4626	4624
765	2715	3706	3720	2473	3707	3592	3722	3708
571	1875	3417	3457	2003	3413	3276	3468	3453
483	1409	3521	3533	2039	3510	3414	3535	3535
0	0	3880	4368	1275	3884	3872	4455	4197
0	0	1929	2313	0	2044	2020	2337	2261
0	0	2891	3050	1178	2887	2933	3099	3021
0	0	3914	4072	869	3968	3818	4075	4045
0	949	4499	4580	1675	4508	4317	4585	4567
0	588	4569	4736	893	4562	4403	4746	4679
0	591	2858	3003	587	2791	2773	3028	2942
0	0	2908	3079	486	2892	2881	3094	3027
0	0	2970	3167	657	2939	2963	3195	3095

X305.365	X305.96	X306.31	X307.365	X309.4	X310.42	X311.3	X314.76	X315.15
3184	2759	1086	3136	2942	0	0	1783	3187
2841	2199	604	2837	2836	783	427	2137	2852
2580	2082	698	2570	2569	0	456	1263	2579
2212	1823	499	2209	2218	555	491	676	2222
3924	3132	1628	3921	3907	1245	1014	2123	3921
4625	3972	3119	4623	4618	3463	2212	3957	4627
3715	2884	2199	3713	3715	2477	2227	2944	3719
3448	2633	1759	3447	3444	1686	1847	2448	3457
3535	2304	1813	3535	3525	2191	1954	2237	3536
4330	2368	0	4338	4369	0	0	0	4381
2301	588	0	2309	2305	0	0	0	2320
3048	1577	653	3027	3034	0	0	1002	3048
4063	2902	923	4050	4031	0	0	1664	4068
4572	3550	1497	4566	4570	1486	0	2985	4572
4705	3077	475	4716	4713	442	0	2043	4730
2991	1369	0	2983	2992	0	0	819	3011
3066	1497	0	3052	3064	0	0	711	3076
3154	1791	0	3150	3166	0	0	1027	3179

X316.115	X316.82	X318.765	X320.025	X327.1	X328.19	X329.15	X331.38	X333.13
0	1098	3150	1249	3163	2545	2554	3166	2442
0	1297	2838	683	2750	1814	1576	2773	2206
0	368	2562	0	2514	1668	1628	2554	2018
0	0	2198	0	2121	1247	1014	2120	1574
0	870	3895	499	3839	2784	2620	3813	3448
1225	3292	4614	1983	4589	4094	3766	4576	4486
845	2587	3713	1052	3687	3117	2549	3694	3457
803	2040	3453	902	3380	2668	1920	3424	2930
732	1987	3524	1002	3497	2729	2082	3507	3240
0	1183	4198	1518	3709	1834	2071	3773	2103
0	0	2273	356	1770	740	569	1990	322
0	914	3037	1301	2795	1697	1888	2869	1830
0	755	4042	678	3878	2485	1714	3775	2440
0	1726	4549	930	4429	3080	2237	4308	3187
0	1778	4697	1782	4480	2099	1425	4144	2282
0	1303	2946	1460	2628	1529	1132	2573	1423
0	888	3017	1006	2622	1540	1237	2651	1320
0	1513	3132	1688	2741	1590	1360	2715	1626

X335.295	X336.675	X337.31	X340.76	X343.095	X344.145	X346.055	X355.13	X356.93
0	2498	0	0	0	2655	1542	0	2845
1788	2464	0	0	0	1830	1462	0	2716
679	1255	0	0	0	1687	1321	0	2225
0	962	0	0	0	1428	1340	0	1735
0	2506	666	0	0	2381	2321	0	3611
2899	4248	2212	1030	1850	3409	2853	1399	4537
2593	3137	2179	1552	2211	2402	2342	1522	3655
1415	2892	1533	1233	1568	2134	2206	1071	3275
1426	2984	1808	1420	1835	1892	1737	1499	3425
0	3646	0	0	0	1289	0	0	3420
0	1536	0	0	0	0	0	0	1418
1276	2746	0	0	0	984	899	0	2648
1921	3183	0	0	0	1981	1506	0	3491
1822	3514	0	0	0	2606	1852	0	4256
701	4070	0	0	0	2005	616	0	4202
1089	2787	0	1124	0	768	0	0	2461
755	2755	0	759	0	703	0	0	2499
1025	2957	0	1070	0	911	0	0	2623

X357.23	X359.285	X361.33	X375.245	X377.4	X378.8	X379.185	X391.28	X393.28
0	0	712	2426	0	0	0	2694	0
0	545	2256	2562	0	0	0	2612	632
0	0	1721	2491	0	0	0	2455	0
0	0	529	2102	491	0	0	2030	0
0	0	853	3714	0	0	0	3778	0
1259	1232	3487	4545	2035	1464	1697	4554	1826
1475	978	3070	3682	1648	1735	1672	3679	1756
964	0	2213	3400	902	1041	1685	3380	1645
1252	812	1969	3453	1531	1288	1893	3497	1826
0	0	681	3202	0	0	1083	3464	870
0	0	358	757	0	0	0	1200	0
0	783	1773	2411	0	0	1147	2680	1087
0	0	2646	3193	0	0	0	3670	0
0	0	2854	4013	0	0	1518	4344	1393
0	0	1801	3548	0	0	1021	4116	871
0	0	1936	2096	0	0	962	2429	733
0	441	1522	2127	0	0	1014	2380	837
0	698	2019	2450	0	0	1398	2657	1154

X399.11	X405.21	X415.2	X417.195	X418.39	X419.295	X420.33	X435.33	X436.295
0	0	0	2387	2455	3078	0	0	3059
0	0	1004	2649	2501	2832	433	0	2801
0	0	598	2489	2264	2573	0	0	2557
0	0	0	1963	1915	2194	0	0	2191
0	998	853	3552	3630	3900	749	0	3906
1143	1716	2083	4506	4586	4620	2324	1301	4621
1167	1469	2454	3698	3657	3716	2014	1207	3708
765	1581	2214	3402	3200	3445	1413	972	3438
991	1849	1907	3500	3454	3534	1816	1109	3529
0	0	815	3699	2501	4184	0	0	3829
0	0	0	1034	596	2189	0	0	2072
0	0	945	2913	2414	3025	0	0	2727
0	0	874	3575	2924	4024	0	0	3932
0	0	1477	4320	3990	4548	0	0	4521
0	0	919	4017	3381	4646	0	0	4572
0	0	1047	2704	1812	2938	0	0	2683
0	612	994	2558	1791	2994	0	0	2779
0	870	1285	2915	2035	3124	0	0	2795

X437.205	X439.125	X441.215	X462.31	X464.31	X478.34	X480.34	X481.34	X483.34
2349	0	1529	2544	1416	0	2032	0	0
2294	655	2523	2030	1945	0	1419	0	599
2233	0	1683	1933	1991	0	1317	0	0
1635	0	1048	1259	595	0	791	0	0
3514	0	2350	3390	2841	0	1931	0	0
4504	1762	4233	4493	4297	1771	3633	1259	1898
3647	1917	3632	3344	2905	1528	2784	1307	1402
3236	1549	3297	2848	2765	1227	2323	908	788
3369	1656	3227	3179	3007	980	2229	1290	1182
1971	0	2516	2144	0	0	1165	0	0
0	0	444	0	0	0	0	0	0
1756	890	2061	1136	0	0	885	0	472
2394	0	2562	2724	0	0	1257	0	0
3529	1191	3600	3738	1661	0	2099	0	0
2668	737	3373	2970	609	0	1262	0	0
1301	777	2431	1377	0	0	0	0	650
1165	691	2112	1282	0	0	0	0	525
1604	1163	2536	1629	0	0	0	0	703
