# Supplementary material for: A methodological approach to correlate tumor heterogeneity with drug distribution profile in mass spectrometry imaging data
Source: Gigascience. 2020 Nov 25;9(11):giaa131. doi: 10.1093/gigascience/giaa131 (PMC7688471; doi:10.1093/gigascience/giaa131)
Supplement: giaa131_Supplemental_Files [file giaa131_supplemental_files.zip › AdditionalFile1.docx]

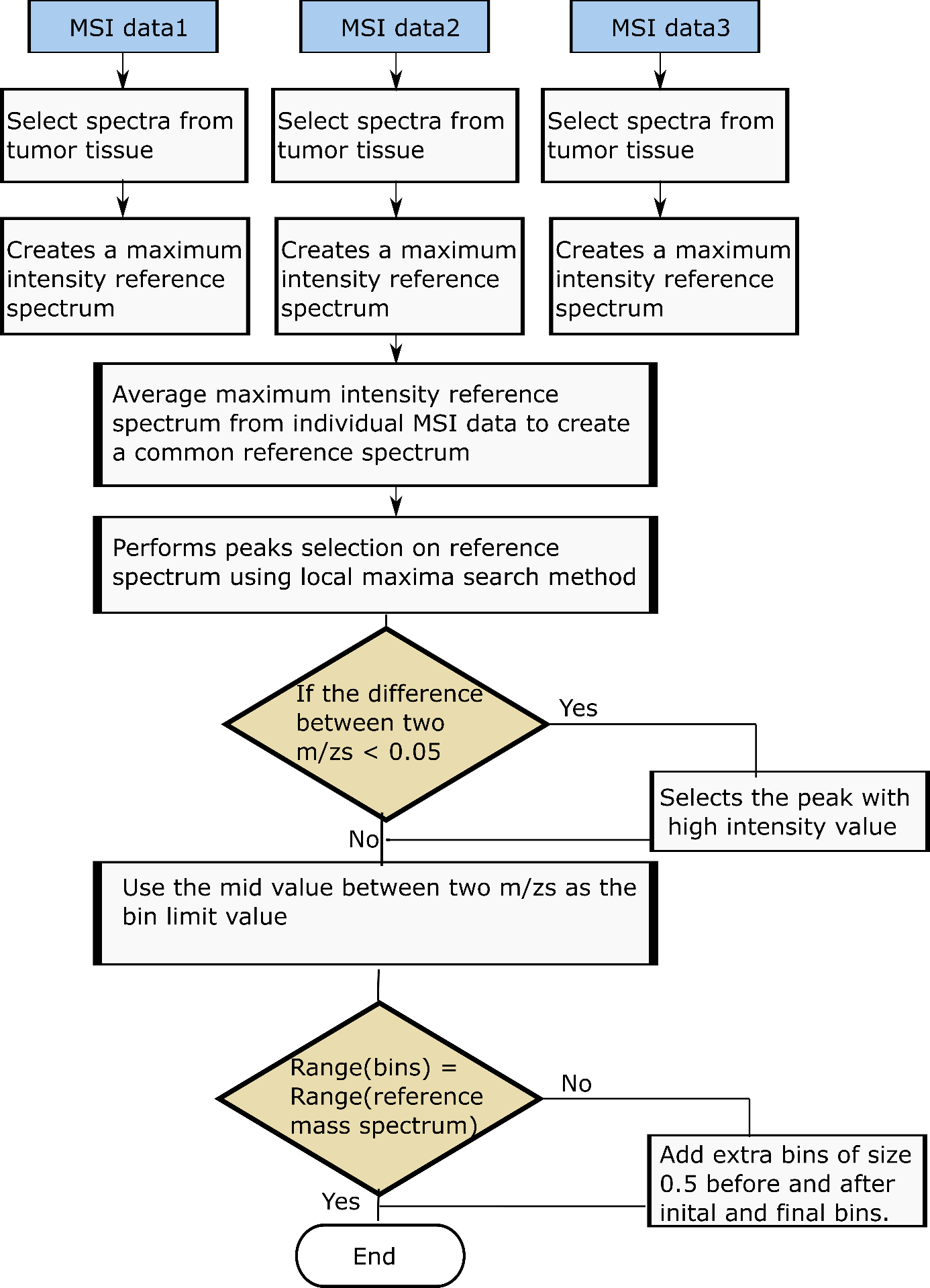


Figure S-1. A schematic workflow of adaptive bins generation for our single tumor MSI data.


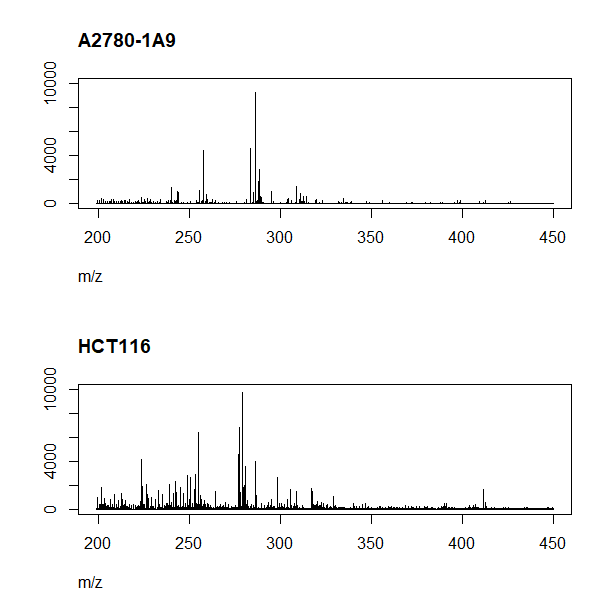


Figure S-2: Reference maximum intensity average spectrum from two tumor (A2780-1A9, HCT116) MSI data.
