# Supplementary material for: A methodological approach to correlate tumor heterogeneity with drug distribution profile in mass spectrometry imaging data
Source: Gigascience. 2020 Nov 25;9(11):giaa131. doi: 10.1093/gigascience/giaa131 (PMC7688471; doi:10.1093/gigascience/giaa131)
Supplement: giaa131_Supplemental_Files [file giaa131_supplemental_files.zip › AdditionalFile10.docx]

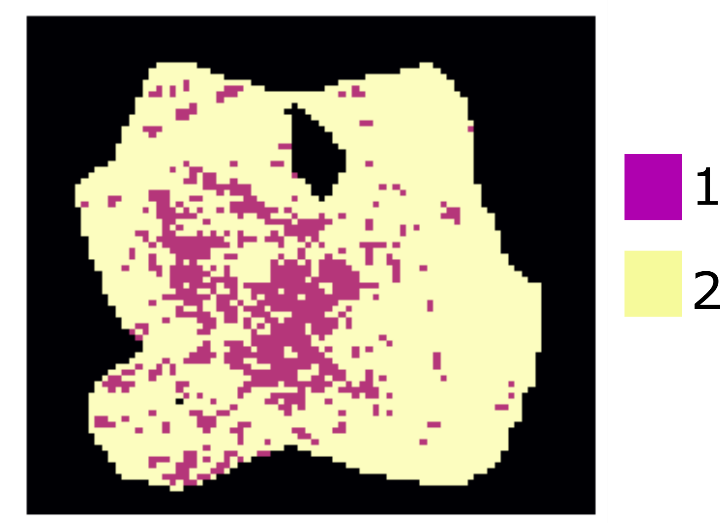


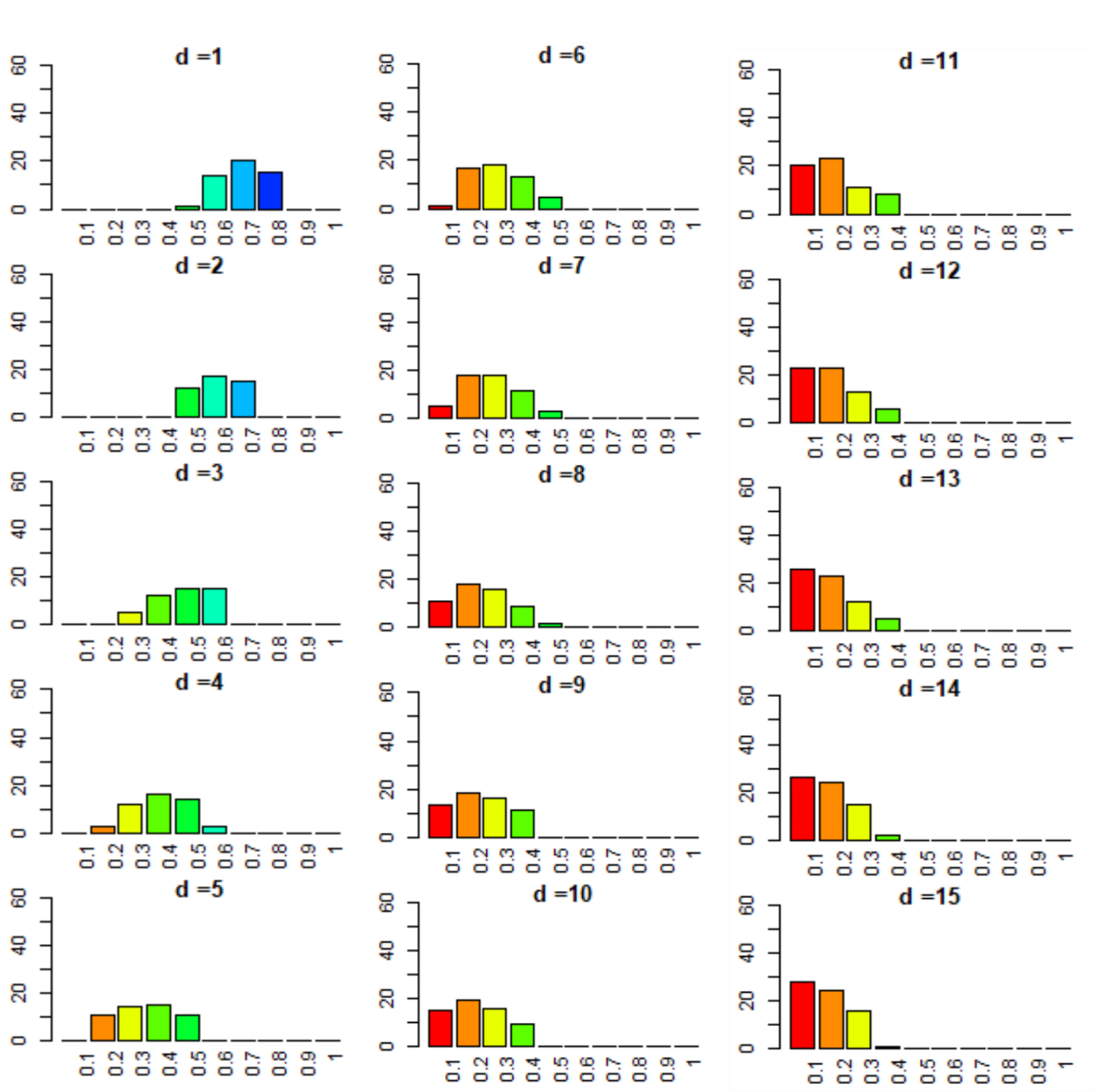


Figure S-1: Top: The image of tumor MSI data used to select variables using the SL method at different lag distances (1-15). The variables selection was performed for cluster 1 in the image. Bottom: The number of variables selected at a different lag distance and with their Moran’s I value (horizontal axis in the individual plots).


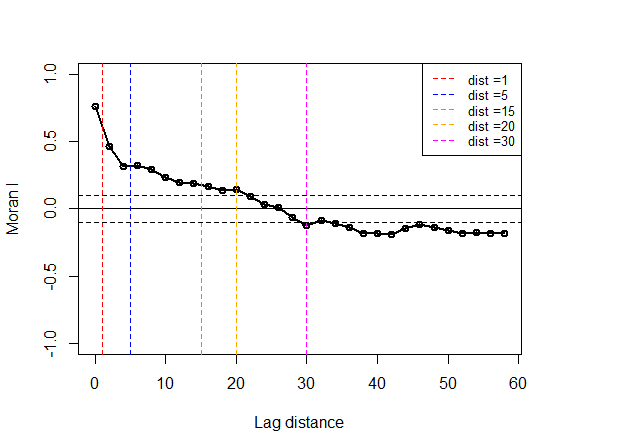

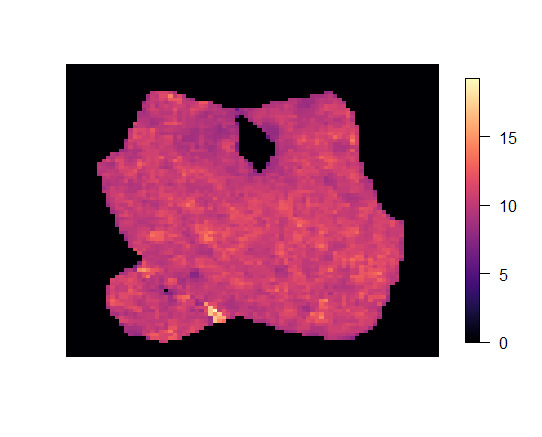


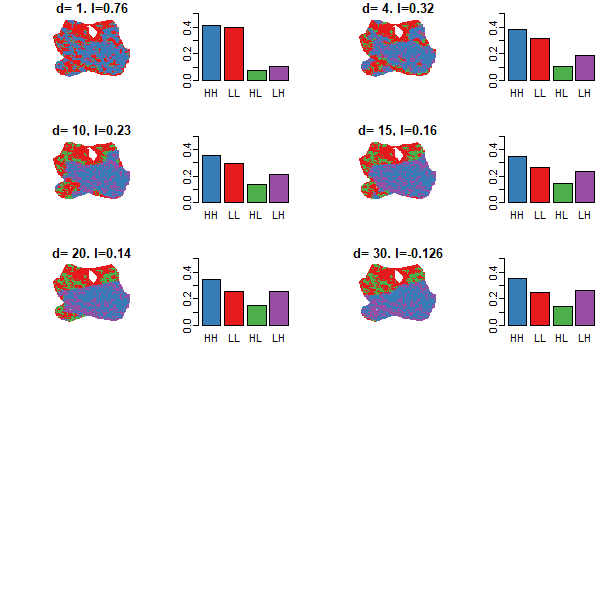


Figure S-2: The drug ion image (Top-left) with its spatial correlogram (Top-right). At the bottom, the LISA maps of a particular drug ion at different lag distances and the corresponding fractions of pixels falling into different zones of each LISA map is shown. The lag distance used to create LISA map and Moran’s I value for the image is shown in the titles.
