# Supplementary material for: A methodological approach to correlate tumor heterogeneity with drug distribution profile in mass spectrometry imaging data
Source: Gigascience. 2020 Nov 25;9(11):giaa131. doi: 10.1093/gigascience/giaa131 (PMC7688471; doi:10.1093/gigascience/giaa131)
Supplement: giaa131_Supplemental_Files [file giaa131_supplemental_files.zip › AdditionalFile2.docx]

**Text S-1**

**Moran index**

Moran’s I provides a global measure of spatial autocorrelation which is computed using the following formula^1^:

$$I= \frac{n}{\sum_{i=1}^{n} (y_{i}-\bar{y})^{2}}\frac{\sum_{i=1}^{n} \sum_{j=1}^{n} w_{ij}(y_{i}-\bar{y})(y_{j}-\bar{y})}{\sum_{i=1}^{n} \sum_{j=1}^{n} w_{ij}}$$

where $n$ equals the total number of observations in the study area, $y_{i}$ is the value of the variable at site $i$, $\underline{y}$ is the mean value of the variable, $w_{ij}$ represents the spatial weights. Moran’s I value is highly dependent upon spatial weight matrix $w_{ij}$ description. One possibility of defining the neighbors ($w_{ij}$=1) is to use a distance threshold, for example, two sites are considered as neighbors if the distance between them is smaller than or equal to some threshold. If most similar observation lies next to each other then Moran’s I returns high spatial autocorrelation value which is close to 1 and vice-versa.

**Moran scatter plot**

Moran scatter plot provides a visual representation of spatial associations in the neighborhood around each observation[1]. It plots the original value of the observations on the horizontal axis and it’s spatially lagged or weighted version on the y-axis. Moran scatters plot contains four spatial regions or quadrants defined by the horizontal line y=0 and the vertical line x=0. Points in the upper right (or high-high) and lower left (or low-low) quadrants indicate positive spatial association that are the higher and lower than the sample mean, respectively. The lower right (or high-low) and upper left (or low-high) quadrants include observations that exhibit negative spatial association, i.e. little similarity to their neighboring ones. Identify map regions based on Moran scatter plot collectively called as local indicators of spatial association (LISA)[2].

**Homogeneity index (HI)**

Homogeneity index (HI) formula, used to measure the homogeneous distribution of different tissue types in our clustered image, is based on our drug homogeneity index (DHI)[3] formula. Instead of summing the value across all grey-levels (as in original formula) we calculate the value for individual grey-level or cluster class using below formula:

$$HI= \frac{{[\sum_{j=N_{u}}^{N_{z}} jP(i,j)]}_{i=1,..,n}}{\sum_{i=1}^{n} \sum_{j=N_{u}}^{N_{z}} P(i,j)}$$

Here,$P(i,j)$ is the gray-level size-zone matrix (GLSZM) contains gray-levels or number of clusters ($n$) for this particular study as rows and the size zones ($N_{z}$) as columns. Numerator of $HI$ shows homogeneity value for individual cluster class which is further normalized based on the size of GLSZM to get the contribution of the particular cluster in a given image.

References

(1) Bivand, R. S.; Pebesma, E. J.; Gomez-Rubio, V. *Applied Spatial Data Analysis with R*; Springer New York: New York, NY, **2008**. https://doi.org/10.1007/978-0-387-78171-6.

(2) Anselin, L. Local Indicators of Spatial Association-LISA. *Geogr. Anal.* **2010**, *27* (2), 93–115. https://doi.org/10.1111/j.1538-4632.1995.tb00338.x.

(3) Prasad, M.; Postma, G.; Morosi, L.; Giordano, S.; Giavazzi, R.; D’Incalci, M.; Falcetta, F.; Davoli, E.; Jansen, J.; Franceschi, P. Drug-Homogeneity Index in Mass-Spectrometry Imaging. *Anal. Chem.* **2018**, *90* (22), 13257–13264. https://doi.org/10.1021/acs.analchem.8b01870.
