# Supplementary material for: A methodological approach to correlate tumor heterogeneity with drug distribution profile in mass spectrometry imaging data
Source: Gigascience. 2020 Nov 25;9(11):giaa131. doi: 10.1093/gigascience/giaa131 (PMC7688471; doi:10.1093/gigascience/giaa131)
Supplement: giaa131_Supplemental_Files [file giaa131_supplemental_files.zip › AdditionalFile3.docx]

**Synthetic spatially auto-correlated data generation steps**

Synthetic spatially auto-correlated data were simulated over an 80x80 grid with two spatial regions of interest of size 40x80. The number of variables included was 100, thus the overall data dimension was 80x80x100. Spatial data were extracted from a multivariate normal distribution, where instead of an ordinary covariance matrix a spatial covariance matrix was used. This spatial covariance matrix stores the association between observations/pixels from different spatial locations. In our synthetic data, we were interested in mimicking the presence of two different spatial “clusters”, for this reason, a cluster-specific spatial covariance matrix was used. All these spatial covariance matrices from the individual clusters were arranged in a single common covariance matrix which was used as an input for the spatial data generation.

To create a spatial covariance matrix for each single cluster, first spatial coordinates from the region of interest were selected. Then, a two-dimensional distance matrix was constructed by calculating the Euclidean distance between all spatial coordinates. The spatial autocorrelation among nearby observations was added using a spherical covariance function since this type of autocorrelation is commonly used in geostatistical models. A detailed description of the covariance function is provided in [1]. The procedure was repeated to create the spatial covariance matrix for another cluster. All these covariance matrices were merged into a single covariance matrix using the approach described in [2]. The spatial data simulated using this approach are commonly identified as called second-order, stationary, isotropic data.

The following spatial parameters were used in the spherical covariance function to model the two different spatial regions: range1=20, sill1=0.5, range2=10, sill2=1.5. The range controls the amount of spatial autocorrelation among nearby pixels while the sill accounts for the amount of variance. Initially, all variables were generated with a mean of zero and a standard deviation of one. Since the main assumption during variables selection is that only variables are over-represented at specific spatial locations, the statistical properties of five variables per cluster were modified by adding a random normal vector of mean one and a standard deviation of 0.1 to the original intensities. These modified variables will be considered as important variables for the clusters. Finally, to add pixels to pixels variability a random vector of mean value between 0.1-0.4 and standard deviation of 0.1 was added to the individual variables. The spatial distribution of one of the variables in the synthetic dataset and the mean spectrum from two different clusters are shown in Figure S-1a and 1c. The spatial behavior of a particular variable is shown using a spatial correlogram (Figure S-1b). The spatial correlogram is a visual tool to assess the presence of spatial autocorrelation in spatial data[3]. In this type of plot, the value of Moran’s index is plotted as a function of lag distance. In Figure S-1b, the blue line represents the correlogram from more “smooth” or high spatial autocorrelation region (range=20, sill =0.5), therefore high Moran’s I values are obtained in comparison to the low spatial autocorrelation region (range=10, sill=1.5). Noteworthy, the red and blue correlograms were calculated from spatially homogeneous (cluster 1 and 2) regions, therefore, Moran’s I value is gradually decreasing. On the contrary, since the green correlogram resulted from the combination of two homogeneous clusters, initially Moran’s I values are gradually decreasing but after a lag distance of around 40 pixels when pixels from two homogeneous regions less overlap Moran’s I value starts increasing. Finally, a multivariate representation of our synthetic data obtained by PCA is shown as a score plot in Figure S-1d.


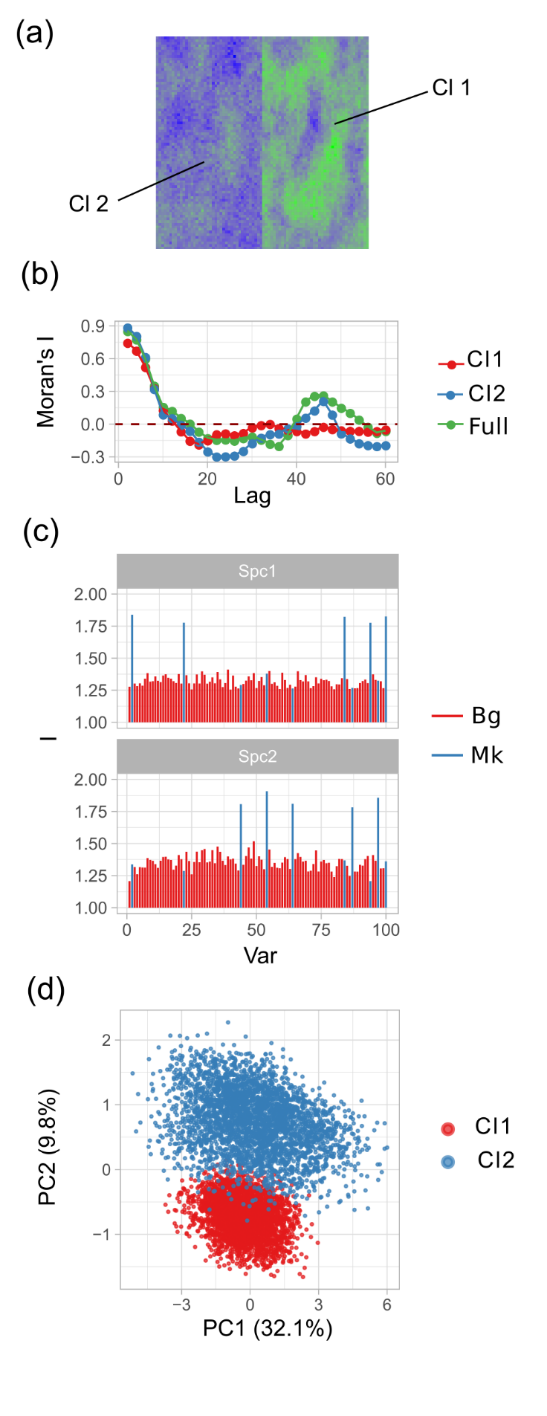


Figure S-1. Plots showing the characteristics of our spatial synthetic data. a) 2D image of a single variable from our synthetic data where Cl1 and Cl2 are two areas with different spatial properties, b) spatial correlogram which a plot of Moran’s I value at different lag distances for a single variable from two different clusters (red: cluster1, blue: cluster2) and from complete data (green). c) mean spectrum from two clusters where important (Mk) and other variables (Bg) are shown in blue and red colour, respectively. d) Principal component analysis (PCA) score plot of synthetic data.

**Compare the performance of spatial and non-spatial methods for variables selection on the synthetic data**

The performance of two spatial (Spatial lag (SL), Spatial error (SE) ) and single non-spatial (Ordinary least square (OLS)) methods was compared on synthetic data for variables selection.

A spatial lag model assumes that dependencies exist directly in the response variable, i.e. the value of the response variable at one location is affected by the value at the nearby locations. Therefore, an additional response variable which is a spatially lagged version of the original response variable is used in the regression method[3]. In contrast, SE tries to cope with spatial autocorrelation by including it in the residuals/error terms of the regression model[3,4]. In other words, SE assumes that the included response variable(s) is not enough to fully explain the spatial process, therefore model residuals are correlated. The basic formulation of OLS and spatial models are given below:

$$Ordinary least square \left( OLS \right) y=X\beta+e$$

$$Spatial lag model \left( SL \right) y= \rho Wy+X\beta+e \left| \rho\right|<1$$

$$Spatial error model \left( SE \right) y=X\beta+ e$$

$$e= \lambda We+ \varepsilon\left| \lambda\right|<1$$

In the above models:

$y$ is a response vector.

$X$ is a matrix of explanatory variables.

$e$ is a vector of error terms.

$W$ is a spatial weight matrix.

$\rho$ is an autoregressive coefficient in the spatial lag model.

$\lambda W$ is a spatially dependent error term.

$\lambda$ is an autoregressive coefficient in the spatial error model.

For variables selection, the performance of the three methods is summarized in Figure S-2. The OLS method selected 61 variables out of the initial 100 variables: 10 were corresponding to the “important” variables while the rest was falsely identified. Remarkably, already in this oversimplified scenario, OLS largely overestimated the number of variables. The situation clearly improved with spatially aware methods, which were tested for different lag distances (1-15) in terms of sensitivity and specificity (Figure S-2B). SE showed good sensitivity at a lag distance of one; however, at the same distance, it had poor accuracy, meaning that it was selecting many irreverent variables (Figure S-2B left). For lags bigger than one, the SE sensitivity drastically decreases. The SL method, instead, showed high accuracy, specificity and sensitivity values for a wide range of lags, displaying a decrease in accuracy only for large distances (high range of spatial autocorrelation) (Figure S-2B right). These results strongly suggested that SL could be the optimal choice for the analysis of real MSI datasets.


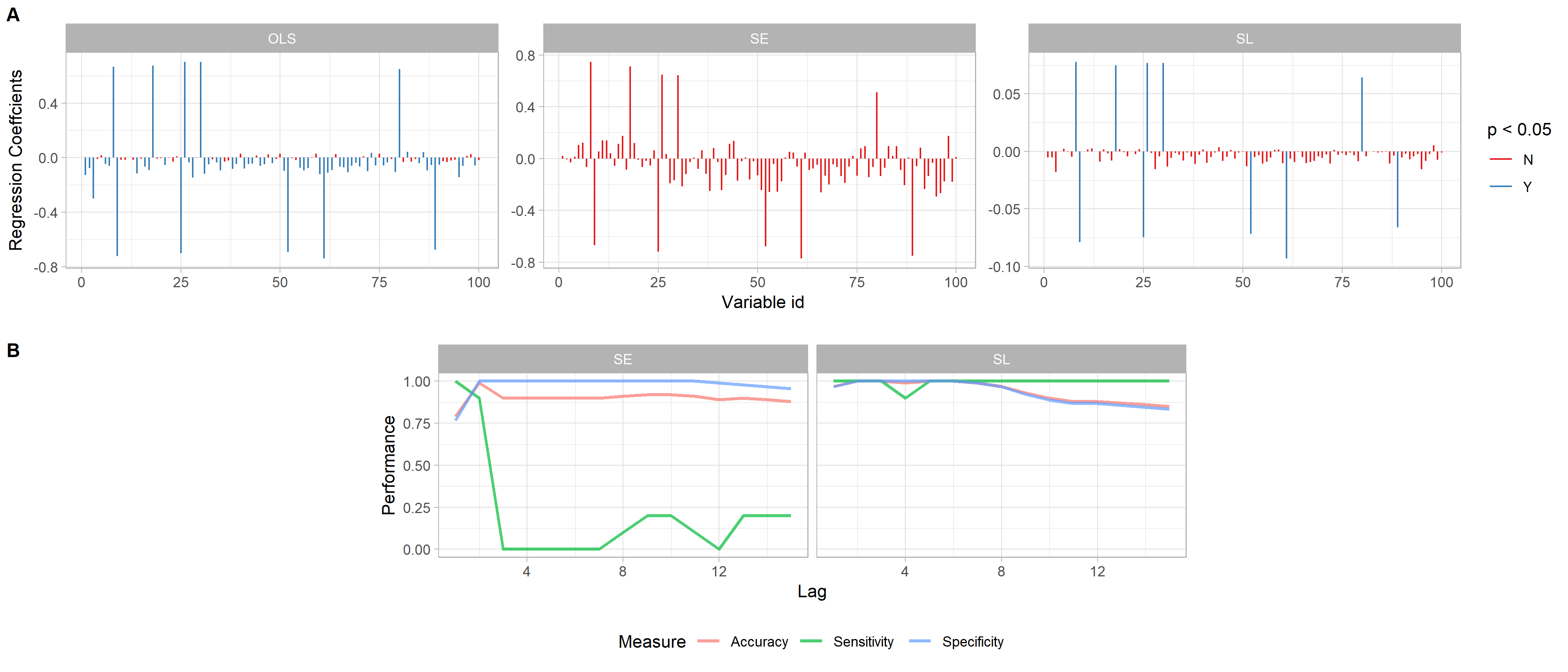


Figure S-2. Comparison of performance of spatial (SL, SE) and one non-spatial (OLS) methods for variables selection in spatial synthetic data. A) Regression coefficients from spatial (lag distance =5) and non-spatial methods where variables selected by each method are highlighted using red lines. b) Performance accuracy, specificity, and sensitivity of SL and SE methods at different lag distances.

References

1. Wackernagel H. Multivariate Geostatistics An Introduction with Applications. 2003.

2. Oliver DS. Gaussian Cosimulation: Modelling of the Cross-Covariance. Math Geol. 2003;35:681–98. <http://link.springer.com/10.1023/B:MATG.0000002984.56637.ef>

3. Bivand RS, Pebesma EJ, Gomez-Rubio V. Applied Spatial Data Analysis with R . Springer. New York, NY: Springer New York; 2008. <http://link.springer.com/10.1007/978-0-387-78171-6>

4. Kissling WD, Carl G. Spatial autocorrelation and the selection of simultaneous autoregressive models. 2008;59–71.
