# Supplementary material for: A methodological approach to correlate tumor heterogeneity with drug distribution profile in mass spectrometry imaging data
Source: Gigascience. 2020 Nov 25;9(11):giaa131. doi: 10.1093/gigascience/giaa131 (PMC7688471; doi:10.1093/gigascience/giaa131)
Supplement: giaa131_Supplemental_Files [file giaa131_supplemental_files.zip › AdditionalFile4.docx]

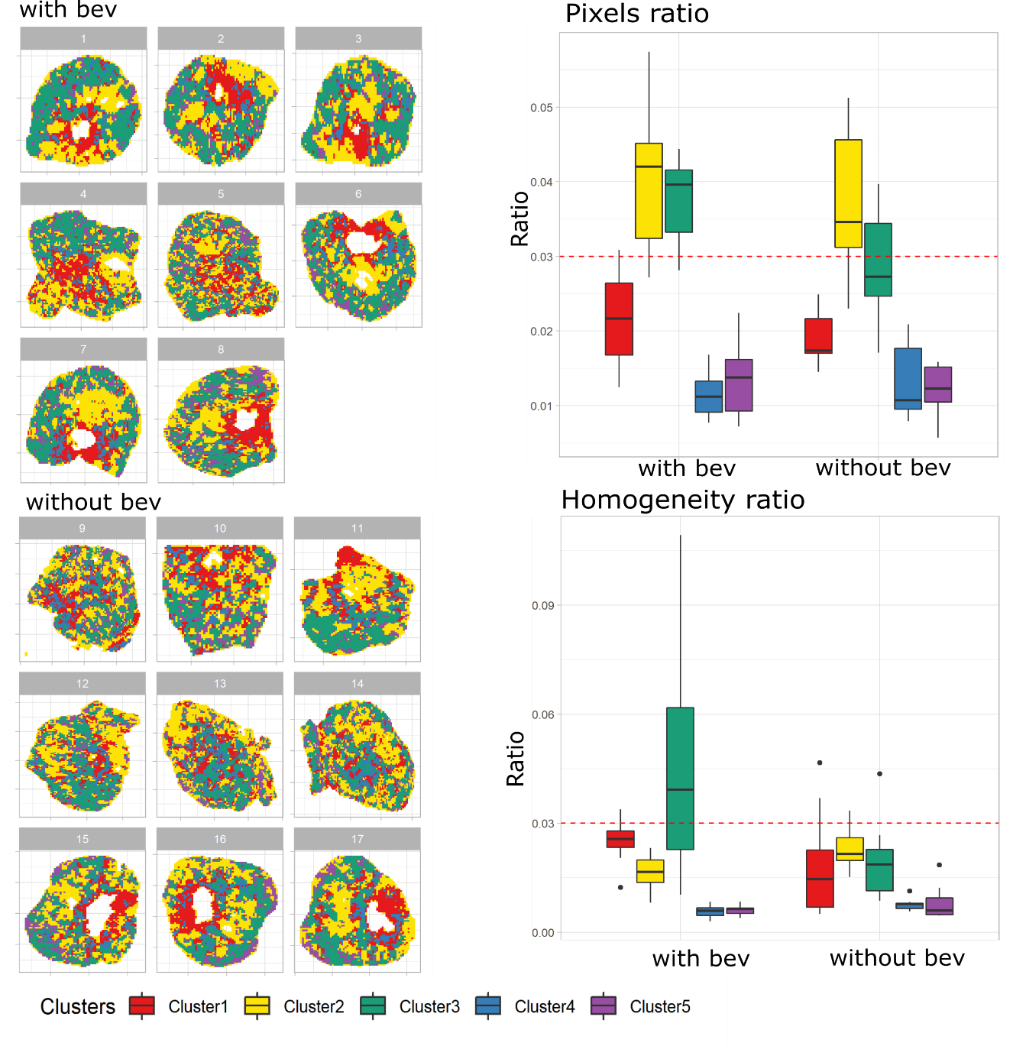


**Figure S-1:** Cluster analysis of HCT116 MSI data with and without bevacizumab treatment. Left: Representation of segmentation maps in MSI data identified by the k-means clustering method. Right: a) fraction of pixels (top) and b) homogeneity ratio (bottom) calculated from individual clusters under two treatment conditions.

**Table S-1**: Ranges and significance values for pixels and homogeneity ratios from different clusters under two treatment conditions. The p-values obtained from the linear mixed model.

| Pixel ratio | Bev+PTX  (mean±sd) | PTX  (mean±sd) | p-value |
| --- | --- | --- | --- |
| Cluster 1 | 0.0162+0.008 | 0.0201+0.012 | 0.5649 |
| Cluster 2 | 0.0245+0.0095 | 0.0217+0.0071 | 0.4391 |
| Cluster 3 | 0.00467+0.0065 | 0.0094+0.0046 | 0.0992 |
| Cluster 5 | 0.0133+0.007 | 0.0123+0.0123 | 0.8051 |

1. A2780-1A9

| Homogeneity ratio | Bev+PTX  (mean±sd) | PTX  (mean±sd) | p-value |
| --- | --- | --- | --- |
| Cluster 1 | 0.0232+0.013 | 0.0176+0.0062 | 0.1822 |
| Cluster 2 | 0.1045+0.122 | 0.0273+0.018 | 0.0651 |
| Cluster 3 | 0.0093+0.008 | 0.0141+0.0104 | 0.1822 |
| Cluster 5 | 0.0175+0.009 | 0.0079+0.0043 | 0.1822 |
|  |  |  |  |

1. HCT116

| Pixel ratio | Bev+PTX  (mean±sd) | PTX  (mean±sd) | p-value |
| --- | --- | --- | --- |
| Cluster 1 | 0.0216+0.0064 | 0.0189+0.0032 | 0.0001 |
| Cluster 2 | 0.0406+0.01 | 0.038+0.0099 | 0.893 |
| Cluster 3 | 0.037+0.006 | 0.0287+0.0069 | 0.0139 |

| Homogeneity ratio | Bev+PTX  (mean±sd) | PTX  (mean±sd) | p-value |
| --- | --- | --- | --- |
| Cluster 1 | 0.0248+0.0064 | 0.0186+0.015 | 0.1912 |
| Cluster 2 | 0.0164+0.0048 | 0.0232+0.0056 | 0.0368 |
| Cluster 3 | 0.0463+0.0332 | 0.0194+0.0109 | 0.0705 |
