# Supplementary material for: A methodological approach to correlate tumor heterogeneity with drug distribution profile in mass spectrometry imaging data
Source: Gigascience. 2020 Nov 25;9(11):giaa131. doi: 10.1093/gigascience/giaa131 (PMC7688471; doi:10.1093/gigascience/giaa131)
Supplement: giaa131_Supplemental_Files [file giaa131_supplemental_files.zip › AdditionalFile5.docx]

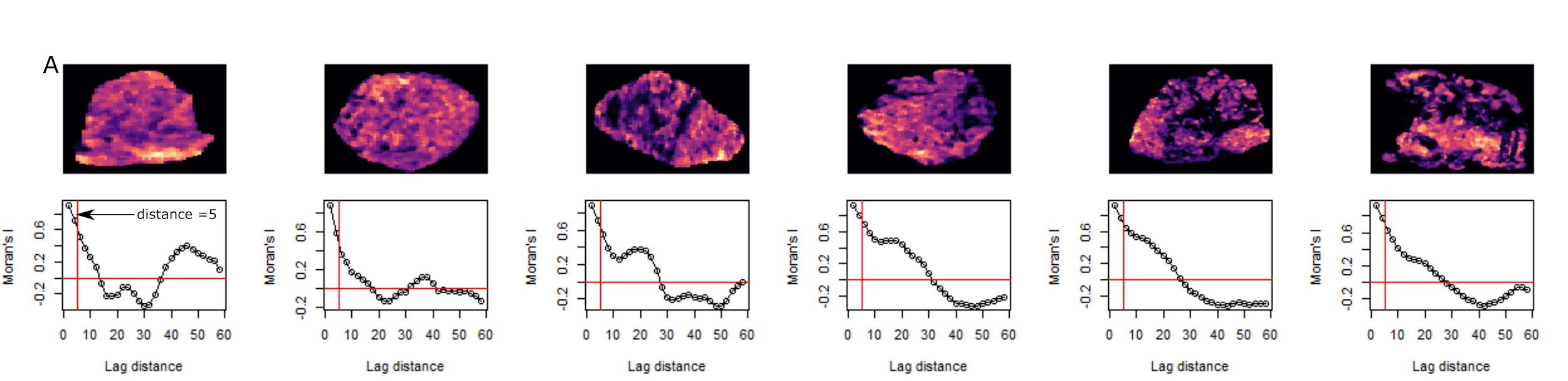


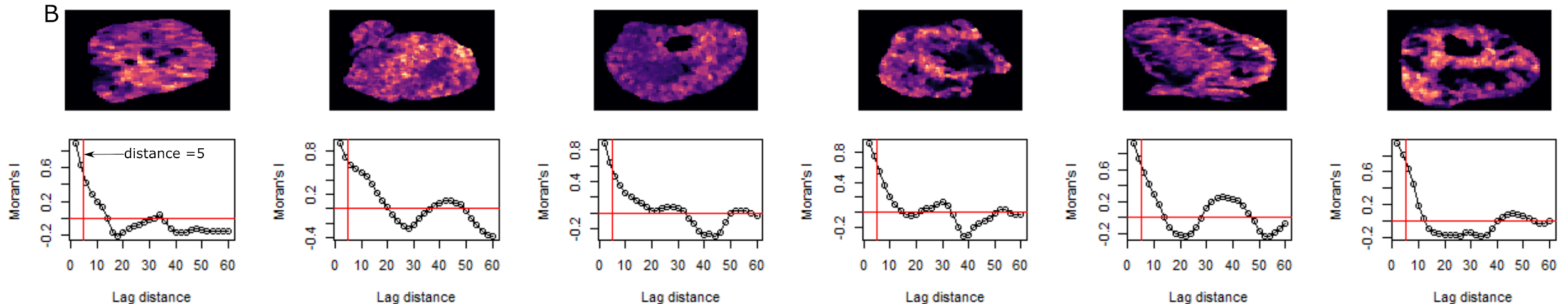


Figure S-1: Spatial correlograms of the drug-ion from A2780 (A) and HCT116 (B) tumour MSI data. A & B) The top row is the 2D intensity map and the bottom is the spatial correlogram of the corresponding drug image. The spatial correlograms are calculated for a lag distance of 1 to 60. In a majority of scenarios high and positive spatial autocorrelation is observed within a lag distance of 5.


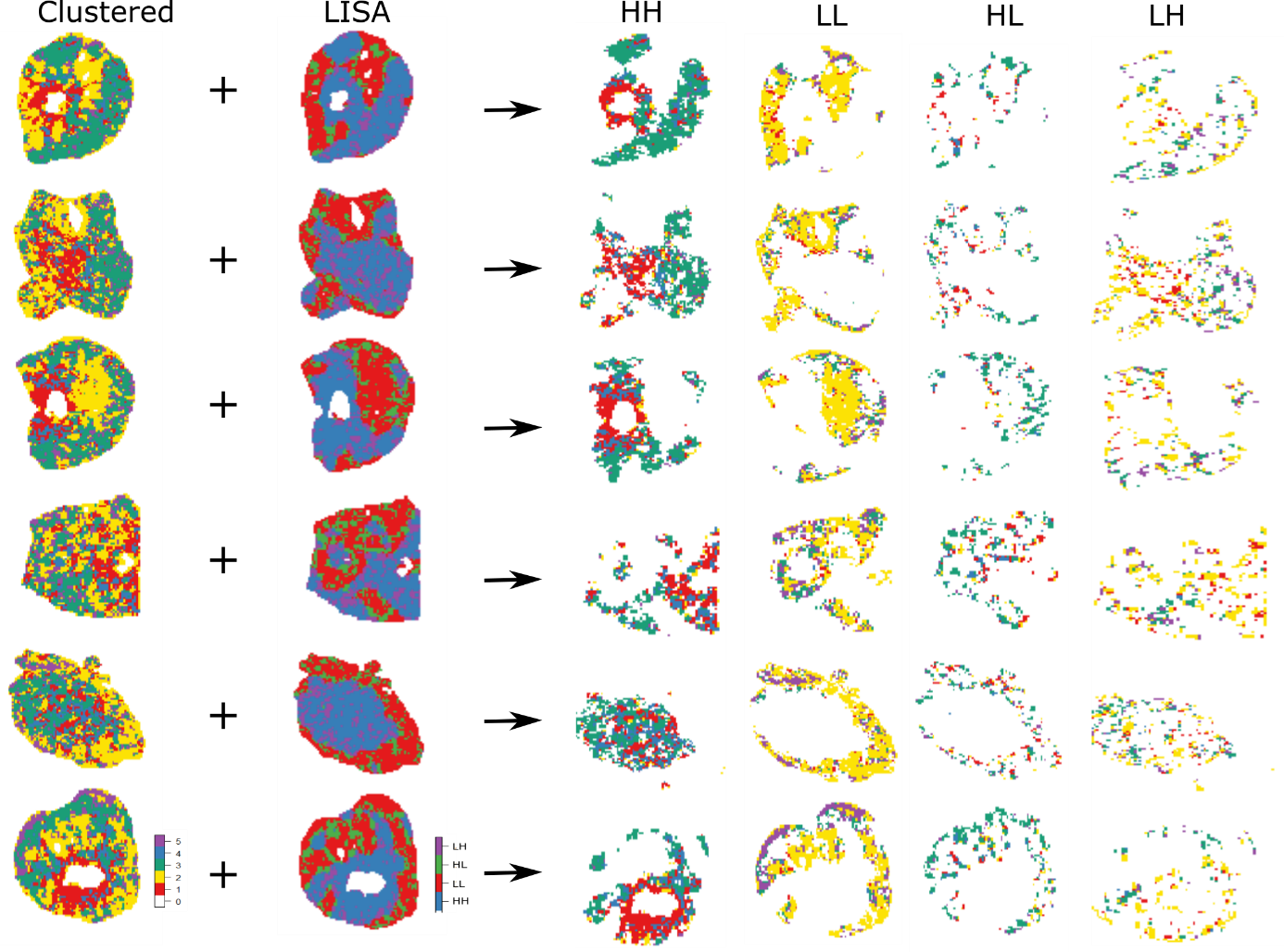


Figure S-2: Clustered image (first column), LISA map (second column) and their combination from certain tumor models are shown. The clusters found in high-high (HH), low-low (LL), high-low (HL), and low-high (LH) zones of LISA map are highlighted. (Right) In LISA map, HH, LL, HL, and LH are zones identified in Moran’s I scatter plot.

Table S-1: The percentages of pixels belonging to different cluster classes which fall into HH, LL, HL and LH zones of the LISA map for HCT116 tumor MSI data shown in Figure S-1.

|  |  | Cluster 1 | Cluster 2 | Cluster 3 | Cluster 4 | Cluster 5 | Cramer’s V |
| --- | --- | --- | --- | --- | --- | --- | --- |
| Image 1 | HH | 9.03% | 1.32% | 27.39% | 6.1% | 1.01% | 0.83 |
|  | LL | 2.4% | 23.74% | 1.88% | 0.06% | 3.76% |  |
|  | HL | 2.27% | 0.45% | 5.1% | 1.32% | 0.42% |  |
|  | LH | 0.69% | 5.72% | 3.97% | 0.45% | 2.89% |  |
| Image 2 | HH | 10.9% | 2.62% | 16.31% | 6.15% | 1.78% | 0.802 |
|  | LL | 2.03% | 20.33% | 3.15% | 0.48% | 4.07% |  |
|  | HL | 3.05% | 1.14% | 4.76% | 1.65% | 0.74% |  |
|  | LH | 2.98% | 10.23% | 3.36% | 0.46% | 3.69% |  |
| Image 3 | HH | 12.58% | 1.58% | 14.53% | 8.2% | 0.75% | 0.68 |
|  | LL | 1.15% | 24.6% | 4.43% | 0.25% | 5.43% |  |
|  | HL | 1.15% | 0.89% | 8.46% | 1.467 | 0.63% |  |
|  | LH | 1.09% | 6.7% | 2.9% | 0.20% | 2.96% |  |
| Image 4 | HH | 11.28% | 2.52% | 7.73% | 7.35% | 0.77% | 0.71 |
|  | LL | 1.41% | 17.34% | 6.02% | 0.47% | 8.67% |  |
|  | HL | 4.48% | 2.18% | 7.82% | 3.03% | 0.89% |  |
|  | LH | 3.12% | 10.08% | 2.26% | 0.21% | 2.35% |  |
| Image 5 | HH | 9.24% | 1.59% | 14.63% | 11.09% | 0.48% | 0.81 |
|  | LL | 1.07% | 25.79% | 1.83% | 0.08% | 7.1% |  |
|  | HL | 3.62% | 1.94% | 4.56% | 1.37% | 1.42% |  |
|  | LH | 1.78% | 5.95% | 3.49% | 0.81% | 2.14% |  |
| Image 6 | HH | 14.43% | 2.03% | 9.33% | 7.52% | 0.55% | 0.81 |
|  | LL | 0.81% | 22.83% | 4.26% | 0.322% | 10.13% |  |
|  | HL | 1.81% | 2.07% | 10.11% | 1.91% | 1.45% |  |
|  | LH | 1.09% | 5.97% | 2.32% | 0.29% | 0.74% |  |


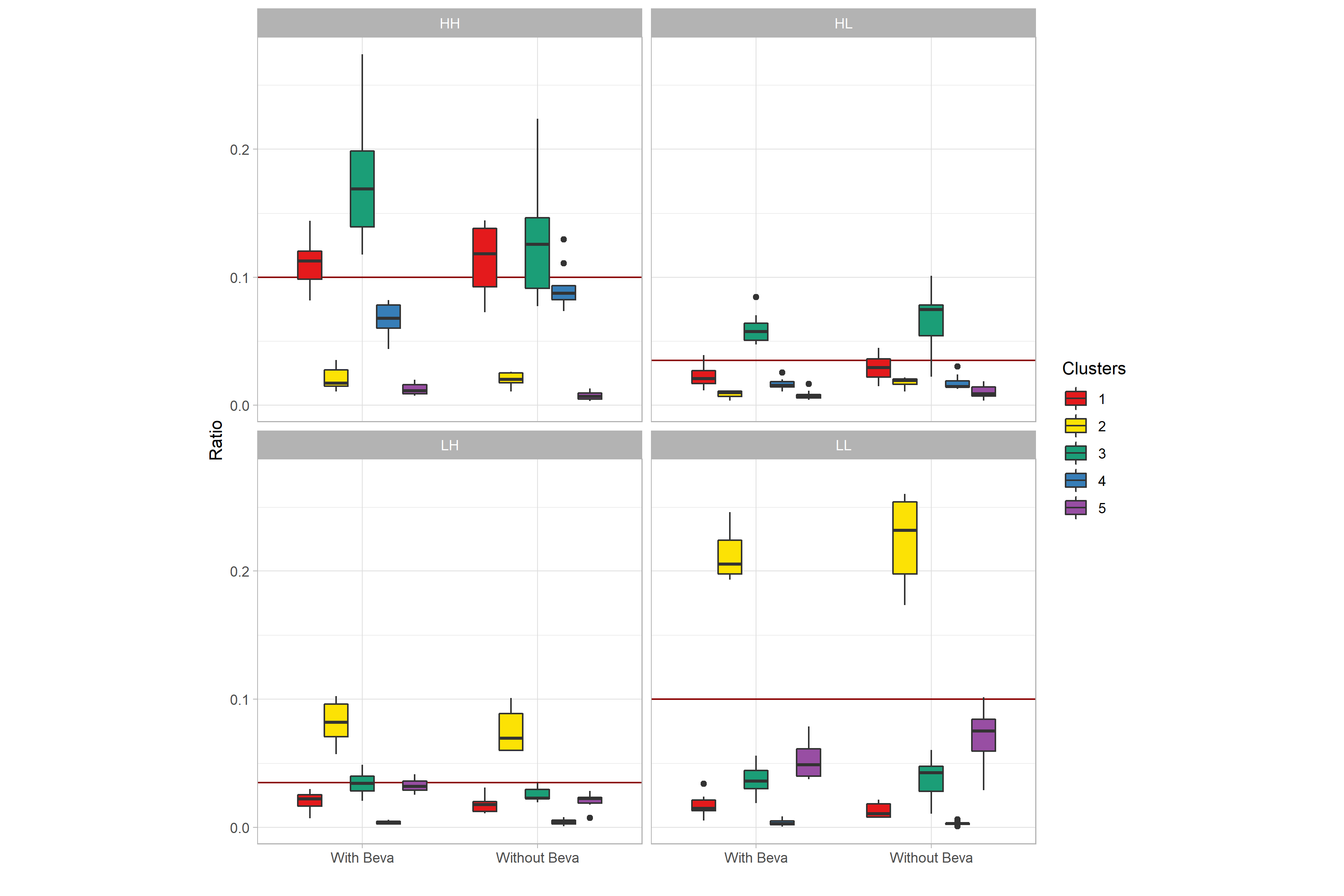


Figure S-3: The quantitative analysis to find the association between drug LISA maps and untargeted clustered images
