# Supplementary material for: A methodological approach to correlate tumor heterogeneity with drug distribution profile in mass spectrometry imaging data
Source: Gigascience. 2020 Nov 25;9(11):giaa131. doi: 10.1093/gigascience/giaa131 (PMC7688471; doi:10.1093/gigascience/giaa131)
Supplement: giaa131_Supplemental_Files [file giaa131_supplemental_files.zip › AdditionalFile6.docx]

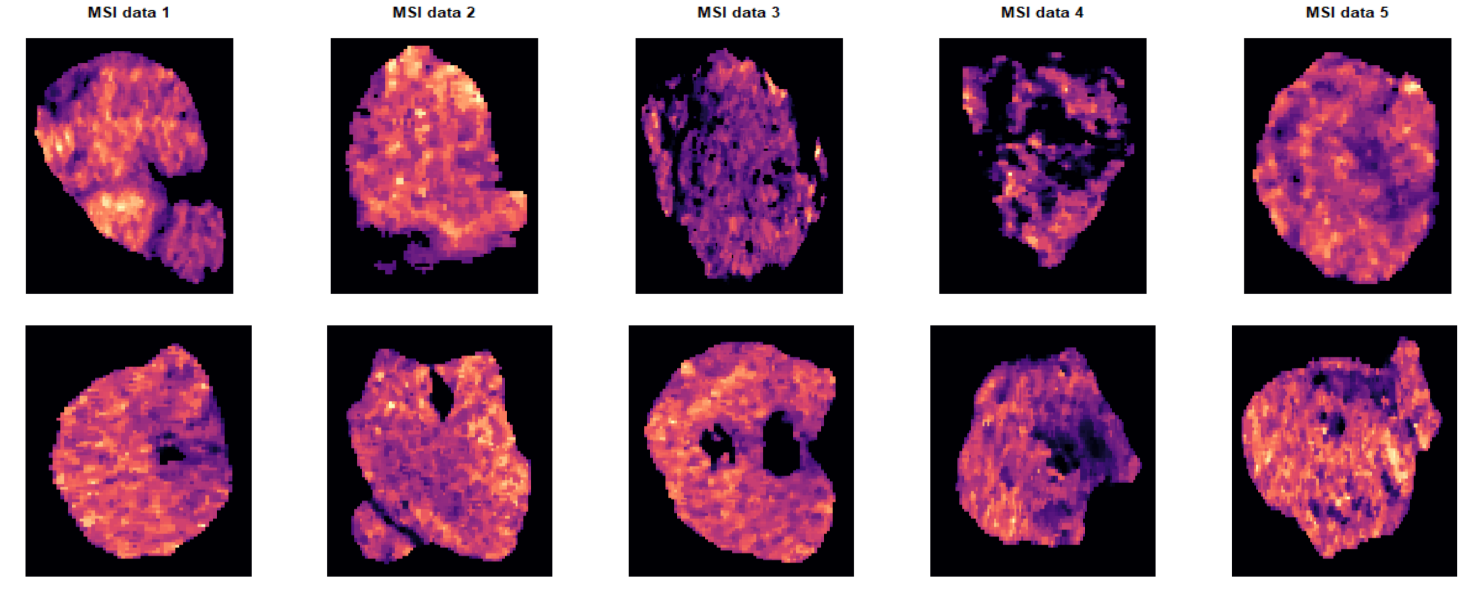


Figure S-1: MS images of selected molecular ions from different clusters from tumor MSI data. Top: 2D Images of molecular ion(m/z=335.41) in different tumor models from A2780 MSI data. Bottom: 2D Images of molecular ion (m/z=281.315) in different tumor models from HCT116 MSI data. As per the spatial lag model, the particular molecular ions (m/z =335.41, 281.315) have high intensity in clusters 2 and 3 for respective MSI tumor data.
