# Supplementary material for: A methodological approach to correlate tumor heterogeneity with drug distribution profile in mass spectrometry imaging data
Source: Gigascience. 2020 Nov 25;9(11):giaa131. doi: 10.1093/gigascience/giaa131 (PMC7688471; doi:10.1093/gigascience/giaa131)
Supplement: giaa131_Supplemental_Files [file giaa131_supplemental_files.zip › AdditionalFile7.docx]

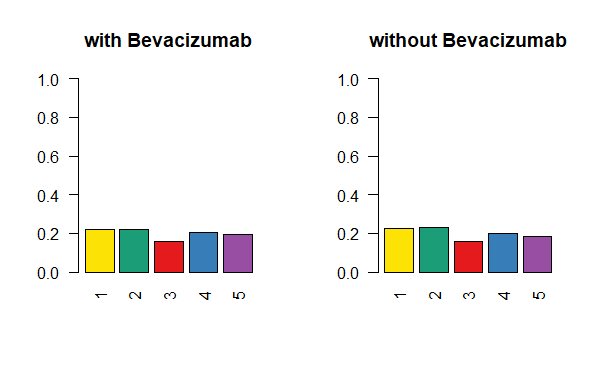


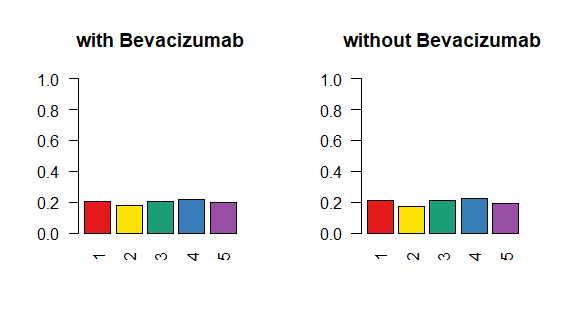


Figure-S 1 The normalized average amount of drug in each cluster-type under two treatment conditions from tumor MSI data (Top row: A2780-1A9, Bottom row : HCT116). For single treatment condition: $Normalized\_avg\_drug= \frac{\sum_{i}^{cluster} \frac{drugconc}{cluster size}}{\sum\sum_{i}^{cluster} \frac{drug conc}{cluster size}}$
