# Supplementary material for: A methodological approach to correlate tumor heterogeneity with drug distribution profile in mass spectrometry imaging data
Source: Gigascience. 2020 Nov 25;9(11):giaa131. doi: 10.1093/gigascience/giaa131 (PMC7688471; doi:10.1093/gigascience/giaa131)
Supplement: giaa131_Supplemental_Files [file giaa131_supplemental_files.zip › AdditionalFile8.docx]

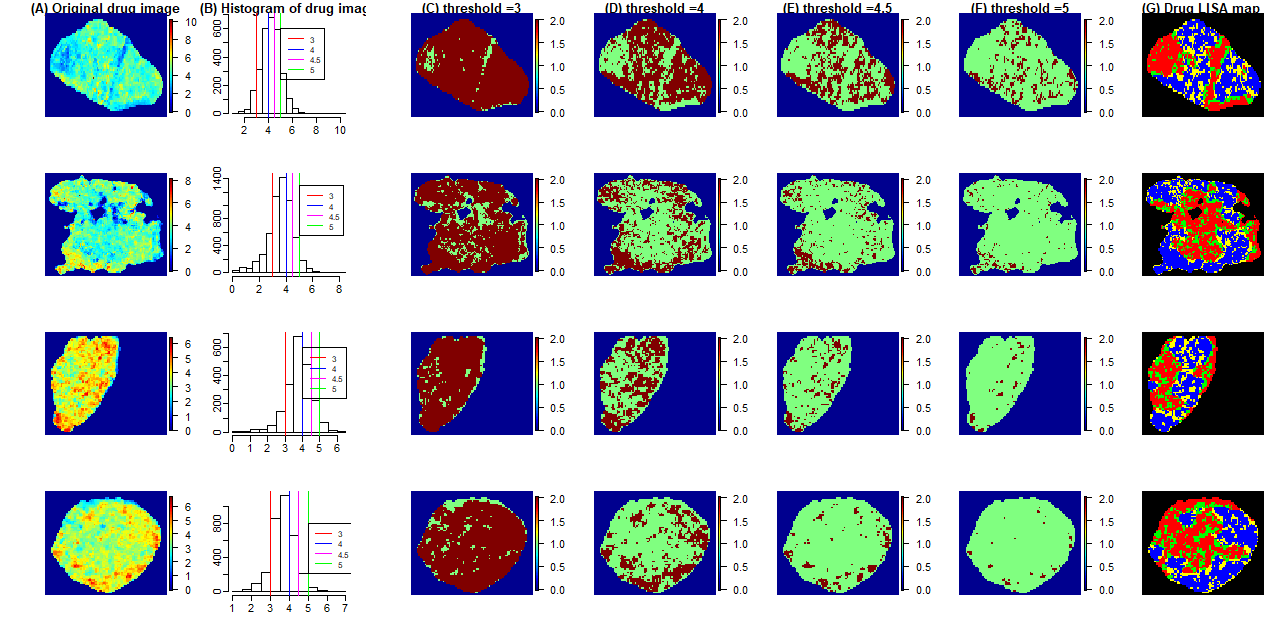


Figure-S 1: Comparison of drug binary image created by selecting manual threshold value based on histogram and LISA method. A) original drug ion image, b) histogram of drug ion, c) binary image derived from drug ion image using a threshold value of C) 3, D) 4, E) 4.5, and F) 5. G) The LISA map of the drug ion image.
