# Supplementary material for: A methodological approach to correlate tumor heterogeneity with drug distribution profile in mass spectrometry imaging data
Source: Gigascience. 2020 Nov 25;9(11):giaa131. doi: 10.1093/gigascience/giaa131 (PMC7688471; doi:10.1093/gigascience/giaa131)
Supplement: giaa131_Response_to_Reviewer_Comments_Original_Submission [file giaa131_response_to_reviewer_comments_original_submission.pdf]

Author's Response To Reviewer Comments

Close

Dear Editor.
We would like to thank the reviewers' for their constructive comments and suggestions. We are including a point-by-point discussion of their comments hereafter. We also revised our manuscript accordingly wherever it was required. The R package from our paper is online available and registered at bio.tools and Scicrunch (bio.tools: corrdrugtumormsi, RRID : SCR_018962). We believe all these changes increase the reproducibility of our paper and now is suitable to publish in the Gigascience journal.

Please consult the uploaded Personal Cover file 'EditorLetter.docx' for the response to the reviewers with figures as additional support of our response and the Peronal Cover file 'Fequency ofmolecularIonsInMSIdata.xlsx' with some data mentioned in our repose to the reviewers.

Yours sincerely
Dr. Geert Postma

Reviewer #1:
Question 1.1 The authors present a computational method for identifying spatial regions with molecularly distinct regions between control and drug therapy using previously published data. The method is well described and paper is somewhat easy to follow. The code and attached data was reviewed as well and appears clear and would be easily translatable to other projects. A more formal implementation as an R package would be desirable as the workflow is quite complex it would benefit to make a more accessible API so less experienced users wouldn't get lost.
Answer: A R package is developed that includes all main methods from our paper (bio.tools: corrdrugtumormsi, RRID : SCR_018962). Interested users can install our R-package from the Github website and refer to vignette for the usage of different functions in our package.

Question 1.2 For step 1 in the processing: How are 'drug' related peaks guaranteed to be removed from the 'microenvironment' segmentation? In processing, it is mentioned that ion peaks with correlation > 0.5 with the drug compound were removed. This seems like it would bias the segmentation if the drug had a very discrete distribution in a very particular histological region. One can imagine a scenario where a drug is distributed in area "A" exclusively along with other endogenous compounds. These endogenous compounds would be then be removed from the segmentation pipeline simply because the drug was highly partitioned into this region. Could the peaks be derived solely from undosed control tissue? Otherwise the authors statement may be misleading.
Answer: We agree with the reviewer's comment that the selection of drug-related peaks based on the correlation approach could give biased results. Unfortunately, in our study, we only had one control MSI data set per cell line, and the selection and removal of drug-related peaks based on a single animal also seem quite biased approach. Therefore, we used a heuristic approach where we removed the peaks that show more than 0.5 correlation with drug ion peak and also the peaks present in less than 20% tumor tissue area. In this way, we expected to have not too many ions with discrete distribution and ions with a high correlation with drug ion peak removed. We have validated this approach, see below.
Drug-related peaks in control animal:
The ion intensity distribution of removed peaks in a control tissue from two tumor MSI data (A2780-1A9, HCT116) is shown in Figure 1 (see Personal Cover file 'EditorLetter.docx'). Since the control animal has not been treated with drug compound we do not expect those removed peaks to be present in MSI data. But, unfortunately, that is not the case, in the figure, the drug (284.12) and its isotopic peaks (285.155) are completely absent in the control animal and other removed peaks are visible. Therefore, it seems that the threshold of 0.5 we used in our study is quite low and we need a much higher threshold to avoid removing non-drug-related peaks. On using a correlation threshold of 0.9 only the drug isotopic peak is removed from our data, therefore instead of 0.5, we will recommend the minimum correlation threshold of 0.9 in our paper.
Apart from the above analysis, we also checked whether specific peaks which could be considered as drug-related peaks were absent in the control tumor MSI data but present in the treated animal data. We did not notice such peaks. There were few empty bins in the control MSI data but those bins are also

empty in the majority of treated animals. This observation was made for both tumor MSI datasets. See added file 'FrequencyofmoelcularIonsInMSIdata.xls' where the count of the pixel with non-zero ion-intensity value across all tumor models is given.
We also looked at the impact of the removed peaks on the segmentation results. An impact of the removed peaks on the segmentation results is expected if the removed peaks are related to a unique spatial structure. The correlation between removed and other remaining peaks was calculated. All removed peaks (except for drug and its isotopic peaks) show a high correlation with many other peaks. Therefore, we don't expect our segmentation results to be biased with our approach. This statement is confirmed by the segmentation of the control tissue. The clustering of control MSI data was performed in both scenarios: with and without drug-related peaks (Figure 2, see Personal Cover file 'EditorLetter.docx') similar cluster structures were derived.

Figure 1, Figure 2, see Personal Cover file 'EditorLetter.docx'

Based on the analysis on control MSI data we agree with the reviewer that our statement regarding the removal of drug-related peaks based on correlation >0.5 is not sufficient therefore in the absence of control tissue a high correlation threshold (0.9) should be used.

Question 1.3 The authors note that mass spectral validation of model-identified differential ions is not possible and that is reasonable. In general, the spatial models presented in the findings are compelling. However, as this paper deals with spatial characterization of tissue, there appears to be no spatial validation. Indeed the obvious choice of the gold standard in pathology, H&E microscopy, is present in Figure 5 but the size of the images is so small it is negligible for spatial validation. Secondly, there are numerous published MSI examples(DOI: 10.1021/jasms.8b04879, doi:10.1074/mcp.O115.053918, https://doi.org/10.1038/srep36814) where there are clean and distinct, immediate visual association of segmented MSI images to histological regions in H&E, but here the segmentation doesn't seem to replicate much of the structure visible in Figure 5, at least AS PRESENTED. This comment isn't to push for models integrating H&E as an input but to have some qualitative result describing
the types of cells present in the tumor regions associated with the major clusters. While molecular histology is valid, it is unusual for it to not mimic classical histology.
Minor comments
All figures containing should have a scale bar indicating the physical dimension of the images.

Answer: We agree with the reviewer's comment that there needs to be more evidence regarding clusters identity and tissue types present in our dataset. However, the MSI data used in this study was initially generated to understand drug homogeneity in different types of solid tumors, and there was little histology-related work done to identify the tissue types. At this stage histological informations available, like proliferation/necrosis, vessels1 allowed us to correlate only what has been discussed in the text. Future studies will be aimed at the validation of different clustering features presented in this work with more specific, dedicated histological studies.
The reviewer's comment is correct that the optical image in Figure 5 does not show proper correlation with our clustered images, therefore we will remove all images except images 2 and 5 were necrosis region is visible both in the optical image and our clustered images.

Small errors:
Introduction
* Techniques of election <- is not proper English. Perhaps 'A valuable technique' would be less awkward.
Answer: modified accordingly.

Reviewer #2:
This is an interesting paper addressing how MSI can be effectively used to better understand the link between drug and the characteristics of malignant tissue. While it is not surprising that the physicochemical properties of both the tissue and the drug are important to passive tumor penetration and local exposure, MSI provides an important opportunity to understand the spatial and temporal dynamics of this process, and the development of effective computational workflows is vital. A few questions/suggestions for the authors follow:

Question 2.1 To what extent can the most prominent histochemical changes occurring post-bevacizumab treatment be captured by m/z range and other experimental settings studied in this untargeted MSI experiment? Since it used a limited mass range, certain important changes (e.g., in cell-surface protein expression, lipid membrane composition, etc.) may not have been measured. The authors may have addressed this question in their cited previous work, but it would be helpful to provide some additional

context.
Answer: We agree with the reviewer, our MSI data cover a limited mass range where we cannot see certain changes. However, the MSI data used in this study was initially generated to understand drug homogeneity in different types of solid tumors, and there was little histology-related work done to identify the tissue types. Our major focus, at this stage was not in a comprehensive metabolite study, but in methodology development. Future studies will be focussed, with more specific histology, on local metabolic effects of drugs and larger mass range will be used¬¬¬

Question 2.2 Since the focus of this paper is methodology, evaluation of the approach against known ground-truths is critical. In that regard, the efforts of the authors in developing a synthetic dataset and evaluating the methods on it is appreciated. There are a few ways that this assessment could be expanded to provide additional information about the robustness of the workflow. For example, Additional File 3 includes plots showing the synthetic data and some of its characteristics, but to what extent do the statistical properties of the synthetic data compare with those of real MSI datasets? The SL method was recommended, but how sensitive is it to the selection of the weight matrix? If it is sensitive, are there any recommendations for selecting the weight matrix based on data characteristics? When bridging to the experimental data, has the method been tested on MSI datasets (including synthetic ones) with available complementary ground-truth labeling to help evaluate the extent to which identified clusters map to known differences? It is mentioned that peaks that were present in less than 20% of the tissue were removed to focus on more common ions. To place this 20% cutoff in context, what was the coverage area of the clusters identified? It seems possible that this step may omit significant portions of tissue heterogeneity. For future applications of this workflow, how should this cutoff threshold be selected? Overall, how robust are the results/workflow recommendations to the choices of distance metric and clustering index?
Answer:
a) MSI data is a type of spatial data where nearby observations are highly correlated with each other. In our study, we had used the spatial autocorrelation function to generate similar spatially autocorrelated synthetic data. In additional File 3, a spatial correlogram plot from our synthetic data is presented, which is quite comparable with the spatial correlogram of drug molecular ions from different tumor MSI data shown in Additional File 5.
b) Yes, the results from spatial methods depend upon the selection of the spatial weight matrix. We performed a small simulation study with MSI data. And, we included our conclusion and recommendation in the discussion section, page 17.
c) In our study, we don’t have ground-truth labeling. Therefore it was not possible to completely validate the identify clusters. The validation of identified clusters was performed with H&E stained images shown in Additional File 5 where the necrosis tissue present in the optical image shows similarity with clusters in the A2780-1A9 and HCT116 data.
d) In our study, we discarded peaks with less than 20% coverage area, assuming that they represent the noisy peaks and could influence our clustering algorithm. The decision of discarding those peaks was based on multiple trials. For the removal of peaks, we tried a threshold of 10%, 20%, and 30%. With a threshold of 10%, we missed some noisy peaks and with 30% we excluded a few extra peaks in our data. Therefore, a threshold of 20% seems a reasonable choice. Moreover, this step of peak removal was performed on each tumor MSI data separately. Therefore, unless particular peaks were present as noise in all datasets, we do not expect them to be completely removed from our input data for the clustering.

The range of different clusters size derived across all tumor models is shown in Table 1 below. In our MSI data, we have identified the clusters of size smaller than 20% of total tumor tissue area which means after removing a fraction of peaks (which we assume noise-related) we are still able to find spatially relevant smaller clusters.

Table 1. Range of different clusters size in two tumor MSI data.

		Cluster 1	Cluster 2	Cluster 3	Cluster 4	Cluster 5
A2780	Minimum size	212	624	40	224	153
	Maximum size	3429	2959	1691	1270	3078
HCT116	Minimum size	337	734	558	208	186
	Maximum size	833	1674	1297	682	605

Question 2.3 Given the dose of the drug administered, how much exposure within the tumor is expected based on pharmacology, and how might this affect the output? It would also help to provide more explanation of Figure S1 in Additional File 7. In it, the concentration (units undefined) of the drug in

each cluster appears to be very similar, across both cell lines and treatment arms; however, the comparison between the clusters and the LISA maps appears to suggest differently. Also, interpretation of the LISA maps of drug exposure in Figure 3 and the data in Table 1 in terms of histology is briefly in the Discussion, but it would be helpful to expand on this.
Answer: For drug MSI data generation, the solid tumors were explanted from mice 6 hours after drug treatment. Therefore we do not expect dramatic changes in tissue architecture due to the drug compound. Moreover, all tumor models have received drug treatment, so if any metabolic change occurs, we expect it to be consistent across all tumor MSI data.
Additional File 7, Figure 1, shows the average amount of drug in each cluster-type under two treatment conditions. The drug average value in each cluster is further normalized with the sum of the average drug in a particular treatment condition. Note, this second step is performed simply to show the drug concentration in the range of 0-1. Apart from that the average drug concentration profile from steps 1 and 2 is similar.

References

1. Cesca, M.; Morosi, L.; Berndt, A.; Fuso Nerini, I.; Frapolli, R.; Richter, P.; Decio, A.; Dirsch, O.; Micotti, E.; Giordano, S.; et al. Bevacizumab-Induced Inhibition of Angiogenesis Promotes a More Homogeneous Intratumoral Distribution of Paclitaxel, Improving the Antitumor Response. Mol. Cancer Ther. 2016, 15 (1), 125–135. https://doi.org/10.1158/1535-7163.MCT-15-0063.

Close
