# Supplementary material for: A methodological approach to correlate tumor heterogeneity with drug distribution profile in mass spectrometry imaging data
Source: Gigascience. 2020 Nov 25;9(11):giaa131. doi: 10.1093/gigascience/giaa131 (PMC7688471; doi:10.1093/gigascience/giaa131)
Supplement: giaa131_Response_to_Reviewer_Comments_Revision_1 [file giaa131_response_to_reviewer_comments_revision_1.pdf]

Author's Response To Reviewer Comments

Close

Dear editor,
Herewith the revised manuscript. We have taken care of all comments and revisions/additions as requested in the manuscript, modified and checked ref.49 and we have registered the software, as requested, and included the identifiers in the manuscript.

Kind regards,
Geert Postma

Close
