# Supplementary material for: A methodological approach to correlate tumor heterogeneity with drug distribution profile in mass spectrometry imaging data
Source: Gigascience. 2020 Nov 25;9(11):giaa131. doi: 10.1093/gigascience/giaa131 (PMC7688471; doi:10.1093/gigascience/giaa131)
Supplement: giaa131_Reviewer_1_Report_Original_Submission — Nathan Heath Patterson -- 5/29/2020 Reviewed [file giaa131_reviewer_1_report_original_submission.pdf]

Reviewer Report

Title: A methodological approach to correlate tumor heterogeneity with drug distribution profile in mass spectrometry imaging (MSI) data

Version: Original Submission **Date:** 5/29/2020

Reviewer name: Nathan Heath Patterson

Reviewer Comments to Author:

The authors present a computational method for identifying spatial regions with molecularly distinct regions between control and drug therapy using previously published data. The method is well described and paper is somewhat easy to follow. The code and attached data was reviewed as well and appears clear and would be easily translatable to other projects. A more formal implementation as an R package would be desirable as the workflow is quite complex it would benefit to make a more accessible API so less experienced users wouldn't get lost.
For step 1 in the processing: How are 'drug' related peaks guaranteed to be removed from the 'microenvironment' segmentation? In processing, it is mentioned that ion peaks with correlation > 0.5 with the drug compound were removed. This seems like it would bias the segmentation if the drug had a very discrete distribution in a very particular histological region. One can imagine a scenario where a drug is distributed in area "A" exclusively along with other endogenous compounds. These endogenous compounds would be then be removed from the segmentation pipeline simply because the drug was highly partitioned into this region. Could the peaks be derived solely from undosed control tissue? Otherwise the authors statement may be misleading.
The authors note that mass spectral validation of model-identified differential ions is not possible and that is reasonable. In general, the spatial models presented in the findings are compelling. However, as this paper deals with spatial characterization of tissue, there appears to be no spatial validation. Indeed the obvious choice of the gold standard in pathology, H&E microscopy, is present in Figure 5 but the size of the images is so small it is negligible for spatial validation. Secondly, there are numerous published MSI examples(DOI: 10.1021/jasms.8b04879, doi:10.1074/mcp.O115.053918, https://doi.org/10.1038/srep36814) where there are clean and distinct, immediate visual association of segmented MSI images to histological regions in H&E, but here the segmentation doesn't seem to replicate much of the structure visible in Figure 5, at least AS PRESENTED. This comment isn't to push for models integrating H&E as an input but to have some qualitative result describing the types of cells present in the tumor regions associated with the major clusters. While molecular histology is valid, it is unusual for it to not mimic classical histology.
Minor comments
All figures containing should have a scale bar indicating the physical dimension of the images.
Small errors:
Introduction
* Techniques of election <- is not proper English. Perhaps 'A valuable technique' would be less

awkward.

Methods

Are the methods appropriate to the aims of the study, are they well described, and are necessary controls included? Choose an item.

Conclusions

Are the conclusions adequately supported by the data shown? Choose an item.

Reporting Standards

Does the manuscript adhere to the journal's guidelines on minimum standards of reporting? Choose an item.

Choose an item.

Statistics

Are you able to assess all statistics in the manuscript, including the appropriateness of statistical tests used? Choose an item.

Quality of Written English

Please indicate the quality of language in the manuscript: Choose an item.

Declaration of Competing Interests

Please complete a declaration of competing interests, considering the following questions:

- Have you in the past five years received reimbursements, fees, funding, or salary from an organisation that may in any way gain or lose financially from the publication of this manuscript, either now or in the future?
- Do you hold any stocks or shares in an organisation that may in any way gain or lose financially from the publication of this manuscript, either now or in the future?
- Do you hold or are you currently applying for any patents relating to the content of the manuscript?
- Have you received reimbursements, fees, funding, or salary from an organization that holds or has applied for patents relating to the content of the manuscript?
- Do you have any other financial competing interests?
- Do you have any non-financial competing interests in relation to this paper?

If you can answer no to all of the above, write 'I declare that I have no competing interests' below. If your reply is yes to any, please give details below.

I declare that I have no competing interests.

I agree to the open peer review policy of the journal. I understand that my name will be included on my report to the authors and, if the manuscript is accepted for publication, my named report including any attachments I upload will be posted on the website along with the authors' responses. I agree for my report to be made available under an Open Access Creative Commons CC-BY license (http://creativecommons.org/licenses/by/4.0/). I understand that any comments which I do not wish to be included in my named report can be included as confidential comments to the editors, which will not be published.

Choose an item.

To further support our reviewers, we have joined with Publons, where you can gain additional credit to further highlight your hard work (see: https://publons.com/journal/530/gigascience). On publication of this paper, your review will be automatically added to Publons, you can then choose whether or not to claim your Publons credit. I understand this statement.

Yes Choose an item.
