# Supplementary material for: A methodological approach to correlate tumor heterogeneity with drug distribution profile in mass spectrometry imaging data
Source: Gigascience. 2020 Nov 25;9(11):giaa131. doi: 10.1093/gigascience/giaa131 (PMC7688471; doi:10.1093/gigascience/giaa131)
Supplement: giaa131_Reviewer_2_Report_Original_Submission — Chanchala Kaddi -- 5/29/2020 Reviewed [file giaa131_reviewer_2_report_original_submission.pdf]

Reviewer Report

Title: A methodological approach to correlate tumor heterogeneity with drug distribution profile in mass spectrometry imaging (MSI) data

Version: Original Submission **Date:** 5/29/2020

Reviewer name: Chanchala Kaddi

Reviewer Comments to Author:

This is an interesting paper addressing how MSI can be effectively used to better understand the link between drug and the characteristics of malignant tissue. While it is not surprising that the physicochemical properties of both the tissue and the drug are important to passive tumor penetration and local exposure, MSI provides an important opportunity to understand the spatial and temporal dynamics of this process, and the development of effective computational workflows is vital. A few questions/suggestions for the authors follow:

- To what extent can the most prominent histochemical changes occurring post-bevacizumab treatment be captured by m/z range and other experimental settings studied in this untargeted MSI experiment? Since it used a limited mass range, certain important changes (e.g., in cell-surface protein expression, lipid membrane composition, etc.) may not have been measured. The authors may have addressed this question in their cited previous work, but it would be helpful to provide some additional context.
- Since the focus of this paper is methodology, evaluation of the approach against known ground-truths is critical. In that regard, the efforts of the authors in developing a synthetic dataset and evaluating the methods on it is appreciated. There are a few ways that this assessment could be expanded to provide additional information about the robustness of the workflow. For example, Additional File 3 includes plots showing the synthetic data and some of its characteristics, but to what extent do the statistical properties of the synthetic data compare with those of real MSI datasets? The SL method was recommended, but how sensitive is it to the selection of the weight matrix? If it is sensitive, are there any recommendations for selecting the weight matrix based on data characteristics? When bridging to the experimental data, has the method been tested on MSI datasets (including synthetic ones) with available complementary ground-truth labeling to help evaluate the extent to which identified clusters map to known differences? It is mentioned that peaks that were present in less than 20% of the tissue were removed to focus on more common ions. To place this 20% cutoff in context, what was the coverage area of the clusters identified? It seems possible that this step may omit significant portions of tissue heterogeneity. For future applications of this workflow, how should this cutoff threshold be selected? Overall, how robust are the results/workflow recommendations to the choices of distance metric and clustering index?
- Given the dose of the drug administered, how much exposure within the tumor is expected based on pharmacology, and how might this affect the output? It would also help to provide more explanation of Figure S1 in Additional File 7. In it, the concentration (units undefined) of the drug in each cluster appears to be very similar, across both cell lines and treatment arms; however, the comparison between

the clusters and the LISA maps appears to suggest differently. Also, interpretation of the LISA maps of drug exposure in Figure 3 and the data in Table 1 in terms of histology is briefly in the Discussion, but it would be helpful to expand on this.

Methods

Are the methods appropriate to the aims of the study, are they well described, and are necessary controls included? Choose an item.

Conclusions

Are the conclusions adequately supported by the data shown? Choose an item.

Reporting Standards

Does the manuscript adhere to the journal's guidelines on [minimum standards of reporting?] Choose an item.

Choose an item.

Statistics

Are you able to assess all statistics in the manuscript, including the appropriateness of statistical tests used? Choose an item.

Quality of Written English

Please indicate the quality of language in the manuscript: Choose an item.

Declaration of Competing Interests

Please complete a declaration of competing interests, considering the following questions:

- Have you in the past five years received reimbursements, fees, funding, or salary from an organisation that may in any way gain or lose financially from the publication of this manuscript, either now or in the future?
- Do you hold any stocks or shares in an organisation that may in any way gain or lose financially from the publication of this manuscript, either now or in the future?
- Do you hold or are you currently applying for any patents relating to the content of the manuscript?
- Have you received reimbursements, fees, funding, or salary from an organization that holds or has applied for patents relating to the content of the manuscript?
- Do you have any other financial competing interests?
- Do you have any non-financial competing interests in relation to this paper?

If you can answer no to all of the above, write 'I declare that I have no competing interests' below. If your reply is yes to any, please give details below.

I declare that I have no competing interests.

I agree to the open peer review policy of the journal. I understand that my name will be included on my report to the authors and, if the manuscript is accepted for publication, my named report including any attachments I upload will be posted on the website along with the authors' responses. I agree for my report to be made available under an Open Access Creative Commons CC-BY license (http://creativecommons.org/licenses/by/4.0/). I understand that any comments which I do not wish to be included in my named report can be included as confidential comments to the editors, which will not be published.

Choose an item.

To further support our reviewers, we have joined with Publons, where you can gain additional credit to further highlight your hard work (see: https://publons.com/journal/530/gigascience). On publication of this paper, your review will be automatically added to Publons, you can then choose whether or not to claim your Publons credit. I understand this statement.

Yes Choose an item.
